# Cobalt-Catalyzed Carbonylative Conversion of Unactivated Alkyl Chlorides

**DOI:** 10.1021/jacs.6c03507

**Published:** 2026-03-30

**Authors:** Chao Xu, Yuanrui Wang, Guang Zeng, Xiao-Feng Wu

**Affiliations:** † Dalian National Laboratory for Clean Energy, Dalian Institute of Chemical Physics, Chinese Academy of Sciences, 116023 Dalian, Liaoning, China; ‡ University of Chinese Academy of Sciences, 101408 Beijing, China; § Leibniz-Institut für Katalyse e.V., Albert-Einstein-Straβe 29a, 18059 Rostock, Germany; ∥ Key Laboratory of Catalysis, Dalian Institute of Chemical Physics, Chinese Academy of Sciences, 116023 Dalian, Liaoning, China

## Abstract

Unactivated alkyl chlorides are ideal but underutilized building blocks in carbonylation due to the high strength of C–Cl bonds and the limitations of existing transition-metal catalysis. Herein, we describe a Salen-cobalt-catalyzed carbonylative transformation of unactivated alkyl chlorides under mild conditions, enabled by the supernucleophilic Co­(I) center that efficiently promotes S_N_2-type C–Cl activation. This system accommodates sterically hindered nucleophiles and proceeds through a well-supported radical-involved organocobalt catalytic cycle, providing a general platform for carbonylation of challenging alkyl chlorides.

## Graphical abstract

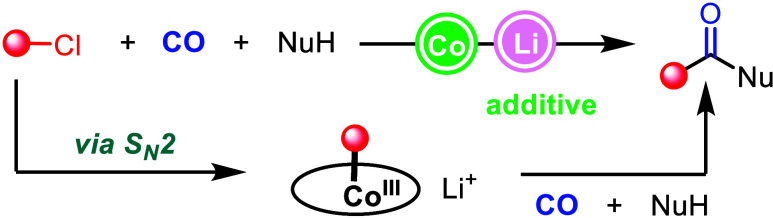

## Introduction

Unactivated alkyl halides represent extremely important alkyl building blocks, as evidenced by their widespread application in cross-coupling reactions.^1^ Alkyl chlorides are particularly attractive due to their superior stability, ready availability, and lower toxicity (Figure 1A).^2^ Despite these advantages, the high bond dissociation energy of C–Cl bonds poses a significant challenge to their activation. Consequently, cross-coupling reactions employing alkyl chlorides remain considerably underdeveloped compared to those using alkyl bromides and iodides.^3^

**Figure 1:**
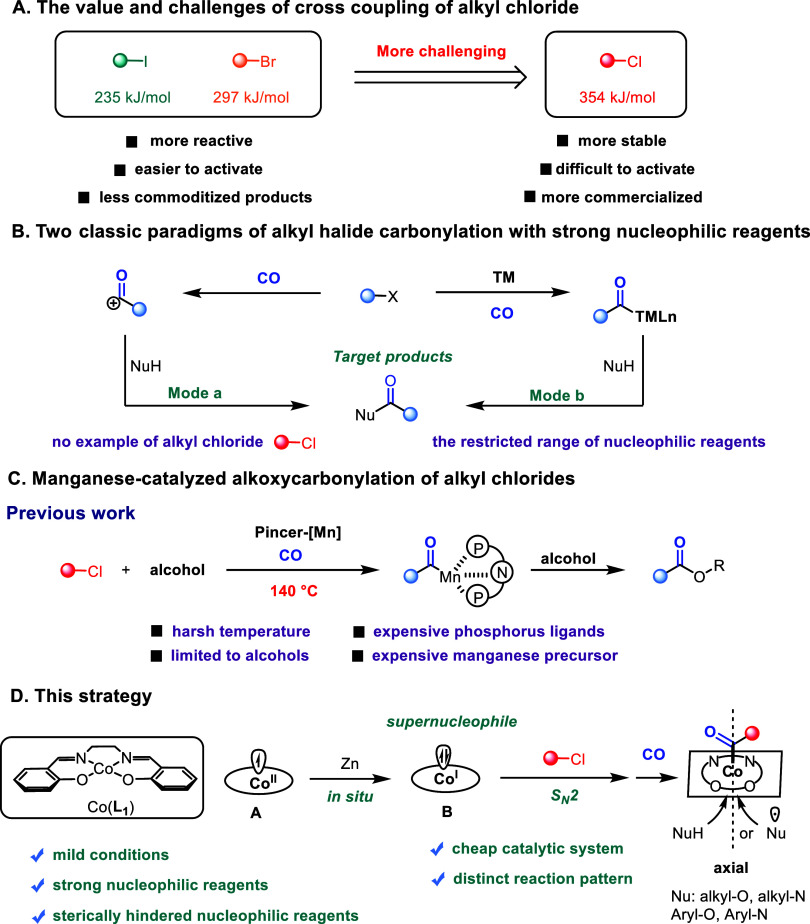
Background and Synopsis of Current Work.

The carbonylative transformation of alkyl halides with strong nucleophiles enables the construction of diverse carbonyl-containing compounds (Figure 1B). Two classic reaction paradigms have been established to date: Mode a: The reaction proceeds via an acyl cation intermediate (acyl chlorides and acyl fluorides are included in this model.).^4^ Owing to the high electrophilicity of this intermediate, the process is compatible with a broad range of nucleophiles. However, this paradigm is almost exclusively limited to alkyl iodides and bromides with no precedent for alkyl chlorides. Notably, the carbonylative transformation of alkyl chlorides was first successfully achieved through Mode b: The nucleophile coordinates to the transition-metal center, and the target product is formed via reductive elimination from a high-valent metal complex.^5^ This approach is highly ligand-dependent and typically requires sterically bulky phosphine ligands, which often impede substrate recognition, especially for sterically hindered nucleophiles.

Previously, our group achieved the direct activation of C­(sp^3^)–Cl bonds at 140 °C using a tridentate PNP manganese complex (Figure 1C).^6^ Nevertheless, this protocol suffers from several limitations: the oxidative addition of C­(sp^3^)–Cl to manganese requires overcoming an extremely high bond energy, thus necessitating elevated temperatures, which, in turn, restrict the use of more reactive nucleophiles due to competing and undesired nucleophilic substitution that erodes reaction selectivity. Furthermore, although manganese is an earth-abundant metal, the required manganese precursors and phosphine ligands are prohibitively expensive. Finally, the bulky phosphine ligands also hinder the reactivity of sterically hindered nucleophiles.

A survey of the literatures reveals that Salen-type cobalt catalysts offer a promising solution.^7^ The highest occupied molecular orbital in Salen-Co­(I) is the weakly antibonding d_*z*_^2^ orbital, whose directional character and high charge density render the metal center strongly nucleophilic along the axis perpendicular to the molecular plane.^8^ This supernucleophilic character allows the cobalt center to react with alkyl chlorides preferentially over common nucleophiles (Figure 1D).^9^ Furthermore, the transfer of electron density into the C­(sp^3^)-Cl antibonding orbital weakens the C–Cl bond, enabling facile bond cleavage under mild conditions. On the other hand, Salen-type ligands possess favorable planarity, allowing nucleophiles to readily coordinate to the metal center from the axial position, which favors the participation of sterically hindered substrates. Notably, certain substrates with low oxidation potentials can engage in coordination as radical species, and the quantum tunneling effect of electrons further enhances the reactivity of the sterically congested compounds.

## Results and Discussion

Our studies commence with the alkoxy carbonylation of primary alkyl chloride **1a** and thiophenol **2a** (Table 1). Our inexpensive and stable catalytic system consists of Co­(**L**_**1**_) (salen-cobalt), a catalytic amount of zinc powder, and DMAc as the solvent. Based on previous observations that additives or electrolytes can stabilize organocobalt species, 1.0 equiv of LiCl was selected as the additive. When sodium carbonate was used as the base, the target product was detected in 12% yield (Table 1, entry 1). A series of solvents were tested: only MeCN (Table 1, entry 2) showed a slightly reduced yield (8%), while the other tested solvents (Table 1, entry 3) completely suppressed the reaction, which may be related to the solubility of LiCl in the solution. (For more details, see Supporting Information). Importantly, the base plays a crucial role in the reaction; using DBU significantly enhances the reaction yield, reaching 88% (Table 1, entries 4–5). Furthermore, increasing the DBU loading to 2.0 equiv leads to near-complete conversion to the target product (98% yield; Table 1, entry 6). Notably, noncarbonylation product was detected as the main side reaction during the optimization process.

**Table 1:**
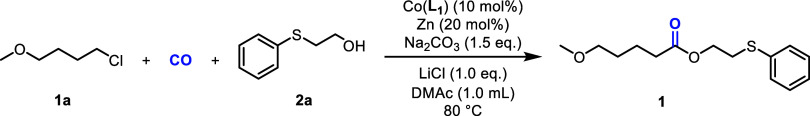
Optimization of Reaction Conditions^a^

entry	deviation from standard	yield^b^ [%]
1	none	12
2	MeCN	8
3	THF, toluene, PhCl, PhCF_3_	0
4	DBU	88
5	NEt_3_, pyridine, K_2_CO_3_, KO^*t*^Bu	10–43
**6**	**DBU (2.0 equiv)**	**98 (89** ^c^ **)**
7	w/o LiCl	25
8	LiCl (0.4 equiv)	98
9	50 °C	71
10	30 °C	53
11	5–6 bar	98
12	1 bar	56
13	w/o [Co]	0
14	w/o Zn	0
15	Co(OAc)_2_·4H_2_O + **L**_**1**_	78^d^

a Reaction conditions: **1a** (0.6 mmol), **2a** (0.3 mmol), Co­(**L**_**1**_) (10 mol %), Zn (20 mol %), Na_2_CO_3_ (0.45 mmol), LiCl (0.3 mmol), CO (40 bar), 80 °C, 16 h.

b The yields were determined by GC using hexadecane as the internal standard.

c Isolated yield.

d Based on entry 6, replaced Co­(**L**_**1**_) with Co­(OAc)_2_·4H_2_O + **L**_**1**_.

Interestingly, further investigation of LiCl loading revealed that while LiCl is not strictly essential, the reaction yield drops sharply to 25% in its absence (Table 1, entry 7). However, reducing its loading to 0.4 equiv has no impact on the reaction yield (98% yield; Table 1, entry 8). Subsequently, more moderate conditions were examined. Lowering the reaction temperature decreased the reaction yield (Table 1, entries 9–10). Similarly, when the CO pressure is reduced to 5–6 bar, the yield remains high (98% yield; Table 1, entry 11), but at 1 bar, the yield is significantly reduced (56% yield; Table 1, entry 12). Control experiments performed in the absence of either Co­(L1) or the Zn reductant resulted in no product formation or conversion of the starting material (Table 1, entries 13–14). Notably, replacing Co­(**L**_**1**_) with Co­(OAc)_2_·4H_2_O and **L**_**1**_ leads to a significant drop in yield (78% yield; Table 1, entry 15).

Upon identifying a suitable catalytic system for alkyl chloride activation, we surveyed the carbonylation of a range of alcohols and phenols (Figure 2). For alcohols as substrates, they exhibit a wide functional group tolerance. The yields are good to excellent for sulfur ether (**1**), ether (**2**), alkyl group (**3**), aryl group (**4**), benzyl alcohol (**5**), heteroaryl groups (**6**), terminal alkene (**7**), internal alkene (**8**), and alkene (**9**). Secondary alcohols can also be converted to the target products with good yields (**10–11**). However, the reaction failed when *tert*-butanol was tested. For phenolic substrates, electron-donating substituents will result in higher yields (**12–14**, **17–19**), while electron-withdrawing substituents will significantly reduce the yield (**15–16**). It is worth noting that, even with a very large steric hindrance, **19** can still be separated and obtained in 89% yield.

**Figure 2:**
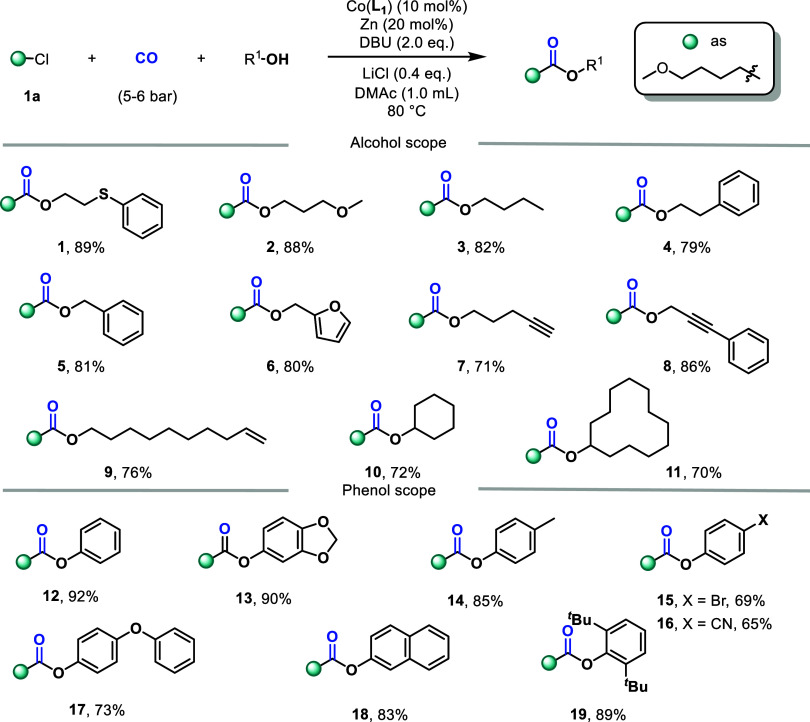
Scope of alcohols or phenols, reaction conditions: **1a** (0.6 mmol), alcohols or phenols (0.3 mmol), Co­(**L**_**1**_) (10 mol %), Zn (20 mol %), DBU (0.6 mmol), LiCl (0.4 equiv), CO (5–6 bar), 80 °C, 16 h. Isolated yield.

Subsequently, more nucleophilic amine substrates were examined (Figure 3). The target compounds **20** and **21** were obtained with yields of 76% and 88% respectively from the primary alkylamine and primary aniline. Fortunately, 8-aminoquinoline can also be obtained as the target compound **22** in a yield of 65%. The yield of secondary amines (**23**, **24**) was higher than that of primary amines. This might be due to the involvement of the alkyl group, which makes the nitrogen atom more electronegative. This protocol also applies to large steric hindrance amides, but compared to alkylamines, aromatic amines are more affected by steric hindrance. When the steric hindrance at the para position of the arylamine is increased, the yield will significantly decrease (**26**, 59% vs **25**, 81%). This suggests that alkylamines and arylamines may follow different reaction pathways. Furthermore, the weakly nucleophilic substrates carbazole and benzimidazole were investigated, and **29** and **30** were obtained with yields of 80% and 70%, respectively.

**Figure 3:**
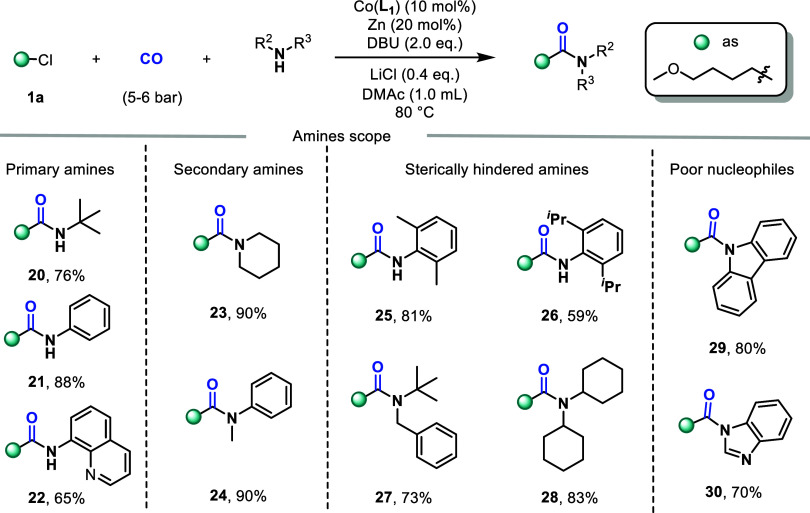
Scope of alkylamine or aniline, reaction conditions: **1a** (0.6 mmol), alkylamine or aniline (0.3 mmol), Co­(**L**_**1**_) (10 mol %), Zn (20 mol %), DBU (0.6 mmol), LiCl (0.4 equiv), CO (5–6 bar), 80 °C, 16 h. Isolated yield.

Next, we turned our attention to the scope of the alkyl chlorides (Figure 4). In general, good to excellent yields of the target products were obtained under the standard conditions. The length of the carbon chain did not affect the excellent yields (**31**, **32**). Likewise, alkyl chlorides with various functional groups, such as carbonyl (**33**), alkynyl (**34**), cyano (**35**), oxhydryl (**36**), TMS (trimethylsilyl, **37**) and chlorine (**38**) were all compatible. Interestingly, for substrates with two reaction sites, simply controlling the amount of the nucleophilic reagent can lead to the highly selective formation of a single carbonylation product, (**38**, 90%).

**Figure 4:**
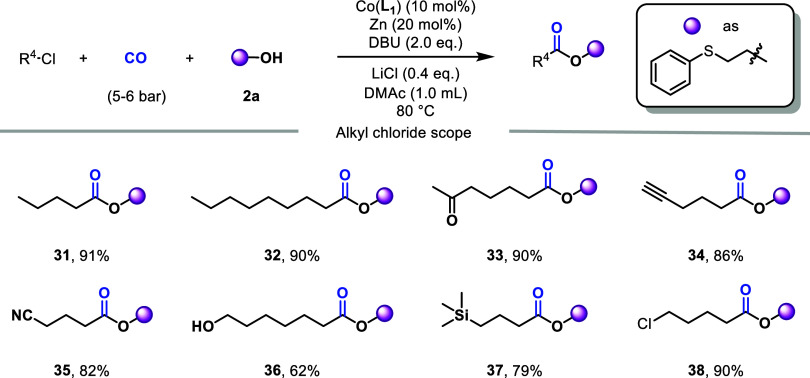
Scope of alkyl chlorides, reaction conditions: alkyl chloride (0.6 mmol), **2a** (0.3 mmol), Co­(**L**_**1**_) (10 mol %), Zn (20 mol %), DBU (0.6 mmol), LiCl (0.4 equiv), CO (5–6 bar), 80 °C, 16 h. Isolated yield.

When the substrate was extended to secondary alkyl chlorides, the target product could not be obtained under the standard conditions. We therefore conducted a systematic screening of additives and catalysts. First, we evaluated a series of lithium salts and electrolytes (Figure 5a). The results indicate that these additives exert a dual effect: on one hand, the cations stabilize the organocobalt intermediates; on the other hand, the anions influence the efficiency of nucleophilic substitution through coordination to the metal center. For primary alkyl chlorides, the S_N_2 nucleophilic substitution proceeds relatively rapidly due to the lower steric hindrance. Thus, to improve the reaction yield, it is sufficient to enhance the stability of the alkylcobalt intermediate, and the nature of the additive anion is not a critical factor. In contrast, for secondary alkyl chlorides, it is necessary not only to stabilize the organocobalt intermediates but also to accelerate the rate of the initial S_N_2 substitution step. In the case of LiCl, the lithium ions stabilize the organocobalt species but the chloride ions coordinate too tightly to the cobalt center, thereby reducing the activity for nucleophilic substitution. Bromide and iodide ions, however, possess electron-donating capabilities, which increase the electron density at the cobalt center and facilitate the S_N_2 reaction. In contrast, ClO_4_^–^, TfO^–^, and BF_4_^–^, due to their large steric bulk, neither promote nor restrict the substitution step, and their beneficial effect primarily stems from Li^+^-mediated stabilization of the organocobalt intermediates. Consequently, the yields obtained with these additives are close to but lower than those achieved with LiBr and LiI. Finally, the results show that other electrolyte cations can also stabilize the organocobalt intermediates, but they are less effective than Li^+^.

**Figure 5:**
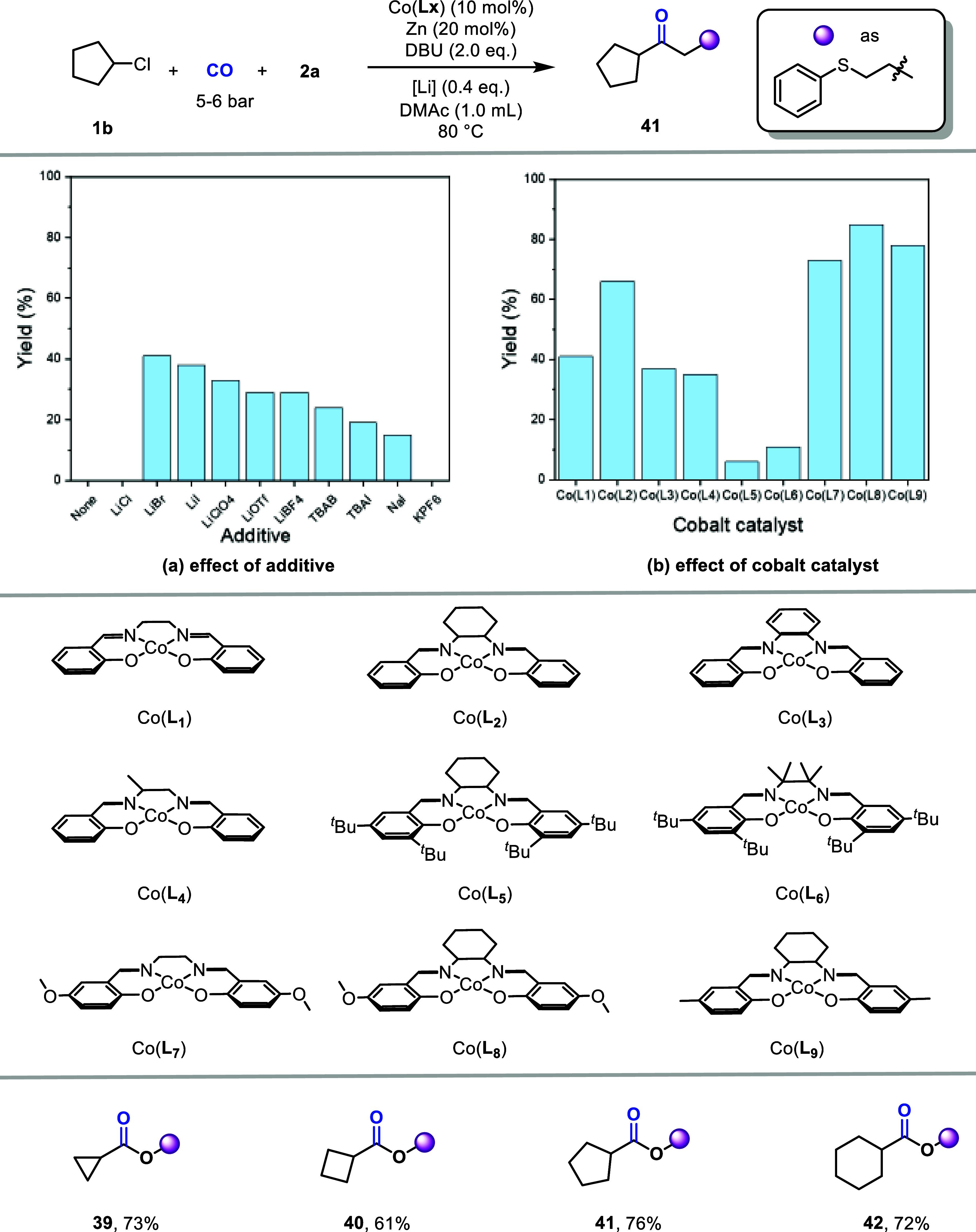
Effect of additives and catalysts of 2° alkyl chlorides, Reaction conditions: **1b** (0.6 mmol), **2a** (0.3 mmol), Co­(**L**_**8**_) (10 mol %), Zn (20 mol %), DBU (0.6 mmol), LiBr (0.4 equiv), CO (5–6 bar), 80 °C, 16 h. The yields were determined by GC using hexadecane as the internal standard.

To further accelerate the rate of nucleophilic substitution, we attempted to increase the electron density at the metal center through ligand modification (Figure 5b), with the aim of promoting the nucleophilic substitution step. Preliminary results with ligands **L**_**1**_–**L**_**4**_ show that the alkyl substituents on the ligand influence nucleophilic substitution through both electronic and steric effects and that the overall reactivity reflects a combination of these two factors. Among them, **L**_**2**_ (cyclohexyl) exhibits a stronger electron-donating ability than **L**_**1**_, and the increase in steric hindrance imposed by its cyclic structure is moderate. Although the planar phenyl ring in **L**_**3**_ introduces little steric hindrance, it does not significantly enhance the nucleophilicity of the catalyst. Ligands **L**_**4**_–**L**_**6**_ further illustrate the impact of steric hindrance on nucleophilic substitution. Subsequently, we modified the phenyl ring with substituents (**L**_**7**_–**L**_**8**_). Gratifyingly, the desired trend was observed: electron-donating groups on the phenyl ring enhanced the nucleophilic activity of the catalyst and improved the reaction yield. With LiBr as the additive and Co­(**L**_**8**_) as the catalyst, we successfully expanded the substrate scope to include secondary alkyl chlorides, affording the corresponding products **39–42** in satisfactory yields.

Importantly, our carbonylation allowed incorporation of the ester fragment into bioactive molecules (Figure 6) such as the Vitamin E (**43**), Estrone (**44**), Mannofuranose (**45**) and Testosterone (**46**). When 1,4-dichlorobutane **1c** was used as the substrate and 3.0 equiv of methanol are added, the double carbonyl compound **47** can be obtained, which can serve as the precursor for nylon-6,6.

**Figure 6:**
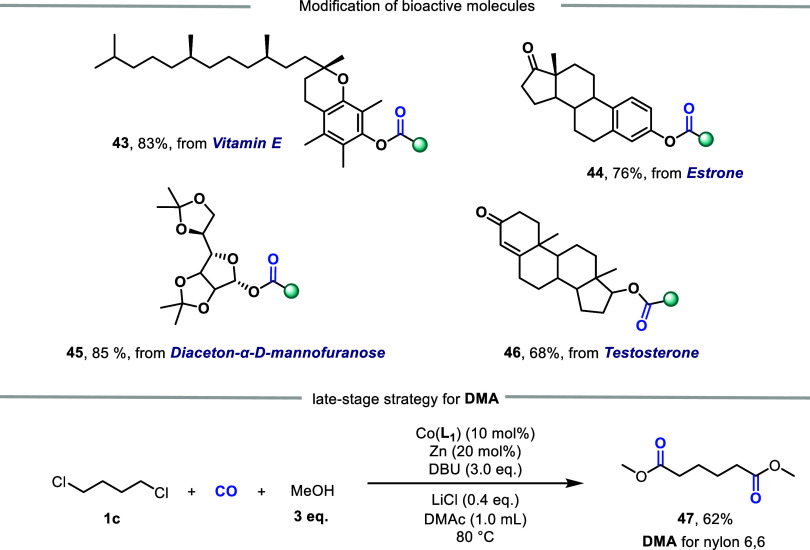
Modification of bioactive molecules and late-strategy for DMA. DMA: Dimethyl Adipate.

To elucidate the mechanism of this cobalt-catalyzed carbonylation, we conducted several control experiments (Figure 7). Under the standard conditions, the addition of 1,1-diphenylethylene (1,1-DPE, a radical trapping agent) to the model reaction resulted in a significantly decreased yield of the target product **1** (8%), while the radical capture product **48** was isolated in 18% yield (Figure 7a). Furthermore, when 6-chlorohexadiene **1d** was employed as the substrate, both acyclic product **49** and cyclic product **50** were formed (Figure 7b-I). Notably, the ratio of acyclic to cyclic product remained essentially constant across different cobalt catalyst loadings (Figure 7b-I, inset). This observation supports the involvement of a carbon-centered radical, which might be generated from the alkylcobalt­(III) intermediate via homolytic cleavage of the C–Co bond. This radical can undergo intramolecular cyclization (or ring-opening) before recombination with the cobalt center in a radical cage-rebound process.^10^ When cyclopropylmethyl chloride **1e** was used as the substrate, the ring-opening product **51** was obtained in 20% yield (Figure 7b-II), providing further evidence of the intermediacy of a carbon-centered radical.

**Figure 7:**
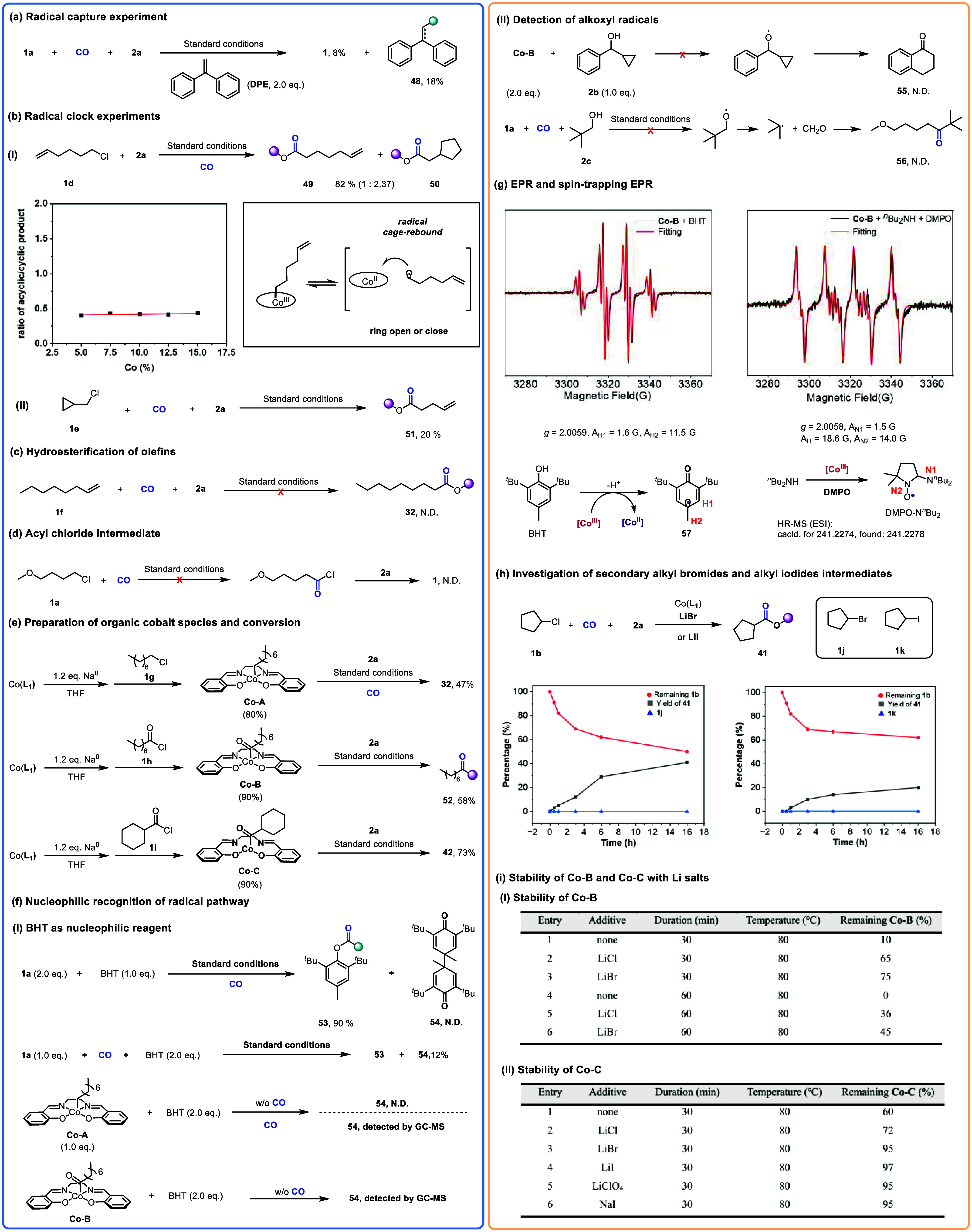
Mechanistic studies. (a) Alkyl radical capture. (b) Radical clock experiments, (I) radical cyclization experiment, (II) radical ring open. (c) Hydroesterification of olefins. (d) Acyl chloride intermediate. (e) Preparation of organic cobalt species and conversion. (f) Nucleophilic recognition of radical pathway. (I) BHT as nucleophilic reagent. (II) Detection of alkoxyl radicals. (g) EPR and spin-trapping EPR. (h) Investigation of secondary alkyl bromides and alkyl iodides intermediates. (i) Stability of Co–B and Co–C with Li salts. (I) Stability of **Co–B**, (II) Stability of **Co–C**.

The possibility of a hydroesterification^11^ pathway was excluded, as subjecting alkene **1f** to the standard conditions did not yield the corresponding ester **32** (Figure 7c). Similarly, the intermediacy of an acyl chloride^4b^ was ruled out, as no target product **1** was detected when **1a** was treated under a CO atmosphere followed by the addition of **2a** (Figure 7d).

Subsequently, a series of well-defined alkylcobalt (**Co-A**) and acyl-cobalt (**Co–B**, **Co–C**) complexes were prepared and fully characterized (Figure 7e). In the ^13^C NMR analysis, the signal for the carbon atom directly bonded to cobalt was not observed. This is attributed to the direct bonding of the carbon to the quadrupolar nucleus ^59^Co (100% natural abundance, I = 7/2, Q = 0.42), which leads to extremely fast T1 relaxation and significant line broadening, rendering the signal undetectable.^12^ When these organocobalt complexes were employed as precatalysts or stoichiometric precursors under the standard reaction conditions, the corresponding products **32** (47%), **52** (58%), and **42** (73%) were isolated in good yields. This result demonstrates that these alkylcobalt and acyl-cobalt species are competent intermediates in the catalytic carbonylation cycle.

The performance of bulky phenol (**19**) and bulky aniline (**25**–**28**) under the standard conditions is notably different, which suggests that distinct reaction pathways may be operative for these nucleophiles. Notably, BHT acts not only as a nucleophilic substrate but also as a well-known radical scavenger. When BHT was used at 2.0 equiv under standard conditions, the dimeric product **54** was isolated in 12% yield (Figure 7f-I). This observation indicates that phenols can be oxidized to the corresponding phenoxyl radical under the reaction conditions. Further control experiments revealed that the presence of CO is essential for this oxidation, and only the organocobalt species generated under catalytic conditions are capable of oxidizing phenol to its radical. To probe the potential involvement of alkoxyl radicals, control experiments were conducted using alcohols with high oxidation potentials (Figure 7f-II). The target products **55** and **56** were not detected, ruling out the intermediacy of alkoxy radicals in this transformation.

To further investigate the nucleophilic reagent conversion, electron paramagnetic resonance (EPR) experiments were conducted. The carbon radical cation (**57**) resulted from the single-electron oxidation of BHT by **Co–B** (Figure 7g, left). However, no corresponding amine radical cation from ^*n*^Bu_2_NH was detected under the same conditions using EPR. Additionally, a *N*-centered radical, which was trapped by 5,5-dimethyl-1-pyrroline *N*-oxide (DMPO), was detected in the reaction involving **Co–B**, ^*n*^Bu_2_NH and DMPO (Figure 7g, right).^13^ This DMPO-trapped *N*-centered radical was confirmed as DMPO-N^*n*^Bu_2_ using high-resolution mass spectrometry (HRMS). All the results indicate that, for phenol and alkylamine substrates, there may also exist a radical recognition pathway.

Mechanistic studies on secondary alkyl chlorides were carried out in parallel with reaction condition optimization (Figure 5). A precedent for the conversion of secondary alkyl chlorides to alkyl iodides followed by carbonylation has been reported.^14^ To probe whether a similar pathway operates here, we designed control experiments using LiBr and LiI as additives (Figure 7h). Product **41** was formed in detectable yields over different reaction times, but no brominated (**1j**) or iodinated (**1k**) intermediates were observed. Furthermore, additives such as LiClO_4_, LiOTf, and LiBF_4_ also improved the reaction yield (Figure 5a), which further confirms that the alkyl chloride substrate is not converted to other halogenated intermediates prior to the carbonylation step.

Finally, we demonstrated that these additives enhance the thermal stability of the organocobalt intermediates at elevated temperatures (Figure 7i). The acyl-cobalt complex derived from a secondary alkyl chloride (**Co–C**) exhibited a higher thermal stability than that derived from a primary alkyl chloride (**Co–B**). This is likely attributed to the greater number of alkyl substituents weakening the metal’s π-backbonding to the carbonyl ligand. We initially hypothesized that **Co–B**, with a stronger C–Co bond, could deliver a higher yield at increased reaction temperatures; however, this strategy proved unsuccessful. Nevertheless, these findings have guided our additive selection and ligand modification, as we now recognize that the yield-limiting factors for secondary alkyl chlorides are associated with the initial S_*N*_2 substitution step (Figure 5).

Based on the above results and previous reports, we propose a plausible mechanism for this carbonylation event (Figure 8).^7^ Catalysis commences with the in situ reduction of Co­(II) precatalyst **A** to active Co­(I) complex **B** using zinc powder. Subsequently, complex **B** undergoes an S_*N*_2 reaction with the alkyl chloride substrate, generating alkylcobalt­(III) intermediate **C**. This intermediate exists in equilibrium with a radical cage pair, where the C–Co bond undergoes homolytic cleavage to generate a carbon-centered radical and a Co­(II) species; this radical can undergo intramolecular rearrangement (e.g., ring-opening or ring-closing) before recombination with the cobalt center to reform **C**. Under a CO atmosphere, alkylcobalt complex **C** undergoes migratory insertion of CO to form acyl-cobalt­(III) species **D**. Subsequent reaction with the nucleophile (NuH) proceeds via two distinct pathways, depending on substrate structure and reaction conditions: (1) Path a involves single-electron transfer (SET) to form the acyl radical **E**, followed by nucleophilic capture and coordination to cobalt; (2) Path b proceeds via direct ligand exchange, where NuH displaces a ligand to form the acyl-cobalt nucleophile complex **F**. Finally, complex **F** undergoes reductive elimination to form the carbonylated product, while regenerating the active Co­(I) species **B** to re-enter the catalytic cycle. It is also worth mentioning the possibility of nucleophilic addition of nucleophile to intermediate **D** to generate the amide product and then reductive elimination of hydride from the cobalt to regenerate complex **B**.

**Figure 8:**
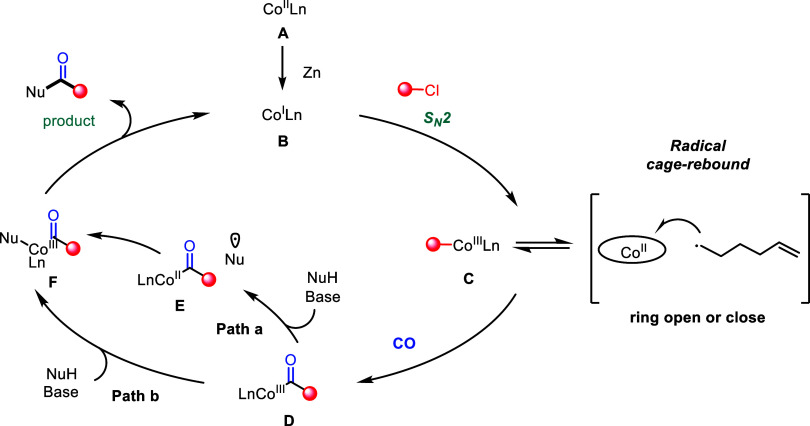
Proposed mechanism.

## Conclusions

In summary, we have developed a versatile platform for the activation of alkyl chlorides by using an inexpensive and stable Salen-cobalt catalytic system. This catalytic system fully exploits the unique steric and electronic properties of Salen–cobalt complexes, enabling the carbonylation of inert C­(sp^3^)–Cl bonds under much milder conditions and expanding the scope of nucleophilic substrates. Additives and ligands play crucial roles in this transformation, affecting the stability of organocobalt intermediates and the nucleophilicity of the metal center, respectively. We anticipate that this distinctive catalytic system will prove valuable for the cross-coupling of inert chemical bonds.

## Supplementary Material



## Data Availability

The data underlying this study are available in the published article and its Supporting Information.

## Notes

General comments, general procedure, optimization details, analytic data and NMR spectra. (PDF)
