# Supplementary material for: Cobalt-Catalyzed Carbonylative Conversion of Unactivated Alkyl Chlorides
Source: J Am Chem Soc. 2026 Mar 30;148(13):14652–61. doi: 10.1021/jacs.6c03507 (PMC13067354; doi:10.1021/jacs.6c03507)

Supporting Information

Cobalt-Catalyzed Carbonylative Conversion of Unactivated Alkyl Chlorides

Chao Xu,[a,b] Yuanrui Wang,[a] Guang Zeng,[d] and Xiao-Feng Wu[a,b,c]*

[a] Dalian National Laboratory for Clean Energy, Dalian Institute of Chemical Physics, Chinese Academy of Sciences, 116023 Dalian, Liaoning, China

[b] University of Chinese Academy of Sciences, 101408 Beijing, China

[c] Leibniz-Institut für Katalyse e. V., Albert-Einstein-Straβe 29a, 18059 Rostock, Germany

[d] Key Laboratory of Catalysis, Dalian Institute of Chemical Physics, Chinese Academy of Sciences, 116023 Dalian, Liaoning, China, Email: xiao-feng.wu@catalysis.de, xwu2020@ dicp.ac.cn

Table of Content

1. General remarks 2
2. Representative Procedure for Synthesis of Cobalt Salen Complexes 3
3. Reaction Development with Primary Alkyl Chlorides 4
4. Reaction Development with Secondary Alkyl Chlorides 8
4. General procedure for carbonylation reaction 10
   4.1 General procedure for carbonylation reaction of primary alkyl chlorides 10
   4.2 General procedure for carbonylation reaction of secondary alkyl chlorides 10
5. Later strategy: Synthesis of the nylon 6,6 precursor **DMA** (**47**) 11
6. Mechanism experiments 12
   6.1 Radical capture experiment 12
   6.2 Radical clock experiments 14
   6.3 Hydroesterification of olefins 18
   6.4 Acyl chloride intermediate[2] 18
   6.5 Synthesis of organic cobalt species 19
   6.6 Conversion of organic cobalt species 23
   6.7 Nucleophilic recognition of radical pathway 25
   6.8 EPR and spin-trapping EPR[7] 28
   6.9 Investigation of secondary alkyl bromides and alkyl iodides intermediates[8] 30
   6.10 Stability Studies of **Co-B** and **Co-C** at Elevated Temperatures With Additive[3] 31
7. Characterization data of products 33
8. Reference 47
9. Spectra of compounds 49

Experimental section

1. General remarks

All chemicals and reagents were obtained from Macklin, Bidepharm and Sigma-Aldrich, and were used without further purification. All solvents were dried by standard techniques and distilled prior to use. Column chromatography was performed on silica gel (200-300 meshes) using petroleum ether (bp. 60~90 °C), ethyl acetate and acetone as eluent. ^{1}H and ^{13}C NMR spectra were taken on Bruker AVANCE III 400 MHz or 700 MHz spectrometers and spectral data were reported in ppm relative to tetramethylsilane (TMS) as the internal standard and $CDCl_3$ or DMSO-d_6 as solvent. All coupling constants (*J*) are reported in Hz with the following abbreviations: s = singlet, d = doublet, dd = double doublet, t = triplet, dt = double triplet, q = quatriplet, m = multiplet, br = broad. Gas chromatography (GC) analyses were performed on an Agilent HP-7890A instrument with a FID detector and HP-5 capillary column (polydimethylsiloxane with 5% phenyl groups, 30 m, 0.32 mm i.d. 0.25 µm film thickness) using argon as carrier gas. Gas chromatography mass spectrometer (GC-MS) analyses were performed on a Shimadzu QP2020 NX instrument. High resolution mass spectra (HRMS) were recorded on Agilent 8890-7250 and Agilent Q-TOF 6540. Unless otherwise noted, all reactions were carried out under carbon monoxide (CO) or nitrogen atmosphere. Because of the high toxicity of carbon monoxide, all the reactions should be performed in an autoclave. The laboratory should be well equipped with a CO detector and alarm system.

2. Representative Procedure for Synthesis of Cobalt Salen Complexes

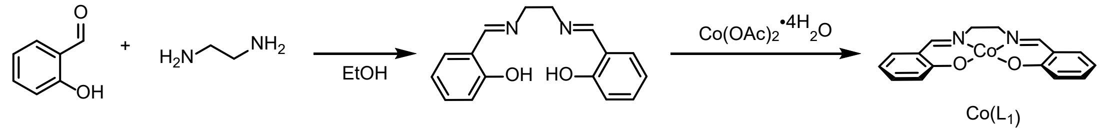

Some salen cobalt complexes can be purchased commercially. Here, synthesis was carried out using Co(L_1) as an example. Salicylaldehyde (1.6 mL), 95% ethanol (80 mL), and ethylenediamine (0.5 mL) were sequentially added to a three-necked flask. The mixture was stirred at room temperature for 4–5 min, resulting in the formation of bright yellow flaky crystals of bis(salicylaldehyde)ethylenediamine. $Co(OAc)_2 \cdot 4H_2O$ (1.9 g) was dissolved in hot water (15 mL), and the resulting solution was transferred to a dropping funnel. The nitrogen cylinder was then opened, the gas pressure and flow regulator were adjusted, and the flask was purged with nitrogen for 3–5 min to ensure complete removal of air. The reflux condenser was subsequently activated, and the solution was heated to gentle boiling. Once the bright yellow flaky crystals had completely dissolved, the cobalt (II) acetate solution was added to the flask, immediately yielding a brown gelatinous precipitate. The mixture was stirred under gentle boiling for 40–50 min, during which the brown precipitate gradually transformed into dark red crystals. The resulting suspension was cooled to room temperature, filtered under reduced pressure, washed thoroughly, and dried under vacuum. The final product was weighed, and the yield was calculated accordingly.

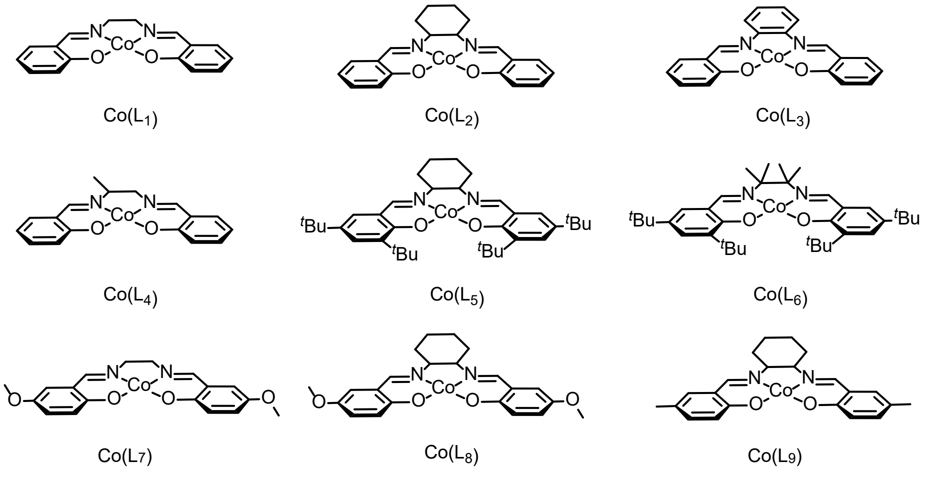

Figure S1: The Cobalt catalyst used in this article

3. Reaction Development with Primary Alkyl Chlorides

Table 1: The effect of solvents.

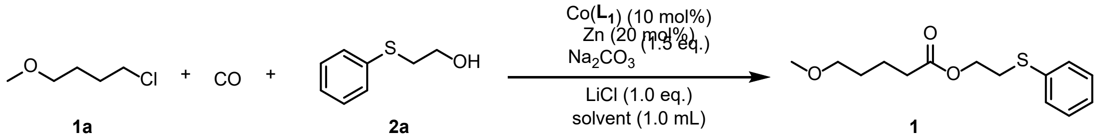

Entry	Solvent	Yield (%)[a]
**1**	**DMAc**	**12**
2	THF	0
3	Toluene	0
4	$PhCF_3$	0
5	PhCl	0
6	DME	0
7	MeCN	8
8	Dioxane	0

Reaction conditions: **1a** (2.0 eq.), **2a** (0.3 mmol), Co(**L**$_1$) (10 mol%), Zn (20 mol%), Na_2CO_3 (1.5 eq.), LiCl (1.0 eq.), solvent (1.0 mL), CO (40 bar), 80 °C, 16 h. [a]Yields were determined by GC with dodecane as an internal standard.

Table 2: The effect of bases.

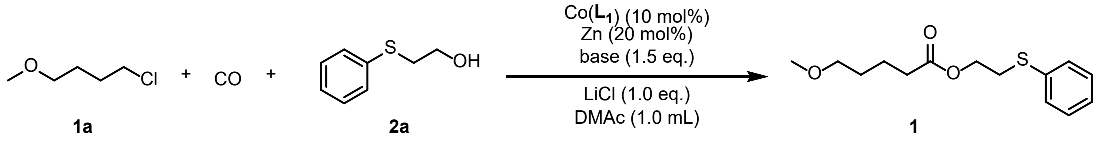

Entry	Base	Yield (%)[a]
1	w/o base	12
2	Et_3N	12
**3**	**DBU**	**88**
4	K_2CO_3	12
5	Na_3PO_4	12
6	K_3PO_4	10
7	Cs_2CO_3	14
8	^{t}BuONa	25
9	^{t}BuOK	43
10	DIPEA	12
11	Pyridine	11

Reaction conditions: **1a** (2.0 eq.), **2a** (0.3 mmol), Co(**L**$_1$) (10 mol%), Zn (20 mol%), base (1.5 eq.), LiCl (1.0 eq.), DMAc (1.0 mL), CO (40 bar), 80 °C, 16 h. [a]Yields were determined by GC with dodecane as an internal standard.

Table 3: The effect of equivalent of base.

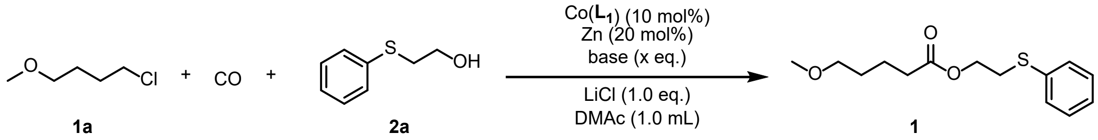

Entry	DBU (x eq.)	Yield (%)[a]
1	1.5	88
2	1.6	90
3	1.8	93
**4**	**2.0**	**98**

Reaction conditions: **1a** (2.0 eq.), **2a** (0.3 mmol), Co(**L**$_1$) (10 mol%), Zn (20 mol%), DBU (x eq.), LiCl (1.0 eq.), DMAc (1.0 mL), CO (40 bar), 80 °C, 16 h. [a]Yields were determined by GC with dodecane as an internal standard.

Table 4: The effect of equivalent of LiCl.

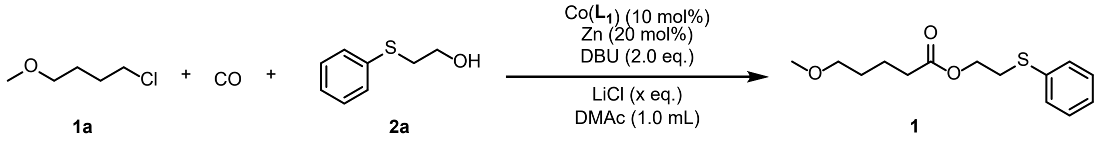

Entry	LiCl (x eq.)	Yield (%)[a]
1	w/o LiCl	25
2	0.2	93
**3**	**0.4**	**98**
4	0.6	98
5	0.8	98
6	1.0	98
7	1.2	92
8	1.4	92

Reaction conditions: **1a** (2.0 eq.), **2a** (0.3 mmol), Co(**L**$_1$) (10 mol%), Zn (20 mol%), DBU (2.0 eq.), LiCl (x eq.), DMAc (1.0 mL), CO (40 bar), 80 °C, 16 h. [a]Yields were determined by GC with dodecane as an internal standard.

Table 5: The effect of equivalent of temperature.

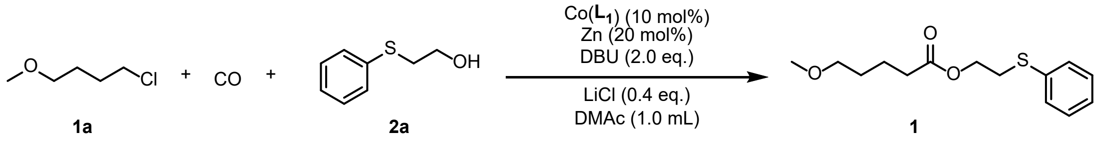

Entry	Temperature °C	Yield (%)[a]
1	30	53
2	40	62
3	50	71
4	60	79
**6**	**80**	**98(89)**[b]

Reaction conditions: **1a** (2.0 eq.), **2a** (0.3 mmol), Co(**L**$_1$) (10 mol%), Zn (20 mol%), DBU (2.0 eq.), LiCl (0.4 eq.), DMAc (1.0 mL), CO (40 bar), x °C, 16 h. [a]Yields were determined by GC with dodecane as an internal standard. [b]Isolated yields.

Table 6: The effect of the pressure of **CO**.

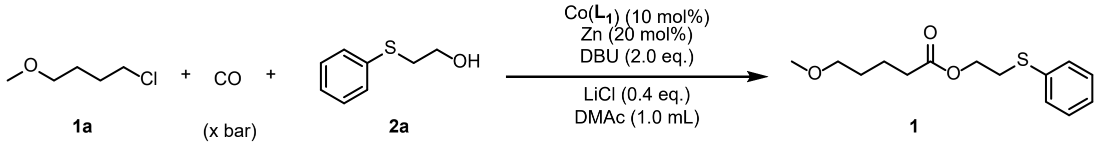

Entry	pressure (x bar)	Yield (%)[a]
1	40	98
2	30	98
3	20	98
4	10	98
**5**	**5-6**	**98**
6	1	56

Reaction conditions: **1a** (2.0 eq.), **2a** (0.3 mmol), Co(**L**$_1$) (10 mol%), Zn (20 mol%), DBU (2.0 eq.), LiCl (0.4 eq.), DMAc (1.0 mL), CO (x bar), 80 °C, 16 h. [a]Yields were determined by GC with dodecane as an internal standard.

Table 7: Other control experiments.

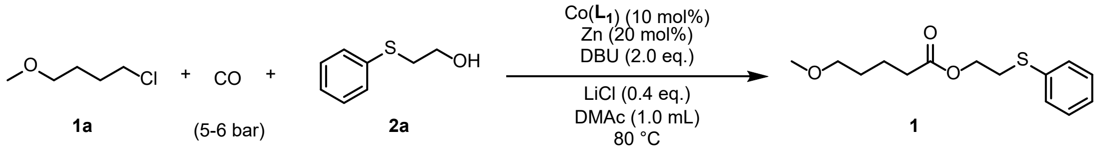

Entry	Deviation from above condition	Yield (%)[a]
1	w/o Zn	0
2	Co(OAc)$_2$•4H$_2$O + **L**$_1$	78
3	Co(OAc)$_2$•4H$_2$O + **L**$_2$	80

Reaction conditions: **1a** (2.0 eq.), **2a** (0.3 mmol), Co(**L**$_1$) (10 mol%), Zn (20 mol%), DBU (2.0 eq.), LiCl (0.4 eq.), DMAc (1.0 mL), CO (5-6 bar), 80 °C, 16 h. [a]Yields were determined by GC with dodecane as an internal standard.

4. Reaction Development with Secondary Alkyl Chlorides

Table 8: The effect of equivalent of lithium salts.

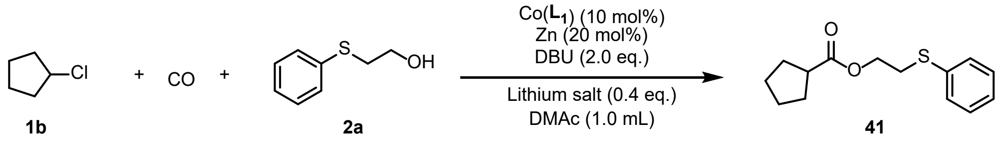

Entry	lithium salt	Yield (%)[a]
1	w/o LiCl	trace
2	LiCl	trace
**3**	**LiBr**	**41**
4	LiI	38
5	$LiClO_4$	33
6	LiOTf	29
7	$LiBF_4$	29
8	TBAB	24
9	TBAI	19

Reaction conditions: **1b** (2.0 eq.), **2a** (0.3 mmol), Co(**L1**) (10 mol%), Zn (20 mol%), DBU (2.0 eq.), lithium salts (0.4 eq.), DMAc (1.0 mL), CO (5-6 bar), 80 °C, 16 h. [a]Yields were determined by GC with dodecane as an internal standard.

Table 9: The effect of temperature.

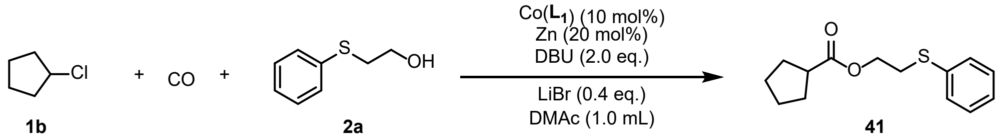

Entry	Temperature x °C	Yield (%)[a]
1	80	41
2	90	42
**3**	100	42
4	110	41
5	120	42
6	130	42
7	140	41

Reaction conditions: **1b** (2.0 eq.), **2a** (0.3 mmol), Co(**L1**) (10 mol%), Zn (20 mol%), DBU (2.0 eq.), LiBr (0.4 eq.), DMAc (1.0 mL), CO (5-6 bar), x °C, 16 h. [a]Yields were determined by GC with dodecane as an internal standard.

Table 10: The effect of equivalent of lithium salts.

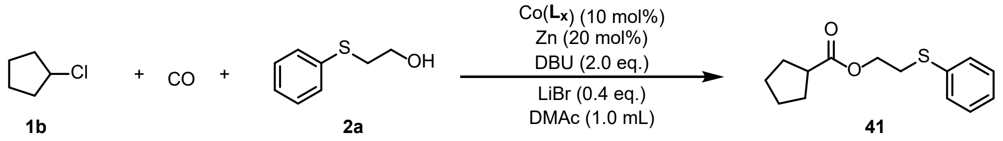

Entry	Co(**L**$_x$)	Yield (%)[a]
1	**L**$_1$	41
2	**L**$_2$	66
3	**L**$_3$	37
4	**L**$_4$	35
5	**L**$_5$	6
6	**L**$_6$	11
7	**L**$_7$	73
8	**L**$_8$	85
9	**L**$_9$	78

Reaction conditions: **1b** (2.0 eq.), **2a** (0.3 mmol), Co(**L**$_x$) (10 mol%), Zn (20 mol%), DBU (2.0 eq.), lithium salts (0.4 eq.), DMAc (1.0 mL), CO (5-6 bar), 80 °C, 16 h. [a]Yields were determined by GC with dodecane as an internal standard.

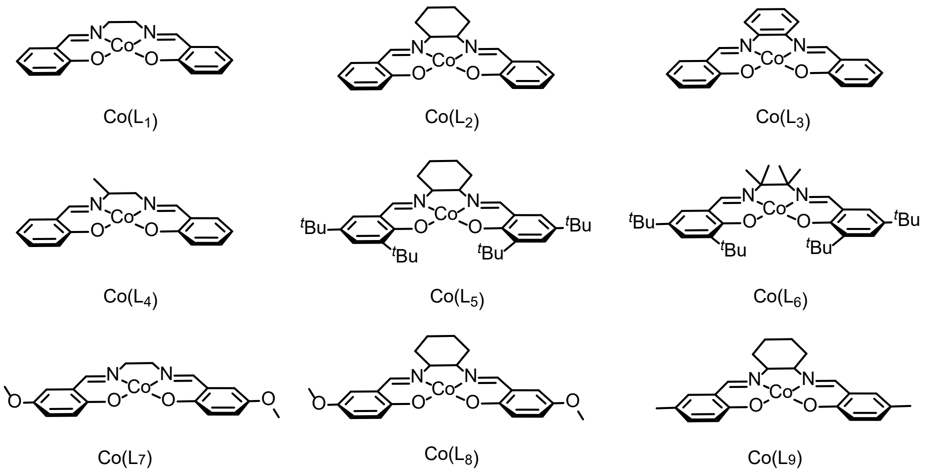

Figure S1: The Cobalt catalyst used in this article

4. General procedure for carbonylation reaction

4.1 General procedure for carbonylation reaction of primary alkyl chlorides

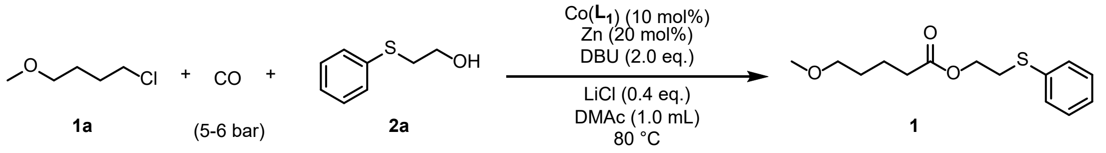

A 4 mL screw-cap vial was charged with Co(**L**$_1$) (10 mol%, 9.8 mg), Zn (20 mol%, 4.0 mg) and an oven-dried stirring bar. The vial was closed with a Teflon septum and cap and connected to the atmosphere via a needle. The vial was purged with nitrogen in a nitrogen-filled glove box, followed by the addition of LiCl (40 mol%, 5.0 mg). After capping the vial tightly, it was taken out of the glove box. Then **1a** (0.6 mmol, 80 μL), **2a** (0.3 mmol, 40 μL), DBU (0.6 mmol, 90 μL), DMAc (1.0 mL) was added with a syringe under N_2 atmosphere, the vial was moved to an alloy plate and put into a Parr 4560 series autoclave (300 mL) under N_2 atmosphere. At room temperature, the autoclave flushed with N_2 three times and CO three times and charged with 5-6 bar CO. The autoclave was placed on a heating plate equipped with a magnetic stirrer. The reaction mixture was heated to 80 °C for 16 h. After the reaction was completed, the mixture was diluted with sat. LiCl (10 mL) and extracted with EtOAc (3 x 10 mL), then the organic phase was washed with sat. brine (20 mL), dried over Na_2SO_4, filtered, and concentrated in vacuo. The crude product was purified by column chromatography to furnish the desired product.

4.2 General procedure for carbonylation reaction of secondary alkyl chlorides

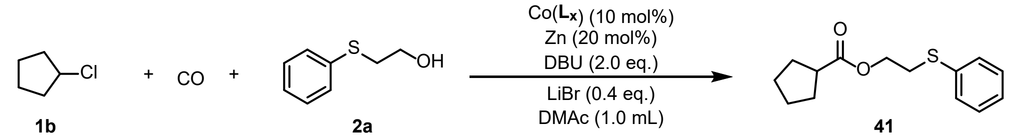

A 4 mL screw-cap vial was charged with Co(**L**$_1$) (10 mol%, 9.8 mg), Zn (20 mol%, 4.0 mg) and an oven-dried stirring bar. The vial was closed with a Teflon septum and cap and connected to the atmosphere via a needle. The vial was purged with nitrogen in a nitrogen-filled glove box, followed by the addition of LiBr (40 mol%, 11.0 mg). After capping the vial tightly, it was taken out of the

glove box. Then **1b** (0.6 mmol, 80 μL), **2a** (0.3 mmol, 40 μL), DBU (0.6 mmol, 90 μL), DMAc (1.0 mL) was added with a syringe under N_2 atmosphere, the vial was moved to an alloy plate and put into a Parr 4560 series autoclave (300 mL) under N_2 atmosphere. At room temperature, the autoclave flushed with N_2 three times and CO three times and charged with 5-6 bar CO. The autoclave was placed on a heating plate equipped with a magnetic stirrer. The reaction mixture was heated to 80 °C for 16 h. After the reaction was completed, the mixture was diluted with sat. LiCl (10 mL) and extracted with EtOAc (3 x 10 mL), then the organic phase was washed with sat. brine (20 mL), dried over Na_2SO_4, filtered, and concentrated in vacuo. The crude product was purified by column chromatography to furnish the desired product.

5. Later strategy: Synthesis of the nylon 6,6 precursor DMA (47)

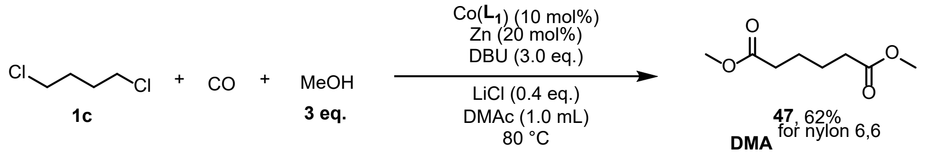

A 4 mL screw-cap vial was charged with Co(**L**$_1$) (10 mol%, 9.8 mg), Zn (20 mol%, 4.0 mg) and an oven-dried stirring bar. The vial was closed with a Teflon septum and cap and connected to the atmosphere via a needle. The vial was purged with nitrogen in a nitrogen-filled glove box, followed by the addition of LiCl (40 mol%, 11.0 mg). After capping the vial tightly, it was taken out of the glove box. Then **1c** (0.3 mmol, 36 μL), methyl alcohol (0.9 mmol, 36 μL), DBU (0.9 mmol, 135 μL), DMAc (1.0 mL) was added with a syringe under N_2 atmosphere, the vial was moved to an alloy plate and put into a Parr 4560 series autoclave (300 mL) under N_2 atmosphere. At room temperature, the autoclave flushed with N_2 three times and CO three times and charged with 5-6 bar CO. The autoclave was placed on a heating plate equipped with a magnetic stirrer. The reaction mixture was heated to 80 °C for 16 h. After the reaction was completed, the mixture was diluted with sat. LiCl (10 mL) and extracted with EtOAc (3 x 10 mL), then the organic phase was washed with sat. brine (20 mL), dried over Na_2SO_4, filtered, and concentrated in vacuso. The crude product was purified by column chromatography to furnish the desired product.

6. Mechanism experiments

6.1 Radical capture experiment

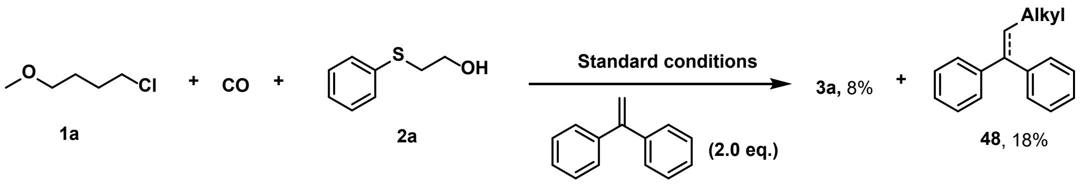

A 4 mL screw-cap vial was charged with CoSalen-I (10 mol%, 9.8 mg), Zn (20 mol%, 4.0 mg), and an oven-dried stirring bar. The vial was closed with a Teflon septum and cap and connected to the atmosphere via a needle. The vial was purged with nitrogen in a nitrogen-filled glove box, followed by the addition of LiCl (40 mol%, 5.0 mg). After capping the vial tightly, it was taken out of the glove box. Then **1a** (0.6 mmol, 80 μL), **2a** (0.3 mmol, 40 μL), 1,1-DPE (0.6 mmol, 107 μL), DBU (0.6 mmol, 90 μL), DMAc (1.0 mL) was added with a syringe under N_2 atmosphere, the vial was moved to an alloy plate and put into a Parr 4560 series autoclave (300 mL) under N_2 atmosphere. At room temperature, the autoclave flushed with N_2 three times and CO three times and charged with 5-6 bar CO. The autoclave was placed on a heating plate equipped with a magnetic stirrer. The reaction mixture was heated to 80 °C for 16 h. After the reaction was completed, the crude product was purified by column chromatography on silica gel to afford the corresponding compounds.

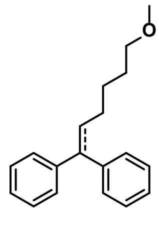

^{1}H NMR (400 MHz, CDCl$_3$) δ 7.49 – 7.06 (m, 25H), 6.07 (t, *J* = 7.2 Hz, 1.5H), 3.88 (t, *J* = 8.0 Hz, 1H), 3.33 – 3.23 (m, 12H), 2.14 (q, *J* = 7.2 Hz, 3H), 2.04 (q, *J* = 7.6 Hz, 2H), 1.66 – 1.46 (m, 8.5H), 2.16 – 1.48 (m, 2H), 1.32 – 1.22 (m, 3H).

^{13}C NMR (100 MHz, CDCl$_3$) δ 145.3, 142.8, 141.8, 140.3, 129.9, 129.8, 128.4, 128.1, 128.1, 127.9, 127.2, 126.9, 126.8, 126.0, 72.8, 72.6, 58.6, 58.6, 51.3, 35.7, 29.5, 29.5, 29.2, 27.9, 26.5, 26.2.

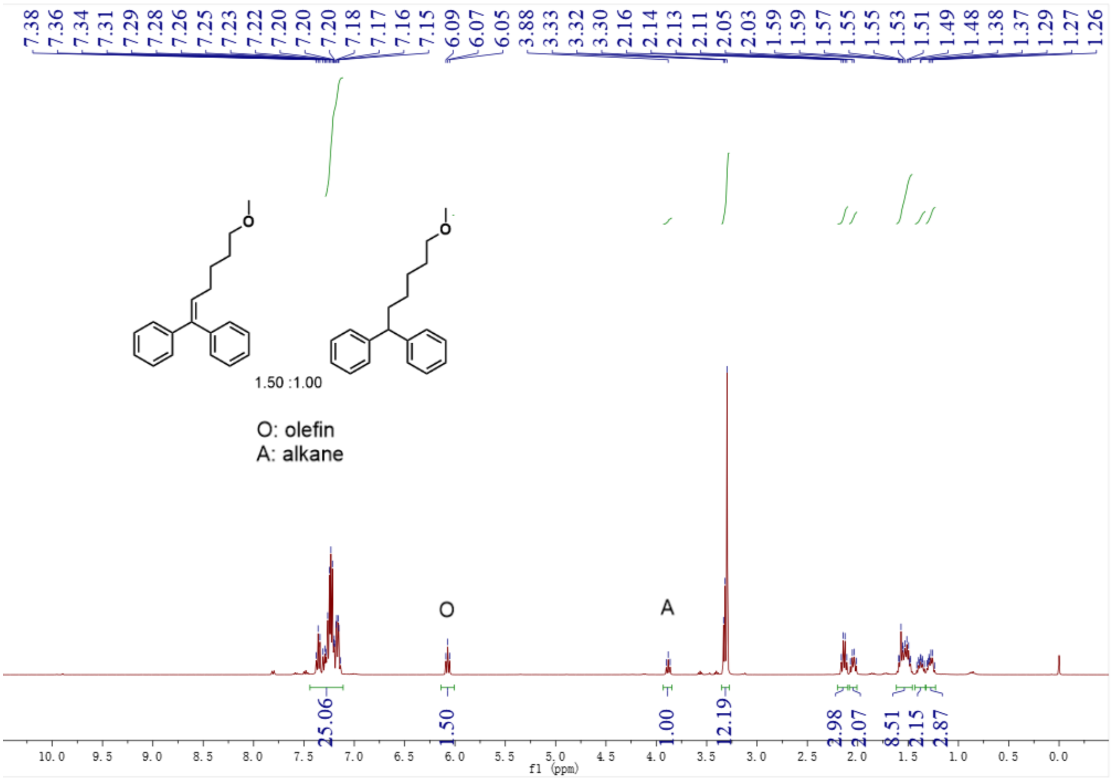
1.50 :1.00
O: olefin
A: alkane
O
A
f1 (ppm)

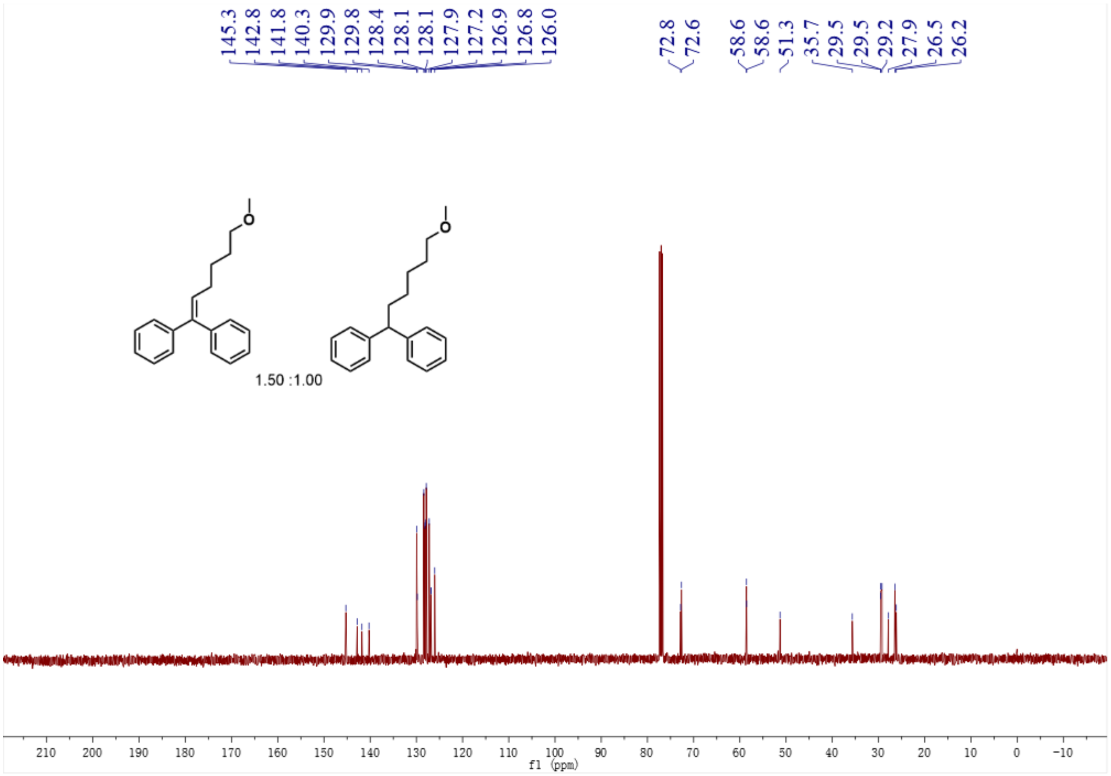
1.50 :1.00
f1 (ppm)

6.2 Radical clock experiments

Radical cyclization experiment[1]

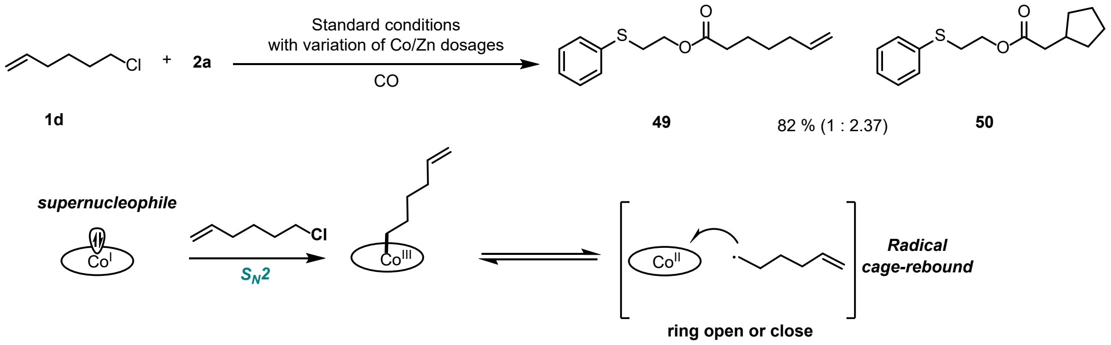

A 4 mL screw-cap vial was charged with Co(L_1) (x mol%), Zn (2x mol%), and an oven-dried stirring bar. The vial was closed with a Teflon septum and cap and connected to the atmosphere via a needle. The vial was purged with nitrogen in a nitrogen-filled glove box, followed by the addition of LiCl (40 mol%, 5.0 mg). After capping the vial tightly, it was taken out of the glove box. Then **1d** (0.6 mmol, 80 µL), **2a** (0.3 mmol, 40 µL), DBU (0.6 mmol, 90 µL), DMAc (1.0 mL) was added with a syringe under N_2 atmosphere, the vial was moved to an alloy plate and put into a Parr 4560 series autoclave (300 mL) under N_2 atmosphere. At room temperature, the autoclave flushed with N_2 three times and CO three times and charged with 5-6 bar CO. The autoclave was placed on a heating plate equipped with a magnetic stirrer. The reaction mixture was heated to 80 °C for 16 h. After the reaction was completed, the crude product was purified by column chromatography on silica gel to afford the corresponding compounds.

Table 11: The Ratio of Acyclized/Cyclized Product with Various Loading of Co(**L_1**)

Entry	Co(**L_1**) Loading (x mol%)	Combined Product Yield (%)	Ratio of Acyclized/cyclized Product[a]
1	**5.0**	78	1/2.32
2	**7.5**	81	1/2.37
3	**10.0**	82	1/2.37
4	**12.5**	83	1/2.39
9	**15.0**	83	1/2.28

[a]Ratio of acyclized to cyclized product was determined by ^{1}H NMR.

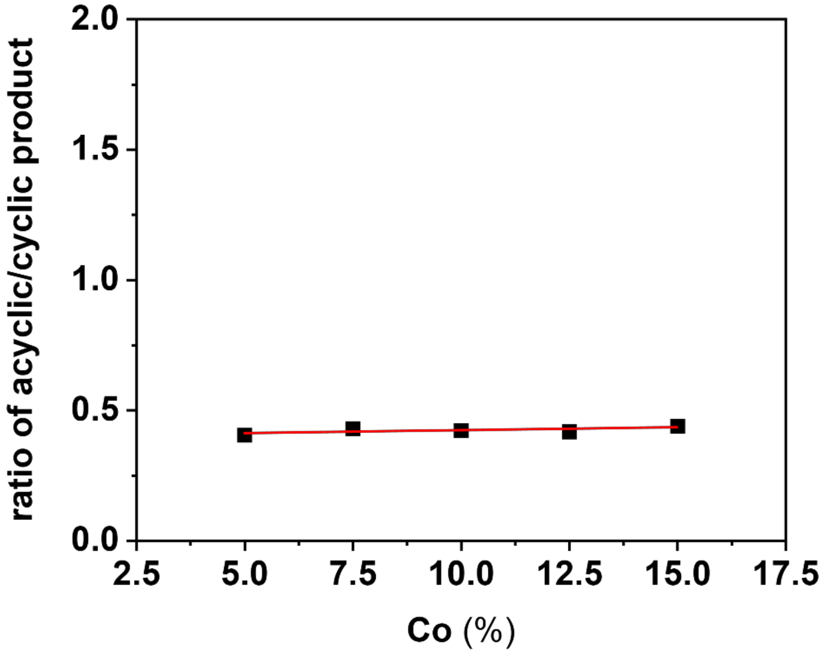

Figure S2: The Ratio of Acyclized/Cyclized Product with Various Loading of Co(**L**$_1$)

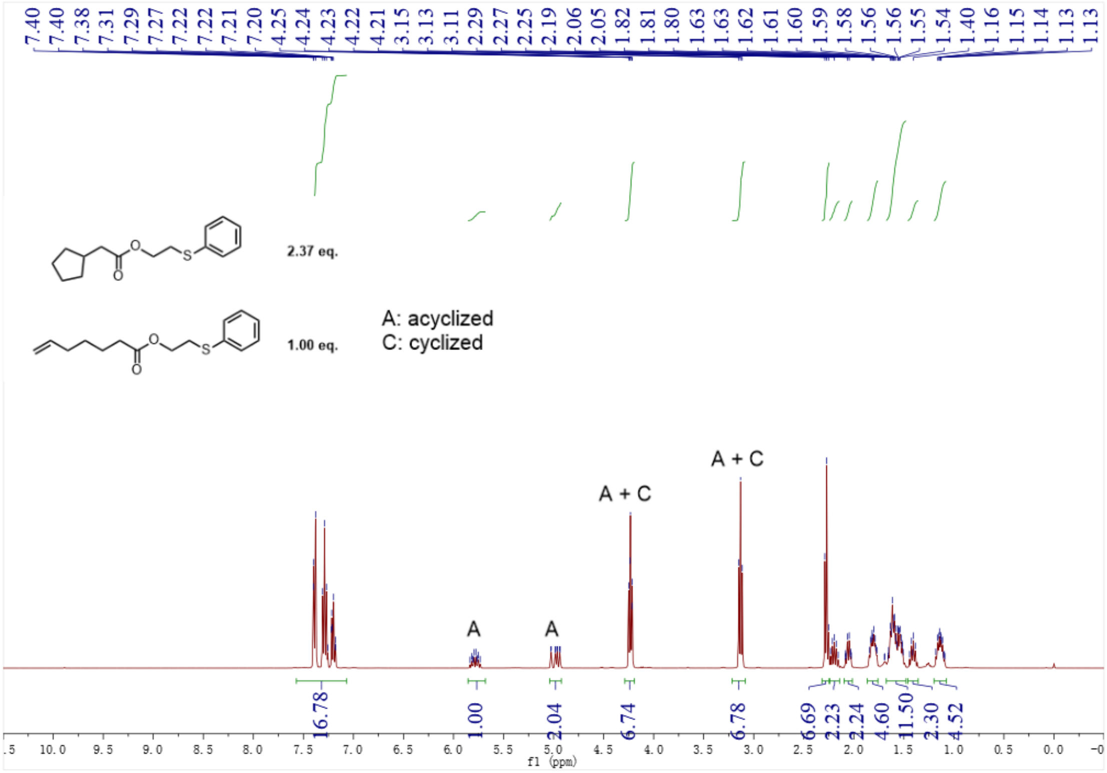

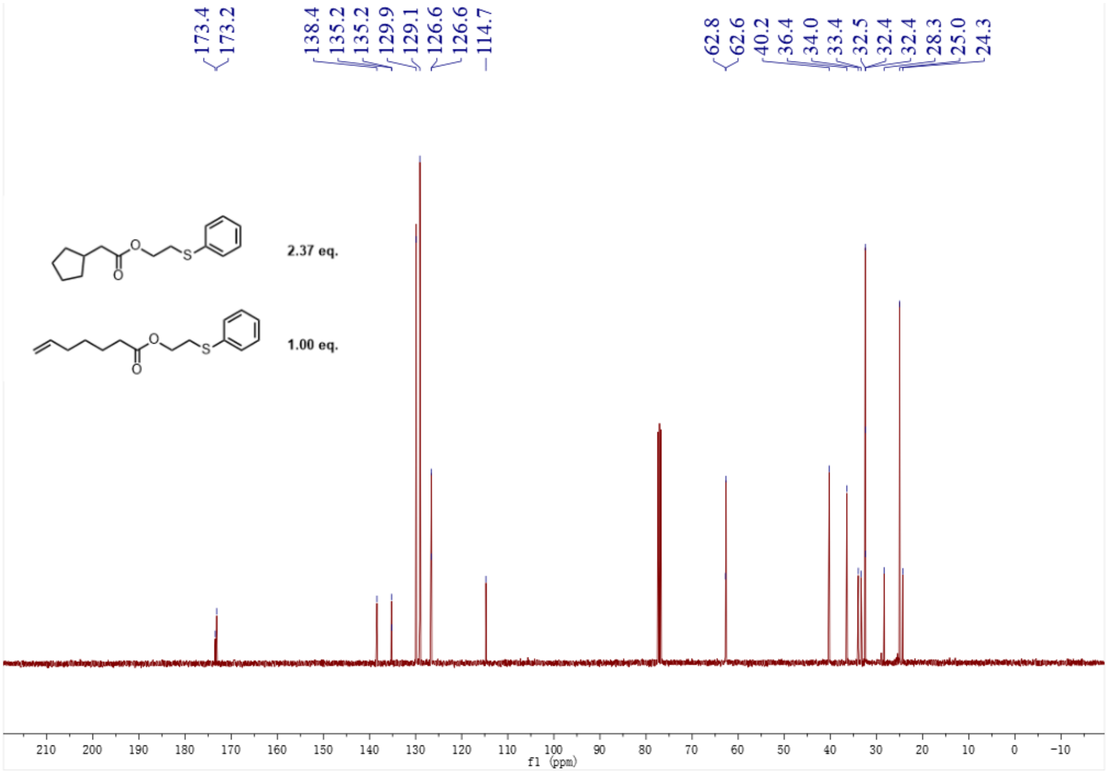

Radical ring-opening experiment

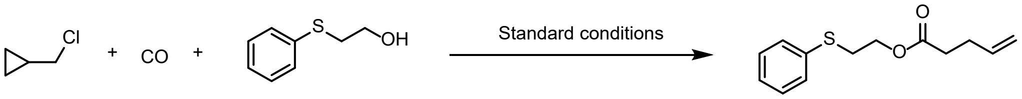

A 4 mL screw-cap vial was charged with Co(L_1) (x mol%), Zn (2x mol%), and an oven-dried stirring bar. The vial was closed with a Teflon septum and cap and connected to the atmosphere via a needle. The vial was purged with nitrogen in a nitrogen-filled glove box, followed by the addition of LiCl (40 mol%, 5.0 mg). After capping the vial tightly, it was taken out of the glove box. Then **1e** (0.6 mmol, 56 μL), **2a** (0.3 mmol, 40 μL), DBU (0.6 mmol, 90 μL), DMAc (1.0 mL) was added with a syringe under N_2 atmosphere, the vial was moved to an alloy plate and put into a Parr 4560 series autoclave (300 mL) under N_2 atmosphere. At room temperature, the autoclave flushed with N_2 three times and CO three times and charged with 5-6 bar CO. The autoclave was placed on a heating plate equipped with a magnetic stirrer. The reaction mixture was heated to 80 °C for 16 h. After the reaction was completed, the crude product was purified by column chromatography on silica gel to afford the corresponding compounds.

2-(Phenylthio)ethyl pent-4-enoate (51)

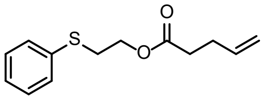

^{1}H NMR (400 MHz, CDCl$_3$) δ 7.27 – 7.20 (m, 2H), 7.14 (t, J = 7.7 Hz, 2H), 7.10 – 7.01 (m, 1H), 5.80 – 5.44 (m, 1H), 5.06 – 4.76 (m, 2H), 4.10 (t, J = 6.8 Hz, 2H), 2.98 (t, J = 6.8 Hz, 2H), 2.44 – 2.12 (m, 4H). **^{13}C NMR (100 MHz, CDCl$_3$)** δ 172.8, 136.6, 135.2, 129.9, 129.9, 129.1, 126.6, 115.6, 62.9, 33.4, 32.4, 28.8. **HRMS (ESI-TOF) m/z:** [M]$^+$: Calcd. for $C_{13}H_{17}O_2S^+$: 237.0944 Found: 237.0941.

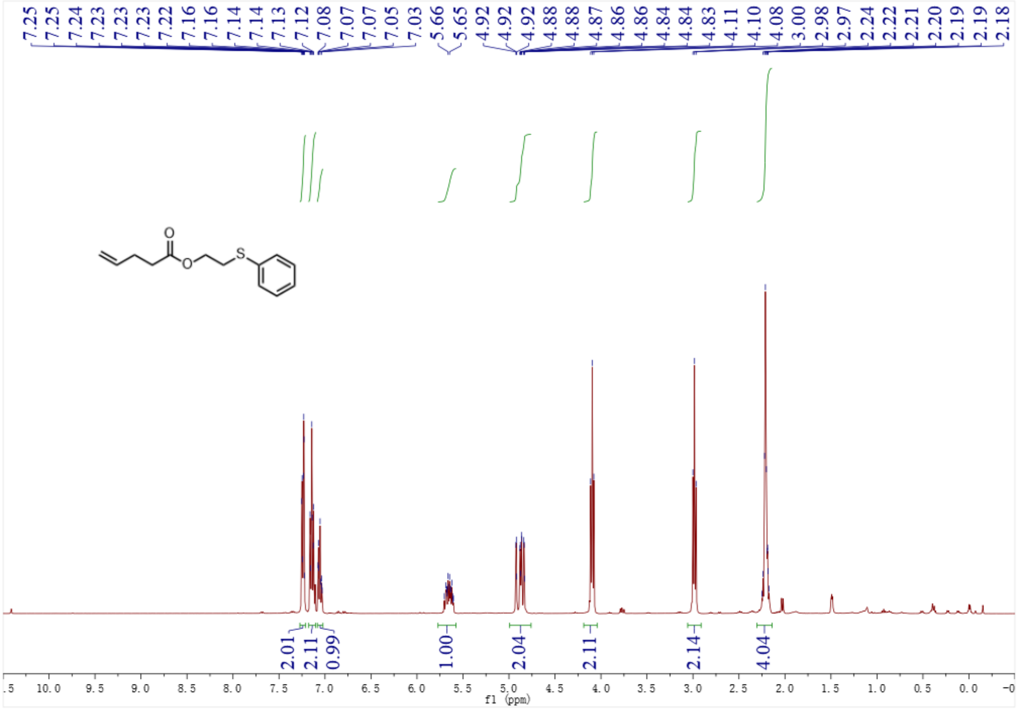

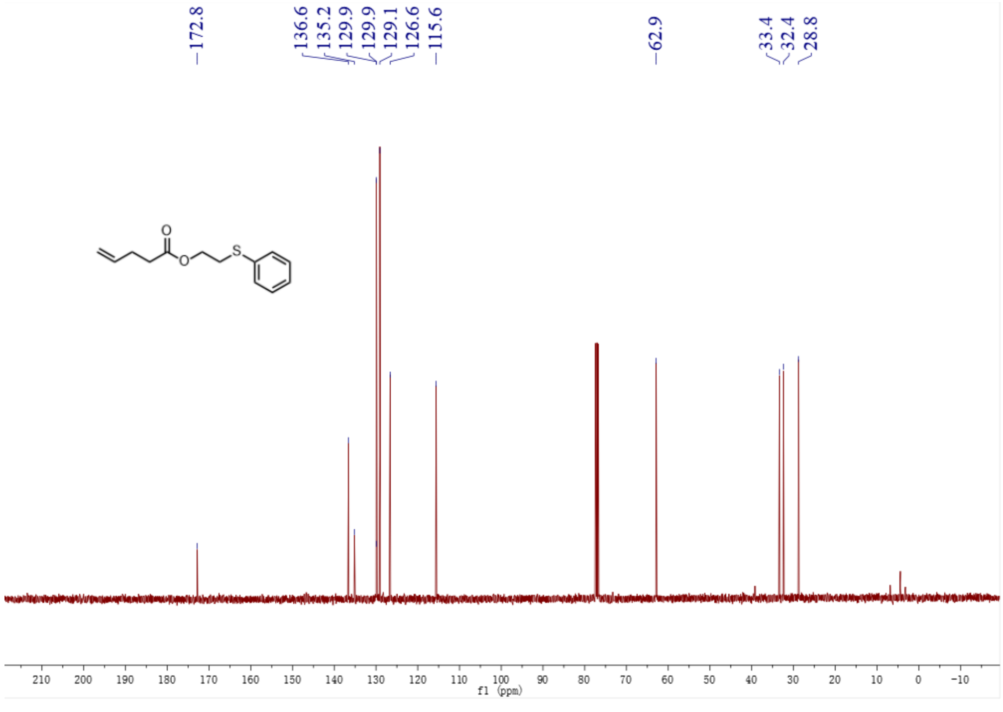

6.3 Hydroesterification of olefins

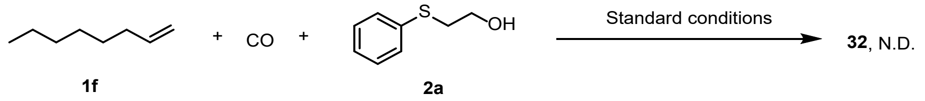

A 4 mL screw-cap vial was charged with Co(L_1) (10 mol%, 9.8 mg), Zn (20 mol%), and an oven-dried stirring bar. The vial was closed with a Teflon septum and cap and connected to the atmosphere via a needle. The vial was purged with nitrogen in a nitrogen-filled glove box, followed by the addition of LiCl (40 mol%, 5.0 mg). After capping the vial tightly, it was taken out of the glove box. Then **1f** (0.6 mmol, 93 μL), **2a** (0.3 mmol, 40 μL), DBU (0.6 mmol, 90 μL), DMAc (1.0 mL) was added with a syringe under N_2 atmosphere, the vial was moved to an alloy plate and put into a Parr 4560 series autoclave (300 mL) under N_2 atmosphere. At room temperature, the autoclave flushed with N_2 three times and CO three times and charged with 5-6 bar CO. The autoclave was placed on a heating plate equipped with a magnetic stirrer. The reaction mixture was heated to 80 °C for 16 h. After the reaction was completed, the reaction solution was filtered and analyzed by GC-MS.

6.4 Acyl chloride intermediate[2]

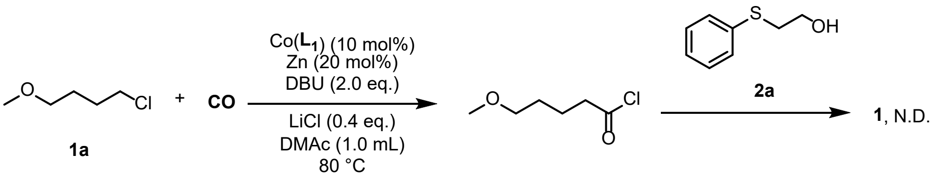

A 4 mL screw-cap vial was charged with Co(L_1) (10 mol%, 9.8 mg), Zn (20 mol%), and an oven-dried stirring bar. The vial was closed with a Teflon septum and cap and connected to the atmosphere via a needle. The vial was purged with nitrogen in a nitrogen-filled glove box, followed by the addition of LiCl (40 mol%, 5.0 mg). After capping the vial tightly, it was taken out of the glove box. Then **1a** (0.6 mmol, 80 μL), DBU (0.6 mmol, 90 μL), DMAc (1.0 mL) was added with a syringe under N_2 atmosphere, the vial was moved to an alloy plate and put into a Parr 4560 series autoclave (300 mL) under N_2 atmosphere. At room temperature, the autoclave flushed with N_2 three times and CO three times and charged with 5-6 bar CO. The autoclave was placed on a heating plate equipped with a magnetic stirrer. The reaction mixture was heated to 80 °C for 16 h. After the reaction is completed, the reaction vessel is cooled to room temperature. Then, **2a** was added to the reaction bottle and stirred for 1 hour. The reaction solution was filtered and then analyzed by GC-MS.

6.5 Synthesis of organic cobalt species

Synthesis of Alkylated $Co^{III}(L_1)$-^{n}Octyl Complex (Co-A)[3]

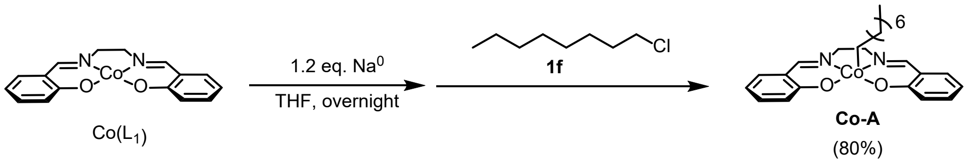

Step 1: In a nitrogen-filled glovebox, a 100 mL round-bottomed bottle was charged with a Co($\mathbf{L_1}$)

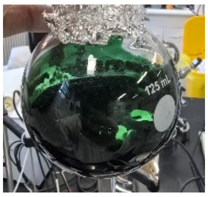

complex (1.35 g, 3.56 mmol 1.00 eq.), a piece of sodium metal (86 mg, 3.7 mmol, 1.1 eq.), and THF (40 mL, 88 mM). The round-bottomed flask was sealed with a rubber stopper and then stirred overnight in the glove box.

Step 2: The resulting dark green solution was filtered through a short plug of celite. Next, the filtrate was transferred into a new 100 mL round-bottomed bottle. The round-bottom flask was sealed with a rubber stopper, charged with a stirrer. It was taken out from the glove box and transferred to a -20 °C refrigerator for 2 hours.

Step 3: The bottle was then wrapped with aluminum foil, and pyridine (5.3 mmol, 1.5 eq.) and 1-chlorooctane (5.3 mmol, 1.5 eq.) were injected simultaneously into the stirring solution over a period of 2 seconds. The reaction was stirred in the dark, at room temperature, for another 20 minutes. The resulting dark solution was poured into 250 mL of water, the resulting suspension was allowed to stir for additional 10 min at room temperature under air, gradually turning bright red. The red suspension was filtered and was washed with both water (50 mL) and pentanes (50 mL) to yield the product **Co-A** as a clumpy red solid (1.10 g, 80%).

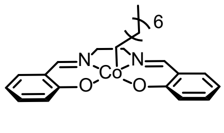

^{1}H NMR (700 MHz, DMSO-*d*$_6$) δ 8.01 (s, 2H), 7.13 (dd, *J* = 7.7, 2.1 Hz, 2H), 7.09 – 7.05 (m, 2H), 6.80 (d, *J* = 8.4 Hz, 2H), 6.38 (t, *J* = 7.0 Hz, 2H), 3.76 – 3.66 (m, 2H), 3.52 – 3.44 (m, 2H), 3.24 – 3.20 (m, 2H), 1.27 – 1.20 (m, 2H), 1.17 – 1.12 (m, 2H), 1.12 – 1.00 (m, 6H), 0.86 – 0.73 (m, 5H).

^{13}C NMR (175 MHz, DMSO-*d*$_6$) δ 166.5, 163.5, 133.8, 132.7, 122.3, 120.9, 113.0, 58.6, 40.0, 32.3, 31.6, 29.2, 29.0, 28.8, 22.5, 14.4.

HRMS (ESI-TOF) m/z: $[M]^+$: Calcd. for $C_{24}H_{31}CoN_2O_2^+$: 438.1718 Found: 438.1711.

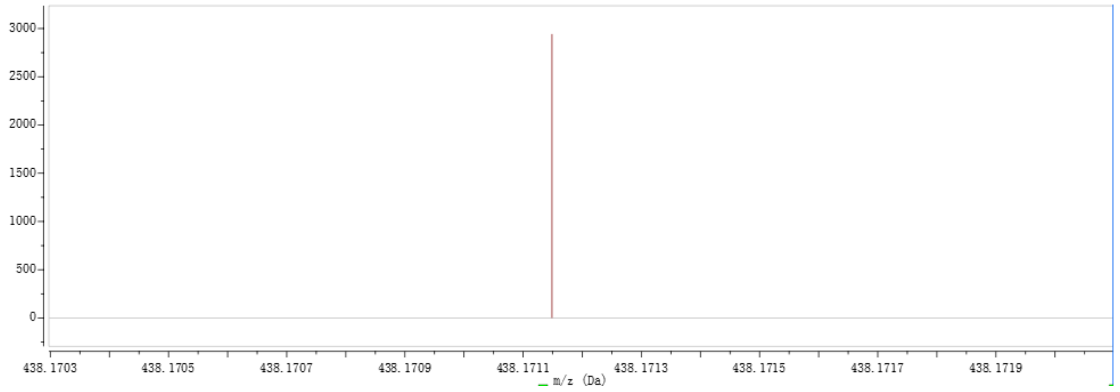

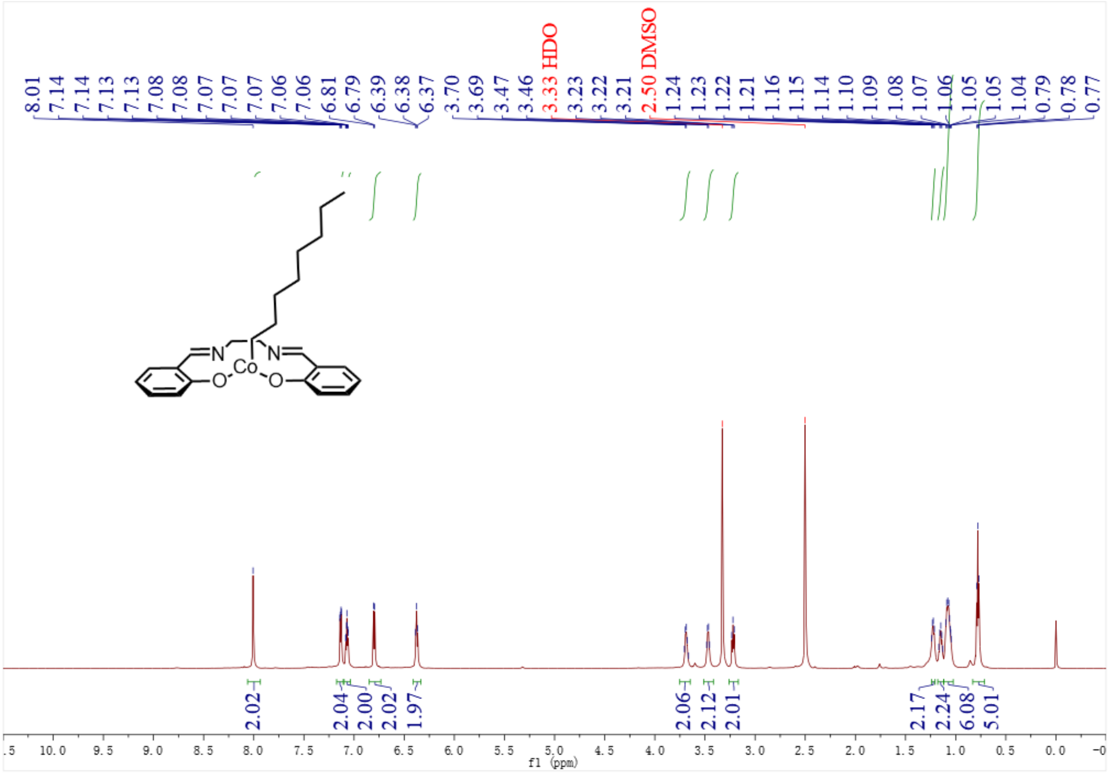

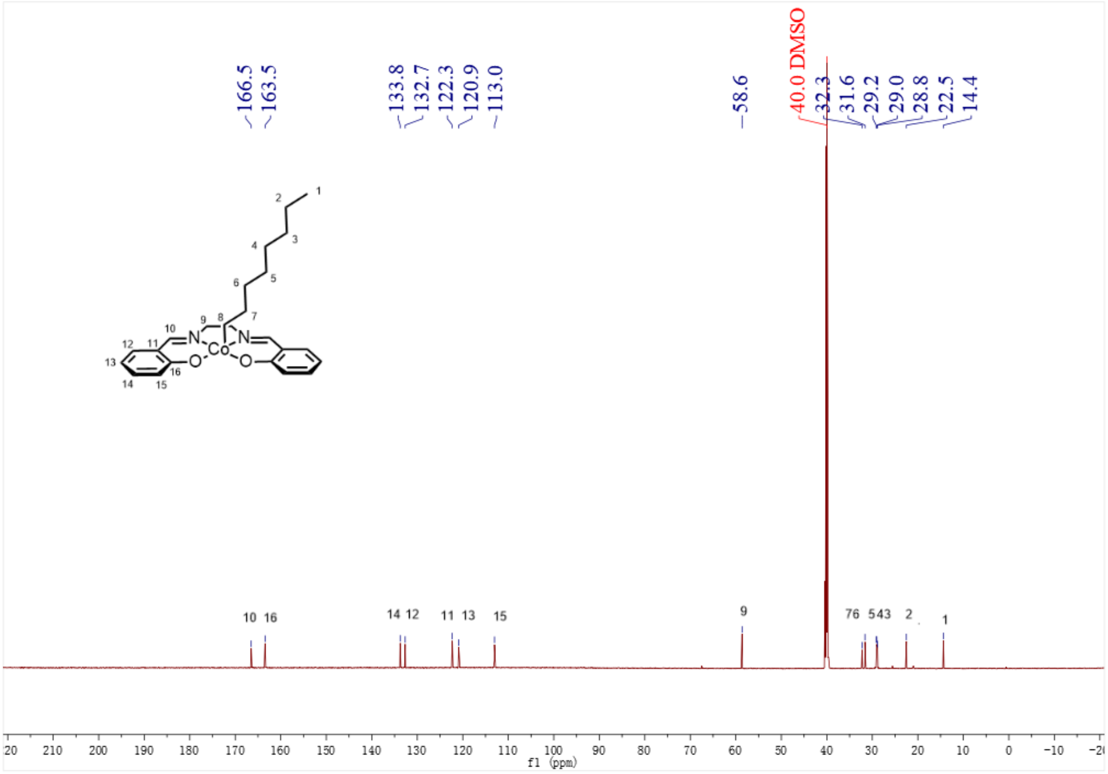

*The carbon is bonded directly to the quadrupole nucleus ^{59}Co (100% abundance, I = 7/2, Q = 0.42) and the T1 relaxation is too fast to allow detection of the coupling. In addition, residual line broadening means that in practice the signal for the coordinated carbon is undetectable.[4]

Synthesis of acylated Co^{III}(L$_1$)-^{n}Octanoyl Complex (Co-B)[5]

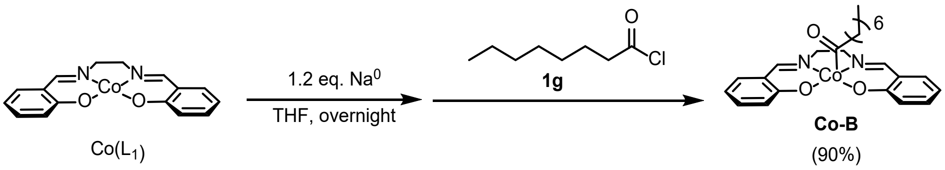

In a nitrogen-filled glovebox, a 100 mL round-bottomed bottle was charged with a Co(**L$_1$**) complex (1.35 g, 3.56 mmol 1.00 eq.), a piece of sodium metal (86 mg, 3.7 mmol, 1.1 eq.), and THF (40 mL, 88 mM). The round-bottomed flask was sealed with a rubber stopper and then stirred overnight in the glove box. The resulting dark green solution was filtered through a short plug of celite. Next, the filtrate was transferred into a new 100 mL round-bottomed bottle. The round-bottom flask was sealed with a rubber stopper, charged with a stirrer. It was taken out from the glove box and transferred to a -20 °C refrigerator for 2 hours. The bottle was then wrapped with aluminum foil, ^{n}octanoyl chloride (5.3 mmol, 1.5 eq.) were injected into the stirring solution dropwise. The reaction was stirred in the dark, at room temperature, for 20 minutes. The reaction mixture was filtered

through a pad of silica gel. The filtrate was concentrated under vacuum to give the crude desired product as a dark brown solid. Then 20 mL of n-hexane was added, and the suspension was pulped for 2 hours. The suspension was filtered to obtain the pure product **Co-B** (90%).

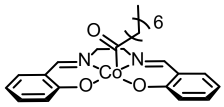

^{1}H NMR (700 MHz, DMSO-d_6) δ 8.00 (s, 2H), 7.17 (d, J = 7.7 Hz, 2H), 7.12 (d, J = 8.4 Hz, 2H), 6.87 (d, J = 8.4 Hz, 2H), 6.43 (s, 2H), 3.61 (s, 4H), 3.36 (s, 4H), 1.36 – 1.25 (m, 2H), 1.17 – 1.07 (m, 2H), 0.99 (s, 4H), 0.95 – 0.87 (m, 2H), 0.76 (t, J = 7.0 Hz, 3H).

^{13}C NMR (175 MHz, DMSO-d_6) δ 166.0, 163.3, 133.8, 133.1, 122.0, 121.1, 113.7, 58.1, 45.9, 40.1, 31.5, 28.8, 28.6, 26.0, 22.4, 14.4.

HRMS (ESI-TOF) m/z: $[M+H]^+$: Calcd. for $C_{24}H_{30}CoN_2O_3^+$: 453.1583 Found: 453.1596.

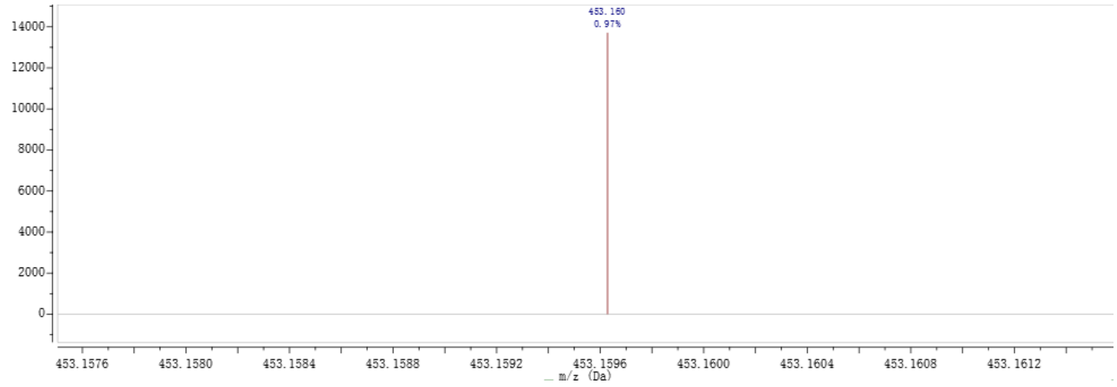

Synthesis of acylated $Co^{III}(L_1)$-^{n}Octanoyl Complex (Co-C)

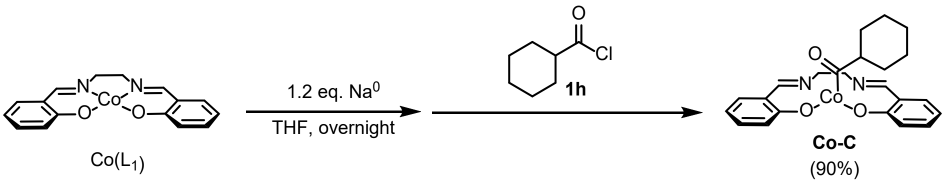

In a nitrogen-filled glovebox, a 100 mL round-bottomed bottle was charged with a Co(**L$_1$**) complex (1.35 g, 3.56 mmol 1.00 eq.), a piece of sodium metal (86 mg, 3.7 mmol, 1.1 eq.), and THF (40 mL, 88 mM). The round-bottomed flask was sealed with a rubber stopper and then stirred overnight in the glove box. The resulting dark green solution was filtered through a short plug of celite. Next, the filtrate was transferred into a new 100 mL round-bottomed bottle. The round-bottom flask was sealed with a rubber stopper, charged with a stirrer. It was taken out from the glove box and transferred to a -20 °C refrigerator for 2 hours. The bottle was then wrapped with aluminum foil, cyclohexyl chloroacetate (5.3 mmol, 1.5 eq.) were injected into the stirring solution dropwise. The

reaction was stirred in the dark, at room temperature, for 20 minutes. The reaction mixture was filtered through a pad of silica gel. The filtrate was concentrated under vacuum to give the crude desired product as a dark brown solid. Then 20 mL of n-hexane was added, and the suspension was pulped for 2 hours. The suspension was filtered to obtain the pure product **Co-C** (90%).

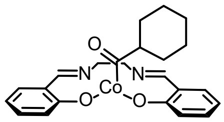

^{1}H NMR (700 MHz, DMSO-*d*$_6$) δ 7.98 (s, 2H), 7.28 – 7.08 (m, 4H), 6.89 (d, *J* = 8.4 Hz, 2H), 6.42 (t, *J* = 7.0 Hz, 2H), 4.27 (t, *J* = 10.5 Hz, 1H), 3.62 (s, 4H), 1.36 (d, *J* = 9.8 Hz, 4H), 1.18 – 0.91 (m, 6H).

^{13}C NMR (175 MHz, DMSO-*d*$_6$) δ 166.6, 163.3, 133.7, 133.1, 121.5, 120.9, 113.8, 58.1, 56.9, 30.1, 26.1, 25.6.

HRMS (ESI-TOF) m/z: $[M]^+$: Calcd. for $C_{23}H_{26}CoN_2O_3^+$: 437.1270 Found: 437.1275.

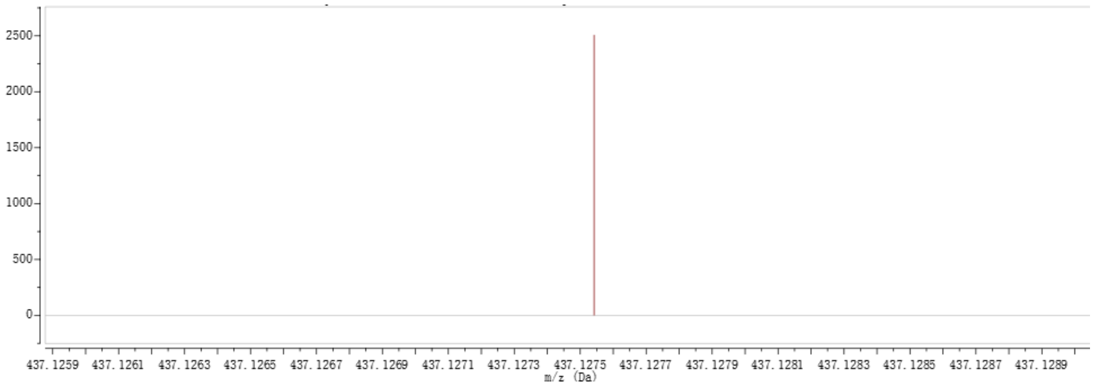

6.6 Conversion of organic cobalt species

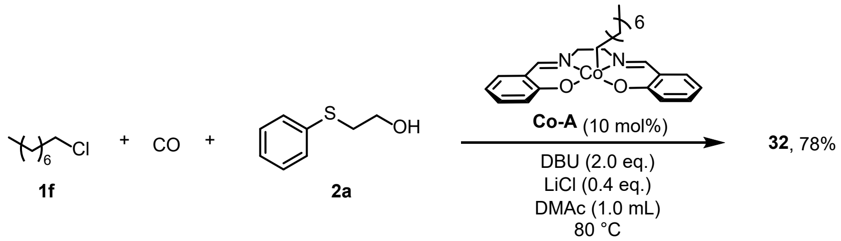

A 4 mL screw-cap vial was charged with Co-A (10 mol%, 13 mg) and an oven-dried stirring bar. The vial was closed with a Teflon septum and cap and connected to the atmosphere via a needle. The vial was purged with nitrogen in a nitrogen-filled glove box, followed by the addition of LiCl (40 mol%, 5.0 mg). After capping the vial tightly, it was taken out of the glove box. Then **1f** (0.6 mmol, 102 μL), **2a** (0.3 mmol, 40 μL), DBU (0.6 mmol, 90 μL), DMAc (1.0 mL) was added with a

syringe under N_2 atmosphere, the vial was moved to an alloy plate and put into a Parr 4560 series autoclave (300 mL) under N_2 atmosphere. At room temperature, the autoclave flushed with N_2 three times and CO three times and charged with 5-6 bar CO. The autoclave was placed on a heating plate equipped with a magnetic stirrer. The reaction mixture was heated to 80 °C for 16 h. After the reaction was completed, the mixture was diluted with sat. LiCl (10 mL) and extracted with EtOAc (3 x 10 mL), then the organic phase was washed with sat. brine (20 mL), dried over Na_2SO_4, filtered, and concentrated in vacuo. The crude product was purified by column chromatography to furnish the desired product.

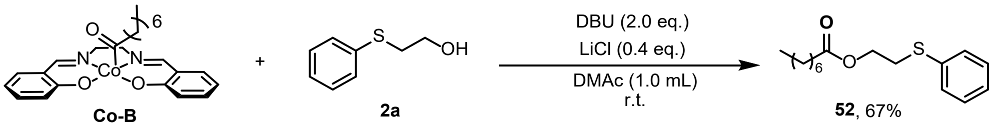

A 4 mL screw-cap vial was charged with Co-B (0.3 mmol, 135.6 mg) and an oven-dried stirring bar. The vial was closed with a Teflon septum and cap and connected to the atmosphere via a needle. The vial was purged with nitrogen in a nitrogen-filled glove box, followed by the addition of LiCl (40 mol%, 5.0 mg). After capping the vial tightly, it was taken out of the glove box. Then **2a** (0.3 mmol, 40 μL), DBU (0.6 mmol, 90 μL), DMAc (1.0 mL) was added with a syringe under N_2 atmosphere, the vial was stirred at room temperature for 16 h. After the reaction was completed, the mixture was diluted with sat. LiCl (10 mL) and extracted with EtOAc (3 x 10 mL), then the organic phase was washed with sat. brine (20 mL), dried over Na_2SO_4, filtered, and concentrated in vacuo. The crude product was purified by column chromatography to furnish the desired product.

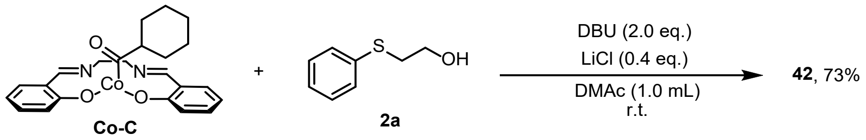

A 4 mL screw-cap vial was charged with Co-B (0.3 mmol, 135.6 mg) and an oven-dried stirring bar. The vial was closed with a Teflon septum and cap and connected to the atmosphere via a needle. The vial was purged with nitrogen in a nitrogen-filled glove box, followed by the addition of LiBr (40 mol%, 11.0 mg). After capping the vial tightly, it was taken out of the glove box. Then **2a** (0.3 mmol, 40 μL), DBU (0.6 mmol, 90 μL), DMAc (1.0 mL) was added with a syringe under N_2 atmosphere, the vial was stirred at room temperature for 16 h. After the reaction was completed, the mixture was diluted with sat. LiCl (10 mL) and extracted with EtOAc (3 x 10 mL), then the organic

phase was washed with sat. brine (20 mL), dried over Na_2SO_4, filtered, and concentrated in vacuo. The crude product was purified by column chromatography to furnish the desired product.

6.7 Nucleophilic recognition of radical pathway

1.0 eq. BHT as the substrate

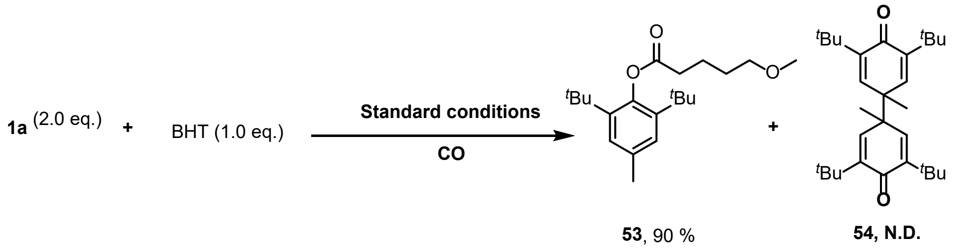

A 4 mL screw-cap vial was charged with Co(**L1**) (10 mol%, 9.8 mg), BHT (0.3 mmol, 66 mg), Zn (20 mol%, 4.0 mg) and an oven-dried stirring bar. The vial was closed with a Teflon septum and cap and connected to the atmosphere via a needle. The vial was purged with nitrogen in a nitrogen-filled glove box, followed by the addition of LiCl (40 mol%, 5.0 mg). After capping the vial tightly, it was taken out of the glove box. Then **1a** (0.6 mmol, 80 μL), DBU (0.6 mmol, 90 μL), DMAc (1.0 mL) was added with a syringe under N_2 atmosphere, the vial was moved to an alloy plate and put into a Parr 4560 series autoclave (300 mL) under N_2 atmosphere. At room temperature, the autoclave flushed with N_2 three times and CO three times and charged with 5-6 bar CO. The autoclave was placed on a heating plate equipped with a magnetic stirrer. The reaction mixture was heated to 80 °C for 16 h. After the reaction was completed, the mixture was diluted with sat. LiCl (10 mL) and extracted with EtOAc (3 x 10 mL), then the organic phase was washed with sat. brine (20 mL), dried over Na_2SO_4, filtered, and concentrated in vacuo. The crude product was purified by column chromatography to furnish the desired product.

2.0 eq. BHT as the substrate

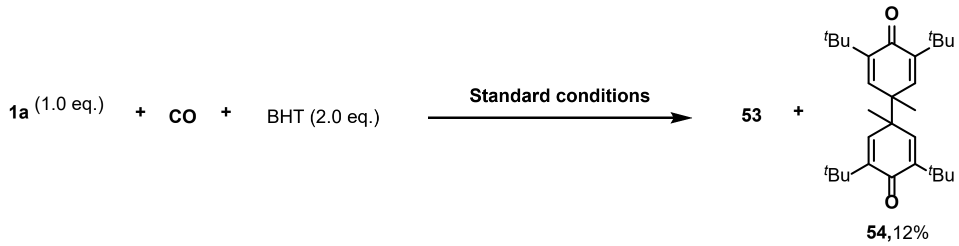

A 4 mL screw-cap vial was charged with Co(**L1**) (10 mol%, 9.8 mg), BHT (0.6 mmol, 132 mg), Zn (20 mol%, 4.0 mg) and an oven-dried stirring bar. The vial was closed with a Teflon septum and cap

and connected to the atmosphere via a needle. The vial was purged with nitrogen in a nitrogen-filled glove box, followed by the addition of LiCl (40 mol%, 5.0 mg). After capping the vial tightly, it was taken out of the glove box. Then **1a** (0.3 mmol, 40 μL), DBU (0.6 mmol, 90 μL), DMAc (1.0 mL) was added with a syringe under N_2 atmosphere, the vial was moved to an alloy plate and put into a Parr 4560 series autoclave (300 mL) under N_2 atmosphere. At room temperature, the autoclave flushed with N_2 three times and CO three times and charged with 5-6 bar CO. The autoclave was placed on a heating plate equipped with a magnetic stirrer. The reaction mixture was heated to 80 °C for 16 h. After the reaction was completed, the mixture was diluted with sat. LiCl (10 mL) and extracted with EtOAc (3 x 10 mL), then the organic phase was washed with sat. brine (20 mL), dried over Na_2SO_4, filtered, and concentrated in vacuo. The crude product was purified by column chromatography to furnish the desired product.

3,3',5,5'-tetra-*tert*-butyl-1,1'-dimethyl-[1,1'-bi(cyclohexane)]-2,2',5,5'-tetraene-4,4'-dione (54)[6]

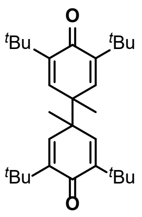

^{1}H NMR (400 MHz, $CDCl_3$) δ 6.56 (s, 4H), 1.42 (s, 6H), 1.22 (s, 36H). **^{13}C NMR (100 MHz, $CDCl_3$)** δ 186.1, 145.4, 143.2, 67.4, 34.5, 29.4, 28.0. M. P.: 78 - 81 °C. **HRMS (ESI-TOF) m/z:** $[M+H]^+$: Calcd. for $C_{30}H_{47}O_2^+$: 439.3571 Found: 439.3568.

<u>Stoichiometric Co-A as the substrate</u>

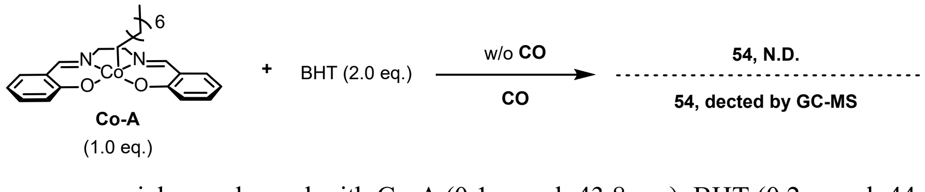

A 4 mL screw-cap vial was charged with Co-A (0.1 mmol, 43.8 mg), BHT (0.2 mmol, 44 mg) and an oven-dried stirring bar. The vial was closed with a Teflon septum and cap and connected to the atmosphere via a needle. The vial was purged with nitrogen in a nitrogen-filled glove box, followed by the addition of LiCl (40 mol%, 5.0 mg). After capping the vial tightly, it was taken out of the glove box. Then DBU (0.6 mmol, 90 μL), DMAc (1.0 mL) was added with a syringe under N_2 atmosphere, the vial was moved to an alloy plate and put into a Parr 4560 series autoclave (One

reaction was performed directly at 80 °C without replacing the atmosphere with CO) under N_2 atmosphere. At room temperature, the autoclave flushed with N_2 three times and CO three times and charged with 5-6 bar CO. The autoclave was placed on a heating plate equipped with a magnetic stirrer. The reaction mixture was heated to 80 °C for 16 h. After the reaction was completed, the mixture was diluted with sat. LiCl (10 mL) and extracted with EtOAc (3 x 10 mL), then the organic phase was washed with sat. brine (20 mL), dried over Na_2SO_4, filtered, and concentrated in vacuo. Upon reaction completion, the mixture was analyzed by TLC and GC-MS.

Stoichiometric Co-B as the substrate

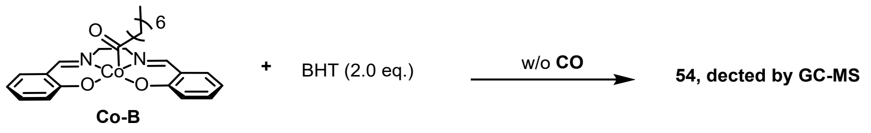

A 4 mL screw-cap vial was charged with Co-B (0.1 mmol, 45.2 mg), BHT (0.2 mmol, 44 mg) and an oven-dried stirring bar. The vial was closed with a Teflon septum and cap and connected to the atmosphere via a needle. The vial was purged with nitrogen in a nitrogen-filled glove box, followed by the addition of LiCl (40 mol%, 5.0 mg). After capping the vial tightly, it was taken out of the glove box. Then DBU (0.6 mmol, 90 μL), DMAc (1.0 mL) was added with a syringe under N_2 atmosphere, the vial was moved to an alloy plate and put into a Parr 4560 series autoclave under N_2 atmosphere. At room temperature, the autoclave flushed with N_2 three times and CO three times and charged with 5-6 bar CO. The autoclave was placed on a heating plate equipped with a magnetic stirrer. The reaction mixture was heated to 80 °C for 16 h. After the reaction was completed, the mixture was diluted with sat. LiCl (10 mL) and extracted with EtOAc (3 x 10 mL), then the organic phase was washed with sat. brine (20 mL), dried over Na_2SO_4, filtered, and concentrated in vacuo. Upon reaction completion, the mixture was analyzed by TLC and GC-MS.

Detection of alkoxyl radicals

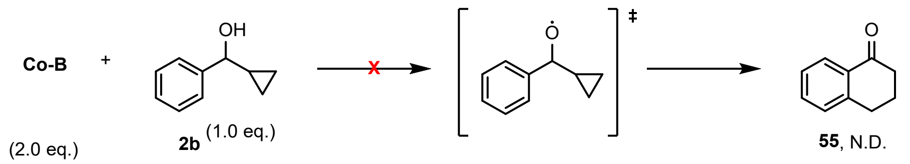

A 4 mL screw-cap vial was charged with **Co-B** (0.2 mmol, 90.4 mg) and an oven-dried stirring bar. The vial was closed with a Teflon septum and cap and connected to the atmosphere via a needle. The vial was purged with nitrogen in a nitrogen-filled glove box, followed by the addition of LiCl

(40 mol%, 5.0 mg). After capping the vial tightly, it was taken out of the glove box. Then **2b** (0.1 mmol, 15 mg), DMAc (1.0 mL) was added with a syringe under N_2 atmosphere. Stirred at 80 °C for 16 h, the mixture was analyzed by GC-MS.

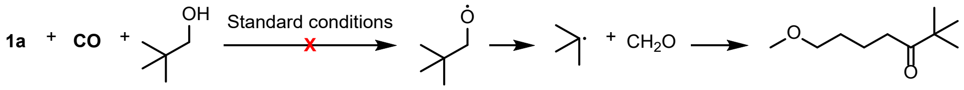

2c **56**, N.D.

A 4 mL screw-cap vial was charged with Co(**L₁**) (10 mol%, 9.8 mg), Zn (20 mol%, 4.0 mg), **2c** (0.3 mmol, 26.4 mg) and an oven-dried stirring bar. The vial was closed with a Teflon septum and cap and connected to the atmosphere via a needle. The vial was purged with nitrogen in a nitrogen-filled glove box, followed by the addition of LiCl (40 mol%, 5.0 mg). After capping the vial tightly, it was taken out of the glove box. Then **1a** (0.6 mmol, 80 μL), DBU (0.6 mmol, 90 μL), DMAc (1.0 mL) was added with a syringe under N_2 atmosphere, the vial was moved to an alloy plate and put into a Parr 4560 series autoclave (300 mL) under N_2 atmosphere. At room temperature, the autoclave flushed with N_2 three times and CO three times and charged with 5-6 bar CO. The autoclave was placed on a heating plate equipped with a magnetic stirrer. The reaction mixture was heated to 80 °C for 16 h. After the reaction was completed, the mixture was analyzed by GC-MS.

6.8 EPR and spin-trapping EPR[7]

<u>**Co-B + BHT:**</u> 10 mg **Co-B** was added in a 4 mL screw-cap vial charged with an oven-dried stirring bar. Then, 1 mL degassed DMAc was added. Next, 22 mg BHT was added and stirred under air at 25 °C for 1 minute. The mixture was put into a simple melting point tube for EPR test. EPR spectra was recorded at room temperature and operated at 9.326475 GHz. Typical spectrometer parameters were shown as follows, sweep width: 150 G; center field set: 3322 G; time constant: 163.84 ms; sweep time: 30.72 s; modulation amplitude: 1.0 G; modulation frequency: 9.326475 GHz; receiver gain: 1.00237×10^4; microwave power: 5.0128 mW.

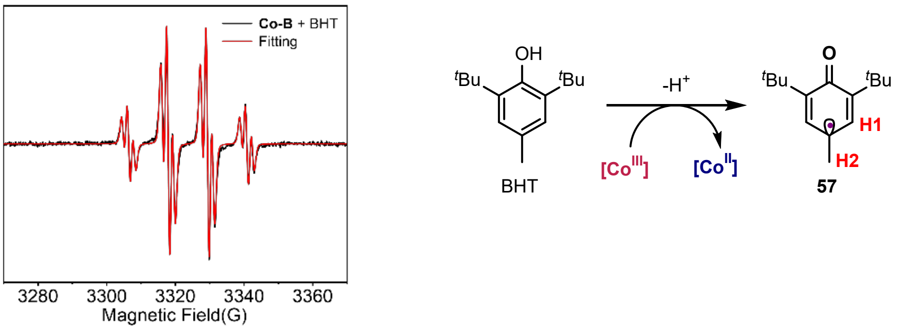

Figure S3: EPR experiments at room temperature of **<u>Co-B + BHT.</u>**

57: Fitting results: *g* = 2.0059, AH1 = 1.6 G, AH2 = 11.5 G

<u>Co-B + ^{n}Bu$_2$NH + DMPO:</u> 10 mg **Co-B** was added in a 4 mL screw-cap vial charged with an oven-dried stirring bar. Then, 1 mL degassed DMAc was added. Next, 10μL ^{n}Bu$_2$NH was added stirred under air at 25 °C for 1 minute. Then 40 μL spin-trapping reagent 5,5-dimethyl-1-pyrroline *N*-oxide (DMPO) was added and reacted for two minutes. The mixture was put into a simple melting point tube for EPR test. EPR spectra was recorded at room temperature and operated at 9.320346 GHz. Typical spectrometer parameters were shown as follows, sweep width: 150 G; center field set: 3320 G; time constant: 163.84 ms; sweep time: 30.72 s; modulation amplitude: 1.0 G; modulation frequency: 9.320346 GHz; receiver gain: 1.002374×10^4; microwave power: 5.012 mW.

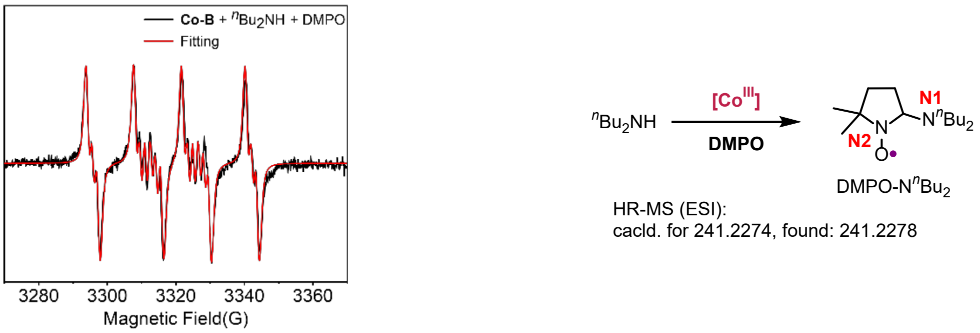

Figure S4: EPR experiments at room temperature of **<u>Co-B + ^{n}Bu$_2$NH + DMPO.</u>**

DMPO-N^{n}Bu$_2$: Fitting results: *g* = 2.0058, AN1 = 1.5 G, AH = 18.6 G, AN2 = 14.0 G

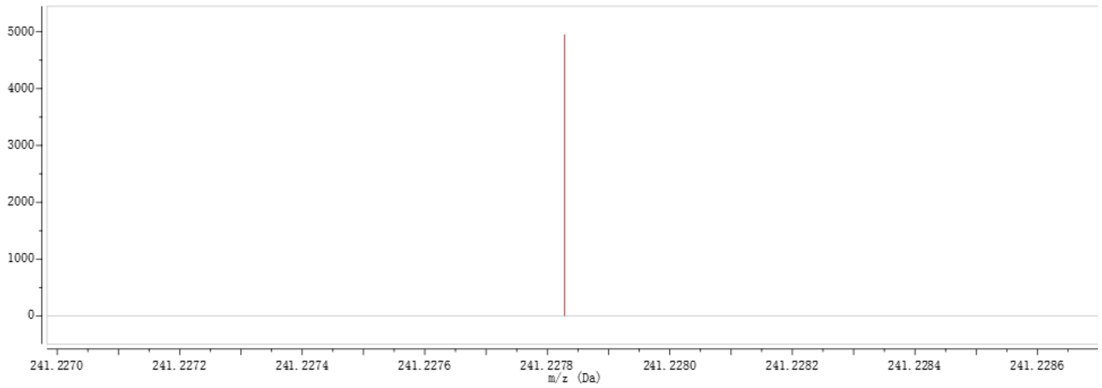

6.9 Investigation of secondary alkyl bromides and alkyl iodides intermediates[8]

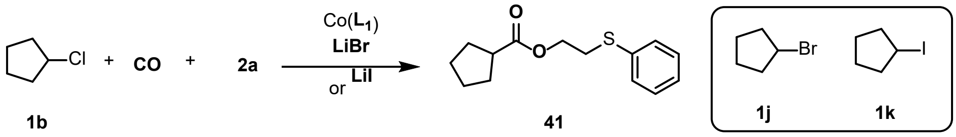

A 4 mL screw-cap vial was charged with Co(**L**$_1$) (10 mol%, 9.8 mg), Zn (20 mol%, 4.0 mg) and an oven-dried stirring bar. The vial was closed with a Teflon septum and cap and connected to the atmosphere via a needle. The vial was purged with nitrogen in a nitrogen-filled glove box, followed by the addition of LiBr (1.0 eq., 26.1 mg) or LiI (1.0 eq., 40.2 mg). After capping the vial tightly, it was taken out of the glove box. Then **1b** (0.6 mmol, 63 μL), **2a** (0.3 mmol, 40 μL), DBU (0.6 mmol, 90 μL), DMAc (1.0 mL) was added with a syringe under N_2 atmosphere, the vial was moved to an alloy plate and put into a Parr 4560 series autoclave (300 mL) under N_2 atmosphere. At room temperature, the autoclave flushed with N_2 three times and CO three times and charged with 5-6 bar CO. The autoclave was placed on a heating plate equipped with a magnetic stirrer. The reaction mixture was heated to 80 °C for set time. After the reaction was completed, immediately placed the autoclave in an ice-water bath to stop the reaction. Added dodecane as an internal standard and use GC to detect the yield.

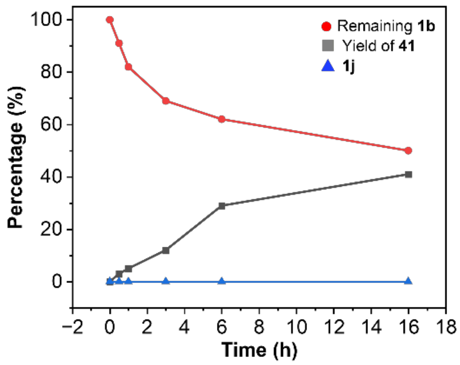

Figure S5: The proportion of each component of LiBr as an additive

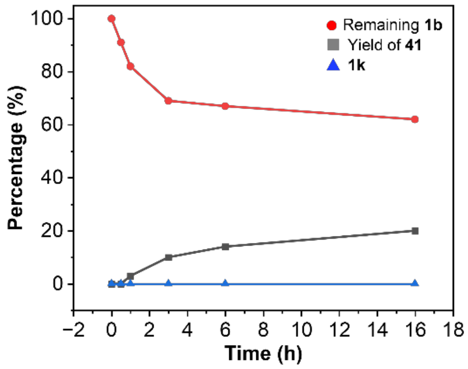

Figure S6: The proportion of each component of LiI as an additive

6.10 Stability Studies of Co-B and Co-C at Elevated Temperatures with Additive[3]

*Reactions were setup in a nitrogen-filled glovebox in the dark. **Co-B** and **Co-C** are moderately light sensitive.

Step 1: A 20 mL vial containing a PTFE stir bar was charged with the following: **Co-B** (32.5 mg, 0.072 mmol, 1.0 eq.), 1,3,5-trimethoxybenzene internal standard (12 mg), and a solvent consisting of $CDCl_3$ (1.20 mL) and *N,N*-dimethylacetamide (2.40 mL). The resulting solution was allowed to stir for 5 minutes before use. A series of 4 mL vials equipped with PTFE stir bars were charged with either LiCl (3 mg, 0.06 mmol, 5 eq.) or LiBr (11 mg, 0.060 mmol, 5.0 eq.). Next, 0.6 mL of a stock solution of **Co-B** was dispensed into each 4 mL vial containing a respective additive. The resulting solutions were allowed to stir for 5 min before being transferred into an NMR tube. NMR tubes were capped, with caps being taped with a vinyl electrical tape and wrapped in aluminum foil during transportation to avoid exposure to ambient light.

Step 2: NMR tubes were placed into a preheated (80 ˚C) thermostatic metal bath and were heated for a specified time followed by a prompt analysis *via* a quantitative ^{1}H NMR spectroscopy. The amount of remaining **Co-B** was calculated with respect to the 1,3,5-trimethoxybenzene internal standard.

^{1}H NMR of internal standard 1,3,5-trimethoxybenzene

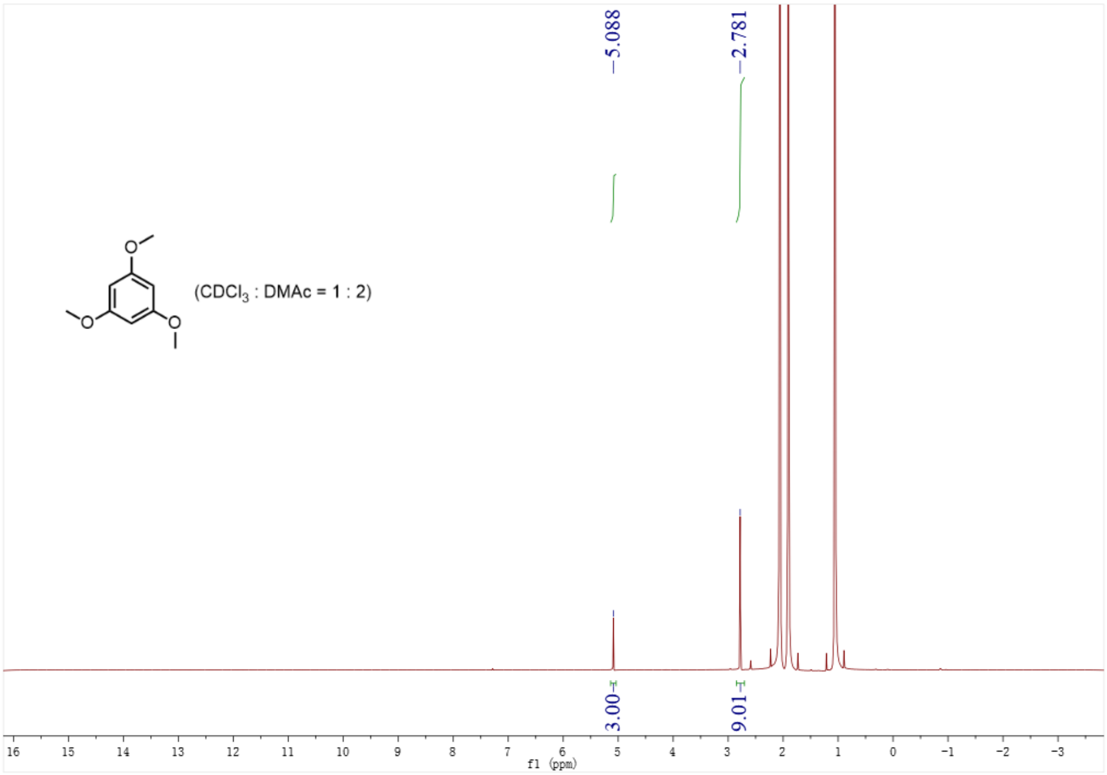

^{1}H NMR of **Co-B** with LiBr for example

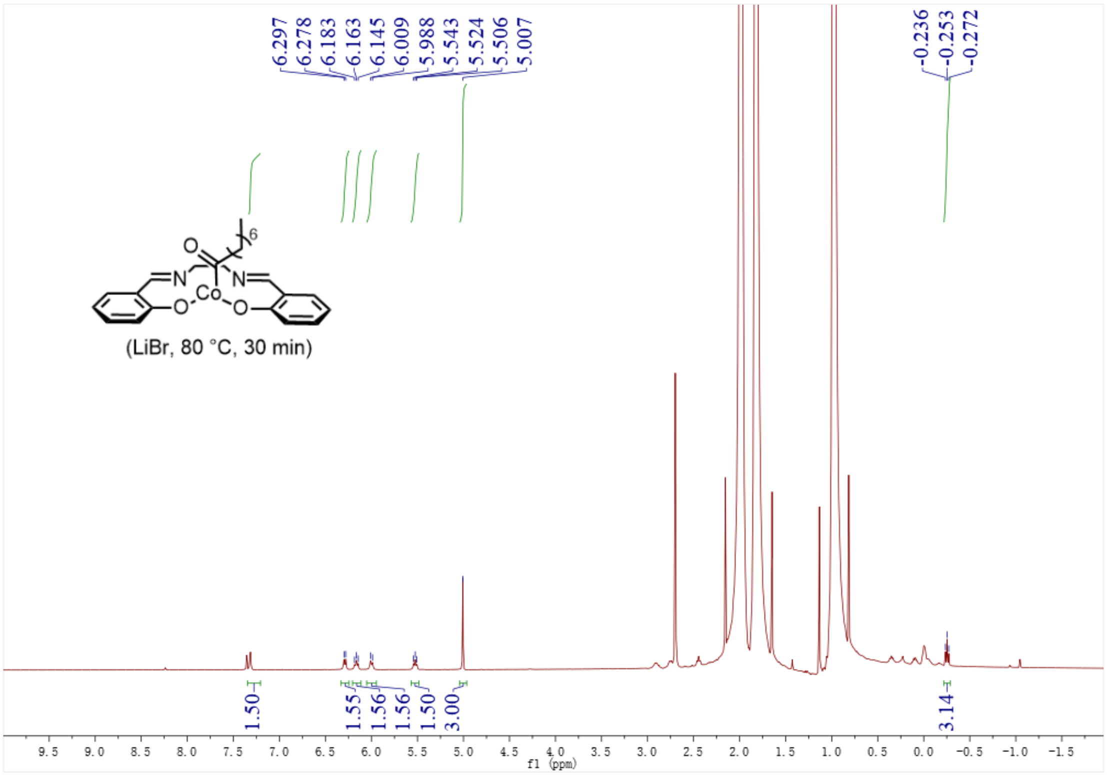

Table 12. Monitored conversion of **Co-B** at 80 ˚C in the presence of electrolytes.

Entry	Additive	Duration (min)	Temperature (°C)	Remaining **Co-B** (%)
1	none	30	80	10
2	LiCl	30	80	65
3	LiBr	30	80	75
4	none	60	80	0
5	LiCl	60	80	36
6	LiBr	60	80	45

Table 13. Monitored conversion of **Co-B** at 80 ˚C in the presence of electrolytes.

Entry	Additive	Duration (min)	Temperature (°C)	Remaining **Co-C** (%)
1	none	30	80	60
2	LiCl	30	80	72
3	LiBr	30	80	95
4	LiI	30	80	97
5	$LiClO_4$	30	80	95
6	NaI	30	80	95

7. Characterization data of products

2-(Phenylthio)ethyl 5-methoxypentanoate (1)

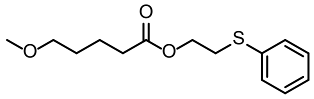

The title compound was prepared following the general procedure, purification by column chromatography on silica gel (petroleum ether/EtOAc = 30:1) yielded (71 mg, 89%) as a colorless oil. **^{1}H NMR (700 MHz, $CDCl_3$)** δ 7.42 – 7.36 (m, 2H), 7.31 – 7.26 (m, 2H), 7.23 – 7.18 (m, 1H), 4.23 (t, J = 7.0 Hz, 2H), 3.36 (t, J = 6.3 Hz, 2H), 3.31 (s, 3H), 3.13 (t, J = 7.0 Hz, 2H), 2.30 (t, J = 7.7 Hz, 2H), 1.69 – 1.63 (m, 2H), 1.62 – 1.55 (m, 2H). ^{13}C NMR (175 MHz, $CDCl_3$) δ 173.3, 135.2, 129.8, 129.0, 126.5, 72.2, 62.8, 58.5, 33.8, 32.4, 29.0, 21.6. **HRMS (ESI-TOF) m/z:** $[M+H]^+$: Calcd. for $C_{14}H_{21}O_3S^+$: 269.1206. Found: 269.1205.

3-Methoxypropyl 5-methoxypentanoate (2)

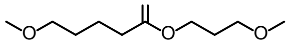

The title compound was prepared following the general procedure, purification by column chromatography on silica gel (petroleum

ether/EtOAc = 30:1) yielded (53 mg, 88%) as a colorless oil. **^{1}H NMR (400 MHz, CDCl$_3$)** δ 4.16 (t, *J* = 6.4 Hz, 2H), 3.44 (t, *J* = 6.4 Hz, 2H), 3.38 (t, *J* = 6.4 Hz, 2H), 3.33 (s, 3H), 3.33 (s, 3H), 2.34 (t, *J* = 7.2 Hz, 2H), 1.97 – 1.85 (m, 2H), 1.75 – 1.66 (m, 2H), 1.65 – 1.56 (m, 2H). **^{13}C NMR (100 MHz, CDCl$_3$)** δ 173.5, 72.2, 69.2, 61.5, 58.7, 58.6, 34.0, 29.0, 29.0, 21.7. **HRMS (ESI-TOF) m/z:** [M+H]$^+$: Calcd. for $C_{10}H_{21}O_4^+$: 205.1435. Found: 205.1433.

Butyl 5-methoxypentanoate (3)

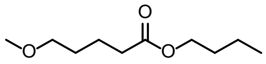

The title compound was prepared following the general procedure, purification by column chromatography on silica gel (petroleum ether/EtOAc = 50:1) yielded (49 mg, 82%) as a colorless oil. **^{1}H NMR (400 MHz, CDCl$_3$)** δ 4.06 (t, *J* = 6.8 Hz, 2H), 3.38 (t, *J* = 6.4 Hz, 2H), 3.33 (s, 3H), 2.33 (t, *J* = 7.2 Hz, 2H), 1.77 – 1.65 (m, 2H), 1.66 – 1.56 (m, 4H), 1.41 – 1.29 (m, 4H), 0.91 (t, *J* = 6.8 Hz, 3H). **^{13}C NMR (100 MHz, CDCl$_3$)** δ 173.7, 72.3, 64.5, 58.5, 34.1, 29.0, 28.3, 28.1, 22.3, 21.7, 13.9. **HRMS (ESI-TOF) m/z:** [M+H]$^+$: Calcd. for $C_{11}H_{23}O_3^+$: 203.1642. Found: 203.1643.

Phenethyl 5-methoxypentanoate (4)

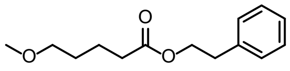

The title compound was prepared following the general procedure, purification by column chromatography on silica gel (petroleum ether/EtOAc = 30:1) yielded (56 mg, 79%) as a colorless oil. **^{1}H NMR (400 MHz, CDCl$_3$)** ^{1}H NMR (400 MHz, Chloroform-*d*) δ 7.41 – 7.36 (m, 2H), 7.33 – 7.26 (m, 2H), 7.24 – 7.17 (m, 1H), 4.24 (t, *J* = 7.2 Hz, 2H), 3.37 (t, *J* = 6.0 Hz, 2H), 3.32 (s, 3H), 3.13 (t, *J* = 6.8 Hz, 2H), 2.30 (t, *J* = 7.2 Hz, 2H), 1.72 – 1.63 (m, 2H), 1.62 – 1.54 (m, 2H). **^{13}C NMR (100 MHz, CDCl$_3$)** δ 173.3, 135.2, 129.9, 129.1, 126.6, 72.2, 62.8, 58.6, 33.8, 32.4, 29.0, 21.6. **HRMS (ESI-TOF) m/z:** [M+H]$^+$: Calcd. for $C_{14}H_{21}O_3^+$: 237.1486. Found: 237.1488.

Benzyl 5-methoxypentanoate (5)

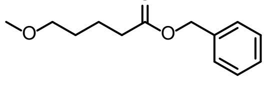

The title compound was prepared following the general procedure, purification by column chromatography on silica gel (petroleum ether/EtOAc = 30:1) yielded (54 mg, 81%) as a colorless oil. **^{1}H NMR (400 MHz, Chloroform-*d*)** δ 7.36 – 7.09 (m, 5H), 5.11 (s, 2H), 3.37 (t, *J* = 6.4 Hz, 2H), 3.31 (s, 3H), 2.39 (t, *J* = 7.2 Hz, 2H), 1.76 – 1.68 (m, 2H), 1.64 – 1.56 (m, 2H). **^{13}C NMR (100 MHz, CDCl$_3$)** δ 173.4, 136.1, 128.6, 128.2, 72.2, 66.1, 58.5, 34.0, 29.0, 21.7. **HRMS (ESI-TOF) m/z:** [M+H]$^+$: Calcd. for $C_{13}H_{19}O_3^+$: 223.1329. Found: 223.1333.

Furan-2-ylmethyl 5-methoxypentanoate (6)

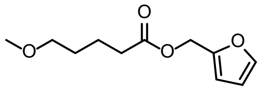

The title compound was prepared following the general procedure, purification by column chromatography on silica gel (petroleum ether/EtOAc = 30:1) yielded (51 mg, 80%) as a colorless oil. **^{1}H NMR (400 MHz, CDCl$_3$)** δ 7.42 (s, 1H), 6.40 (d, *J* = 3.2 Hz, 1H), 6.38 – 6.30 (m, 1H), 5.06 (s, 2H), 3.37 (t, *J* = 6.0 Hz, 2H), 3.31 (s, 3H), 2.37 (t, *J* = 7.2 Hz, 2H), 1.77 – 1.65 (m, 2H), 1.64 – 1.52 (m, 2H). **^{13}C NMR (100 MHz, CDCl$_3$)** δ 173.1, 149.6, 143.2, 110.5, 110.5, 72.2, 58.5, 57.9, 33.8, 28.9, 21.6. **HRMS (ESI-TOF) m/z:** $[M+H]^+$: Calcd. for $C_{11}H_{17}O_4^+$: 213.1122 Found: 213.1125.

Pent-4-yn-1-yl 5-methoxypentanoate (7)

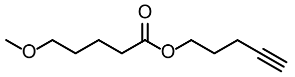

The title compound was prepared following the general procedure, purification by column chromatography on silica gel (petroleum ether/EtOAc = 30:1) yielded (42 mg, 71%) as a colorless oil. **^{1}H NMR (400 MHz, CDCl$_3$)** δ 4.18 (t, *J* = 6.4 Hz, 2H), 3.38 (t, *J* = 6.4 Hz, 2H), 3.33 (s, 3H), 2.34 (t, *J* = 7.2 Hz, 2H), 2.29 (td, *J* = 7.2, 2.8 Hz, 2H), 1.97 (t, *J* = 2.7 Hz, 1H), 1.86 (p, *J* = 6.4 Hz, 2H), 1.75 – 1.66 (m, 2H), 1.65 – 1.56 (m, 2H). **^{13}C NMR (100 MHz, CDCl$_3$)** δ 173.5, 83.0, 72.2, 69.0, 62.8, 58.5, 34.0, 29.0, 27.5, 21.7, 15.2. **HRMS (ESI-TOF) m/z:** $[M+H]^+$: Calcd. for $C_{11}H_{19}O_3^+$: 199.1329. Found: 199.1332.

3-Phenylprop-2-yn-1-yl 5-methoxypentanoate (8)

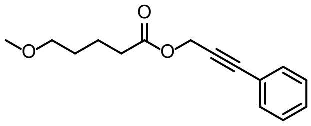

The title compound was prepared following the general procedure, purification by column chromatography on silica gel (petroleum ether/EtOAc = 30:1) yielded (63 mg, 86%) as a colorless oil. **^{1}H NMR (400 MHz, CDCl$_3$)** δ 7.45 (dd, *J* = 6.8, 2.4 Hz, 2H), 7.35 – 7.27 (m, 3H), 4.91 (s, 2H), 3.38 (t, *J* = 6.4 Hz, 2H), 3.31 (s, 3H), 2.41 (t, *J* = 7.2 Hz, 2H), 1.79 – 1.70 (m, 2H), 1.67 – 1.58 (m, 2H). **^{13}C NMR (100 MHz, CDCl$_3$)** δ 172.8, 131.9, 128.7, 128.3, 122.2, 86.4, 83.1, 72.2, 58.5, 52.7, 33.8, 28.9, 21.6. **HRMS (ESI-TOF) m/z:** $[M+H]^+$: Calcd. for $C_{15}H_{19}O_3^+$: 247.1329. Found: 247.1327.

Dec-9-en-1-yl 5-methoxypentanoate (9)

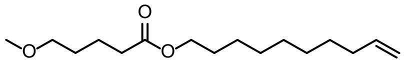

The title compound was prepared following the general procedure, purification by column chromatography on silica gel (petroleum ether/EtOAc = 30:1) yielded (61 mg, 76%) as a colorless oil. **^{1}H NMR (400 MHz, CDCl$_3$)** δ 5.91 – 5.71 (m, 1H), 4.99 (dd, *J* = 17.2, 2.0 Hz, 1H), 4.93 (dd, *J* = 10.0, 2.0 Hz, 1H), 4.06 (t, *J* = 6.8 Hz, 2H), 3.38 (t, *J* = 6.4 Hz, 2H), 3.32 (s, 3H), 2.33 (t, *J* = 7.2 Hz, 2H), 2.04 (q, *J* = 7.2

Hz, 2H), 1.74 – 1.65 (m, 2H), 1.64 – 1.55 (m, 4H), 1.44 – 1.25 (m, 10H). **^{13}C NMR (100 MHz, CDCl$_3$)** δ 173.6, 139.1, 114.2, 72.3, 64.4, 58.5, 34.1, 33.8, 29.3, 29.2, 29.0, 29.0, 28.9, 28.6, 25.9, 21.7. **HRMS (ESI-TOF) m/z:** [M+H]$^+$: Calcd. for $C_{16}H_{31}O_3^+$: 271.2268. Found: 271.2264.

Cyclohexyl 5-methoxypentanoate (10)

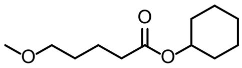

The title compound was prepared following the general procedure, purification by column chromatography on silica gel (petroleum ether/EtOAc = 30:1) yielded (46 mg, 72%) as a colorless oil. **^{1}H NMR (400 MHz, CDCl$_3$)** δ 4.84 – 4.67 (m, 1H), 3.38 (t, *J* = 6.4 Hz, 2H), 3.33 (s, 3H), 2.31 (t, *J* = 7.2 Hz, 2H), 1.94 – 1.80 (m, 2H), 1.76 – 1.51 (m, 8H), 1.46 – 1.32 (m, 4H). **^{13}C NMR (100 MHz, CDCl$_3$)** δ 173.1, 72.4, 72.3, 58.5, 34.5, 31.7, 29.0, 25.4, 23.8, 21.8. **HRMS (ESI-TOF) m/z:** [M+H]$^+$: Calcd. for $C_{12}H_{23}O_3^+$: 215.1642. Found: 215.1645.

Cyclododecyl 5-methoxypentanoate (11)

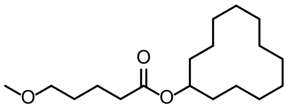

The title compound was prepared following the general procedure, purification by column chromatography on silica gel (petroleum ether/EtOAc = 30:1) yielded (62 mg, 70%) as a colorless oil. **^{1}H NMR (400 MHz, CDCl$_3$)** δ 5.14 – 4.95 (m, 1H), 3.38 (t, *J* = 6.4 Hz, 2H), 3.33 (s, 3H), 2.30 (t, *J* = 7.2 Hz, 2H), 1.85 – 1.64 (m, 4H), 1.64 – 1.55 (m, 2H), 1.56 – 1.46 (m, 2H), 1.42 – 1.28 (m, 18H). **^{13}C NMR (100 MHz, CDCl$_3$)** δ 173.3, 72.3, 72.0, 58.6, 34.4, 29.1, 29.0, 24.1, 23.9, 23.4, 23.2, 21.8, 20.9. **HRMS (ESI-TOF) m/z:** [M+H]$^+$: Calcd. for $C_{18}H_{35}O_3^+$: 299.2581. Found: 299.2574.

Phenyl 5-methoxypentanoate (12)

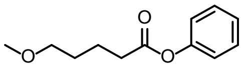

The title compound was prepared following the general procedure, purification by column chromatography on silica gel (petroleum ether/EtOAc = 50:1) yielded (57 mg, 92%) as a colorless oil. **^{1}H NMR (400 MHz, CDCl$_3$)** δ 7.36 (t, *J* = 7.6 Hz, 2H), 7.24 – 7.18 (m, 1H), 7.07 (dd, *J* = 7.6, 1.6 Hz, 2H), 3.43 (t, *J* = 6.4 Hz, 2H), 3.34 (s, 3H), 2.59 (t, *J* = 7.6 Hz, 2H), 1.88 – 1.78 (m, 2H), 1.73 – 1.66 (m, 2H). **^{13}C NMR (100 MHz, CDCl$_3$)** δ 172.1, 150.7, 129.4, 125.7, 121.6, 72.2, 58.6, 34.1, 29.0, 21.7. **HRMS (ESI-TOF) m/z:** [M+H]$^+$: Calcd. for $C_{12}H_{17}O_3^+$: 209.1173. Found: 209.1177.

Benzo[*d*][1,3]dioxol-5-yl 5-methoxypentanoate (13)

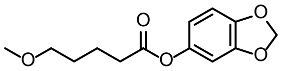

The title compound was prepared following the general procedure, purification by column chromatography on silica gel (petroleum

ether/EtOAc = 30:1) yielded (68 mg, 90%) as a colorless oil. **^{1}H NMR (400 MHz, $CDCl_3$)** δ 6.76 (d, J = 8.4 Hz, 1H), 6.59 (d, J = 2.0 Hz, 1H), 6.51 (dd, J = 8.4, 2.0 Hz, 1H), 5.96 (s, 2H), 3.42 (t, J = 6.0 Hz, 2H), 3.34 (s, 3H), 2.55 (t, J = 7.2 Hz, 2H), 1.91 – 1.75 (m, 2H), 1.73 – 1.62 (m, 2H). **^{13}C NMR (100 MHz, $CDCl_3$)** δ 172.3, 148.0, 145.3, 145.0, 113.9, 107.9, 103.7, 101.7, 72.2, 58.6, 34.0, 29.0, 21.7. **HRMS (ESI-TOF) m/z:** $[M+H]^+$: Calcd. for $C_{13}H_{17}O_5^+$: 253.1071. Found: 253.1078.

***p*-Tolyl 5-methoxypentanoate (14)**

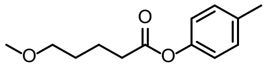

The title compound was prepared following the general procedure, purification by column chromatography on silica gel (petroleum ether/EtOAc = 50:1) yielded (57 mg, 85%) as a colorless oil. **^{1}H NMR (400 MHz, $CDCl_3$)** δ 7.16 (d, J = 8.0 Hz, 2H), 6.95 (d, J = 8.4 Hz, 2H), 3.43 (t, J = 6.4 Hz, 2H), 3.34 (s, 3H), 2.58 (t, J = 7.6 Hz, 2H), 2.33 (s, 3H), 1.91 – 1.78 (m, 2H), 1.75 – 1.64 (m, 2H). **^{13}C NMR (100 MHz, $CDCl_3$)** δ 172.3, 148.5, 135.4, 129.9, 121.2, 72.2, 58.6, 34.1, 29.0, 21.7, 20.9. **HRMS (ESI-TOF) m/z:** $[M+H]^+$: Calcd. for $C_{13}H_{19}O_3^+$: 223.1329. Found: 223.1333.

4-Bromophenyl 5-methoxypentanoate (15)

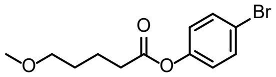

The title compound was prepared following the general procedure, purification by column chromatography on silica gel (petroleum ether/EtOAc = 50:1) yielded (59 mg, 69%) as a colorless oil. **^{1}H NMR (400 MHz, $CDCl_3$)** δ 7.48 (d, J = 8.8 Hz, 2H), 6.97 (d, J = 8.8 Hz, 2H), 3.43 (t, J = 6.0 Hz, 2H), 3.34 (s, 3H), 2.58 (t, J = 7.2 Hz, 2H), 1.89 – 1.77 (m, 2H), 1.73 – 1.64 (m, 2H). **^{13}C NMR (100 MHz, $CDCl_3$)** δ 171.7, 149.7, 132.4, 123.4, 118.8, 72.2, 58.6, 34.0, 28.9, 21.7. **HRMS (ESI-TOF) m/z:** $[M+H]^+$: Calcd. for $C_{12}H_{16}BrO_3^+$: 287.0278. Found: 287.0279.

4-Cyanophenyl 5-methoxypentanoate (16)

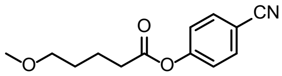

The title compound was prepared following the general procedure, purification by column chromatography on silica gel (petroleum ether/EtOAc = 10:1) yielded (45 mg, 65%) as a colorless oil. **^{1}H NMR (400 MHz, $CDCl_3$)** δ 7.65 – 7.50 (m, 2H), 7.01 – 6.82 (m, 2H), 4.03 (t, J = 6.4 Hz, 2H), 3.49 – 3.42 (m, 2H), 3.35 (s, 3H), 2.02 – 1.84 (m, 2H), 1.80 – 1.71 (m, 2H). **^{13}C NMR (101 MHz, $CDCl_3$)** δ 162.4, 134.0, 119.3, 115.2, 103.7, 72.2, 68.1, 58.6, 26.1, 25.9. **HRMS (ESI-TOF) m/z:** $[M+H]^+$: Calcd. for $C_{13}H_{16}NO_3^+$: 234.1125. Found: 234.1128.

4-Phenoxyphenyl 5-methoxypentanoate (17)

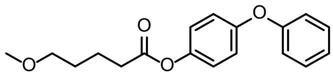

The title compound was prepared following the general procedure, purification by column chromatography on silica gel (petroleum ether/EtOAc = 30:1) yielded (66 mg, 73%) as a colorless oil. **^{1}H NMR (400 MHz, CDCl$_3$)** δ 7.42 – 7.29 (m, 2H), 7.13 – 7.08 (m, 1H), 7.06 – 7.02 (m, 2H), 7.02 – 6.97 (m, 4H), 3.43 (t, J = 6.4 Hz, 2H), 3.34 (s, 3H), 2.59 (t, J = 7.6 Hz, 2H), 1.88 – 1.78 (m, 2H), 1.75 – 1.64 (m, 2H). **^{13}C NMR (100 MHz, CDCl$_3$)** δ 172.2, 157.3, 154.7, 146.1, 129.8, 123.4, 122.7, 119.6, 118.8, 72.2, 58.6, 34.1, 29.0, 21.7. **HRMS (ESI-TOF) m/z:** [M+H]$^+$: Calcd. for $C_{18}H_{21}O_4^+$: 301.1435. Found: 301.1439.

Naphthalen-2-yl 5-methoxypentanoate (18)

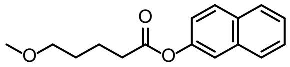

The title compound was prepared following the general procedure, purification by column chromatography on silica gel (petroleum ether/EtOAc = 50:1) yielded (64 mg, 83%) as a colorless oil. **^{1}H NMR (400 MHz, CDCl$_3$)** δ 7.88 – 7.81 (m, 2H), 7.81 – 7.77 (m, 1H), 7.55 (d, J = 2.4 Hz, 1H), 7.51 – 7.42 (m, 2H), 7.21 (dd, J = 8.8, 2.4 Hz, 1H), 3.45 (t, J = 6.4 Hz, 2H), 3.35 (s, 3H), 2.65 (t, J = 7.6 Hz, 2H), 1.96 – 1.82 (m, 2H), 1.78 – 1.68 (m, 2H). **^{13}C NMR (100 MHz, CDCl$_3$)** δ 172.2, 148.4, 133.8, 131.4, 129.4, 127.8, 127.6, 126.6, 125.7, 121.2, 118.5, 72.2, 58.6, 34.2, 29.0, 21.8. **HRMS (ESI-TOF) m/z:** [M+H]$^+$: Calcd. for $C_{16}H_{19}O_3^+$: 259.1329. Found: 259.1325.

2,6-Di-*tert*-butylphenyl 5-methoxypentanoate (19)

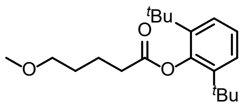

The title compound was prepared following the general procedure, purification by column chromatography on silica gel (petroleum ether/EtOAc = 50:1) yielded (85 mg, 89%) as a colorless oil. **^{1}H NMR (400 MHz, CDCl$_3$)** δ 7.30 (d, J = 7.6 Hz, 2H), 7.11 (t, J = 8.0 Hz, 1H), 3.42 (t, J = 6.4 Hz, 2H), 3.34 (s, 3H), 2.66 (t, J = 7.6 Hz, 2H), 1.93 – 1.78 (m, 2H), 1.77 – 1.64 (m, 2H), 1.33 (s, 18H). **^{13}C NMR (100 MHz, CDCl$_3$)** δ 173.3, 148.1, 142.4, 126.3, 125.5, 72.4, 58.6, 35.5, 35.4, 31.5, 29.2, 21.1. **HRMS (ESI-TOF) m/z:** [M+H]$^+$: Calcd. for $C_{20}H_{33}O_3^+$: 321.2425. Found: 321.2426.

***N*-(*tert*-Butyl)-5-methoxypentanamide (20)**

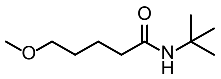

The title compound was prepared following the general procedure, purification by column chromatography on silica gel (petroleum ether/EtOAc = 10:1) yielded (43 mg, 76%) as a colorless oil. **^{1}H NMR (400 MHz, CDCl$_3$)** δ 5.41 (s, 1H), 3.39 (t, J = 6.4 Hz, 2H), 3.33 (s, 3H), 2.13 (d, J = 7.2 Hz, 1H), 1.72 – 1.64 (m, 2H), 1.63 – 1.55 (m, 2H),

1.34 (s, 9H). **^{13}C NMR (100 MHz, CDCl$_3$)** δ 172.2, 72.6, 58.6, 51.0, 37.3, 29.0, 28.8, 22.7. **HRMS (ESI-TOF) m/z:** $[M+H]^+$: Calcd. for $C_{10}H_{22}NO_2^+$: 188.1646. Found: 188.1644.

5-Methoxy-*N*-phenylpentanamide (21)

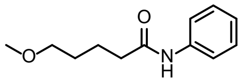

The title compound was prepared following the general procedure, purification by column chromatography on silica gel (petroleum ether/EtOAc = 2:1) yielded (55 mg, 88%) as a colorless oil. **^{1}H NMR (400 MHz, CDCl$_3$)** δ 8.22 (s, 1H), 7.52 (d, *J* = 8.0 Hz, 2H), 7.27 (t, *J* = 8.0 Hz, 2H), 7.06 (t, *J* = 7.2 Hz, 1H), 3.39 (t, *J* = 6.4 Hz, 2H), 3.32 (s, 3H), 2.37 (t, *J* = 7.2 Hz, 2H), 1.84 – 1.73 (m, 2H), 1.69 – 1.58 (m, 2H). **^{13}C NMR (100 MHz, CDCl$_3$)** δ 171.8, 138.2, 128.9, 124.1, 120.0, 72.6, 58.6, 37.2, 28.8, 22.8. **HRMS (ESI-TOF) m/z:** $[M+H]^+$: Calcd. for $C_{12}H_{18}NO_2^+$: 208.1333. Found: 208.1335.

5-Methoxy-*N*-(naphthalen-1-yl)pentanamide (22)

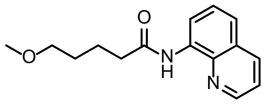

The title compound was prepared following the general procedure, purification by column chromatography on silica gel (petroleum ether/EtOAc = 2:1) yielded (50 mg, 65%) as a colorless oil. **^{1}H NMR (400 MHz, CDCl$_3$)** δ 9.83 (s, 1H), 9.02 – 8.62 (m, 2H), 8.15 (dd, *J* = 8.0, 1.6 Hz, 1H), 7.59 – 7.38 (m, 3H), 3.45 (t, *J* = 6.4 Hz, 2H), 3.35 (s, 2H), 2.60 (t, *J* = 7.2 Hz, 2H), 2.08 – 1.85 (m, 2H), 1.79 – 1.66 (m, 2H). **^{13}C NMR (100 MHz, CDCl$_3$)** δ 171.6, 148.1, 138.4, 136.4, 134.6, 127.9, 127.4, 121.6, 121.4, 116.4, 72.5, 58.6, 37.9, 29.1, 22.5. **HRMS (ESI-TOF) m/z:** $[M+H]^+$: Calcd. for $C_{15}H_{19}N_2O_2^+$: 259.1442. Found: 259.1447.

5-Methoxy-1-(piperidin-1-yl)pentan-1-one (23)

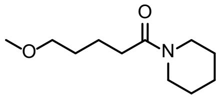

The title compound was prepared following the general procedure, purification by column chromatography on silica gel (petroleum ether/EtOAc = 2:1) yielded (54 mg, 90%) as a colorless oil. **^{1}H NMR (400 MHz, CDCl$_3$)** δ 3.55 (t, *J* = 5.6 Hz, 2H), 3.40 (t, *J* = 6.0 Hz, 4H), 3.33 (s, 3H), 2.35 (t, *J* = 7.2 Hz, 2H), 1.76 – 1.60 (m, 6H), 1.59 – 1.49 (m, 4H). **^{13}C NMR (100 MHz, CDCl$_3$)** δ 171.2, 72.5, 58.5, 46.7, 42.6, 33.1, 29.3, 26.6, 25.6, 24.6, 22.1. **HRMS (ESI-TOF) m/z:** $[M+H]^+$: Calcd. for $C_{11}H_{22}NO_2^+$: 200.1646. Found: 200.1650.

5-Methoxy-*N*-methyl-*N*-phenylpentanamide (24)

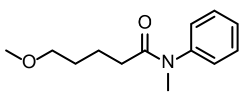

The title compound was prepared following the general procedure, purification by column chromatography on silica gel (petroleum ether/EtOAc = 2:1) yielded (60 mg, 90%) as a colorless oil. **^{1}H NMR (400 MHz, CDCl$_3$)** δ 7.42 (t,

J = 7.6 Hz, 2H), 7.36 – 7.31 (m, 1H), 7.23 – 7.14 (m, 2H), 3.70 – 3.10 (m, 8H), 2.10 (t, J = 7.4 Hz, 2H), 1.69 – 1.59 (m, 2H), 1.54 – 1.42 (m, 2H). **^{13}C NMR (100 MHz, CDCl$_3$)** δ 172.9, 144.2, 129.7, 127.7, 127.3, 72.4, 58.5, 37.3, 33.7, 29.1, 22.2. **HRMS (ESI-TOF) m/z:** [M+H]$^+$: Calcd. for $C_{13}H_{20}NO_2^+$: 222.1489. Found: 222.1491.

***N*-(2,6-Dimethylphenyl)-5-methoxypentanamide (25)**

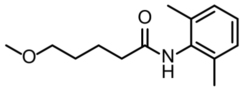

The title compound was prepared following the general procedure, purification by column chromatography on silica gel (petroleum ether/EtOAc = 2:1) yielded (57 mg, 81%) as a colorless oil. **^{1}H NMR (400 MHz, CDCl$_3$)** δ 7.12 – 6.98 (m, 3H), 3.40 (t, J = 6.0 Hz, 2H), 3.33 (s, 3H), 2.37 (t, J = 7.2 Hz, 2H), 2.16 (s, 6H), 1.90 – 1.71 (m, 2H), 1.71 – 1.58 (m, 2H). **^{13}C NMR (100 MHz, CDCl$_3$)** δ 171.5, 135.4, 134.1, 128.1, 127.1, 72.5, 58.6, 36.2, 29.1, 22.9, 18.5, 18.5. **HRMS (ESI-TOF) m/z:** [M+H]$^+$: Calcd. for $C_{14}H_{22}NO_2^+$: 236.1646. Found: 236.1641.

***N*-(2,6-Diisopropylphenyl)-5-methoxypentanamide (26)**

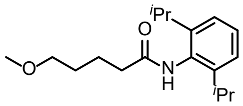

The title compound was prepared following the general procedure, purification by column chromatography on silica gel (petroleum ether/EtOAc = 2:1) yielded (51 mg, 59%) as a colorless soild (the product is a mixture of two isomers, main product : minor product = 3.5 : 1). **^{1}H NMR (400 MHz, CDCl$_3$)** δ 7.32 – 7.26 (m, 1H), 7.17 (d, J = 7.6 Hz, 2H), 6.85 (s, 1H), 3.45 (t, J = 6.4 Hz, 2H), 3.35 (s, 3H), 3.12 – 3.01 (m, 2H), 2.46 (t, J = 7.2 Hz, 2H), 1.92 – 1.78 (m, 2H), 1.77 – 1.67 (m, 2H), 1.20 (s, 6H), 1.18 (s, 6H). **^{13}C NMR (100 MHz, CDCl$_3$)** δ 175.7, 172.2, 147.0, 146.3, 131.2, 129.1, 128.3, 123.9, 123.4, 72.5, 72.4, 58.6, 58.5, 36.5, 31.8, 29.2, 29.1, 28.8, 28.4, 24.7, 23.6, 22.9, 22.6, 21.2. **HRMS (ESI-TOF) m/z:** [M+H]$^+$: Calcd. for $C_{18}H_{30}NO_2^+$: 292.2272. Found: 292.2275.

***N*-Benzyl-*N*-(tert-butyl)-5-methoxypentanamide (27)**

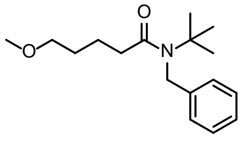

The title compound was prepared following the general procedure, purification by column chromatography on silica gel (petroleum ether/EtOAc = 2:1) yielded (61 mg, 73%) as a colorless oil. **^{1}H NMR (400 MHz, CDCl$_3$)** δ 7.38 – 7.32 (m, 2H), 7.28 – 7.25 (m, 1H), 7.20 (d, J = 7.6 Hz, 2H), 4.59 (s, 2H), 3.33 (t, J = 6.5 Hz, 2H), 3.28 (s, 3H), 2.30 (t, J = 7.2 Hz, 2H), 1.80 – 1.63 (m, 2H), 1.61 – 1.50 (m, 2H), 1.43 (s, 9H). **^{13}C NMR (100 MHz, CDCl$_3$)** δ 174.2, 139.6, 128.8, 127.0, 125.5, 72.6, 58.5, 57.7, 48.6, 35.5, 29.2, 29.1, 28.8, 22.2. **HRMS (ESI-TOF) m/z:** [M+H]$^+$: Calcd. for $C_{17}H_{28}NO_2^+$:

278.2155. Found: 278.2159.

***N,N*-Dicyclohexyl-5-methoxypentanamide (28)**

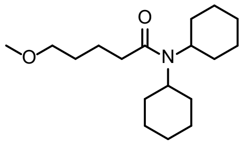

The title compound was prepared following the general procedure, purification by column chromatography on silica gel (petroleum ether/EtOAc = 2:1) yielded (73 mg, 83%) as a colorless oil. **^{1}H NMR (400 MHz, CDCl$_3$)** δ 3.54 – 3.43 (m, 1H), 3.40 (t, *J* = 6.4 Hz, 2H), 3.33 (s, 3H), 2.89 (t, *J* = 6.9 Hz, 1H), 2.31 (t, *J* = 7.2 Hz, 2H), 1.88 – 1.81 (m, 2H), 1.76 (d, *J* = 7.3 Hz, 2H), 1.71 – 1.42 (m, 12H), 1.36 – 1.06 (m, 6H). **^{13}C NMR (100 MHz, CDCl$_3$)** δ 171.8, 72.6, 58.5, 57.7, 55.8, 34.9, 31.4, 30.2, 29.2, 26.6, 26.1, 25.4, 25.3, 22.1. **HRMS (ESI-TOF) m/z:** $[M+H]^+$: Calcd. for $C_{18}H_{34}NO_2^+$: 296.2585. Found: 296.2587.

1-(9*H*-carbazol-9-yl)-5-methoxypentan-1-one (29)

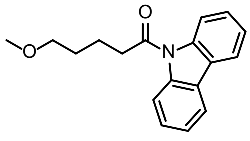

The title compound was prepared following the general procedure, purification by column chromatography on silica gel (petroleum ether/EtOAc = 20:1) yielded (67 mg, 80%) as a colorless oil. **^{1}H NMR (400 MHz, CDCl$_3$)** δ 8.21 (d, *J* = 8.8 Hz, 2H), 7.97 (d, *J* = 7.6 Hz, 2H), 7.52 – 7.43 (m, 2H), 7.36 (t, *J* = 7.6 Hz, 2H), 3.47 (t, *J* = 6.4 Hz, 2H), 3.34 (s, 3H), 3.15 (t, *J* = 7.2 Hz, 2H), 2.07 – 1.94 (m, 2H), 1.85 – 1.73 (m, 2H). **^{13}C NMR (100 MHz, CDCl$_3$)** δ 173.1, 138.6, 127.3, 126.4, 123.5, 119.8, 116.5, 72.5, 58.7, 38.9, 29.1, 21.6. **HRMS (ESI-TOF) m/z:** $[M+H]^+$: Calcd. for $C_{18}H_{20}NO_2^+$: 282.1489. Found: 282.1490.

1-(1*H*-benzo[*d*]imidazol-1-yl)-5-methoxypentan-1-one (30)

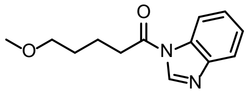

The title compound was prepared following the general procedure, purification by column chromatography on silica gel (petroleum ether/EtOAc = 15:1) yielded (49 mg, 70%) as a colorless oil. **^{1}H NMR (400 MHz, CDCl$_3$)** δ 8.43 (s, 1H), 8.28 – 8.20 (m, 1H), 7.86 – 7.77 (m, 1H), 7.50 – 7.36 (m, 2H), 3.46 (t, *J* = 6.0 Hz, 2H), 3.33 (s, 3H), 3.06 (t, *J* = 7.2 Hz, 2H), 2.07 – 1.92 (m, 2H), 1.86 – 1.68 (m, 2H). **^{13}C NMR (100 MHz, CDCl$_3$)** δ 170.3, 143.9, 141.0, 125.9, 125.0, 120.6, 115.6, 72.3, 58.7, 35.7, 28.8, 21.4. **HRMS (ESI-TOF) m/z:** $[M+H]^+$: Calcd. for $C_{13}H_{17}N_2O_2^+$: 233.1285. Found: 233.1290.

2-(Phenylthio)ethyl pentanoate (31)

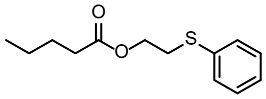

The title compound was prepared following the general procedure, purification by column chromatography on silica gel (petroleum

ether/EtOAc = 100:1) yielded (65 mg, 91%) as a colorless oil. **^{1}H NMR (400 MHz, Chloroform-*d*)** δ 7.42 – 7.37 (m, 2H), 7.33 – 7.26 (m, 2H), 7.23 – 7.17 (m, 1H), 4.23 (t, *J* = 6.8 Hz, 2H), 3.13 (t, *J* = 6.8 Hz, 2H), 2.27 (t, *J* = 7.6 Hz, 2H), 1.58 (p, *J* = 7.6 Hz, 2H), 1.33 (h, *J* = 7.2 Hz, 2H), 0.91 (t, *J* = 7.6 Hz, 3H). **^{13}C NMR (100 MHz, CDCl$_3$)** δ 173.6, 135.2, 129.9, 129.1, 126.6, 62.7, 33.9, 32.4, 26.9, 22.2, 13.7. **HRMS (ESI-TOF) m/z:** [M+H]$^+$: Calcd. for $C_{13}H_{19}O_2S^+$: 239.1101. Found: 239.1107.

2-(Phenylthio)ethyl nonanoate (32)

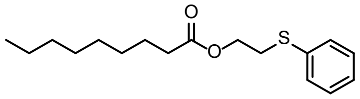

The title compound was prepared following the general procedure, purification by column chromatography on silica gel (petroleum ether/EtOAc = 100:1) yielded (79 mg, 90%) as a colorless oil. **^{1}H NMR (400 MHz, CDCl$_3$)** δ 7.39 (d, *J* = 7.6 Hz, 2H), 7.29 (t, *J* = 7.6 Hz, 2H), 7.20 (t, *J* = 7.2 Hz, 1H), 4.23 (t, *J* = 7.2 Hz, 2H), 3.13 (t, *J* = 6.8 Hz, 2H), 2.26 (t, *J* = 7.2 Hz, 2H), 1.65 – 1.53 (m, 2H), 1.29 – 1.24 (m, 10H), 0.88 (t, *J* = 6.4 Hz, 3H). **^{13}C NMR (100 MHz, CDCl$_3$)** δ 173.6, 135.2, 129.8, 129.1, 126.5, 62.7, 34.2, 32.4, 31.8, 29.2, 29.1, 24.9, 22.7, 14.1. **HRMS (ESI-TOF) m/z:** [M+H]$^+$: Calcd. for $C_{17}H_{27}O_2S^+$: 295.1727. Found: 295.1732.

2-(Phenylthio)ethyl 6-oxoheptanoate (33)

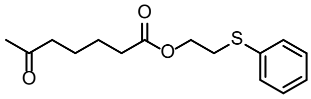

The title compound was prepared following the general procedure, purification by column chromatography on silica gel (petroleum ether/EtOAc = 30:1) yielded (76 mg, 90%) as a colorless oil. **^{1}H NMR (400 MHz, CDCl$_3$)** δ 7.45 – 7.37 (m, 2H), 7.30 (t, *J* = 7.6 Hz, 2H), 7.24 – 7.17 (m, 1H), 4.24 (t, *J* = 7.2 Hz, 2H), 3.14 (t, *J* = 6.8 Hz, 2H), 2.49 – 2.39 (m, 2H), 2.33 – 2.24 (m, 2H), 2.13 (s, 3H), 1.69 – 1.54 (m, 4H). **^{13}C NMR (100 MHz, CDCl$_3$)** δ 208.5, 173.1, 135.1, 129.9, 129.1, 126.6, 62.8, 43.2, 33.8, 32.4, 29.9, 24.3, 23.1. **HRMS (ESI-TOF) m/z:** [M+H]$^+$: Calcd. for $C_{15}H_{21}O_3S^+$: 281.1206. Found: 281.1204.

2-(Phenylthio)ethyl hex-5-ynoate (34)

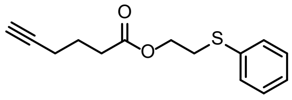

The title compound was prepared following the general procedure, purification by column chromatography on silica gel (petroleum ether/EtOAc = 100:1) yielded (64 mg, 86%) as a colorless oil. **^{1}H NMR (400 MHz, Chloroform-*d*)** δ 7.43 – 7.37 (m, 2H), 7.30 (t, *J* = 7.6 Hz, 2H), 7.24 – 7.18 (m, 1H), 4.25 (t, *J* = 6.8 Hz, 2H), 3.14 (t, *J* = 6.8 Hz, 2H), 2.41 (t, *J* = 7.6 Hz, 2H), 2.25 (td, *J* = 6.8, 2.4 Hz, 2H), 2.00 – 1.93 (m, 1H), 1.88 – 1.76 (m, 2H). **^{13}C NMR (100 MHz, CDCl$_3$)** δ 172.8, 135.1, 129.9, 129.1, 126.6, 83.2, 69.2, 62.9,

32.7, 32.4, 23.5, 17.8. **HRMS (ESI-TOF) m/z:** $[M+H]^+$: Calcd. for $C_{14}H_{17}O_2S^+$: 249.0944. Found: 249.0948.

2-(Phenylthio)ethyl 4-cyanobutanoate (35)

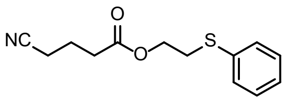

The title compound was prepared following the general procedure, purification by column chromatography on silica gel (petroleum ether/EtOAc = 10:1) yielded (61 mg, 82%) as a colorless oil. **^{1}H NMR (700 MHz, CDCl$_3$)** δ 7.39 (d, J = 8.4 Hz, 2H), 7.30 (t, J = 7.7 Hz, 2H), 7.24 – 7.20 (m, 1H), 4.27 (td, J = 7.0, 1.4 Hz, 2H), 3.15 (td, J = 7.0, 1.4 Hz, 2H), 2.50 – 2.40 (m, 4H), 2.00 – 1.90 (m, 2H). **^{13}C NMR (175 MHz, CDCl$_3$)** δ 171.7, 135.0, 130.2, 130.0, 129.1, 129.1, 126.8, 119.0, 63.3, 32.5, 32.2, 20.7, 16.5. **HRMS (ESI-TOF) m/z:** $[M+H]^+$: Calcd. for $C_{13}H_{16}O_2S^+$: 250.0897. Found: 250.0899.

2-(Phenylthio)ethyl 7-hydroxyheptanoate (36)

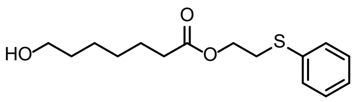

The title compound was prepared following the general procedure, purification by column chromatography on silica gel (petroleum ether/EtOAc = 5:1) yielded (52 mg, 62%) as a colorless oil. **^{1}H NMR (400 MHz, CDCl$_3$)** δ 7.39 (d, J = 7.6 Hz, 2H), 7.32 – 7.26 (m, 2H), 7.21 (t, J = 7.2 Hz, 1H), 4.24 (t, J = 6.8 Hz, 2H), 3.63 (t, J = 6.8 Hz, 2H), 3.14 (t, J = 6.8 Hz, 2H), 2.28 (t, J = 7.6 Hz, 2H), 1.69 – 1.51 (m, 4H), 1.42 – 1.29 (m, 4H). **^{13}C NMR (100 MHz, CDCl$_3$)** δ 173.6, 135.2, 129.9, 129.1, 126.6, 62.9, 62.8, 34.0, 32.5, 32.4, 28.9, 25.4, 24.8. **HRMS (ESI-TOF) m/z:** $[M+H]^+$: Calcd. for $C_{15}H_{23}O_3S^+$: 283.1363. Found: 283.1361.

2-(Phenylthio)ethyl 4-(trimethylsilyl)butanoate (37)

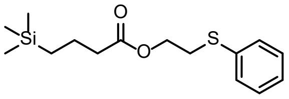

The title compound was prepared following the general procedure, purification by column chromatography on silica gel (petroleum ether/EtOAc = 100:1) yielded (70 mg, 79%) as a colorless oil. **^{1}H NMR (400 MHz, CDCl$_3$)** δ 7.42 – 7.38 (m, 2H), 7.30 (t, J = 7.6 Hz, 2H), 7.25 – 7.19 (m, 1H), 4.25 (t, J = 7.2 Hz, 2H), 3.15 (t, J = 7.2 Hz, 2H), 2.30 (t, J = 7.2 Hz, 2H), 1.69 – 1.56 (m, 2H), 0.56 – 0.48 (m, 2H), 0.00 (s, 9H). **^{13}C NMR (100 MHz, CDCl$_3$)** δ 175.2, 137.0, 131.6, 130.8, 128.3, 64.4, 39.5, 34.2, 21.5, 18.2, 0.0. **HRMS (ESI-TOF) m/z:** $[M+H]^+$: Calcd. for $C_{15}H_{25}O_2SSi^+$: 297.1340. Found: 297.1348.

2-(Phenylthio)ethyl 5-chloropentanoate (38)

The title compound was prepared following the general procedure, purification by column chromatography on silica gel (petroleum ether/EtOAc = 100:1) yielded (73 mg, 90%) as a colorless

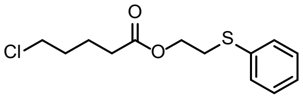

oil. **^{1}H NMR (700 MHz, CDCl$_3$)** δ 7.42 – 7.36 (m, 2H), 7.30 (t, *J* = 7.7 Hz, 2H), 7.23 – 7.18 (m, 1H), 4.25 (t, *J* = 7.0 Hz, 2H), 3.53 (t, *J* = 6.3 Hz, 2H), 3.14 (t, *J* = 7.0 Hz, 2H), 2.30 (t, *J* = 7.7 Hz, 2H), 1.85 – 1.77 (m, 2H), 1.77 – 1.70 (m, 2H). **^{13}C NMR (175 MHz, CDCl$_3$)** δ 172.9, 135.1, 129.9, 129.1, 126.6, 62.9, 44.4, 33.2, 32.4, 31.8, 22.2. **HRMS (ESI-TOF) m/z:** [M+H]$^+$: Calcd. for $C_{13}H_{18}ClO_2S^+$: 273.0711. Found: 273.0715.

2-(Phenylthio)ethyl cyclopropanecarboxylate (39)

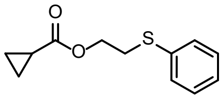

The title compound was prepared following the general procedure, purification by column chromatography on silica gel (petroleum ether/EtOAc = 100:1) yielded (49 mg, 73%) as a colorless oil. **^{1}H NMR (400 MHz, CDCl$_3$)** δ 7.43 – 7.36 (m, 2H), 7.29 (t, *J* = 7.6 Hz, 2H), 7.23 – 7.16 (m, 1H), 4.24 (t, *J* = 7.2 Hz, 2H), 3.14 (t, *J* = 7.2 Hz, 2H), 1.69 – 1.51 (m, 1H), 1.06 – 0.94 (m, 2H), 0.92 – 0.80 (m, 2H). **^{13}C NMR (100 MHz, CDCl$_3$)** δ 174.7, 135.2, 129.8, 129.1, 126.5, 62.9, 32.4, 12.8, 8.6. **HRMS (ESI-TOF) m/z:** [M+H]$^+$: Calcd. for $C_{12}H_{15}O_2S^+$: 223.0788. Found: 223.0789.

2-(Phenylthio)ethyl cyclobutanecarboxylate (40)

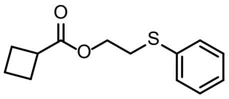

The title compound was prepared following the general procedure, purification by column chromatography on silica gel (petroleum ether/EtOAc = 100:1) yielded (43 mg, 61%) as a colorless oil. **^{1}H NMR (400 MHz, CDCl$_3$)** δ 7.42 – 7.38 (m, 2H), 7.33 – 7.25 (m, 2H), 7.23 – 7.17 (m, 1H), 4.24 (t, *J* = 6.8 Hz, 2H), 3.25 – 3.05 (m, 3H), 2.48 – 2.12 (m, 4H), 2.04 – 1.83 (m, 2H). **^{13}C NMR (100 MHz, CDCl$_3$)** δ 175.3, 135.2, 129.8, 129.1, 126.5, 62.8, 38.0, 32.4, 25.2, 18.4. **HRMS (ESI-TOF) m/z:** [M+H]$^+$: Calcd. for $C_{13}H_{17}O_2S^+$: 269.1206. Found: 269.1211.

2-(Phenylthio)ethyl cyclopentanecarboxylate (41)

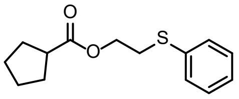

The title compound was prepared following the general procedure, purification by column chromatography on silica gel (petroleum ether/EtOAc = 100:1) yielded (54 mg, 76%) as a colorless oil. **^{1}H NMR (400 MHz, CDCl$_3$)** δ 7.43 – 7.37 (m, 2H), 7.29 (t, *J* = 7.6 Hz, 2H), 7.22 – 7.17 (m, 1H), 4.23 (t, *J* = 6.8 Hz, 2H), 3.13 (t, *J* = 6.8 Hz, 2H), 2.76 – 2.64 (m, 1H), 1.91 – 1.81 (m, 2H), 1.81 – 1.72 (m, 2H), 1.70 – 1.63 (m, 2H), 1.62 – 1.50 (m, 2H). **^{13}C NMR (100 MHz, CDCl$_3$)** δ 176.6, 135.3, 129.8, 129.1, 126.5, 62.7, 43.7, 32.4, 30.0, 25.8. **HRMS (ESI-TOF) m/z:** [M+H]$^+$: Calcd. for $C_{14}H_{19}O_2S^+$: 251.1101. Found: 251.1107.

2-(Phenylthio)ethyl cyclohexanecarboxylate (42)

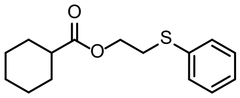

The title compound was prepared following the general procedure, purification by column chromatography on silica gel (petroleum ether/EtOAc = 100:1) yielded (57 mg, 72%) as a colorless oil. **^{1}H NMR (400 MHz, CDCl$_3$)** δ 7.34 – 7.28 (m, 2H), 7.24 – 7.18 (m, 2H), 7.16 – 7.10 (m, 1H), 4.15 (t, *J* = 6.8 Hz, 2H), 3.05 (t, *J* = 6.8 Hz, 2H), 2.18 (tt, *J* = 11.3, 3.6 Hz, 1H), 1.86 – 1.76 (m, 2H), 1.70 – 1.62 (m, 2H), 1.59 – 1.51 (m, 1H), 1.44 – 1.28 (m, 2H), 1.24 – 1.05 (m, 4H). **^{13}C NMR (100 MHz, CDCl$_3$)** δ 175.8, 135.3, 129.9, 129.1, 126.5, 62.6, 43.1, 32.5, 29.0, 25.7, 25.4. **HRMS (ESI-TOF) m/z:** [M+H]$^+$: Calcd. for $C_{14}H_{19}O_2S^+$: 251.1101. Found: 251.1103.

(*S*)-2,5,6,8-Tetramethyl-2-((3*R*,7*R*)-3,7,11-trimethyldodecyl)chroman-7-yl 5-methoxypentanoate (43)

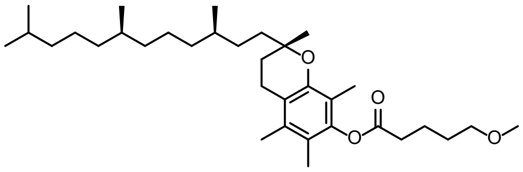

The title compound was prepared following the general procedure, purification by column chromatography on silica gel (petroleum ether/EtOAc = 30:1) yielded (132 mg, 83%) as a colorless oil. **^{1}H NMR (400 MHz, CDCl$_3$)** δ 3.43 (t, *J* = 6.4 Hz, 2H), 3.34 (s, 3H), 2.72 – 2.54 (m, 4H), 2.08 (s, 3H), 2.00 (s, 3H), 1.96 (s, 3H), 1.91 – 1.82 (m, 2H), 1.81 – 1.67 (m, 4H), 1.60 – 1.50 (m, 2H), 1.44 – 1.34 (m, 4H), 1.30 – 1.19 (m, 10H), 1.17 – 1.02 (m, 6H), 0.92 – 0.80 (m, 12H). **^{13}C NMR (100 MHz, CDCl$_3$)** δ 172.1, 149.4, 140.5, 126.7, 124.9, 123.0, 117.3, 75.0, 72.3, 58.6, 39.4, 37.5, 37.5, 37.5, 37.3, 33.9, 32.8, 32.7, 29.2, 28.0, 24.8, 24.5, 22.8, 22.7, 21.9, 21.0, 20.6, 19.8, 19.7, 13.0, 12.2, 11.9. **HRMS (ESI-TOF) m/z:** [M+H]$^+$: Calcd. for $C_{34}H_{59}O_4^+$: 531.4408. Found: 531.4405.

13-Methyl-17-oxo-7,8,9,11,12,13,14,15,16,17-decahydro-6*H*-cyclopenta[*a*]phenanthren-3-yl 5-methoxypentanoate (44)

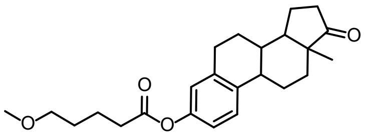

The title compound was prepared following the general procedure, purification by column chromatography on silica gel (petroleum ether/EtOAc = 20:1) yielded (87 mg, 76%) as a colorless oil. **^{1}H NMR (400 MHz, CDCl$_3$)** δ 7.26 (d, *J* = 8.8 Hz, 1H), 6.84 (dd, *J* = 8.8, 2.8 Hz, 1H), 6.80 (d, *J* = 2.4 Hz, 1H), 3.42 (t, *J* = 6.4 Hz, 2H), 3.33 (s, 3H), 2.95 – 2.85 (m, 2H), 2.57 (t, *J* = 7.2 Hz, 2H), 2.53 – 2.44 (m, 1H), 2.41 – 2.34 (m, 1H), 2.31 – 2.22 (m, 1H), 2.19 – 1.92 (m, 4H), 1.89 – 1.76 (m, 2H), 1.75 – 1.66 (m, 2H), 1.65 – 1.39 (m, 6H), 0.89 (s, 3H). **^{13}C NMR (100 MHz, CDCl$_3$)**

δ 220.6, 172.2, 148.6, 137.9, 137.3, 126.3, 121.6, 118.8, 72.2, 58.6, 50.4, 47.9, 44.1, 38.0, 35.8, 34.1, 31.6, 29.4, 29.0, 26.3, 25.8, 21.8, 21.6, 13.8. **HRMS (ESI-TOF) m/z:** $[M+H]^+$: Calcd. for $C_{24}H_{33}O_4^+$: 385.2374. Found: 385.2378.

(4*R*,6*S*)-6-((*S*)-2,2-dimethyl-1,3-dioxolan-4-yl)-2,2-dimethyltetrahydrofuro[3,4-*d*][1,3]dioxol-4-yl 5-methoxypentanoate (45)

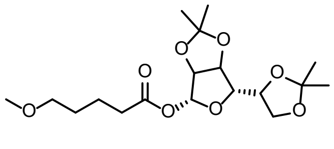

The title compound was prepared following the general procedure, purification by column chromatography on silica gel (petroleum ether/EtOAc = 3:1) yielded (95 mg, 85%) as a colorless oil. **^{1}H NMR (400 MHz, CDCl$_3$)** δ 6.14 (s, 1H), 4.85 (dd, *J* = 6.0, 3.6 Hz, 1H), 4.69 (d, *J* = 5.6 Hz, 1H), 4.44 – 4.36 (m, 1H), 4.12 – 4.07 (m, 1H), 4.06 – 3.99 (m, 2H), 3.39 (t, *J* = 6.0 Hz, 2H), 3.33 (s, 3H), 2.50 – 2.30 (m, 2H), 1.80 – 1.66 (m, 2H), 1.65 – 1.56 (m, 2H), 1.48 (s, 3H), 1.46 (s, 3H), 1.38 (s, 3H), 1.34 (s, 3H). **^{13}C NMR (100 MHz, CDCl$_3$)** δ 171.9, 113.2, 109.3, 100.6, 85.0, 82.2, 79.3, 72.8, 72.1, 66.8, 58.5, 33.9, 28.8, 26.9, 25.9, 25.1, 24.6, 21.4. **HRMS (ESI-TOF) m/z:** $[M+H]^+$: Calcd. for $C_{18}H_{31}O_8^+$: 375.2104. Found: 375.2105.

10,13-Dimethyl-3-oxo-2,3,6,7,8,9,10,11,12,13,14,15,16,17-tetradecahydro-1*H*-cyclopenta[a]phenanthren-17-yl 5-methoxypentanoate (46)

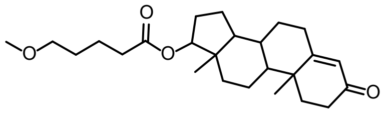

The title compound was prepared following the general procedure, purification by column chromatography on silica gel (petroleum ether/EtOAc = 20:1) yielded (102 mg, 85%) as a colorless oil. ^{1}H NMR (400 MHz, Chloroform-*d*) δ 5.73 (s, 1H), 4.61 (d, *J* = 8.4 Hz, 1H), 3.39 (t, *J* = 6.4 Hz, 2H), 3.33 (s, 3H), 2.51 – 2.26 (m, 6H), 2.22 – 2.12 (m, 1H), 2.06 – 2.00 (m, 1H), 1.90 – 1.82 (m, 1H), 1.82 – 1.75 (m, 1H), 1.72 – 1.54 (m, 8H), 1.52 – 1.21 (m, 4H), 1.19 (s, 3H), 1.12 – 0.91 (m, 3H), 0.84 (s, 3H). ^{13}C NMR (101 MHz, CDCl$_3$) δ 199.4, 173.5, 170.9, 124.0, 82.3, 72.3, 58.6, 53.7, 50.2, 42.5, 38.6, 36.6, 35.7, 35.4, 34.2, 33.9, 32.7, 31.5, 29.0, 27.5, 23.5, 21.8, 20.5, 17.4, 12.1. **HRMS (ESI-TOF) m/z:** $[M+H]^+$: Calcd. for $C_{25}H_{39}O_4^+$: 403.2843. Found: 403.2841.

Dimethyl adipate (47)

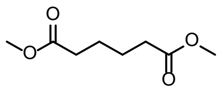

The title compound was prepared following the general procedure, purification by column chromatography on silica gel (petroleum ether/EtOAc = 20:1) yielded (32 mg, 62%) as a colorless oil. **^{1}H NMR (400 MHz, CDCl$_3$)** δ 3.67 (s, 6H), 2.47 – 2.27 (m, 4H), 1.66 (td, *J* = 6.4, 5.6, 3.2 Hz, 4H). **^{13}C NMR (100 MHz, CDCl$_3$)** δ 173.8, 51.5, 33.7,

24.4. **HRMS (ESI-TOF) m/z:** $[M+H]^+$: Calcd. for $C_8H_{15}O_4^+$: 175.0965. Found: 175.0967.

2-(Phenylthio)ethyl octanoate (52)

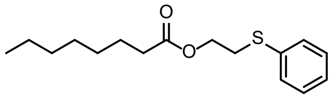

The title compound was prepared by using **Co-B** as substrate, purification by column chromatography on silica gel (petroleum ether/EtOAc = 100:1) yielded (49 mg, 58%) as a colorless oil. **^{1}H NMR (400 MHz, CDCl$_3$)** δ 7.34 – 7.27 (m, 2H), 7.21 (t, *J* = 7.6 Hz, 2H), 7.12 (t, *J* = 7.2 Hz, 1H), 4.16 (t, *J* = 6.8 Hz, 2H), 3.05 (t, *J* = 6.8 Hz, 2H), 2.18 (t, *J* = 7.2 Hz, 2H), 1.60 – 1.45 (m, 2H), 1.34 – 1.14 (m, 10H), 0.80 (t, *J* = 6.8 Hz, 3H). **^{13}C NMR (100 MHz, CDCl$_3$)** δ 134.2, 128.8, 128.0, 125.5, 61.7, 33.1, 31.4, 30.6, 28.0, 27.9, 23.9, 21.6, 13.0. **HRMS (ESI-TOF) m/z:** $[M+H]^+$: Calcd. for $C_{16}H_{25}O_2S^+$: 281.1570. Found: 281.1577.

2,6-Di-*tert*-butyl-4-methylphenyl 5-methoxypentanoate (53)

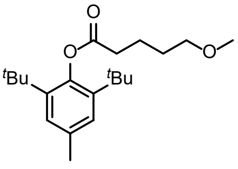

The title compound was prepared following the general procedure, purification by column chromatography on silica gel (petroleum ether/EtOAc = 50:1) yielded (90 mg, 90%) as a colorless oil. **^{1}H NMR (700 MHz, CDCl$_3$)** δ 7.10 (s, 2H), 3.42 (t, *J* = 6.3 Hz, 2H), 3.34 (s, 3H), 2.64 (t, *J* = 7.7 Hz, 2H), 2.31 (s, 3H), 1.82 (p, *J* = 7.7 Hz, 2H), 1.70 (s, 18H), 1.31 (s, 5H). **^{13}C NMR (175 MHz, CDCl$_3$)** δ 173.5, 145.9, 142.0, 134.4, 127.1, 72.4, 58.6, 35.5, 35.2, 31.5, 29.2, 21.5, 21.1. **HRMS (ESI-TOF) m/z:** $[M+H]^+$: Calcd. for $C_{21}H_{35}O_3^+$: 335.2581. Found: 335.2588.

8. Reference

1. (a) Zheng, P.-R.; Xu, W.-H.; Wang, H.-P.; Wang, D.; Wu, X.-Q.; XU, T. Deoxygenative Arylboration of Aldehydes via Copper and Nickel/Photoredox Catalysis. *ACS Catal.* **2022**, *12* (24), 14926−14933. (b) Biswas, S.; Weix, D. J. Mechanism and Selectivity in Nickel-Catalyzed Cross-Electrophile Coupling of Aryl Haldies with Alkyl Halides. *J. Am. Chem. Soc.* **2013**, *135* (43), 16192-16197. (c) Breitenfeld, J.; Ruiz, J.; Wodrich, M. D.; Hu, X. Bimetallic Oxidative Addition Involving Radical Intermediates in Nickel Catalyzed Alkyl-Alkyl Kumada Coupling Reactions. *J. Am. Chem. Soc.* **2013**, *135* (32), 12004-12012.

2. Faculak, M. S.; Veatch, A. M.; Alexanian, E. J. Cobalt-catalyzed synthesis of amides from alkenes and amines promoted by light. *Science*. **2024**, 383 (6678), 77–81.

3. Zubaydi, S. A.; Onuigbo, I. O.; Truesdell, B. L.; Sevov, C. S. Cobalt-Catalyzed Electroreductive Alkylation of Unactivated Alkyl Chlorides with Conjugated Olefins. *Angew. Chem. Int. Ed.* **2024**, *63*, e202313830.

4. Møller, M. S.; Kongsted, J.; McKenzie C. J. Preparation of organocobalt(III) complexes via O_2 activation. *Dalton Trans.* **2021**, *50* (14), 4819–4829.

5. Jiang, D.-H.; Li, X.-Z.; Xiao, M.-D.; Cheng, L.-J. Cobalt-Catalyzed Intramolecular Markovnikov Hydrocarbonylation of Unactivated Alkenes via Hydrogen Atom Transfer. *Angew. Chem. Int. Ed.* **2024**, *63*, e202412828.

6. Sharif, S.; Rodriguez, M. J.; Lu, Y.; Kopach, M. E.; Mitchell, D.; Hunter, H. N.; Organ, M. G. Sodium Butylated Hydroxytoluene (NaBHT) as a New and Efficient Hydride Source for Pd-Catalysed Reduction Reactions. *Chem. Eur. J.* **2019***, 25* (57), 13099 – 13103.

7. Wang, J.-H.; Wang, S.-C.; Wei, Z.-H.; Wang, P.-J.; Cao, Y.-W.; Huang, Y.; He, L.; Lei, A.-W. *Science*. **2024**, *386*, 776–782.

9. Spectra of compounds

[1]**H NMR** spectrum of **1** ($CDCl_3$)

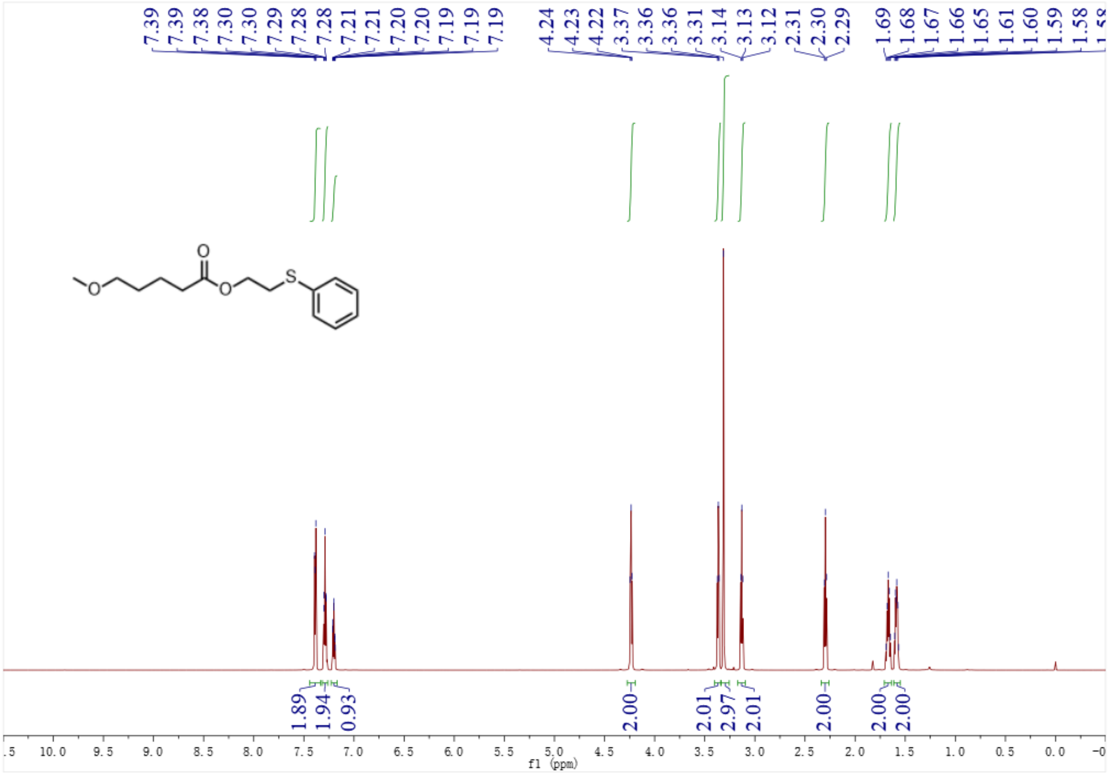

[13]**C NMR** spectrum of **1** ($CDCl_3$)

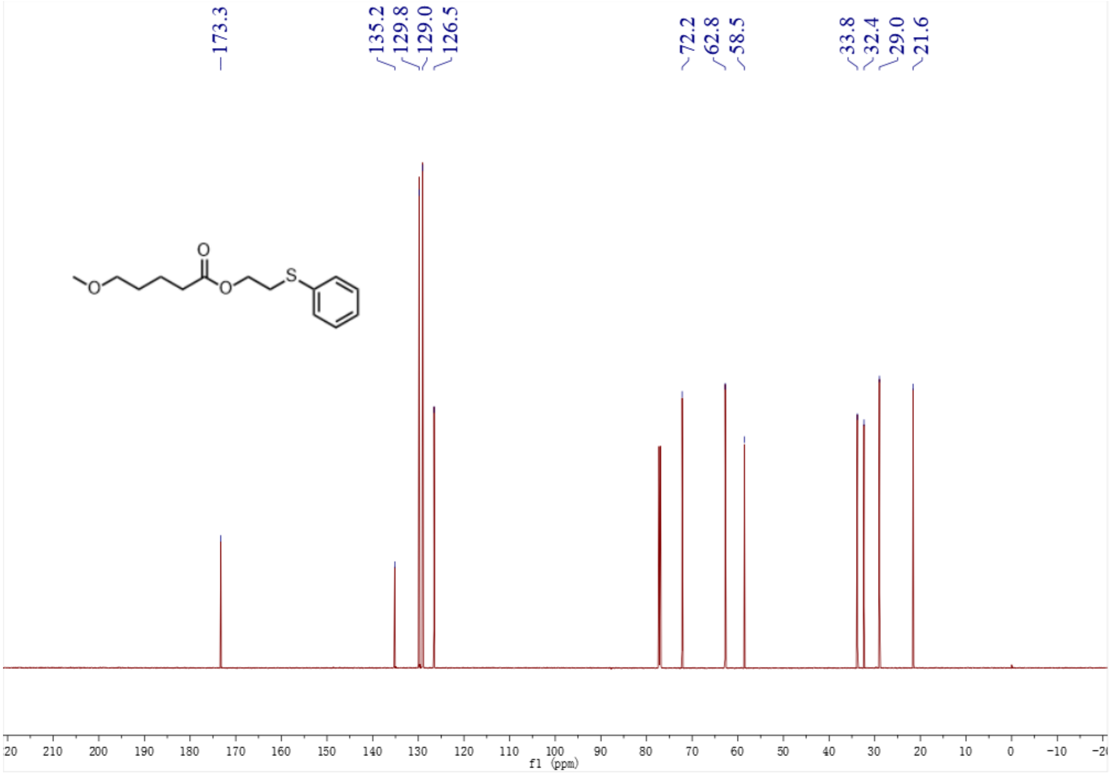

^{1}H NMR spectrum of **2** ($CDCl_3$)

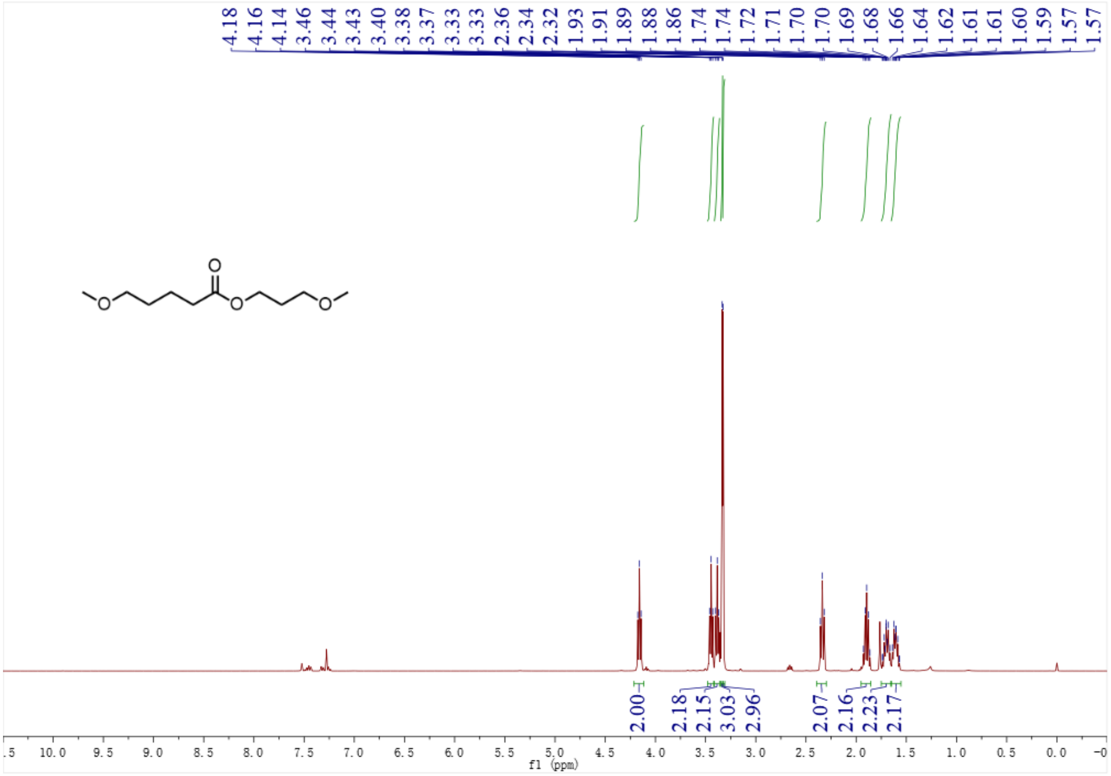

^{13}C NMR spectrum of **2** ($CDCl_3$)

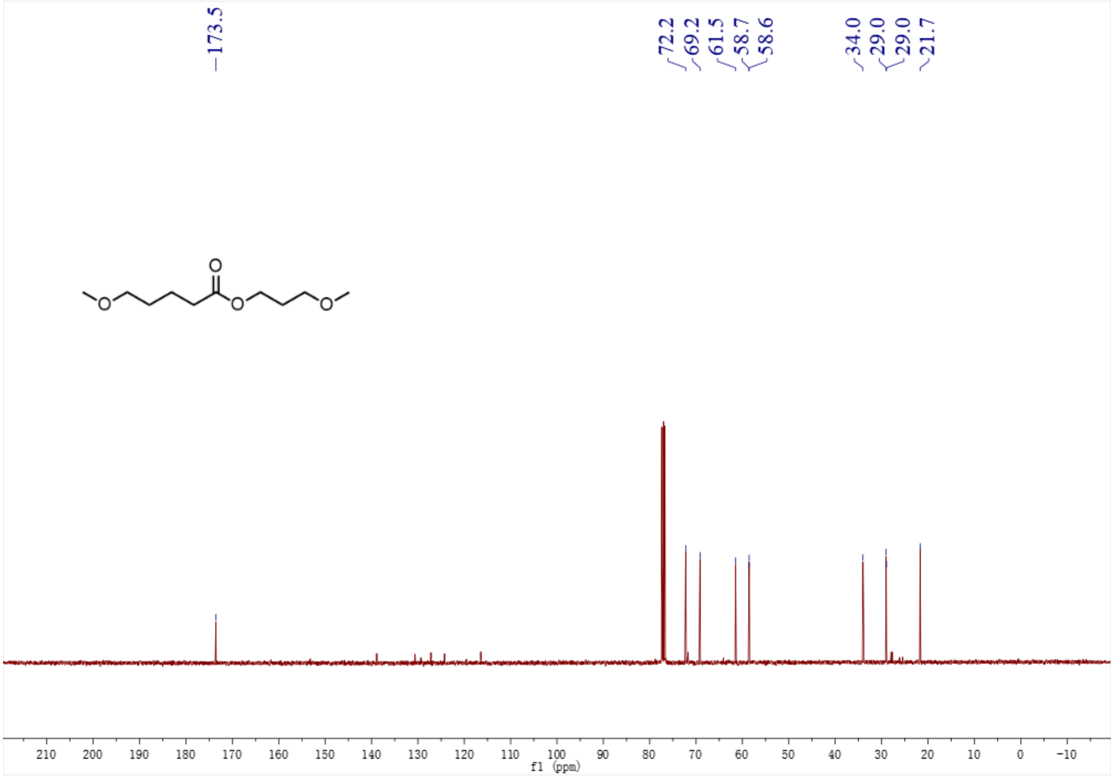

^{1}H NMR spectrum of **3** ($CDCl_3$)

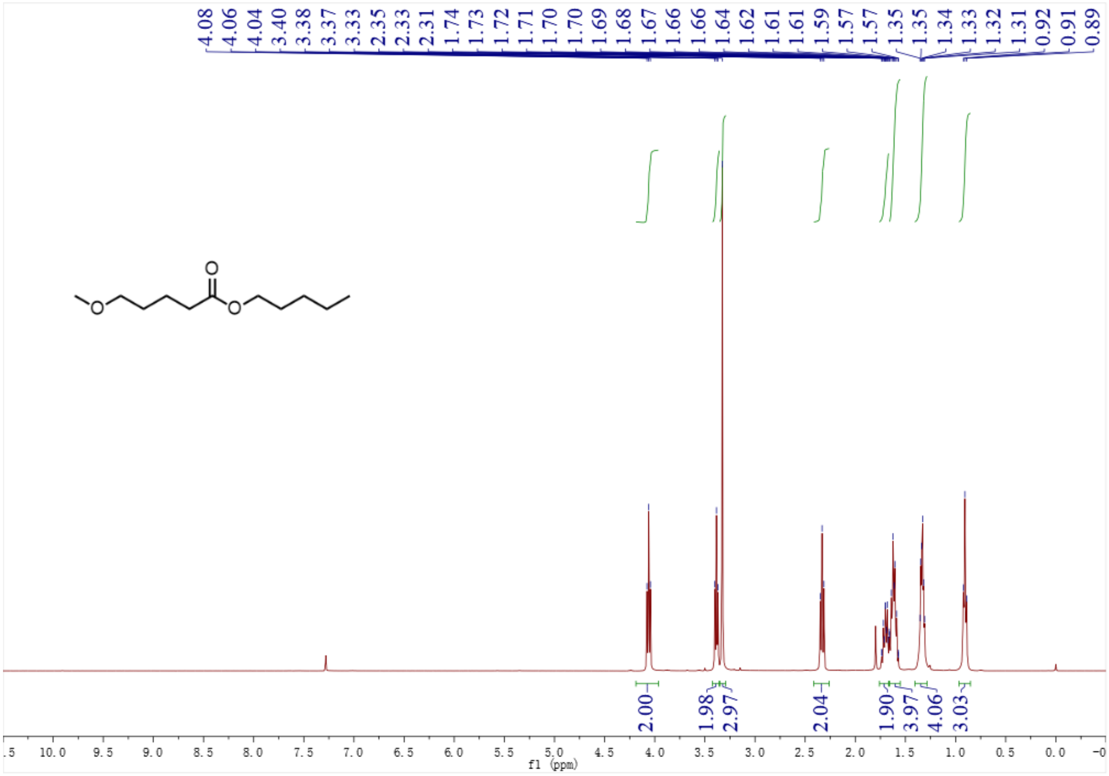

^{13}C NMR spectrum of **3** ($CDCl_3$)

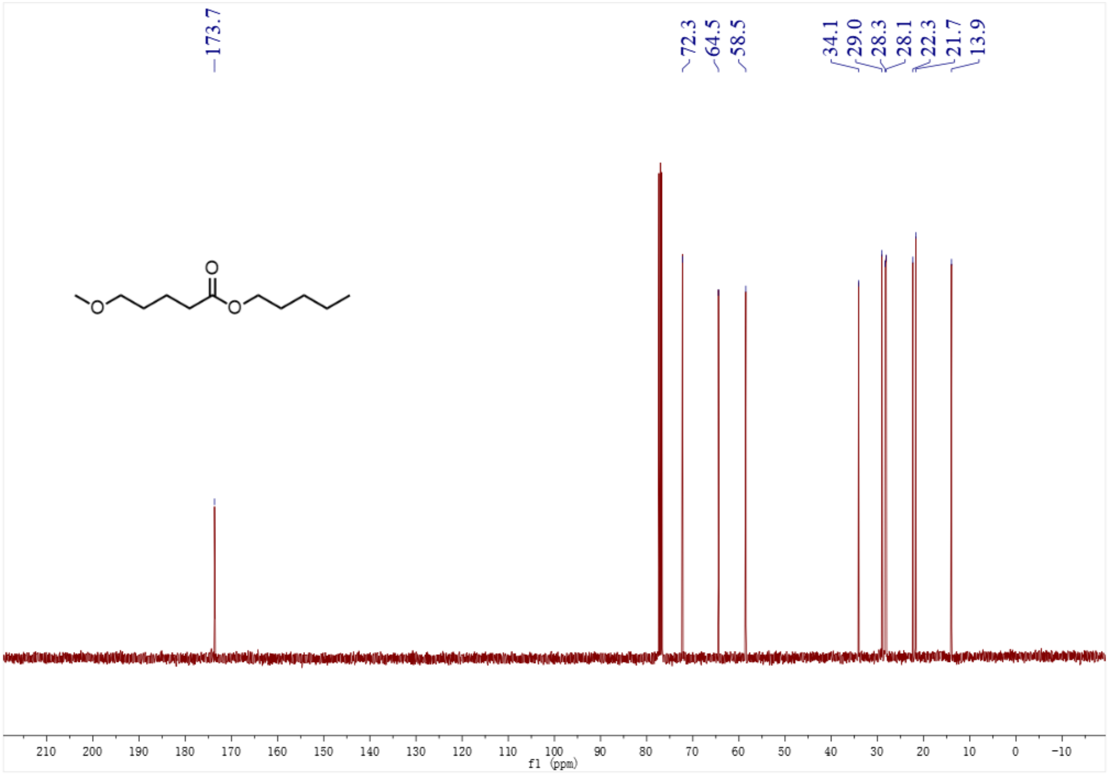

^{1}H NMR spectrum of **4** ($CDCl_3$)

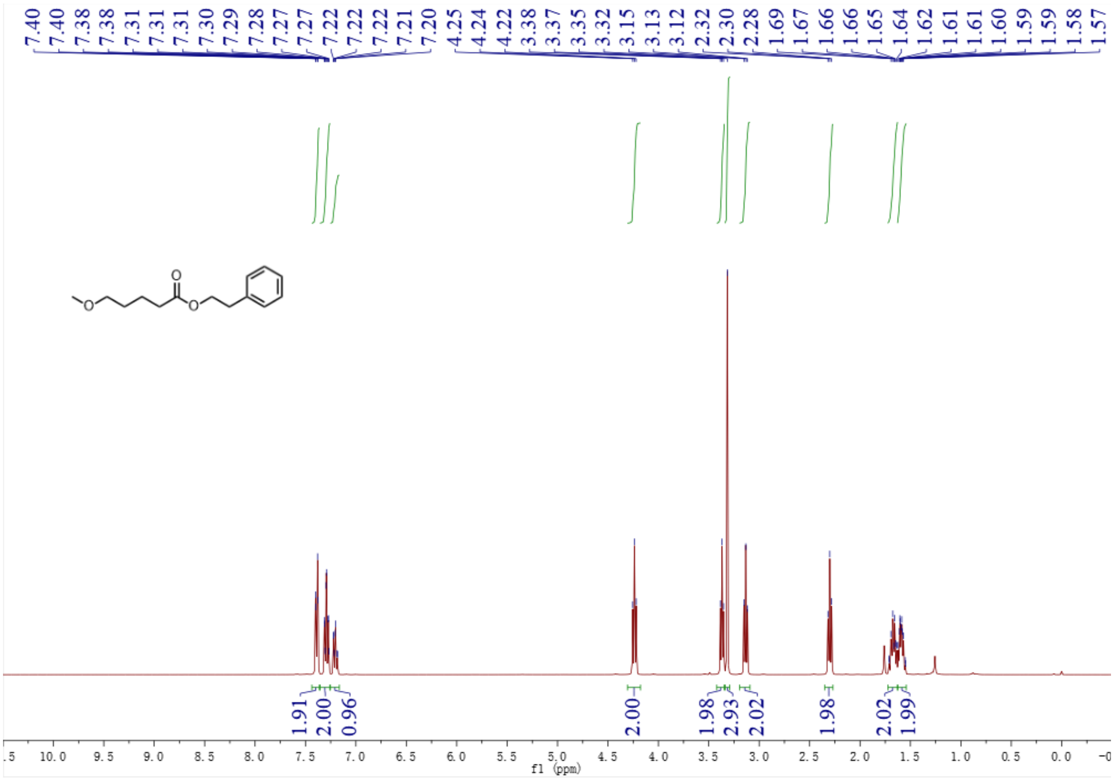

^{13}C NMR spectrum of **4** ($CDCl_3$)

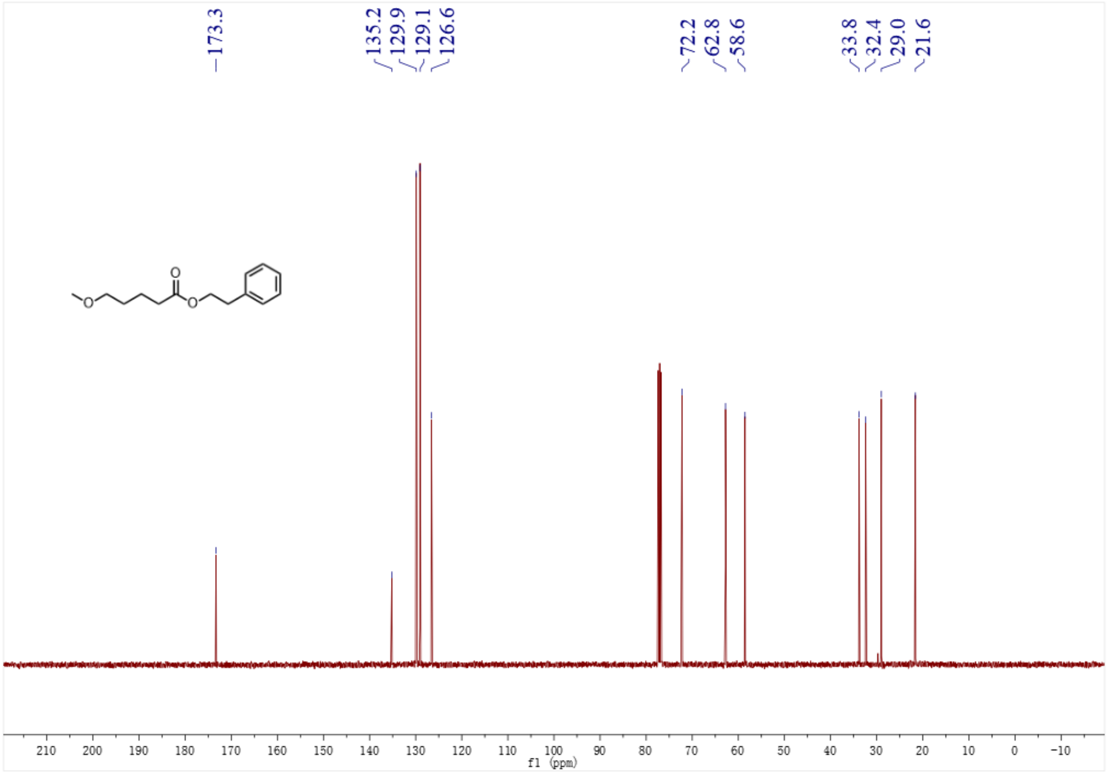

^{1}H NMR spectrum of **5** ($CDCl_3$)

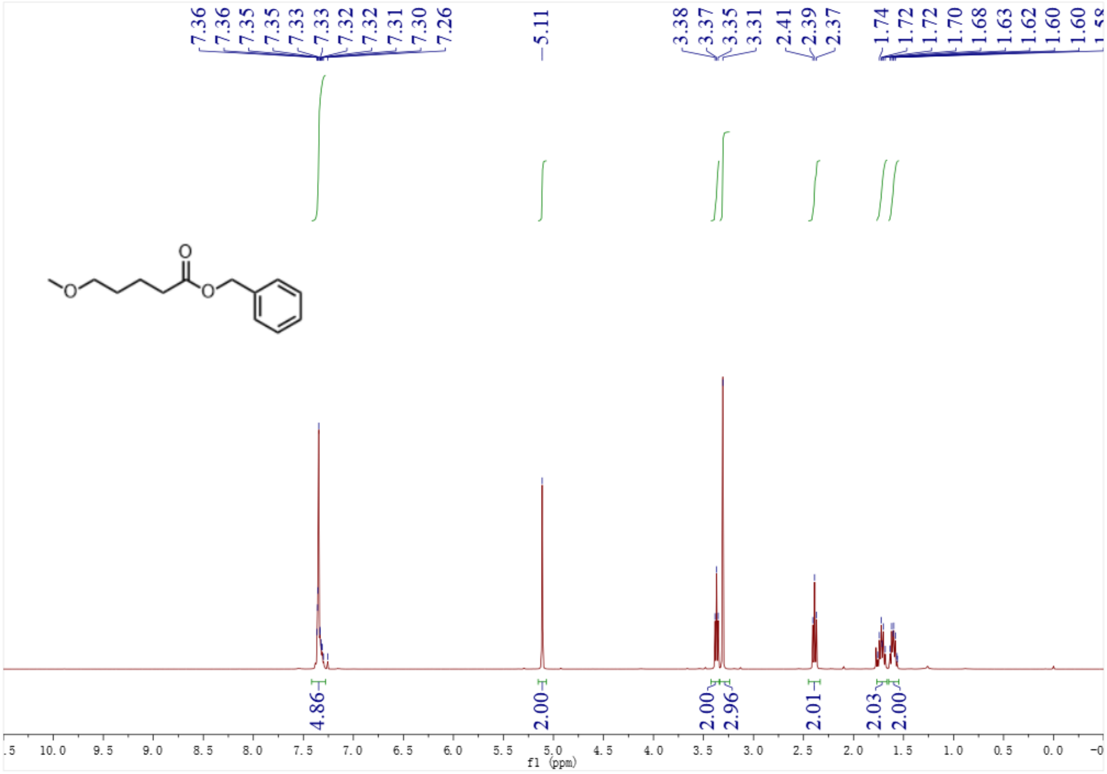

^{13}C NMR spectrum of **5** ($CDCl_3$)

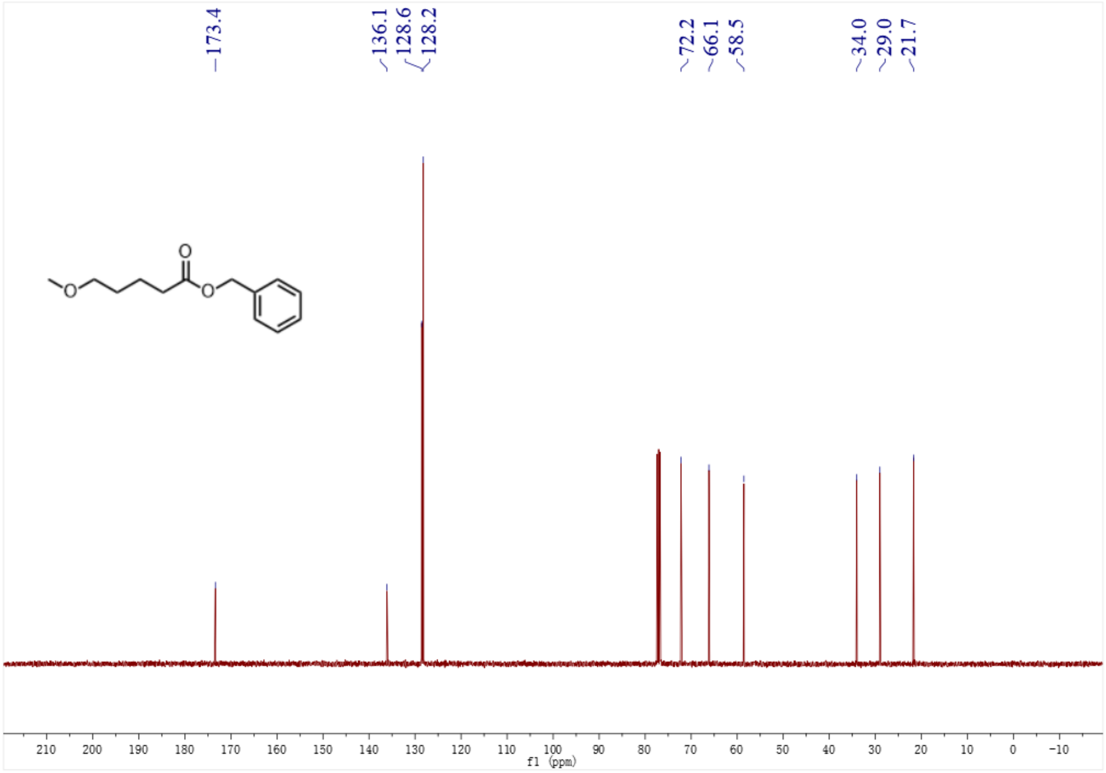

^{1}H NMR spectrum of **6** ($CDCl_3$)

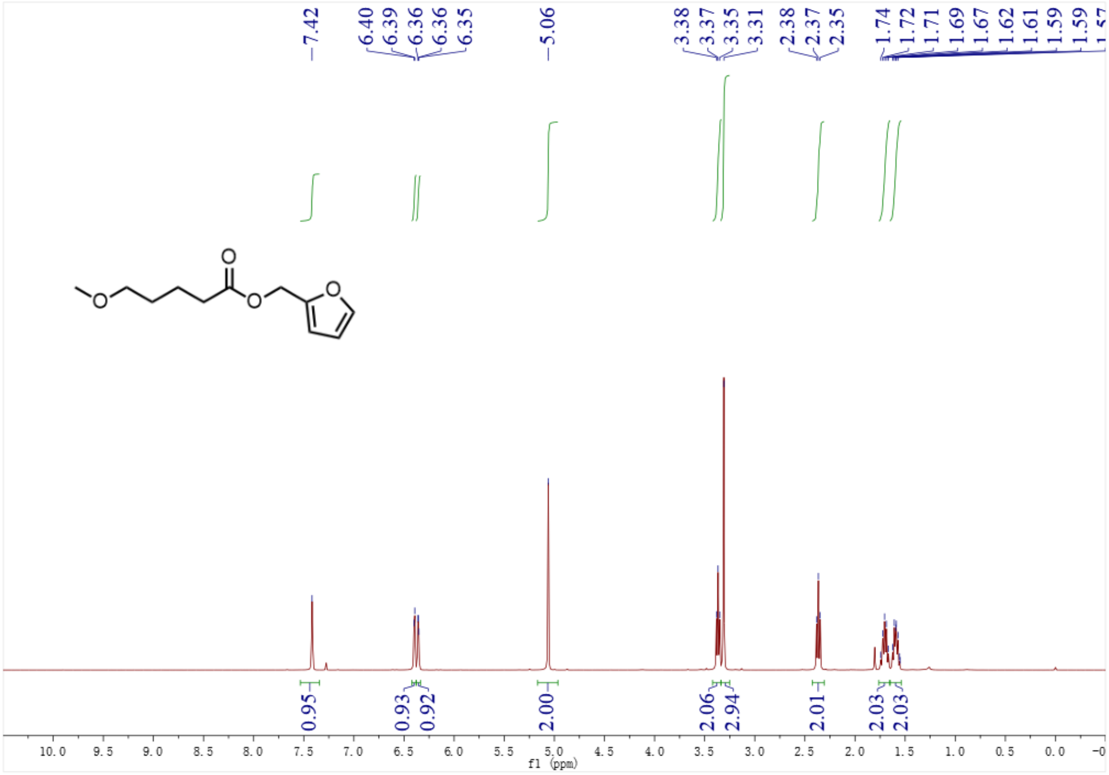

^{13}C NMR spectrum of **6** ($CDCl_3$)

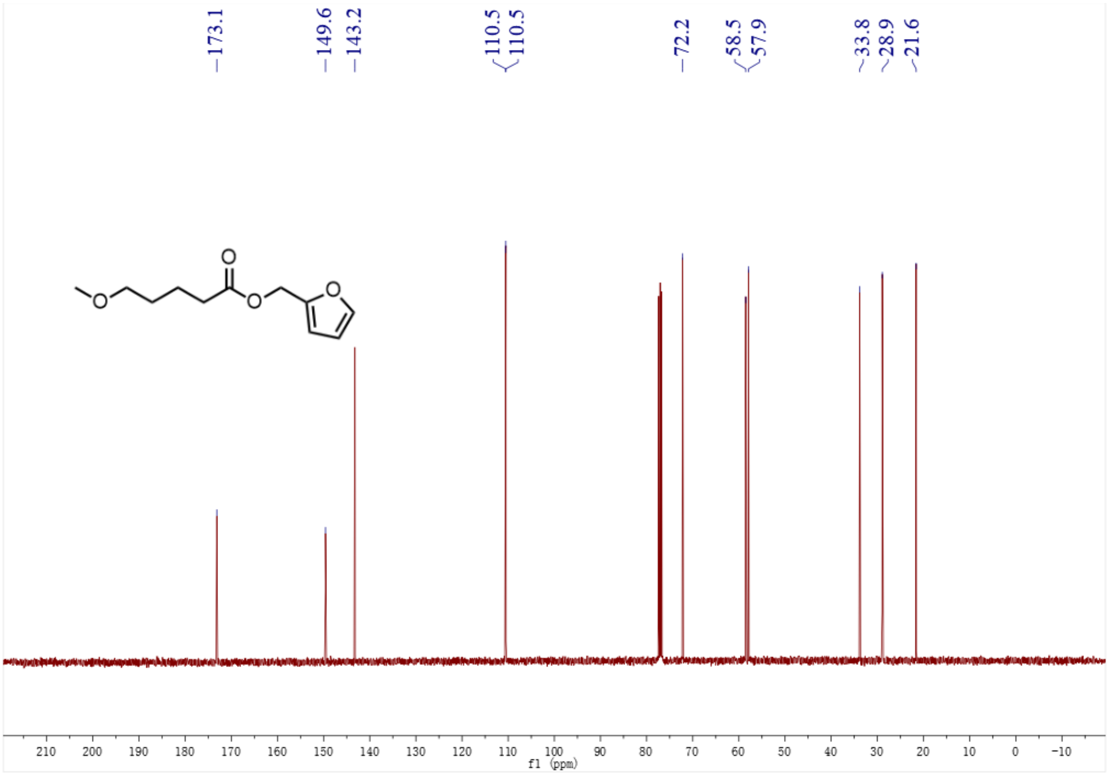

^{1}H NMR spectrum of **7** ($CDCl_3$)

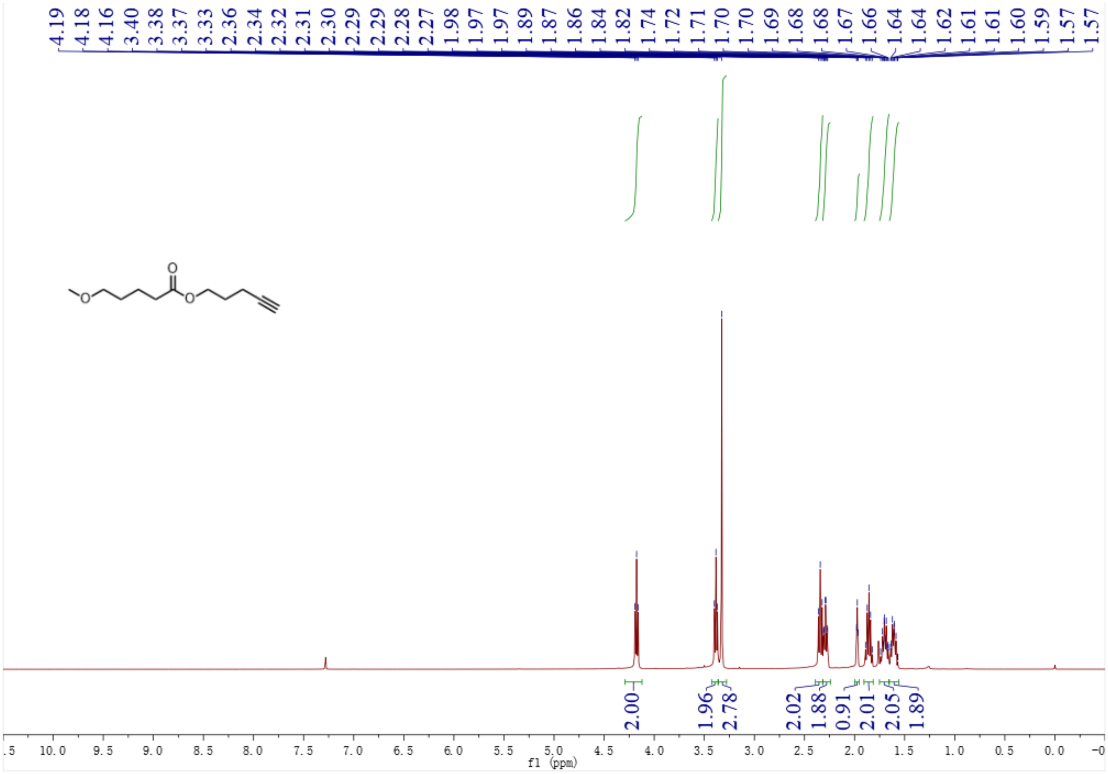

^{13}C NMR spectrum of **7** ($CDCl_3$)

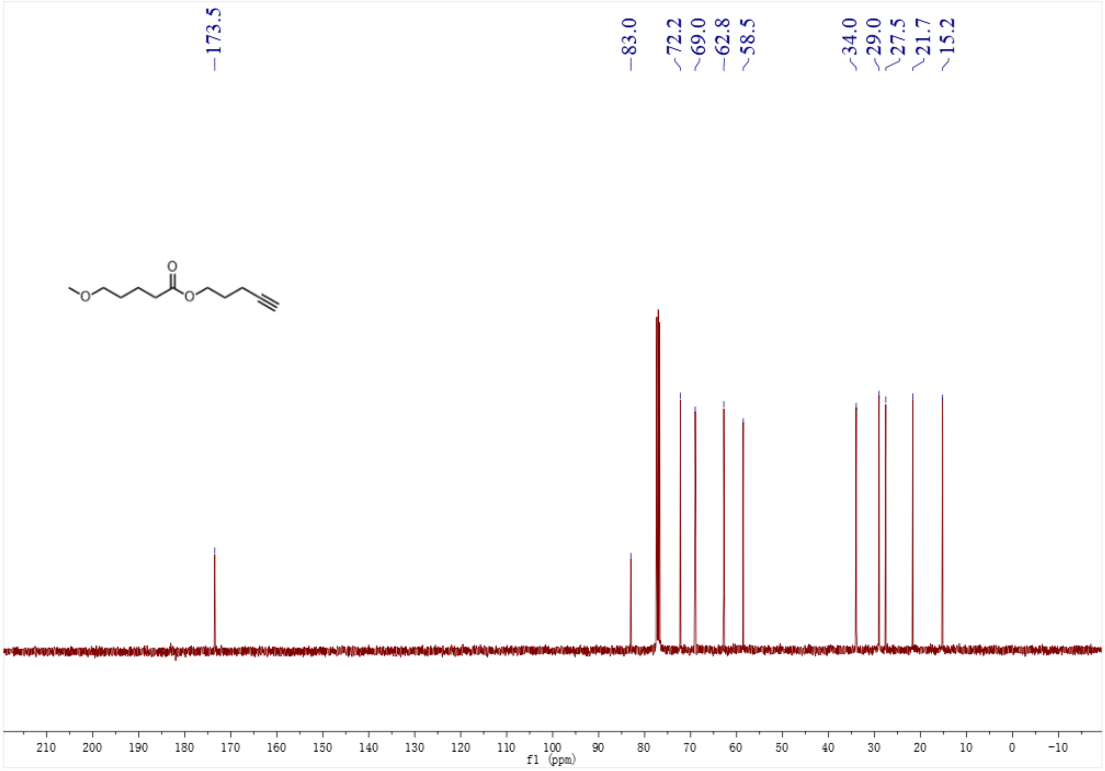

^{1}H NMR spectrum of **8** ($CDCl_3$)

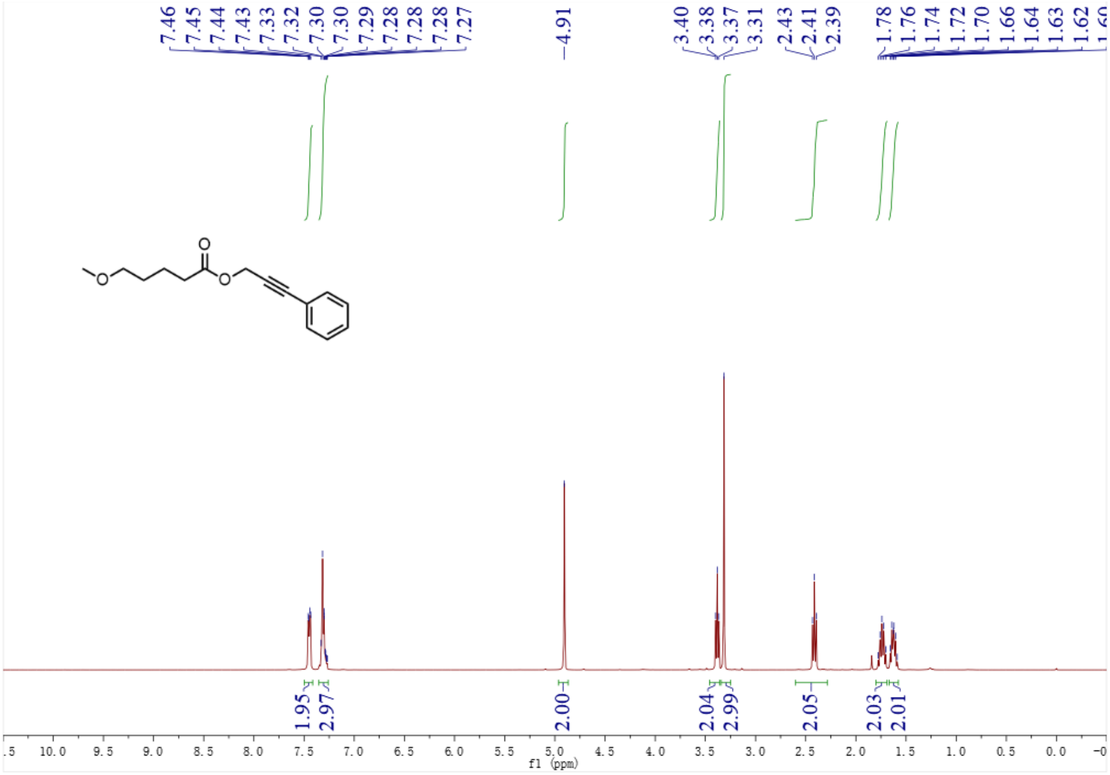

^{13}C NMR spectrum of **8** ($CDCl_3$)

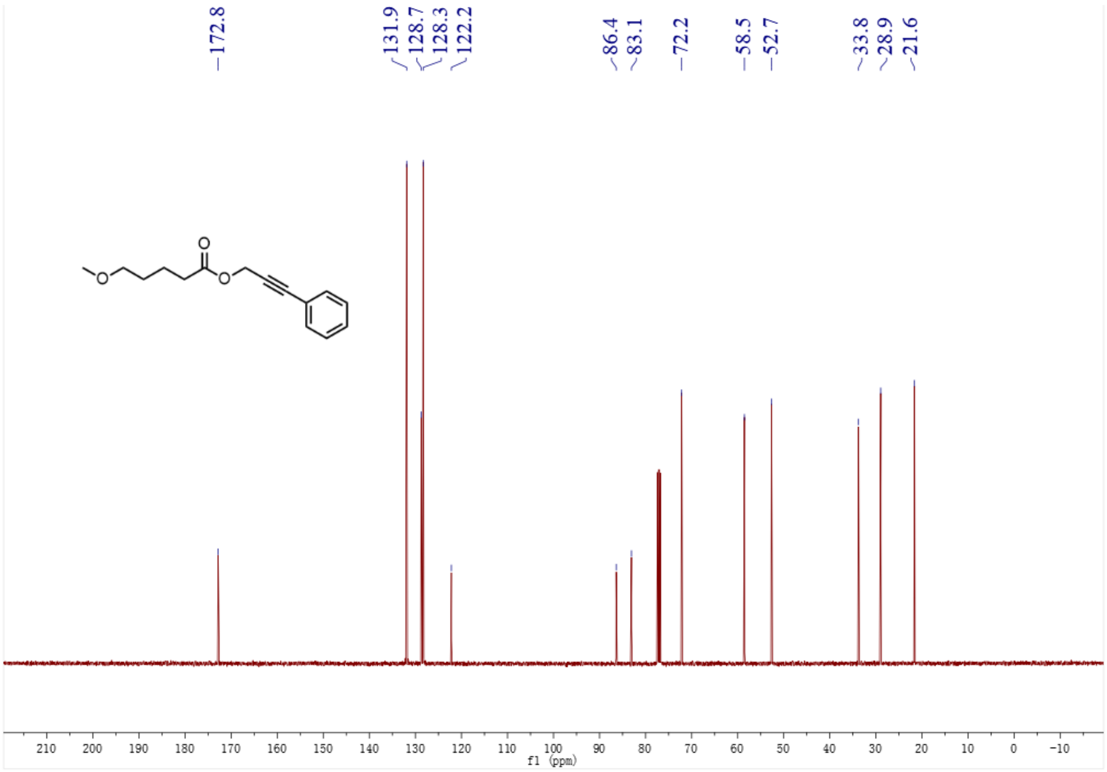

^{1}**H NMR** spectrum of **9** ($CDCl_3$)

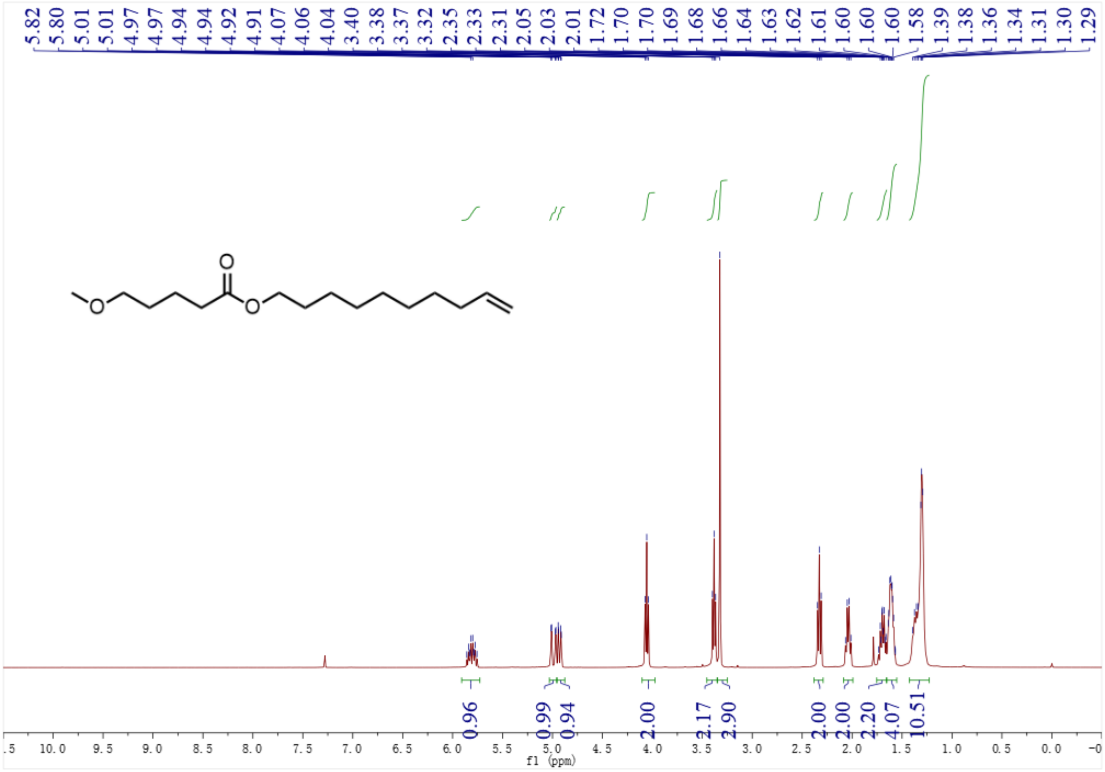

^{13}**C NMR** spectrum of **9** ($CDCl_3$)

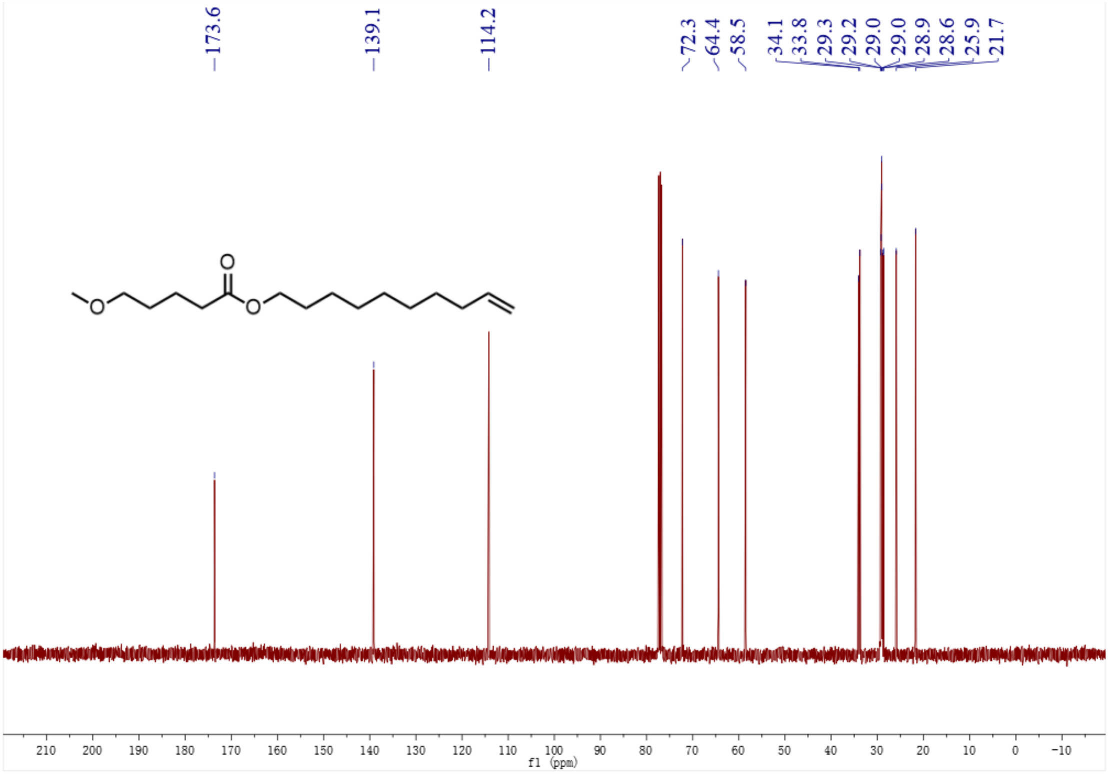

^{1}H NMR spectrum of **10** ($CDCl_3$)

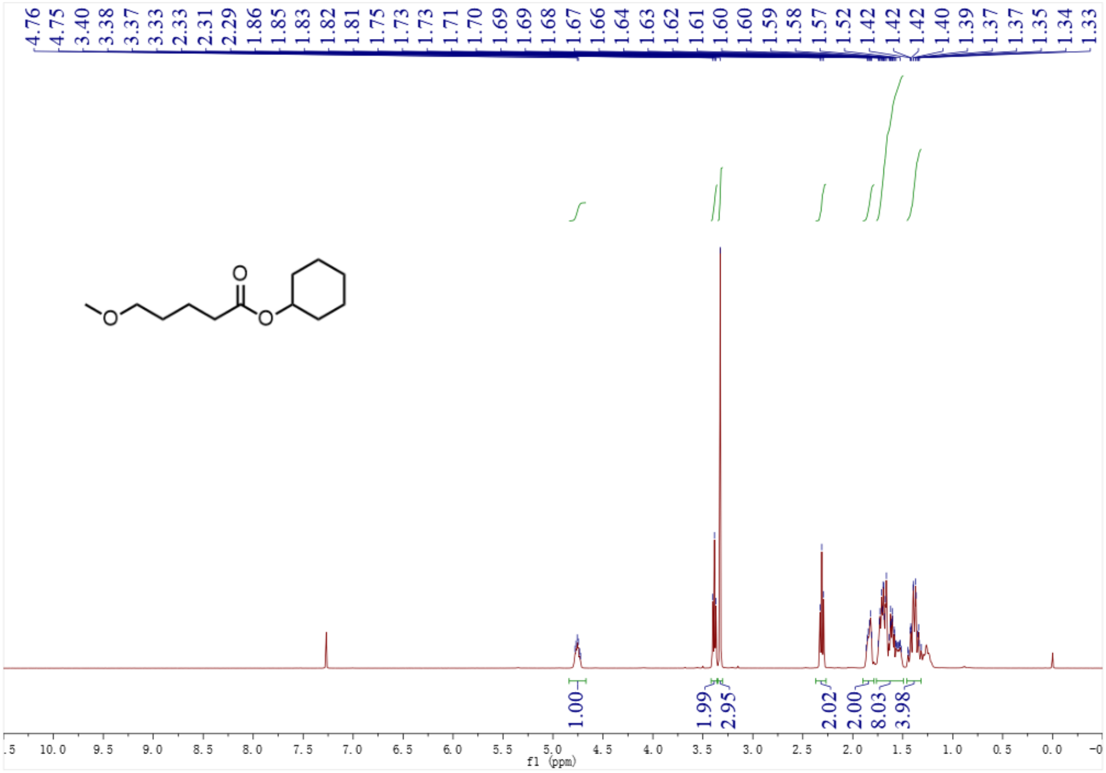

^{13}C NMR spectrum of **10** ($CDCl_3$)

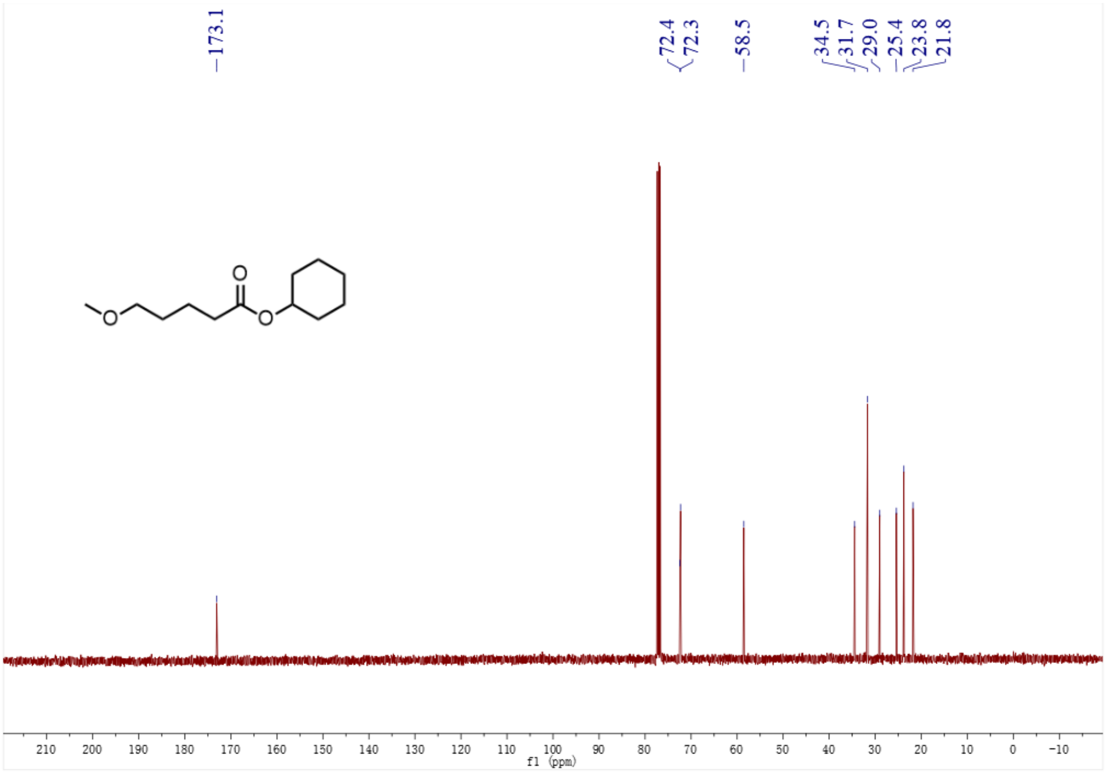

[1]**H NMR** spectrum of **11** (CDCl$_3$)

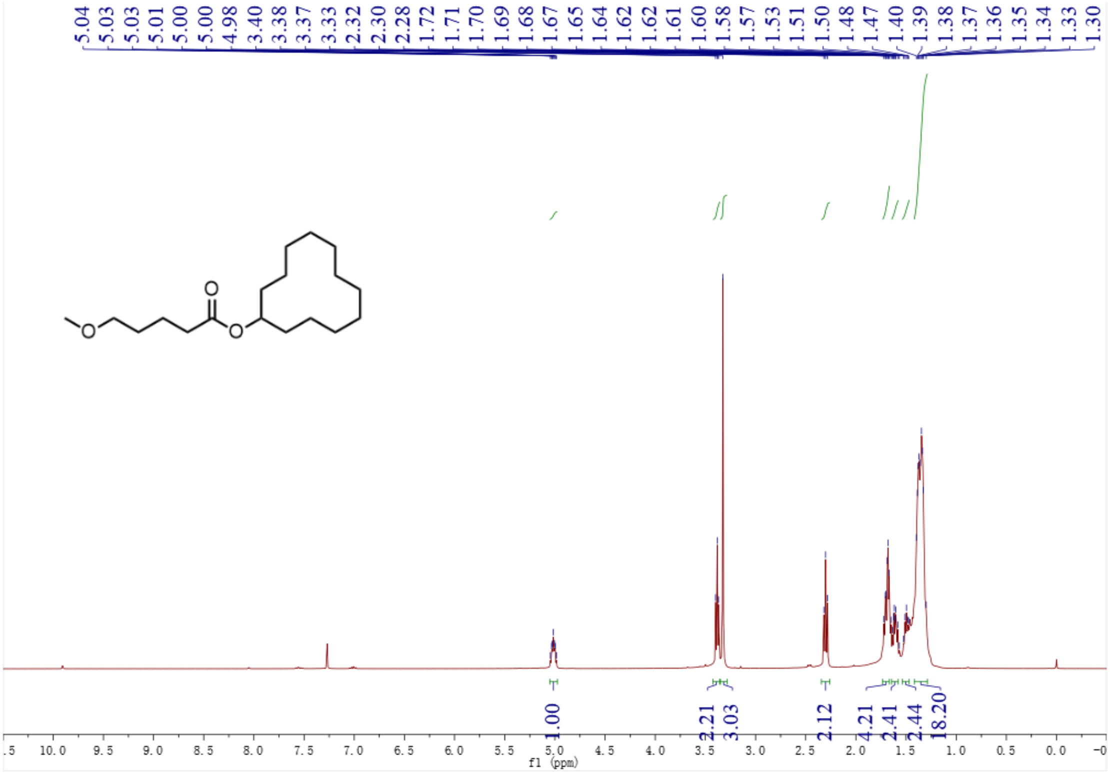

[13]**C NMR** spectrum of **11** (CDCl$_3$)

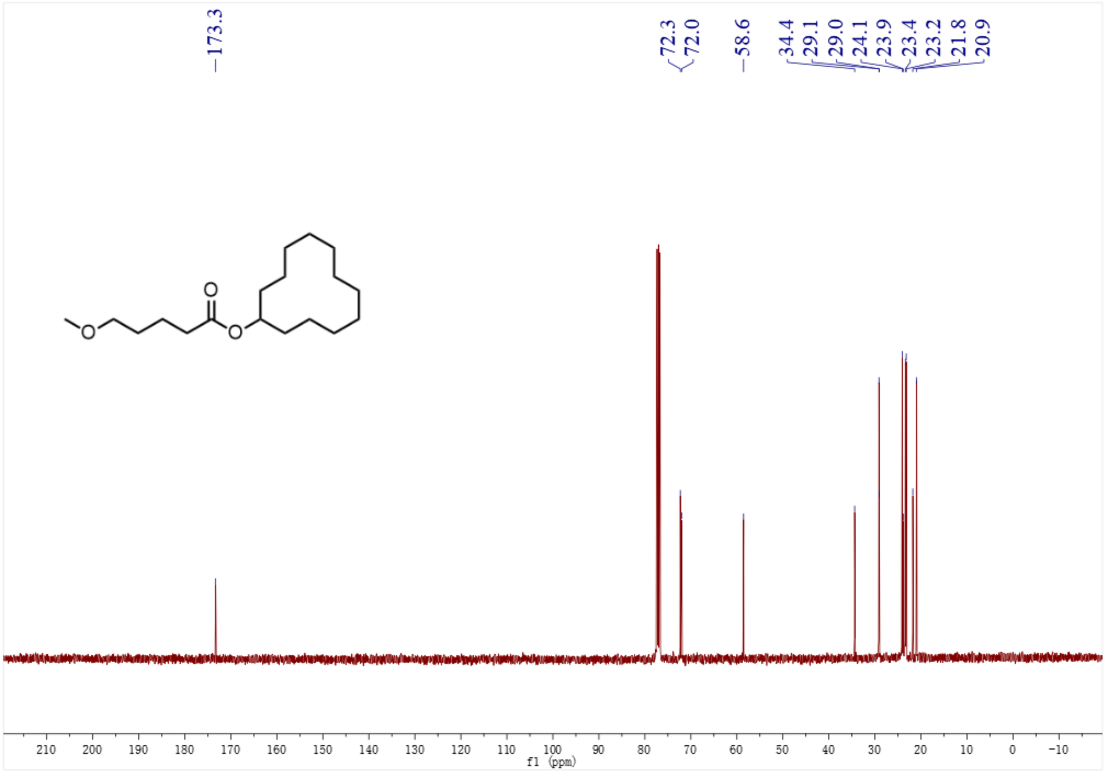

^{1}H NMR spectrum of **12** ($CDCl_3$)

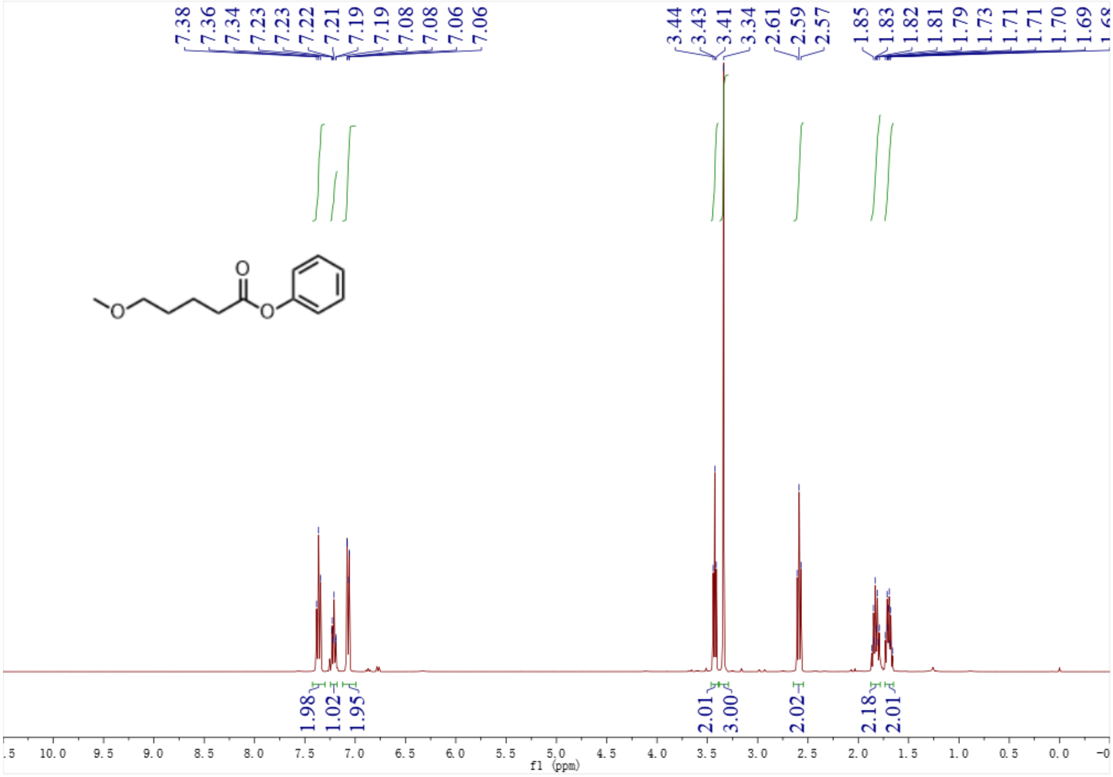

^{13}C NMR spectrum of **12** ($CDCl_3$)

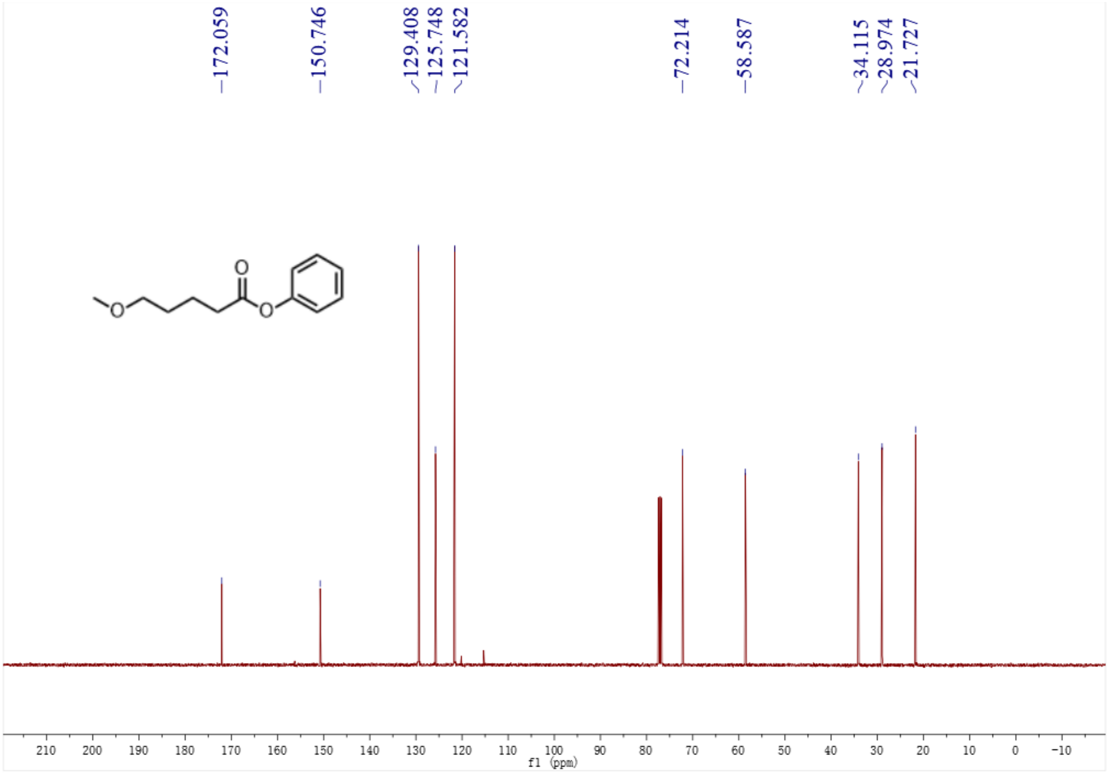

[1]**H NMR** spectrum of **13** ($CDCl_3$)

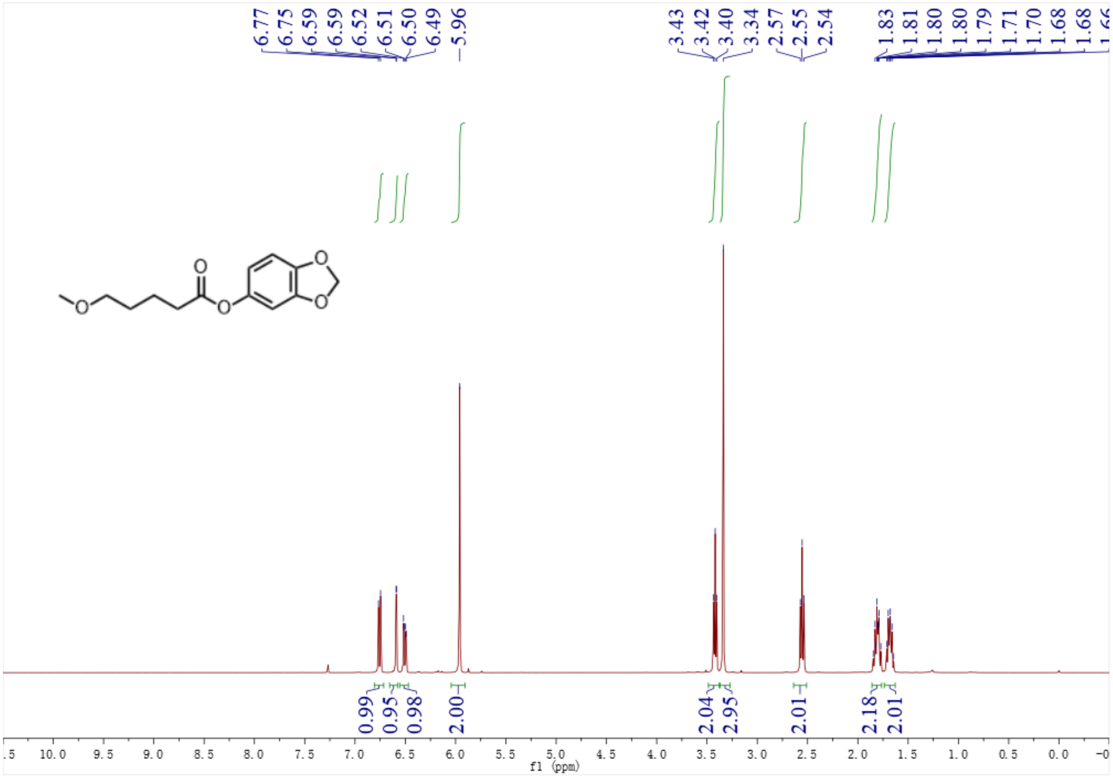

[13]**C NMR** spectrum of **13** ($CDCl_3$)

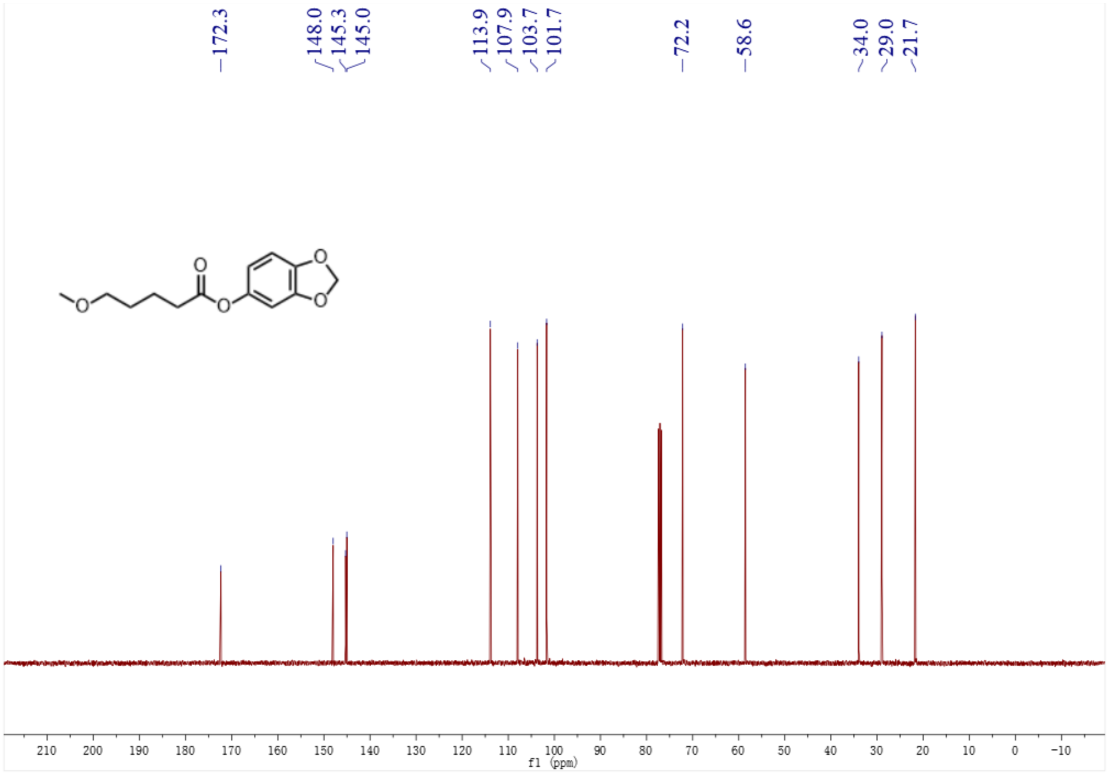

^{1}H **NMR** spectrum of **14** ($CDCl_3$)

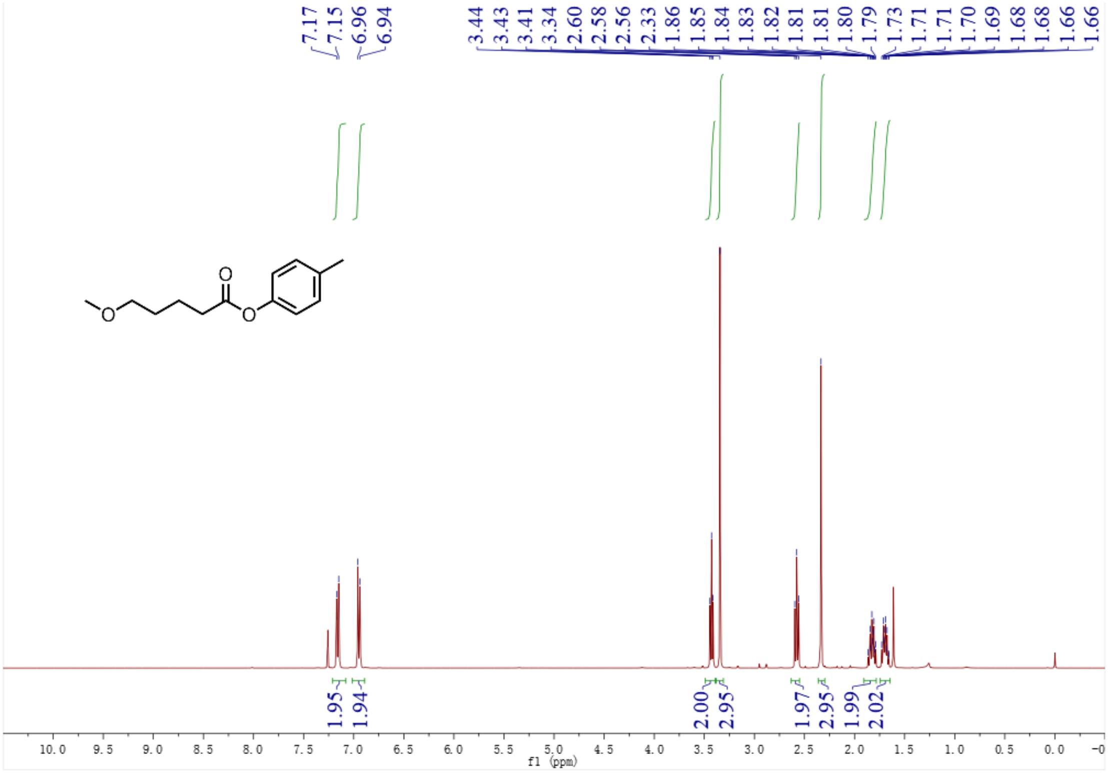

^{13}C **NMR** spectrum of **14** ($CDCl_3$)

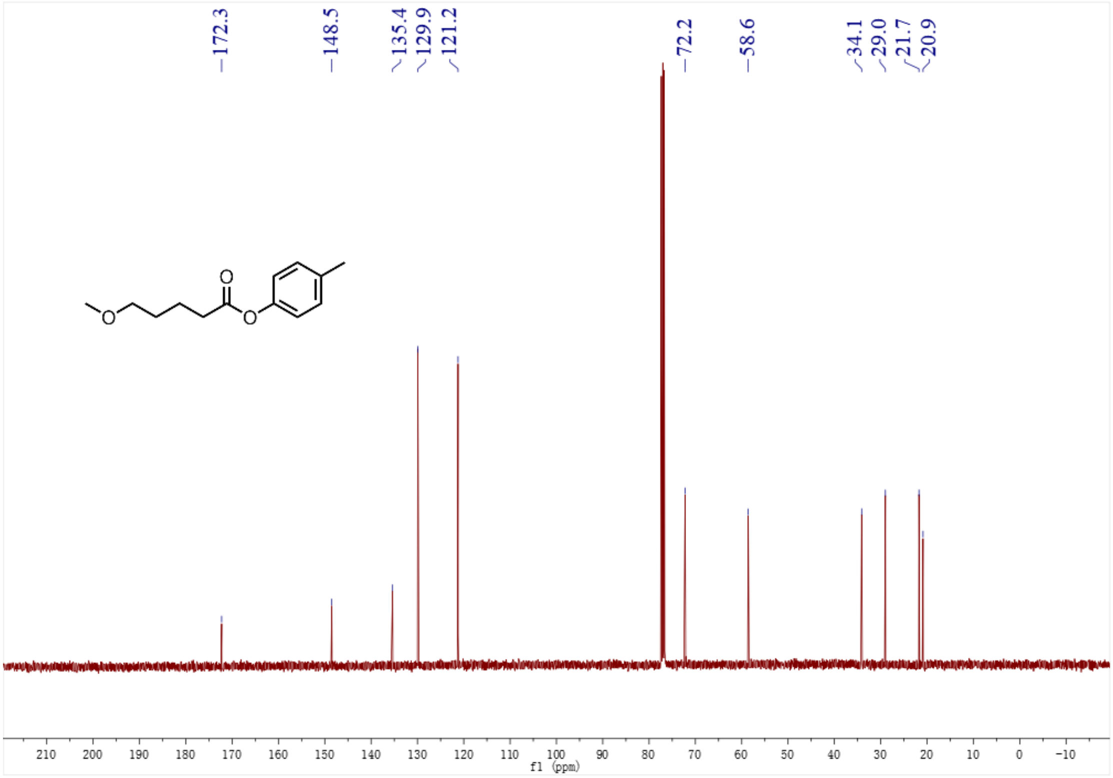

[1]**H NMR** spectrum of **15** ($CDCl_3$)

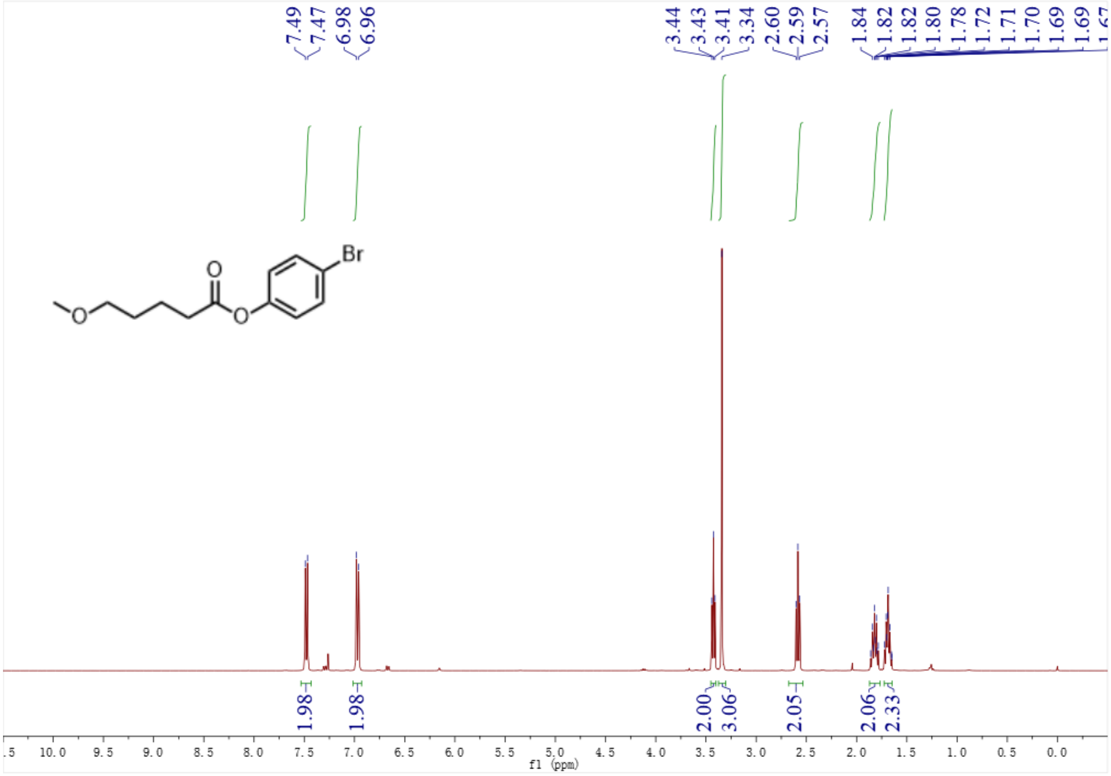

[13]**C NMR** spectrum of **15** ($CDCl_3$)

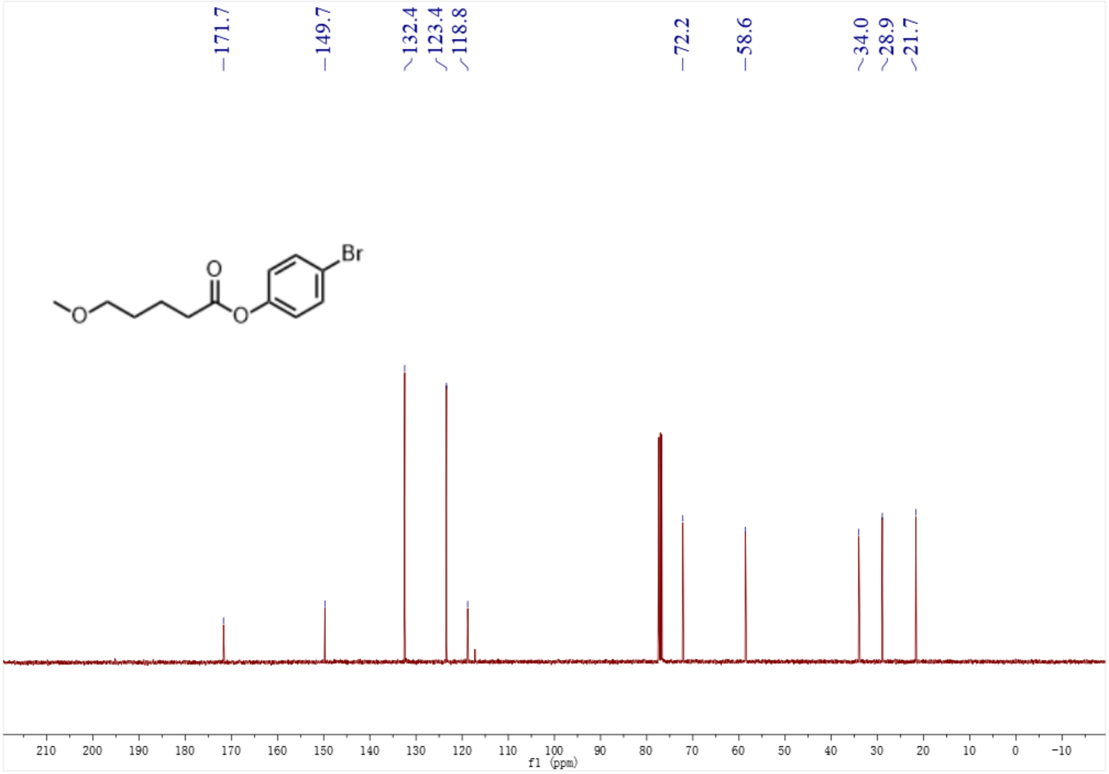

[1]**H NMR** spectrum of **16** ($CDCl_3$)

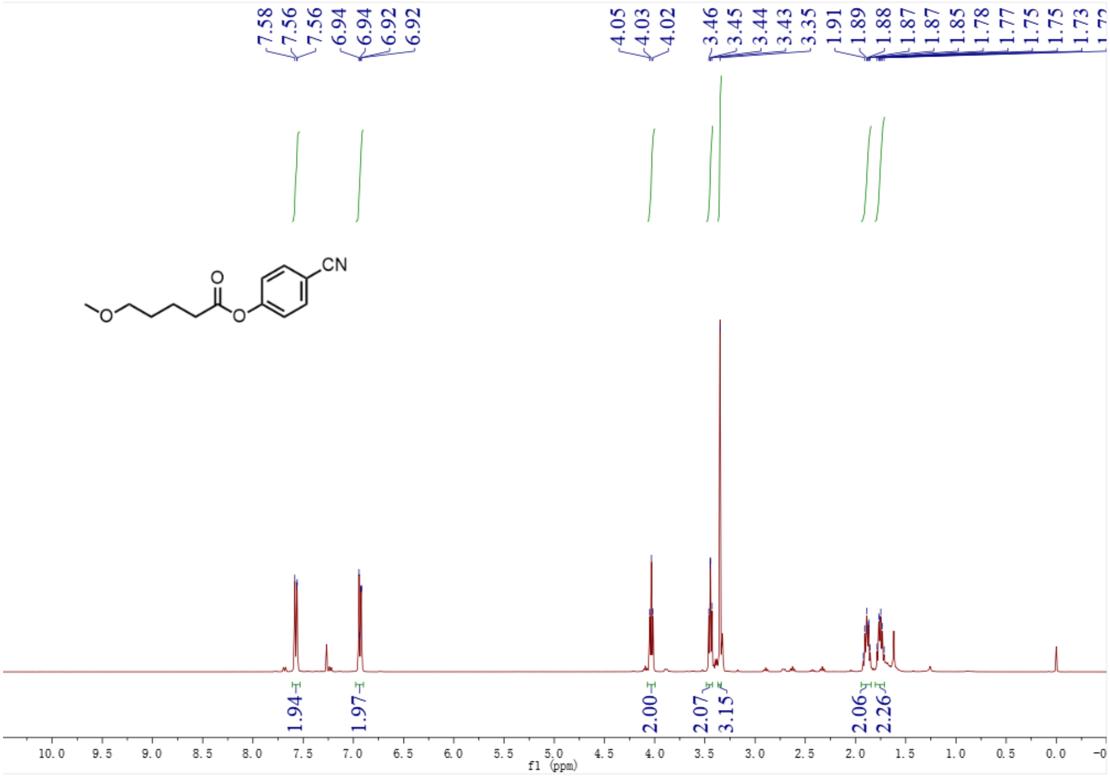

[13]**C NMR** spectrum of **16** ($CDCl_3$)

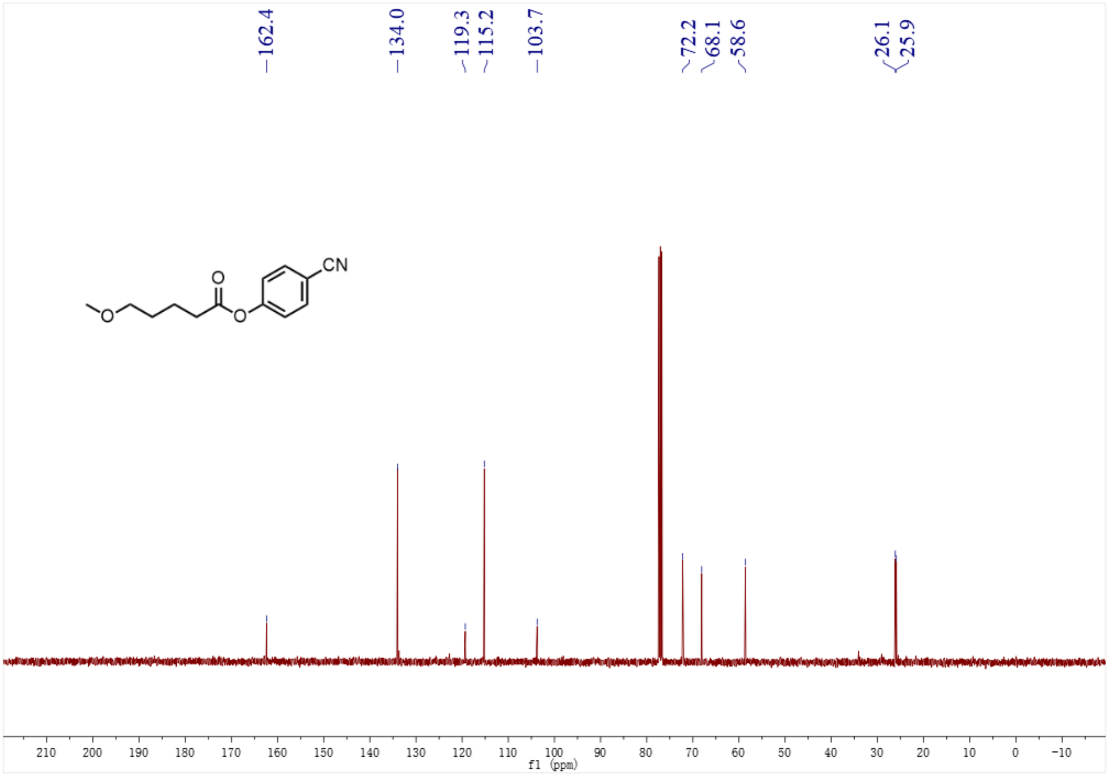

^{1}**H NMR** spectrum of **17** ($CDCl_3$)

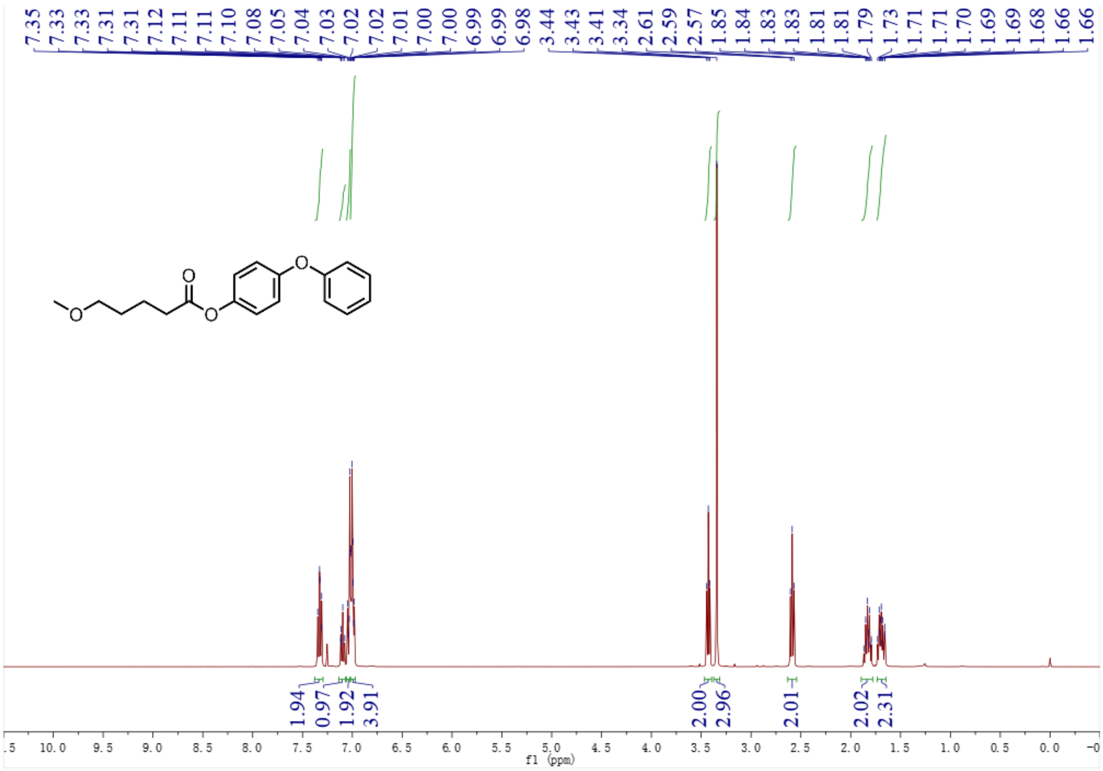

^{13}**C NMR** spectrum of **17** ($CDCl_3$)

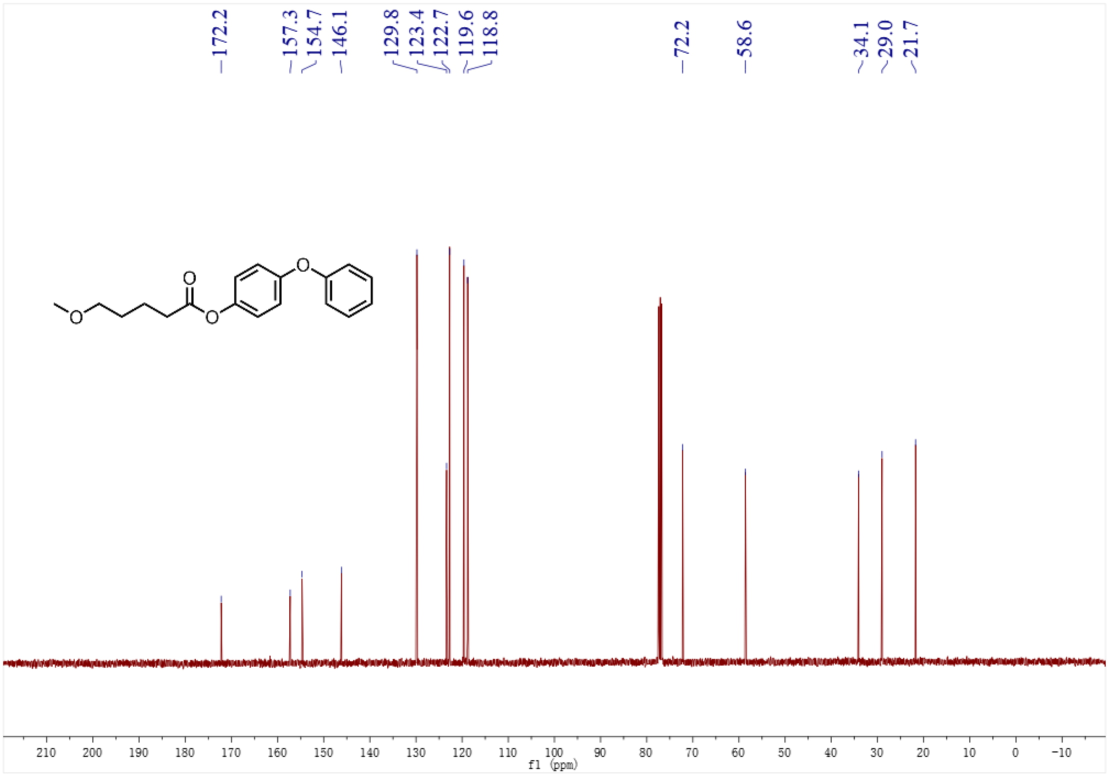

^{1}**H NMR** spectrum of **18** ($CDCl_3$)

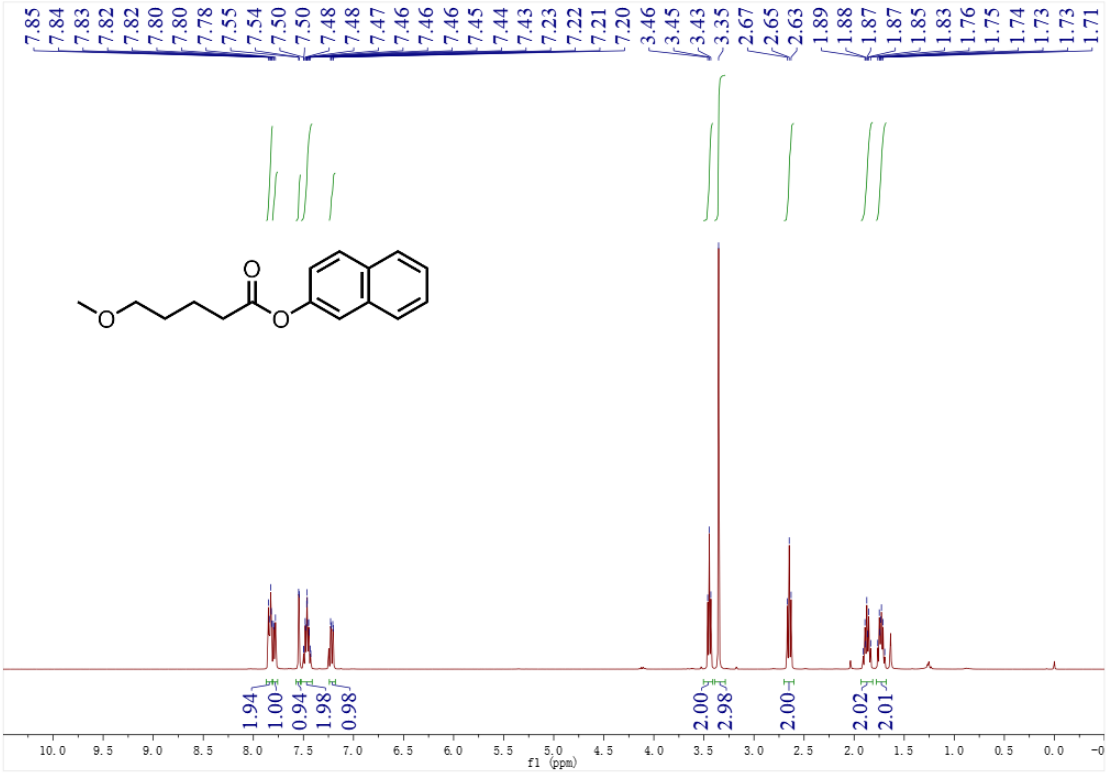

^{13}**C NMR** spectrum of **18** ($CDCl_3$)

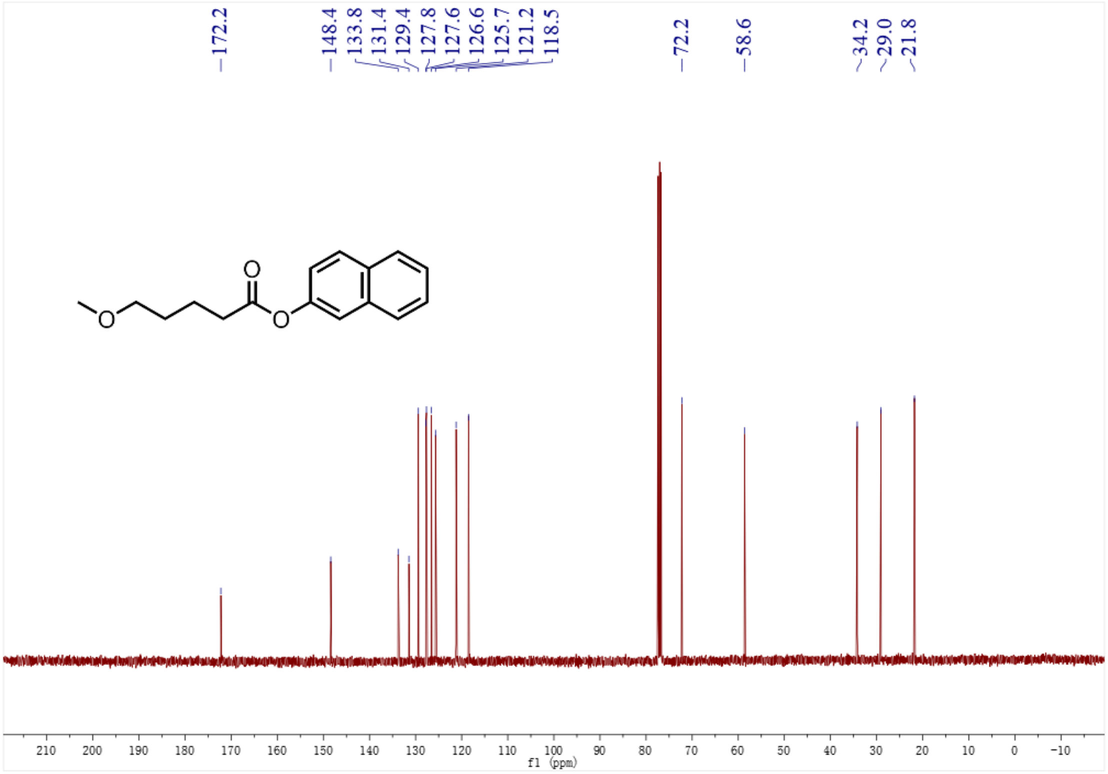

^{1}H NMR spectrum of **19** ($CDCl_3$)

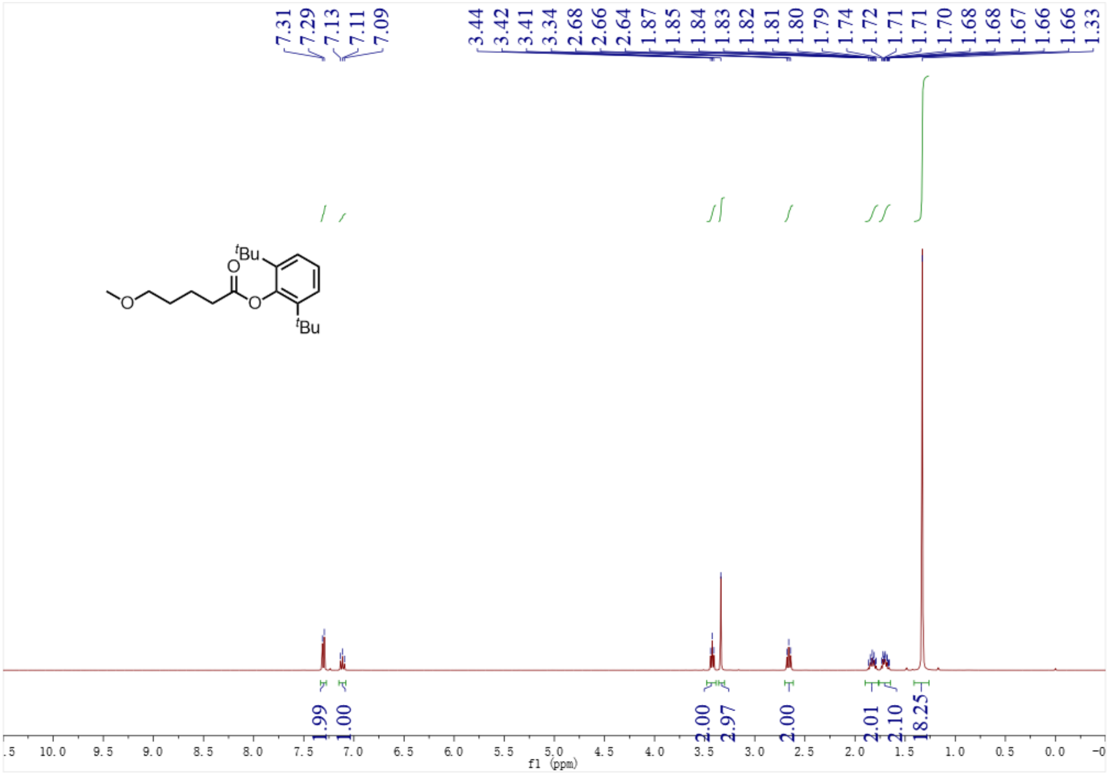

^{13}C NMR spectrum of **19** ($CDCl_3$)

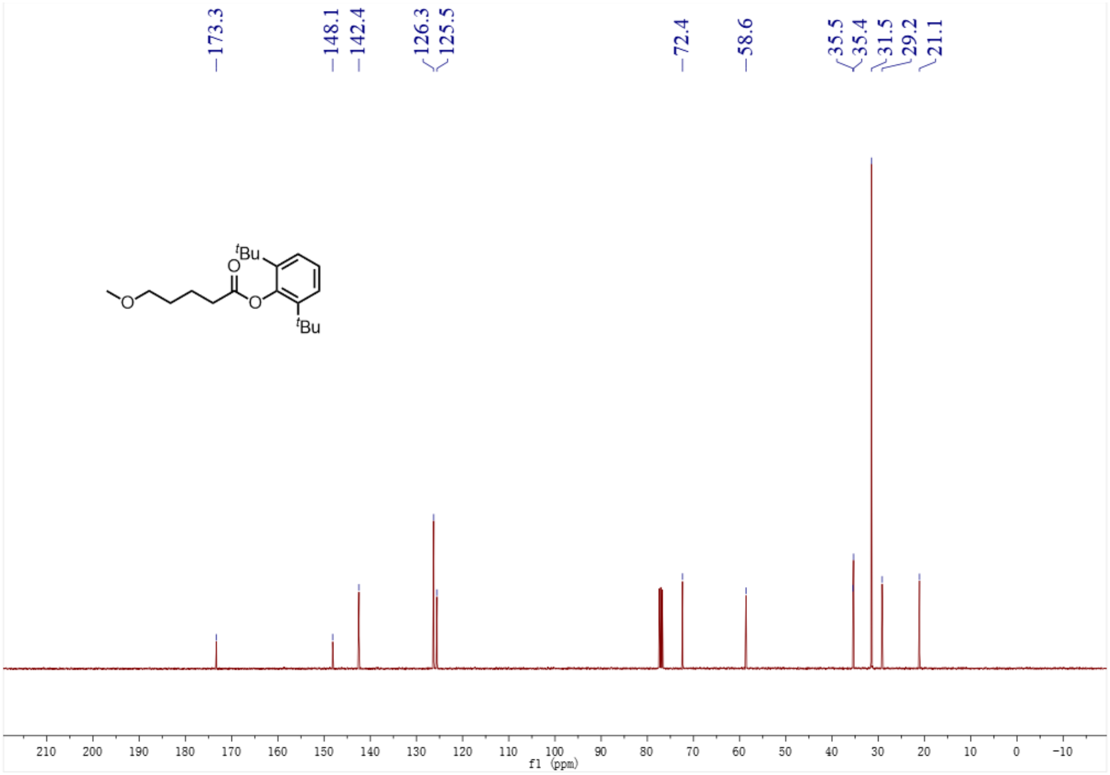

^{1}H NMR spectrum of **20** ($CDCl_3$)

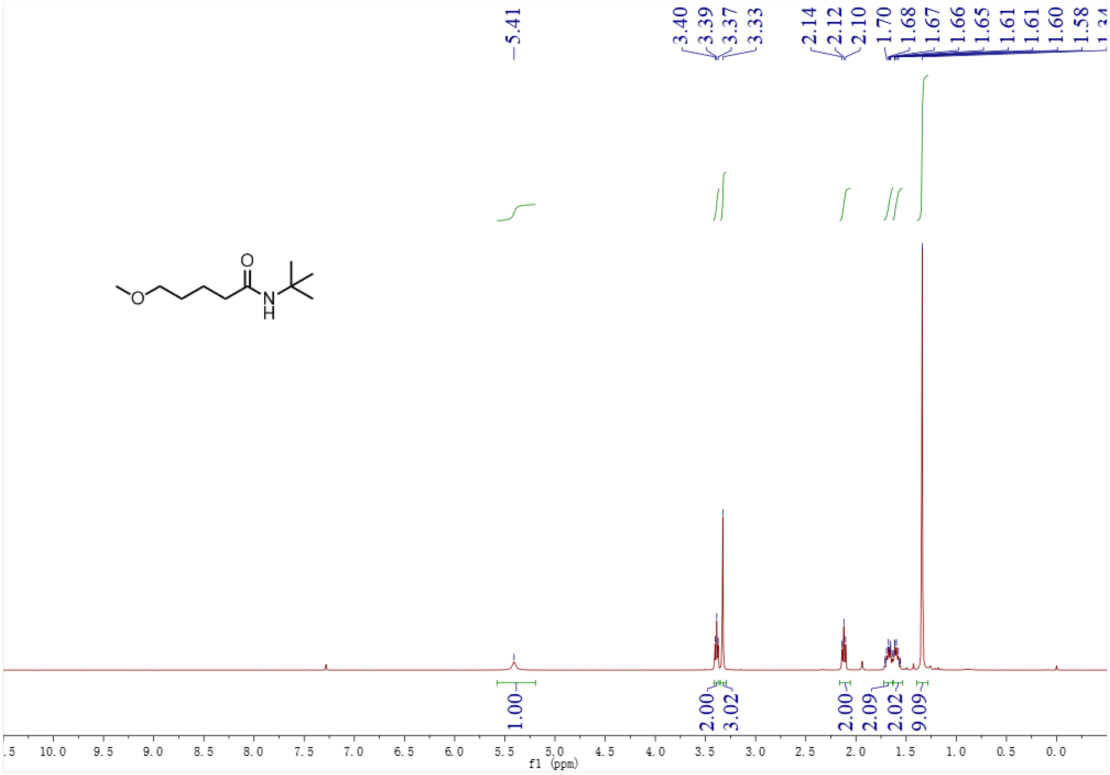

^{13}C NMR spectrum of **20** ($CDCl_3$)

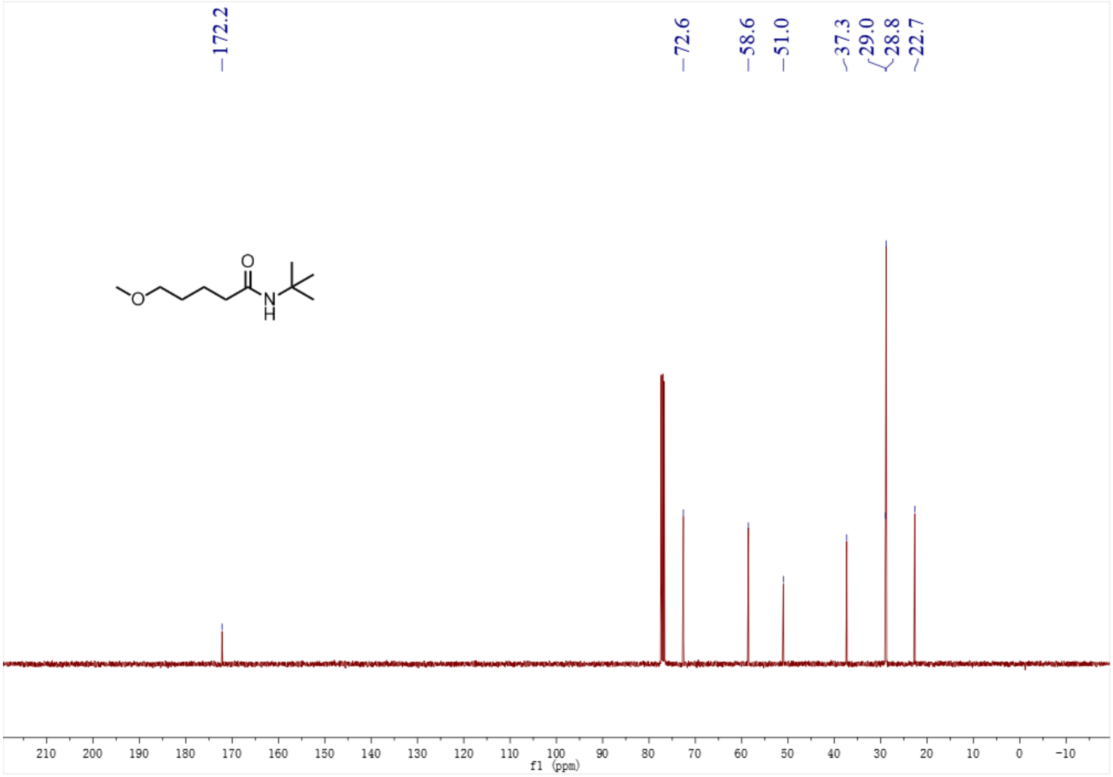

^{1}H NMR spectrum of **21** ($CDCl_3$)

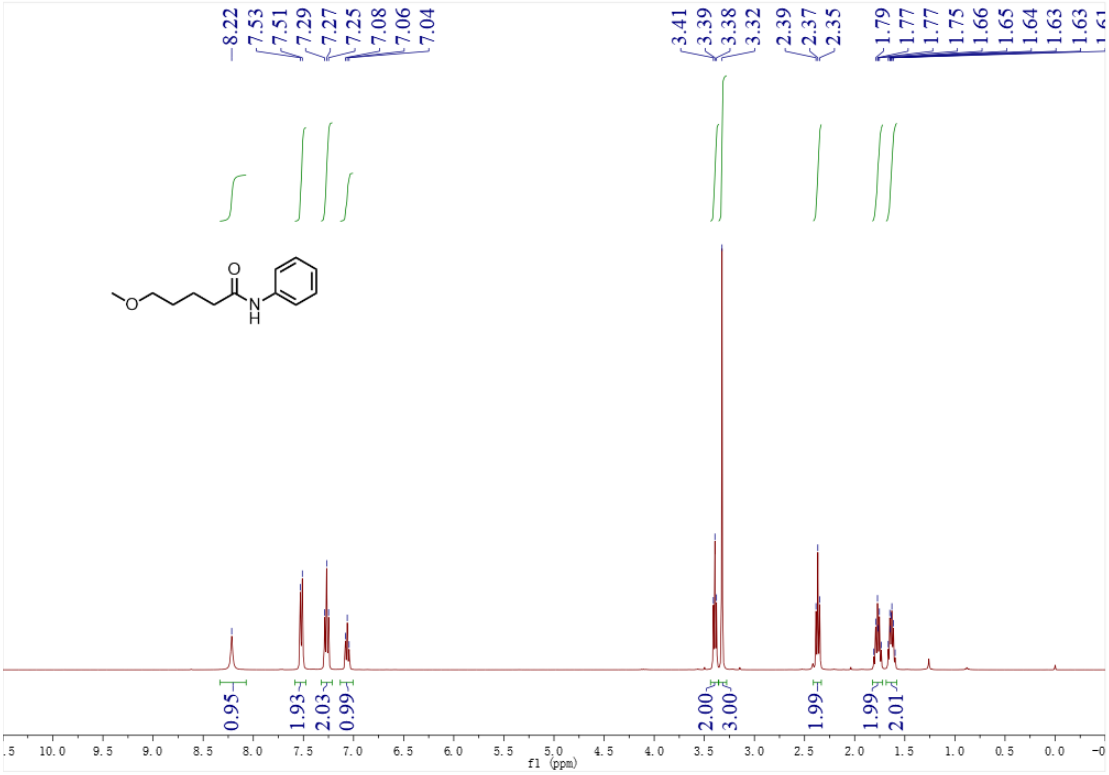

^{13}C NMR spectrum of **21** ($CDCl_3$)

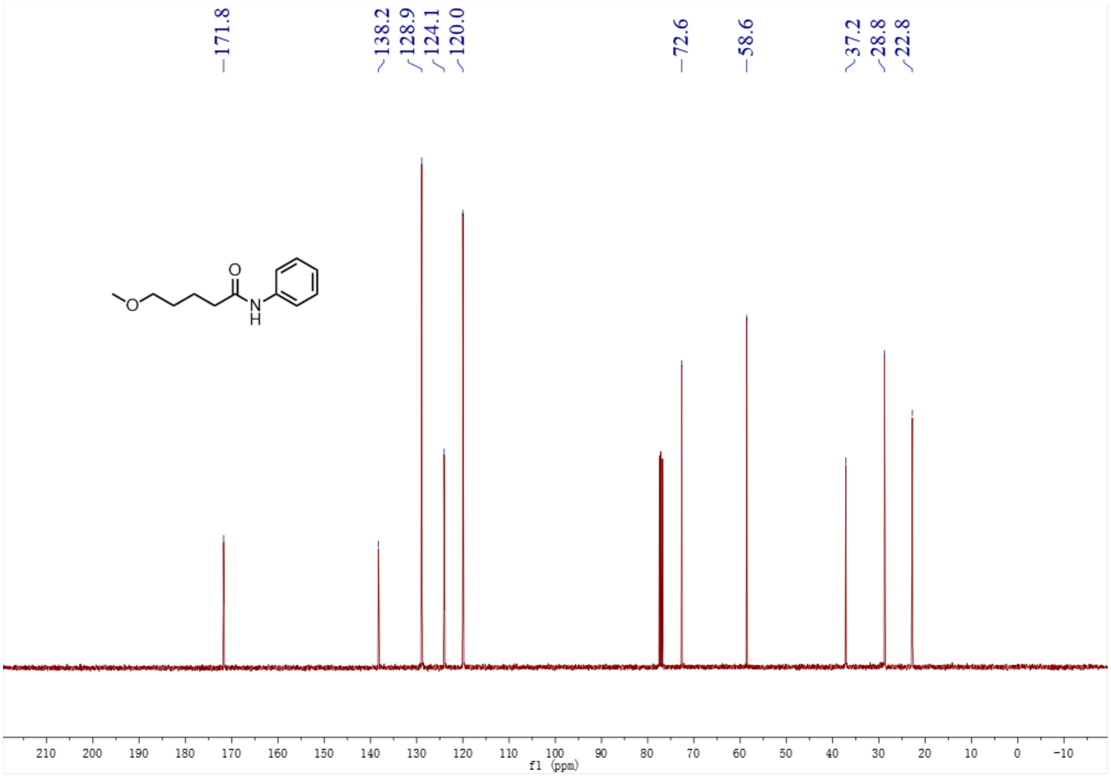

^{1}H NMR spectrum of **22** ($CDCl_3$)

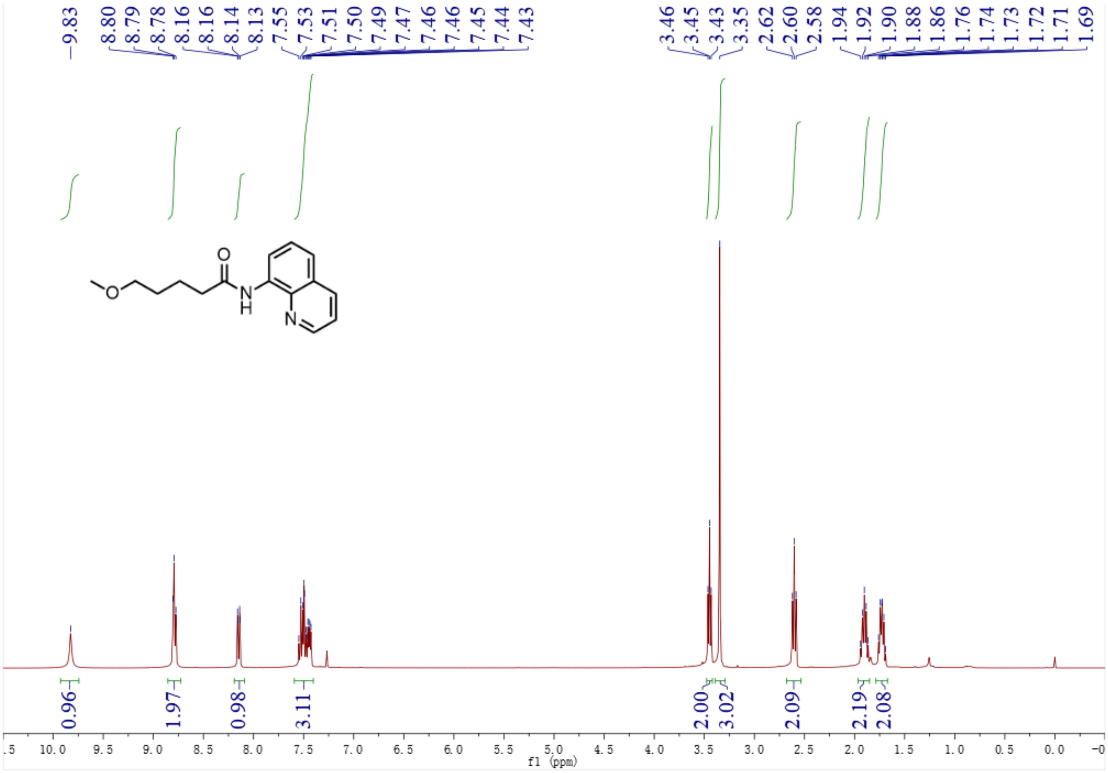

^{13}C NMR spectrum of **22** ($CDCl_3$)

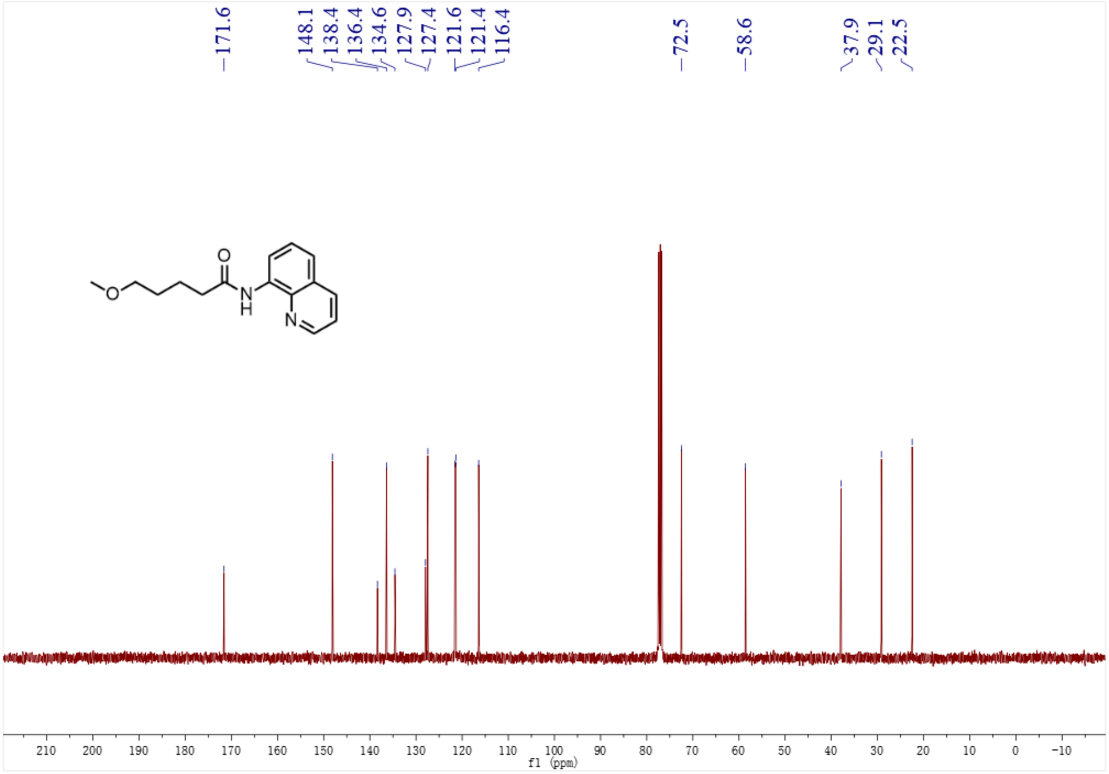

^{1}**H NMR** spectrum of **23** ($CDCl_3$)

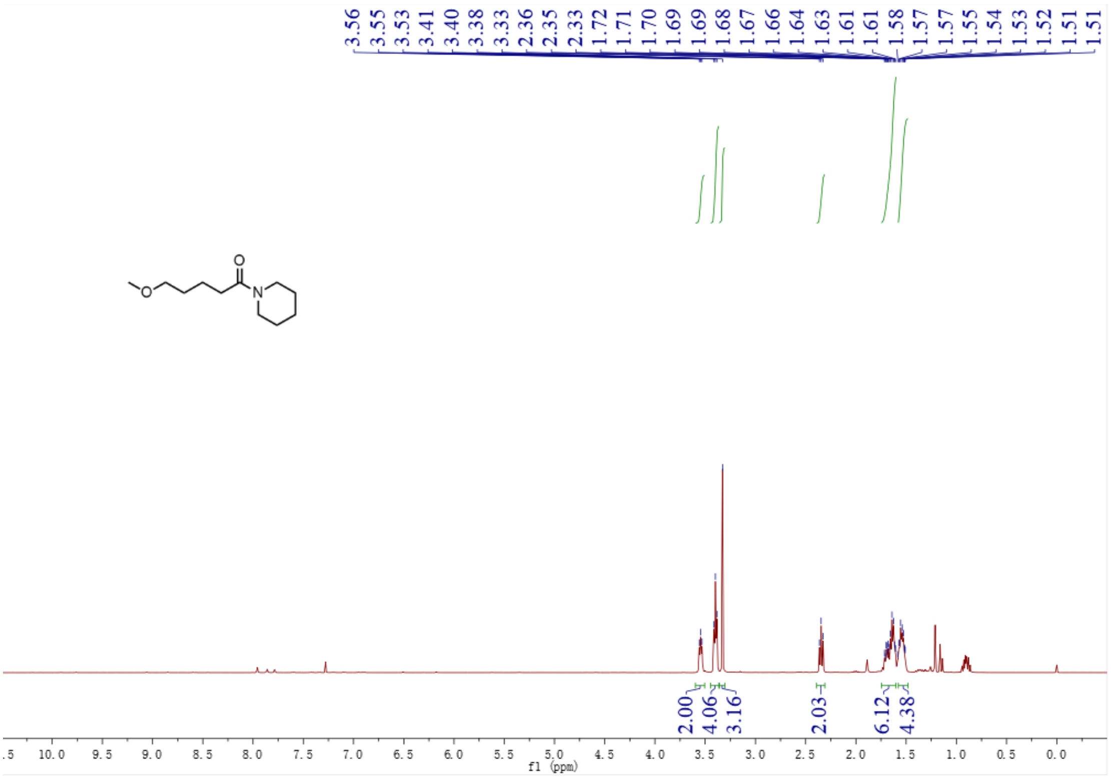

^{13}**C NMR** spectrum of **23** ($CDCl_3$)

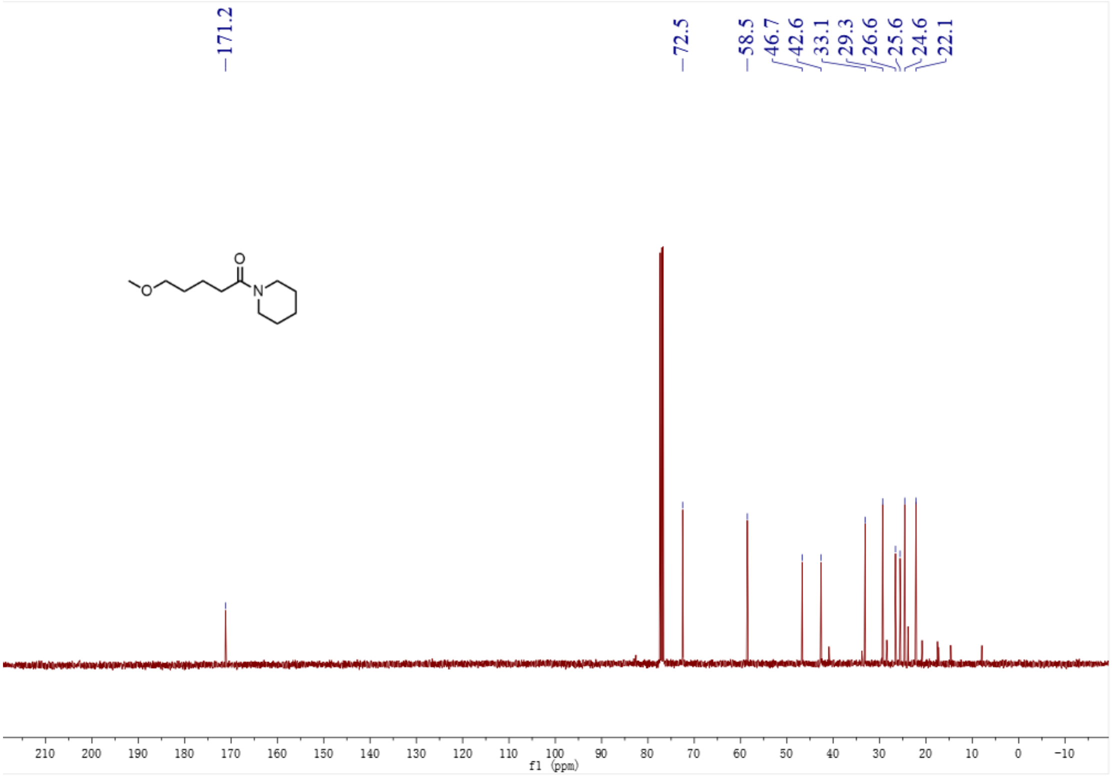

^{1}H NMR spectrum of **24** ($CDCl_3$)

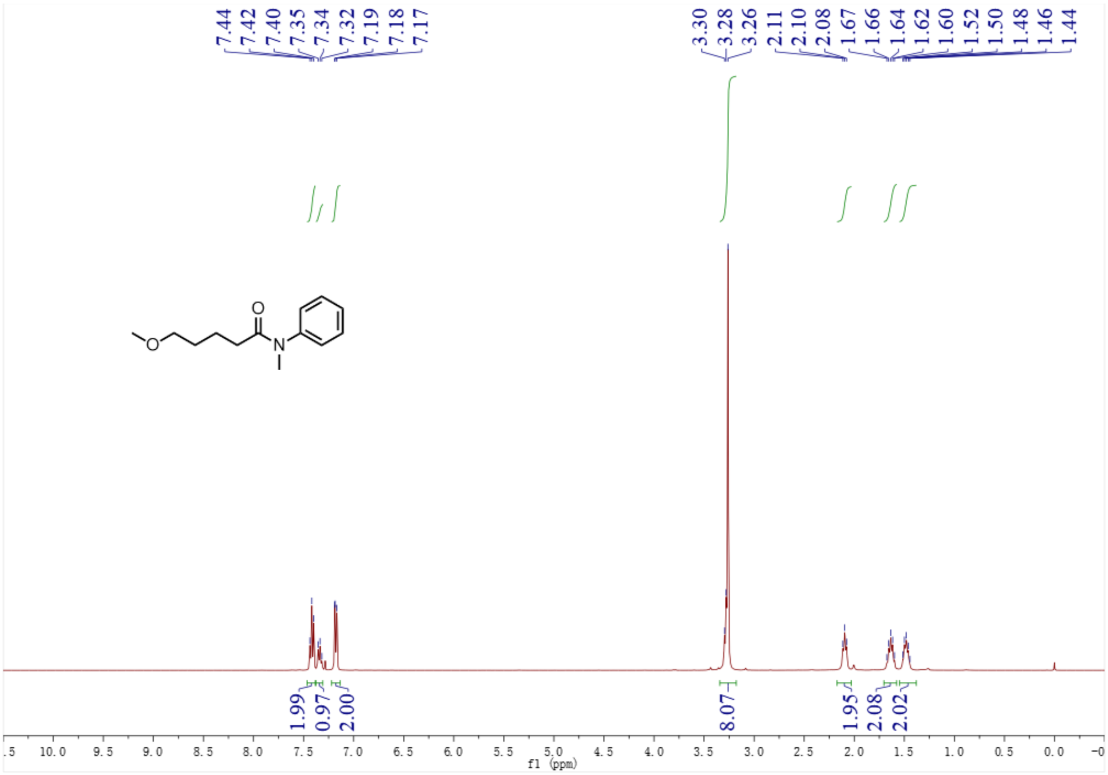

^{13}C NMR spectrum of **24** ($CDCl_3$)

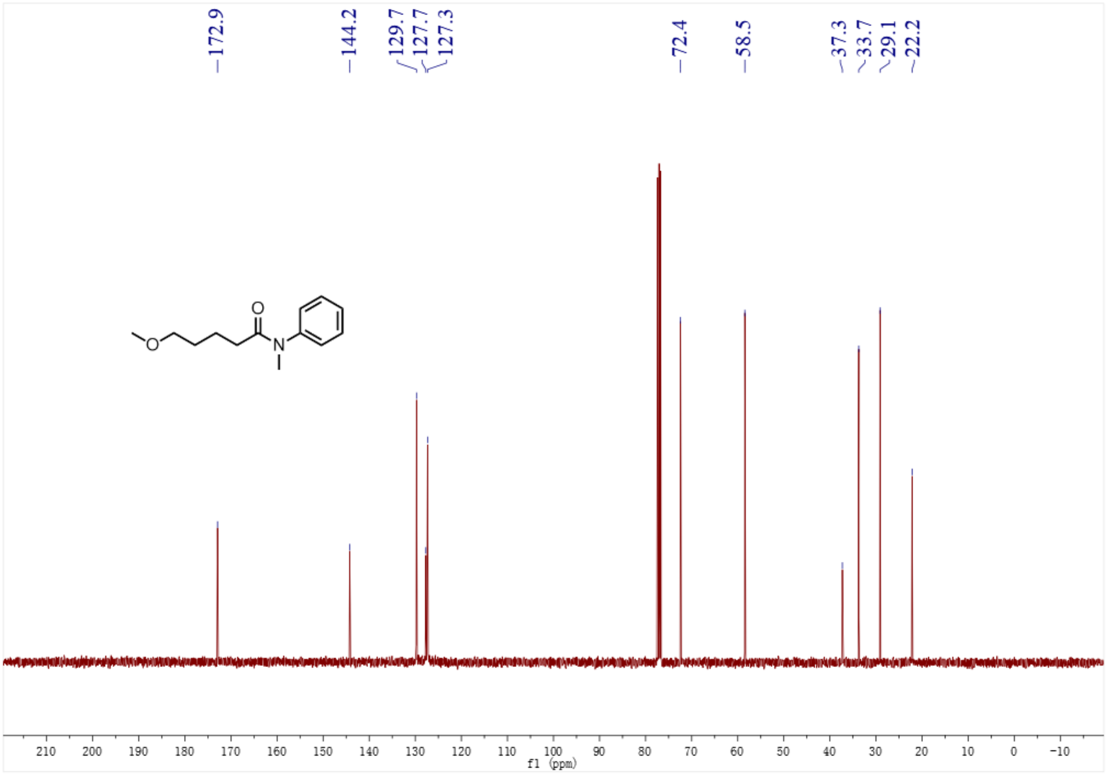

^{1}H NMR spectrum of **25** ($CDCl_3$)

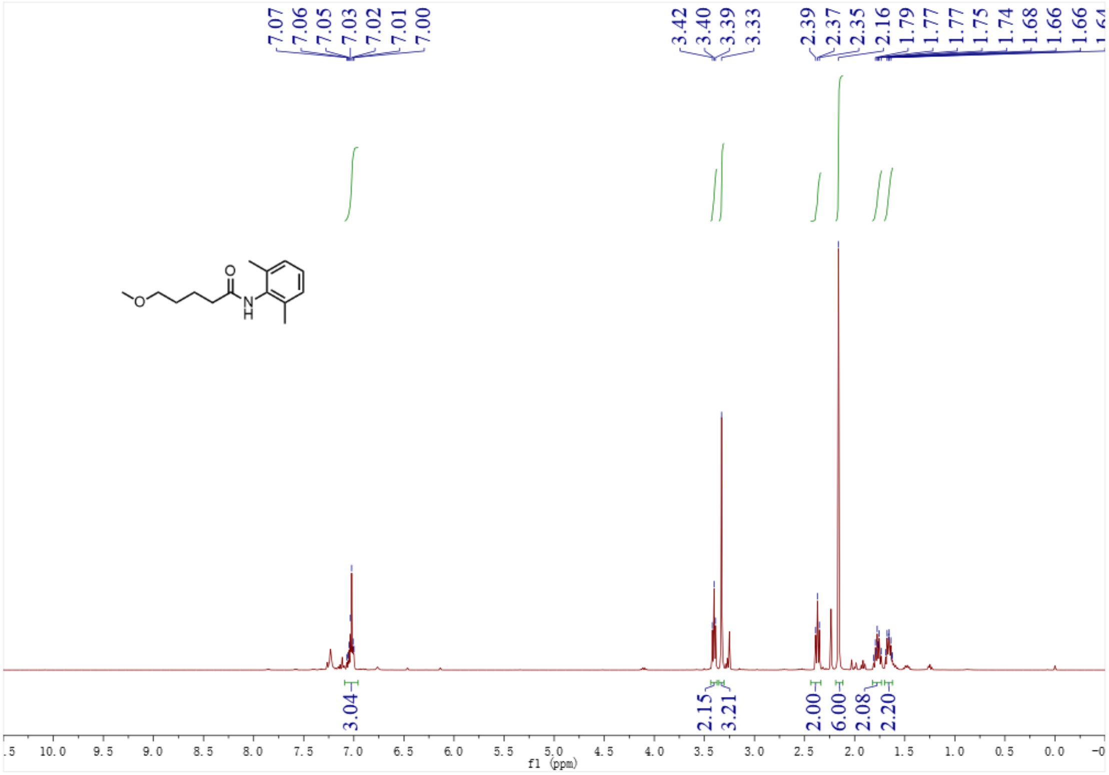

^{13}C NMR spectrum of **25** ($CDCl_3$)

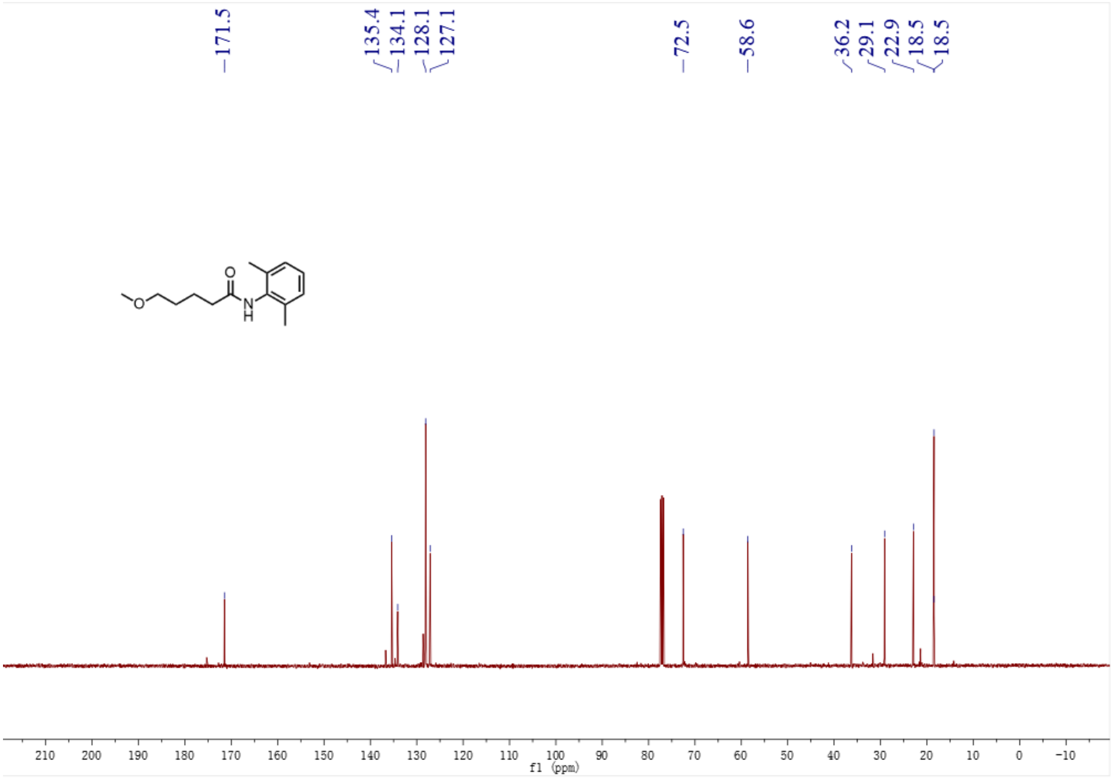

^{1}H NMR spectrum of **26** ($CDCl_3$)

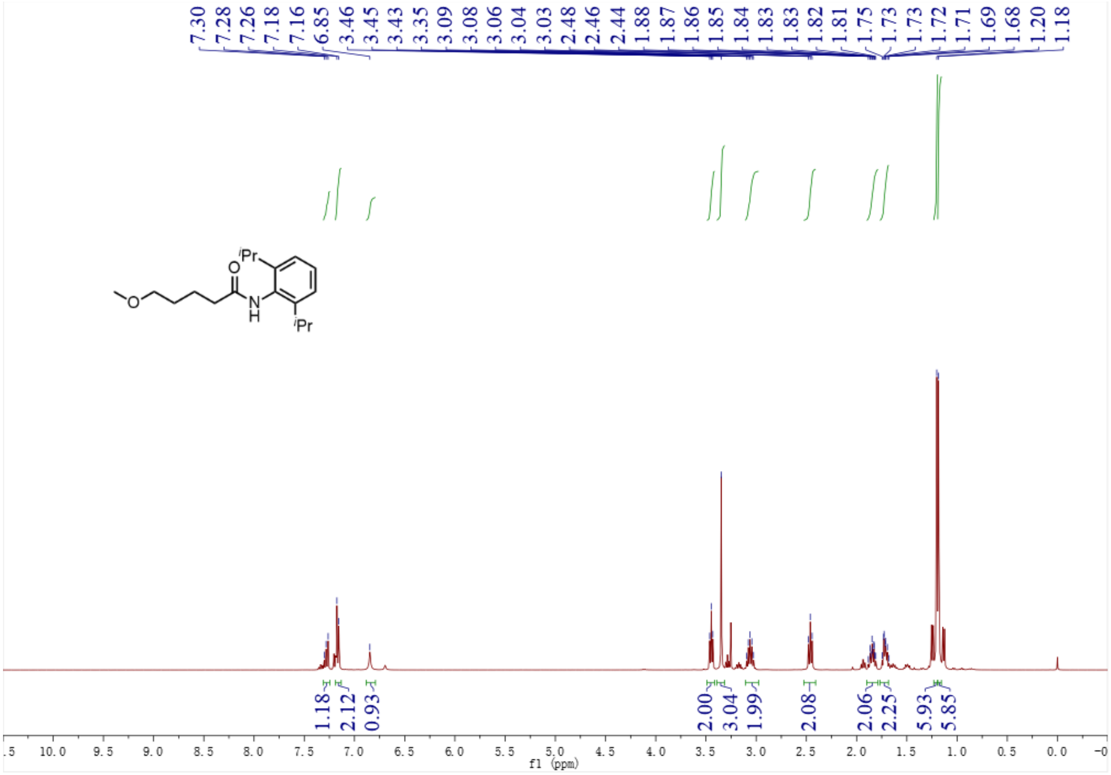

^{13}C NMR spectrum of **26** ($CDCl_3$)

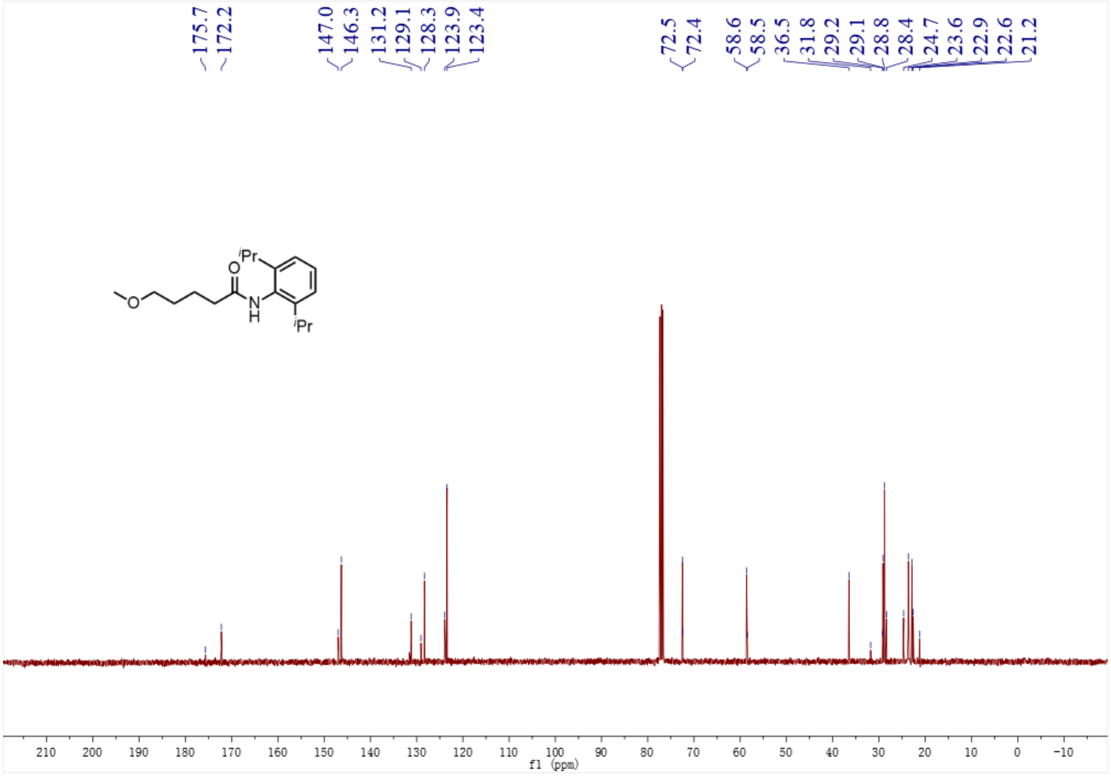

^{1}H NMR spectrum of **27** ($CDCl_3$)

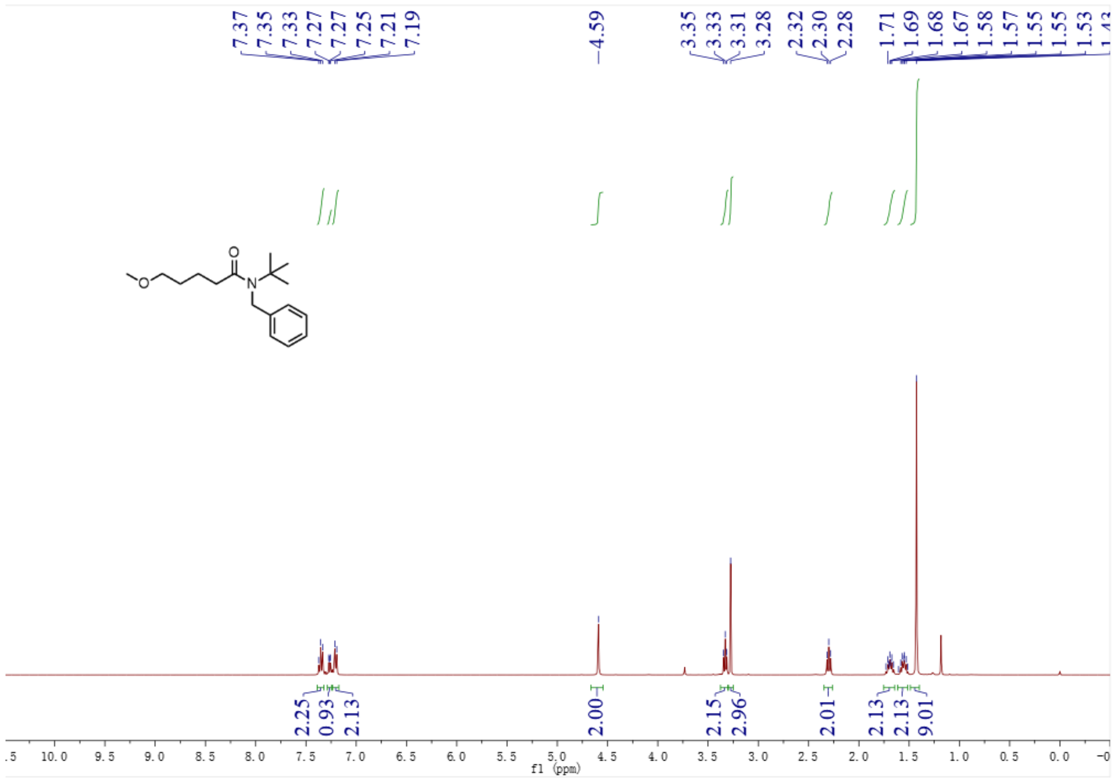

^{13}C NMR spectrum of **27** ($CDCl_3$)

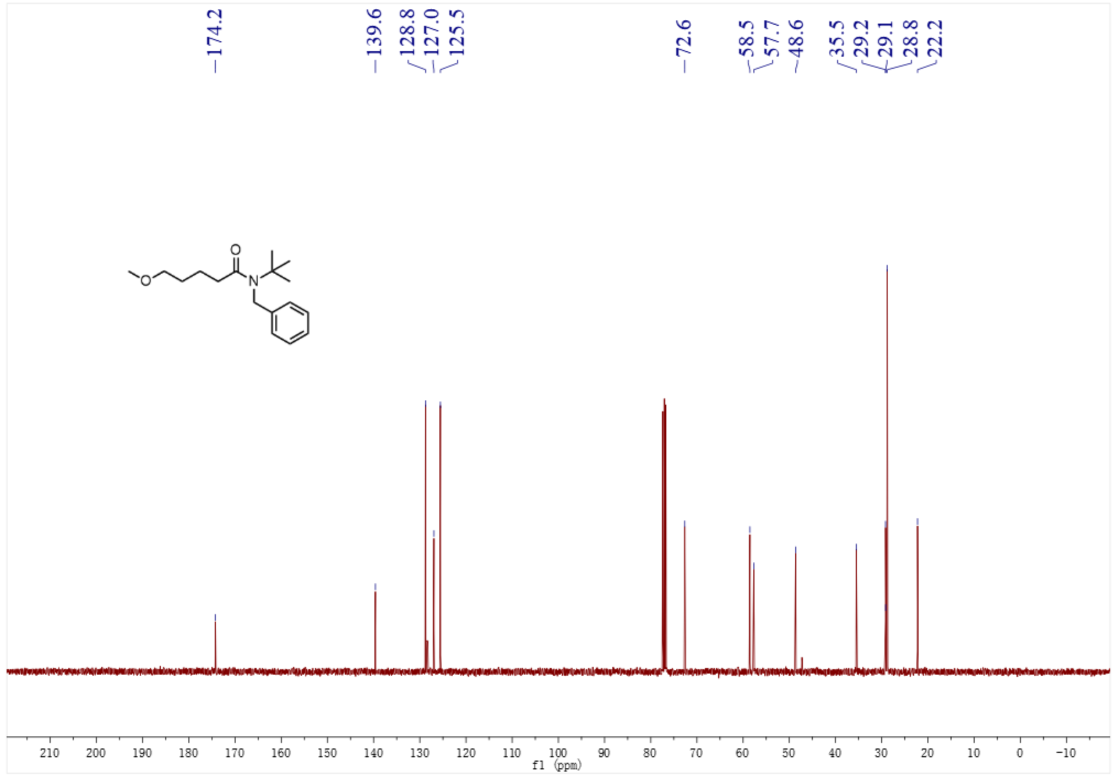

^{1}H NMR spectrum of **28** ($CDCl_3$)

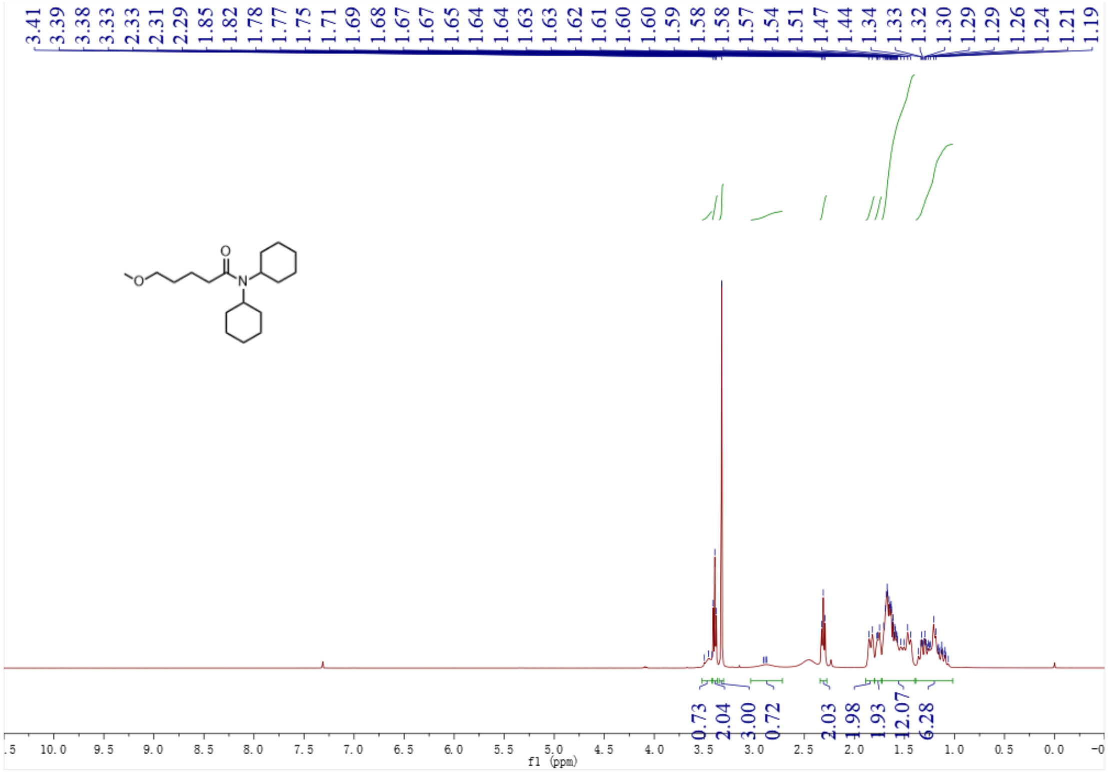

^{13}C NMR spectrum of **28** ($CDCl_3$)

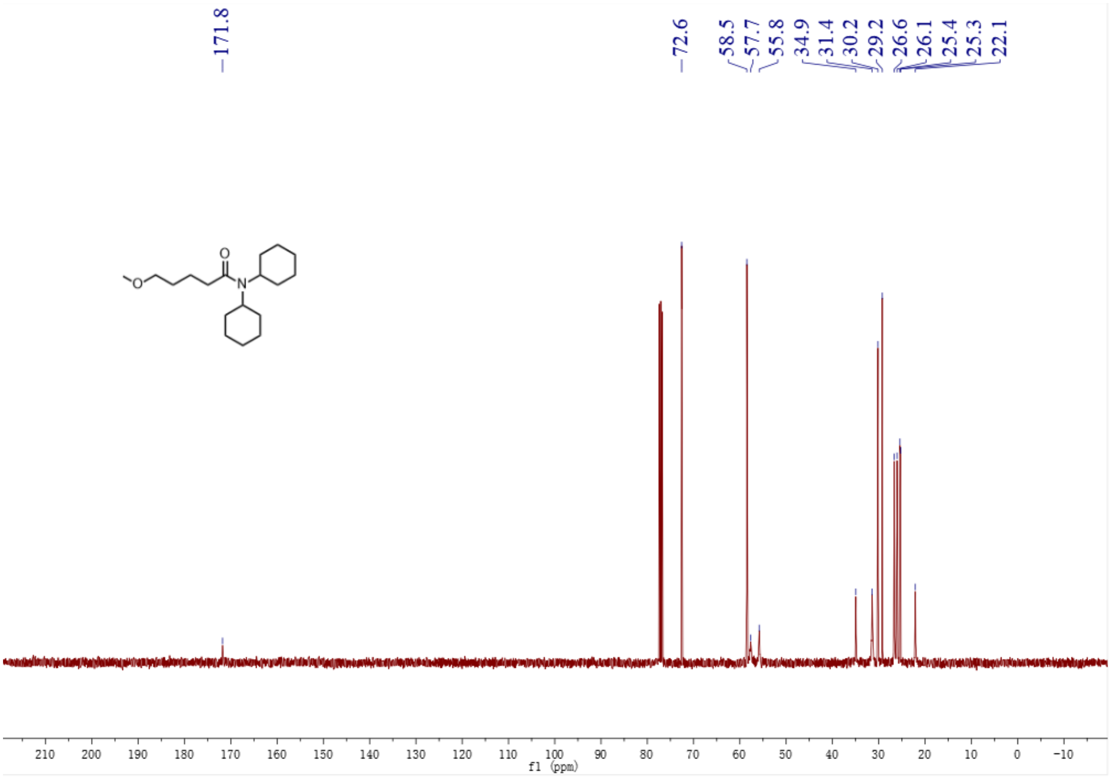

^{1}H NMR spectrum of **29** ($CDCl_3$)

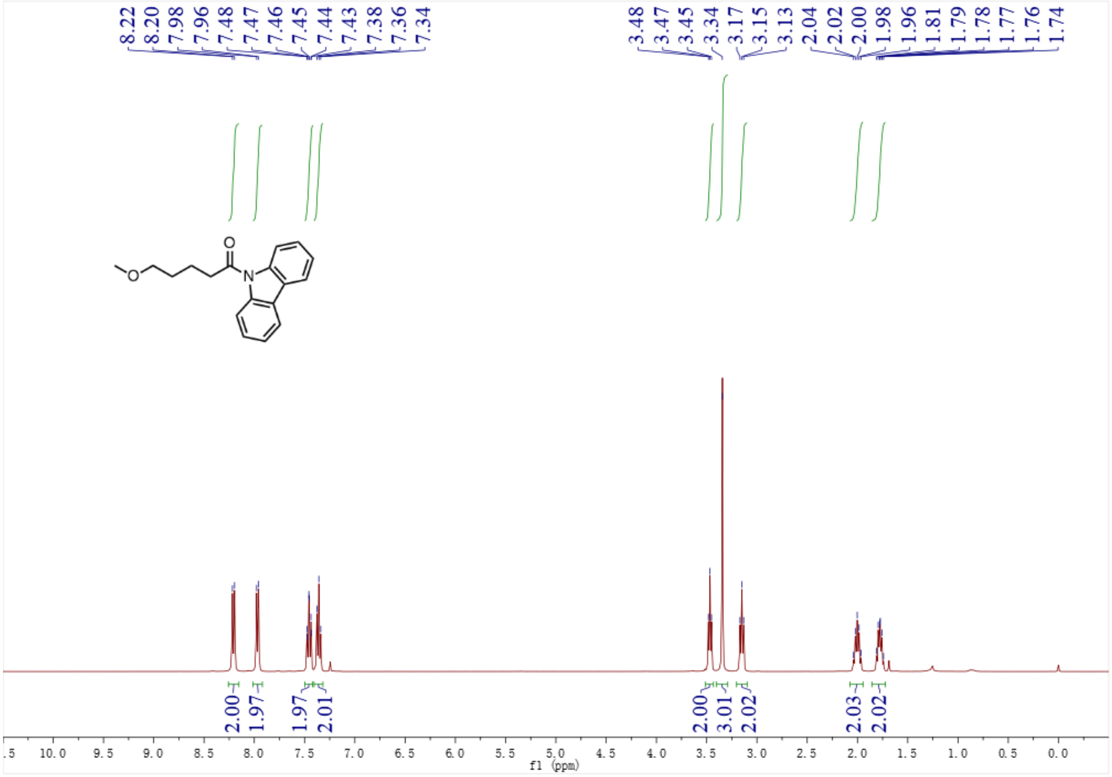

^{13}C NMR spectrum of **29** ($CDCl_3$)

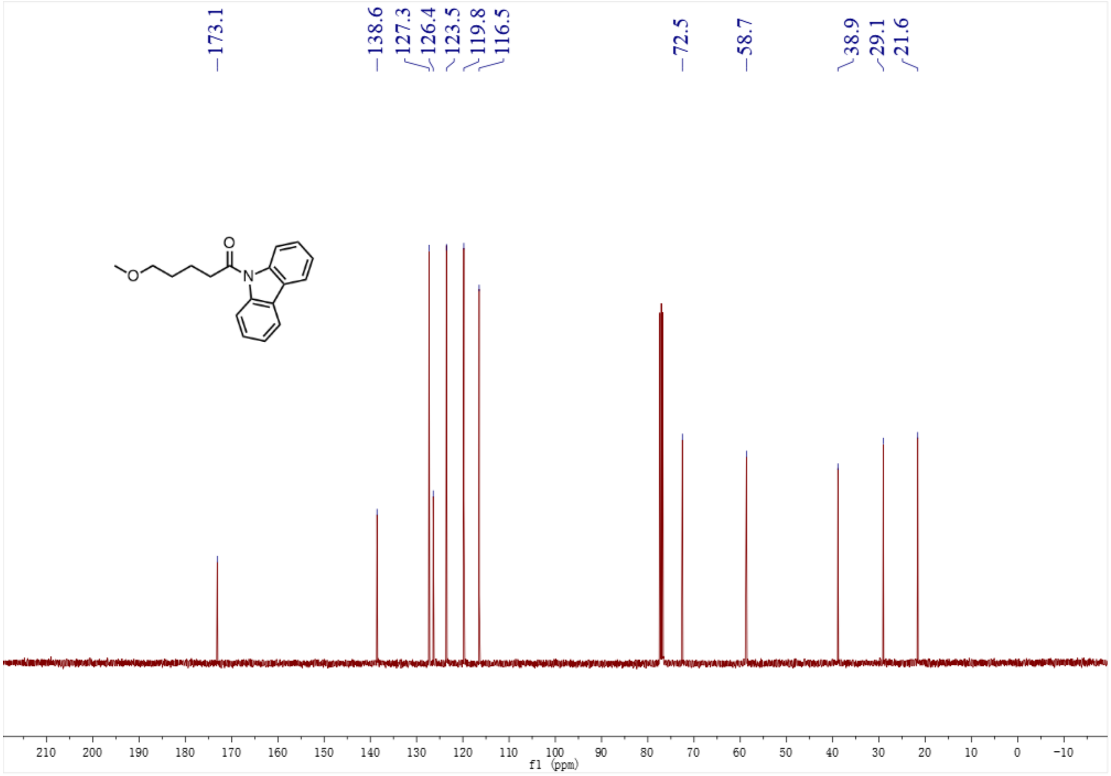

^{1}H NMR spectrum of **30** ($CDCl_3$)

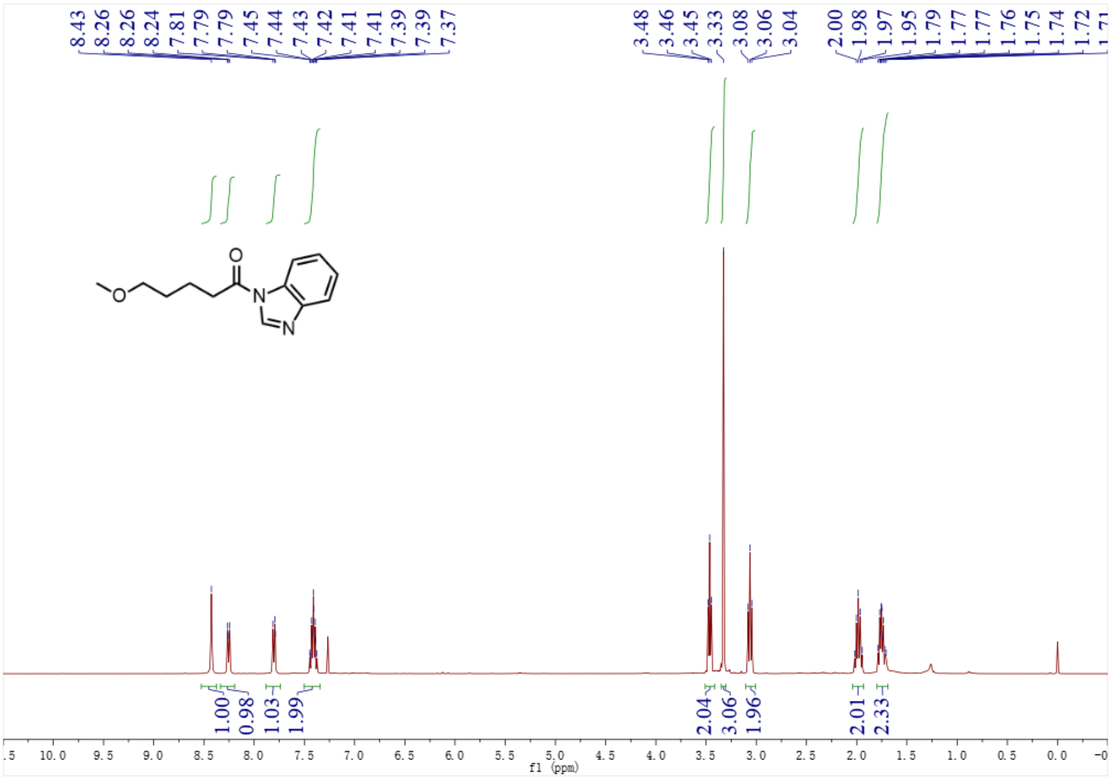

^{13}C NMR spectrum of **30** ($CDCl_3$)

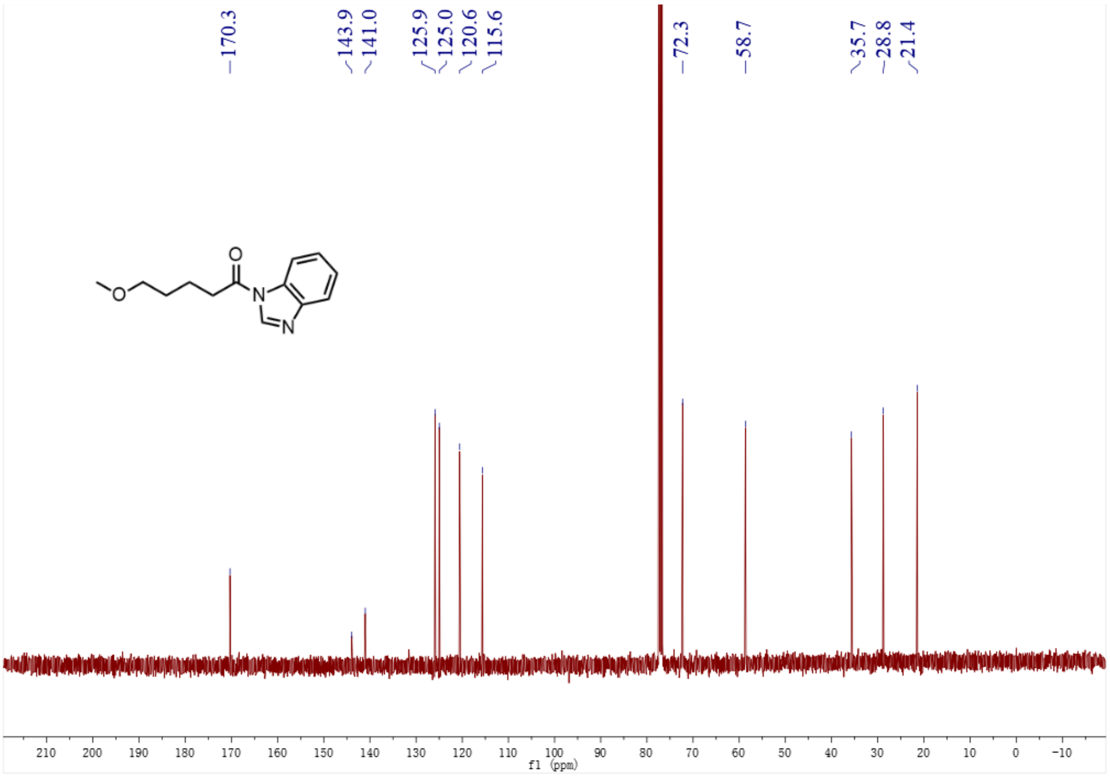

[1]**H NMR** spectrum of **31** ($CDCl_3$)

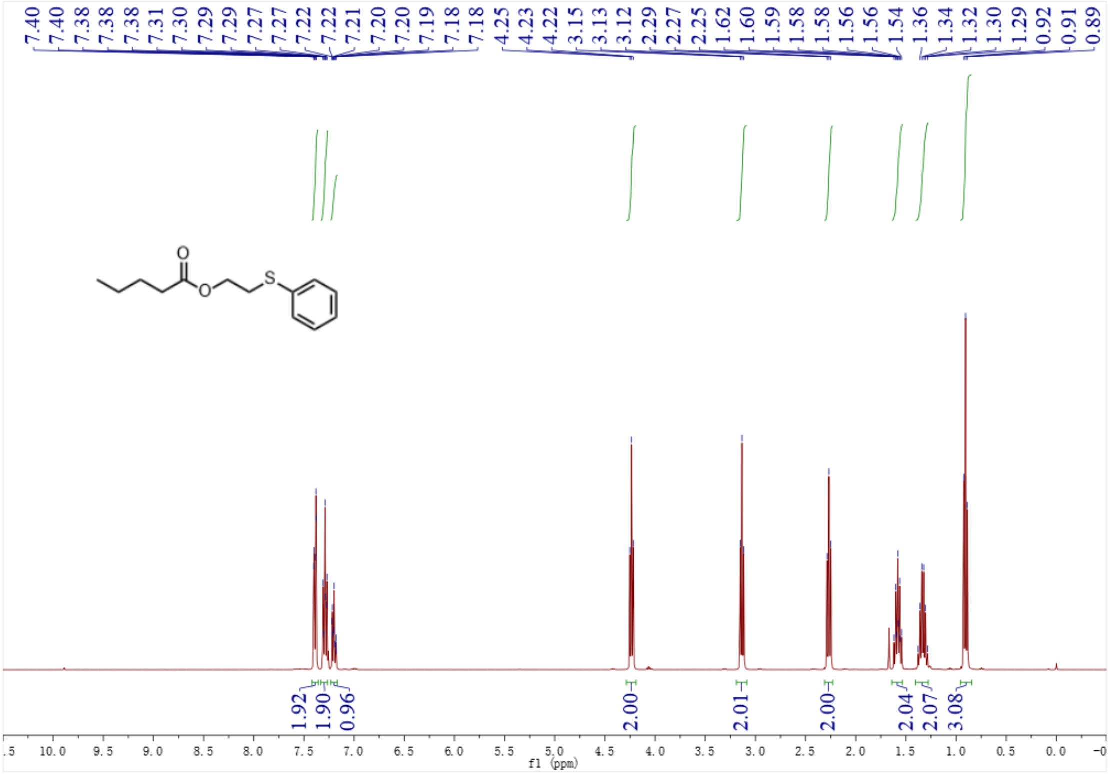

[13]**C NMR** spectrum of **31** ($CDCl_3$)

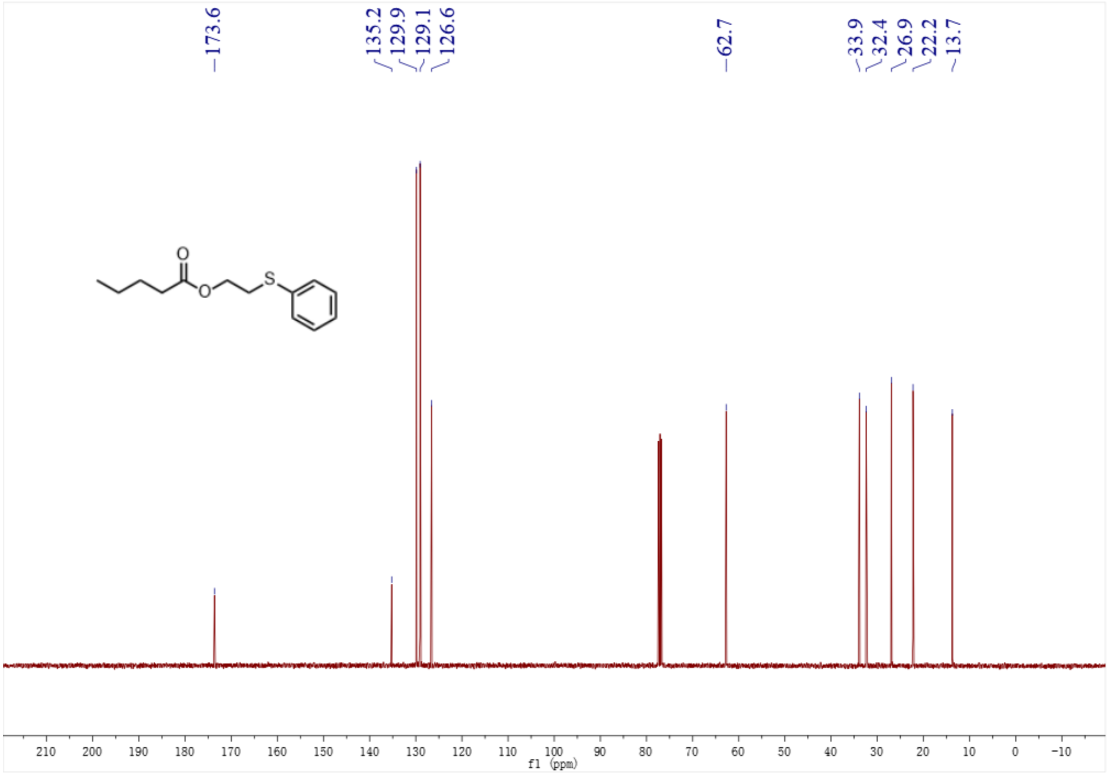

¹H NMR spectrum of **32** ($CDCl_3$)

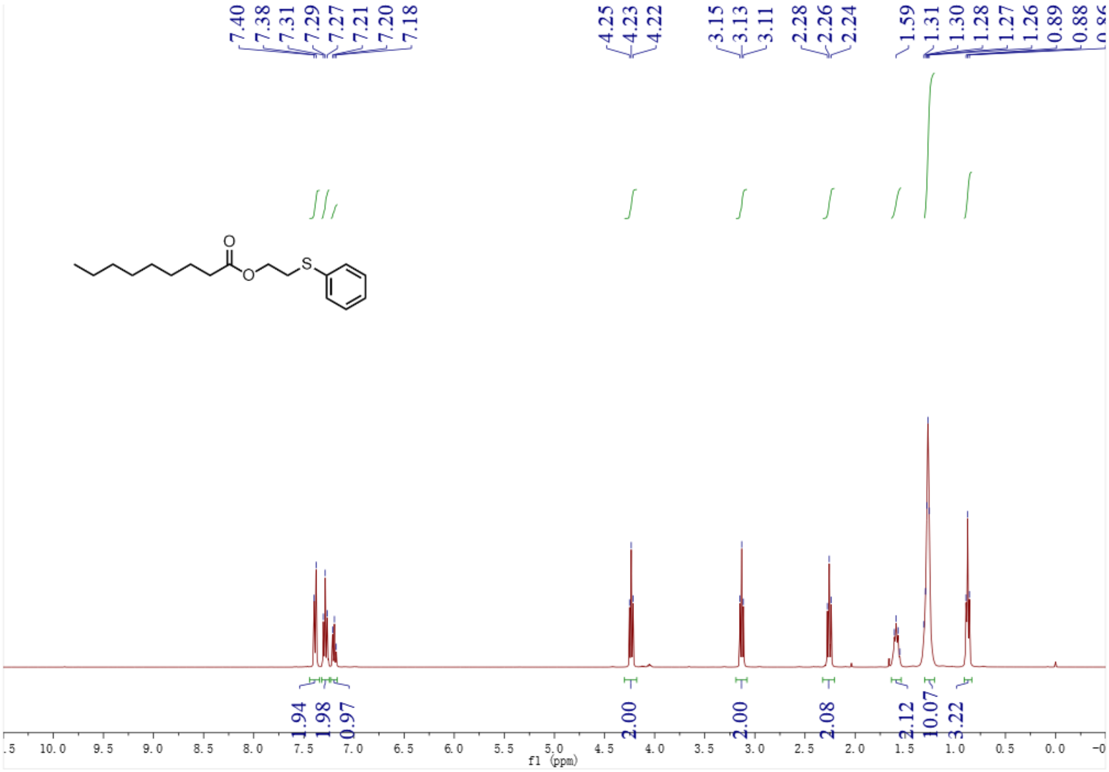

¹³C NMR spectrum of **32** ($CDCl_3$)

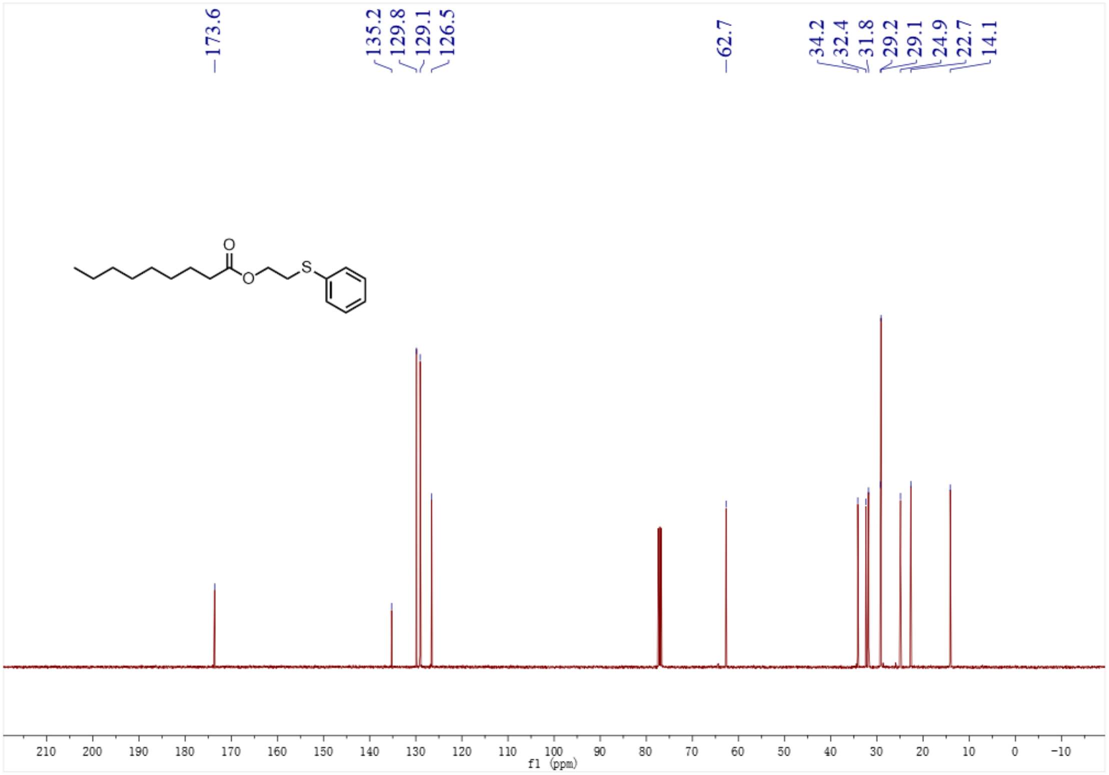

^{1}H NMR spectrum of **33** ($CDCl_3$)

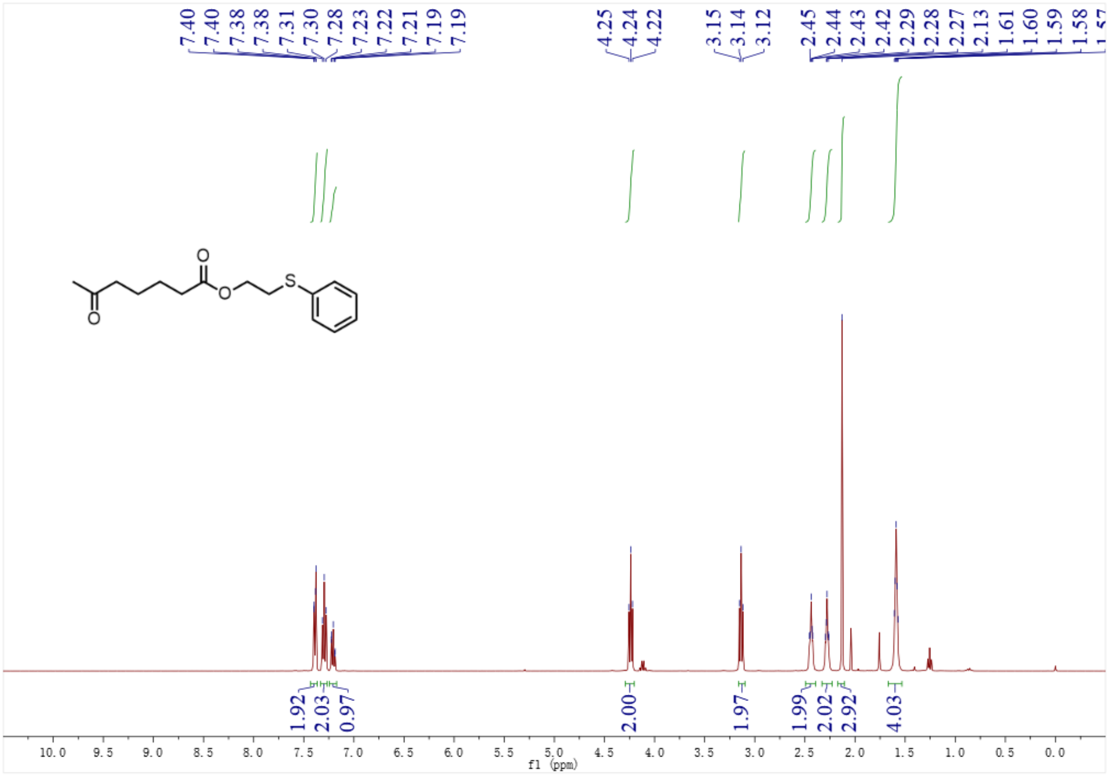

^{13}C NMR spectrum of **33** ($CDCl_3$)

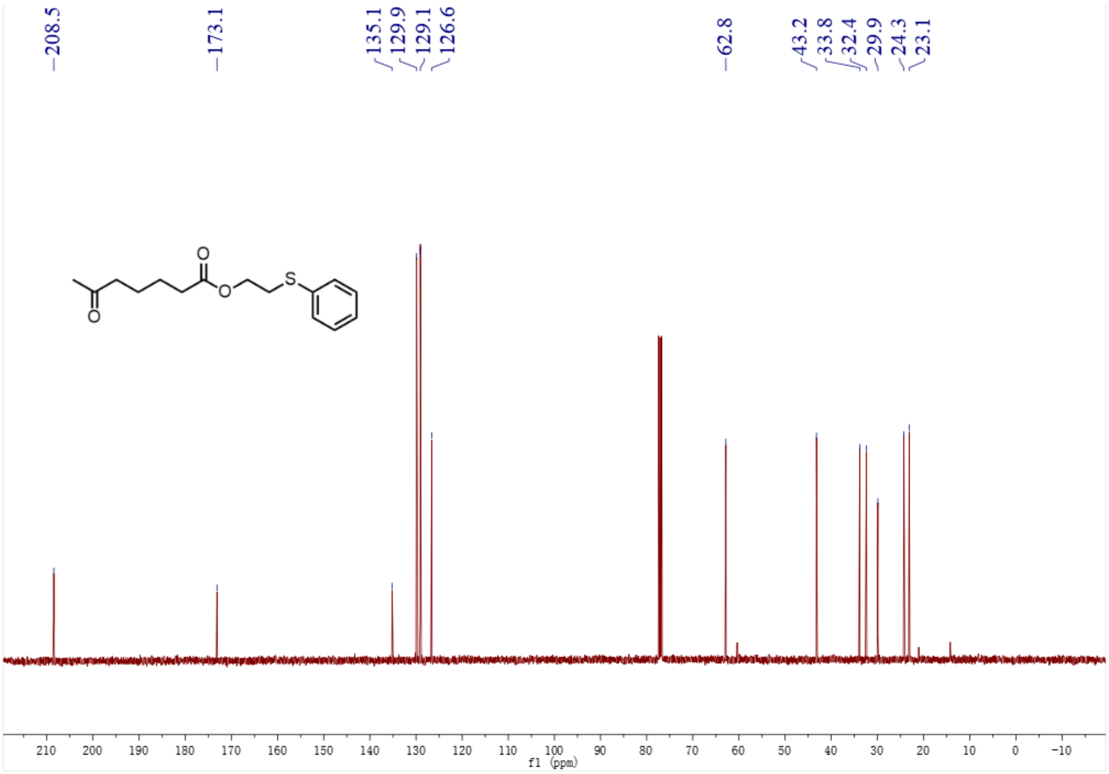

^{1}H NMR spectrum of **34** (CDCl$_3$)

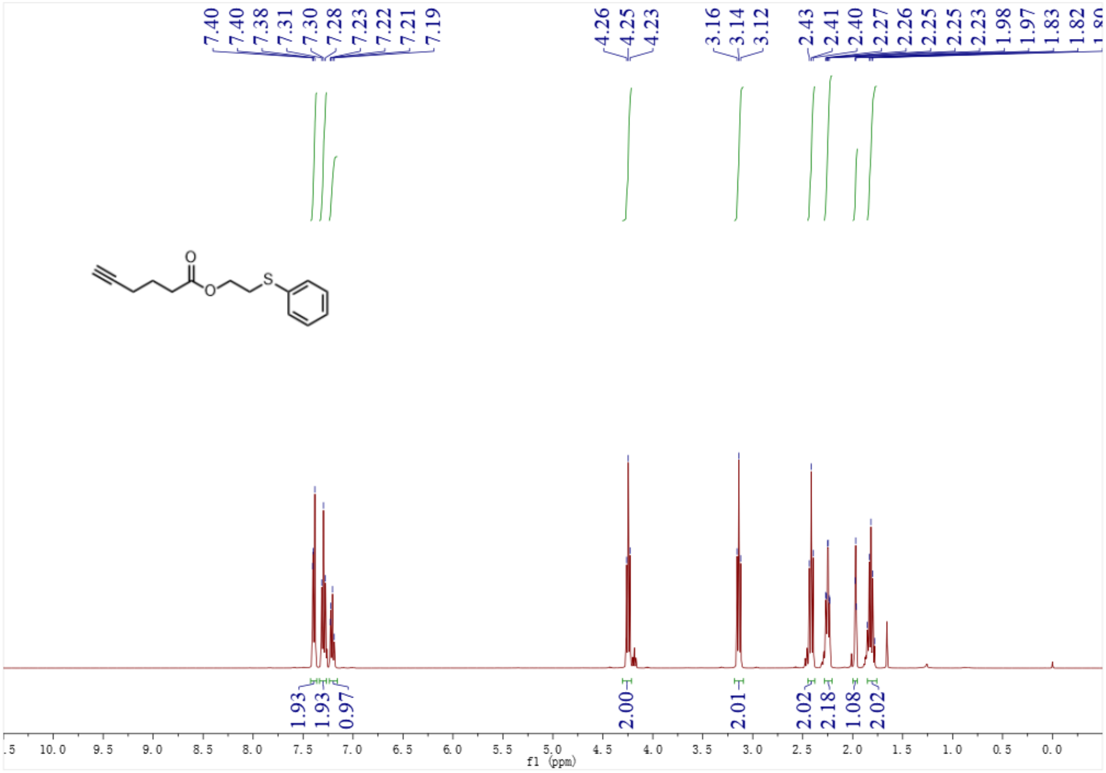

^{13}C NMR spectrum of **34** (CDCl$_3$)

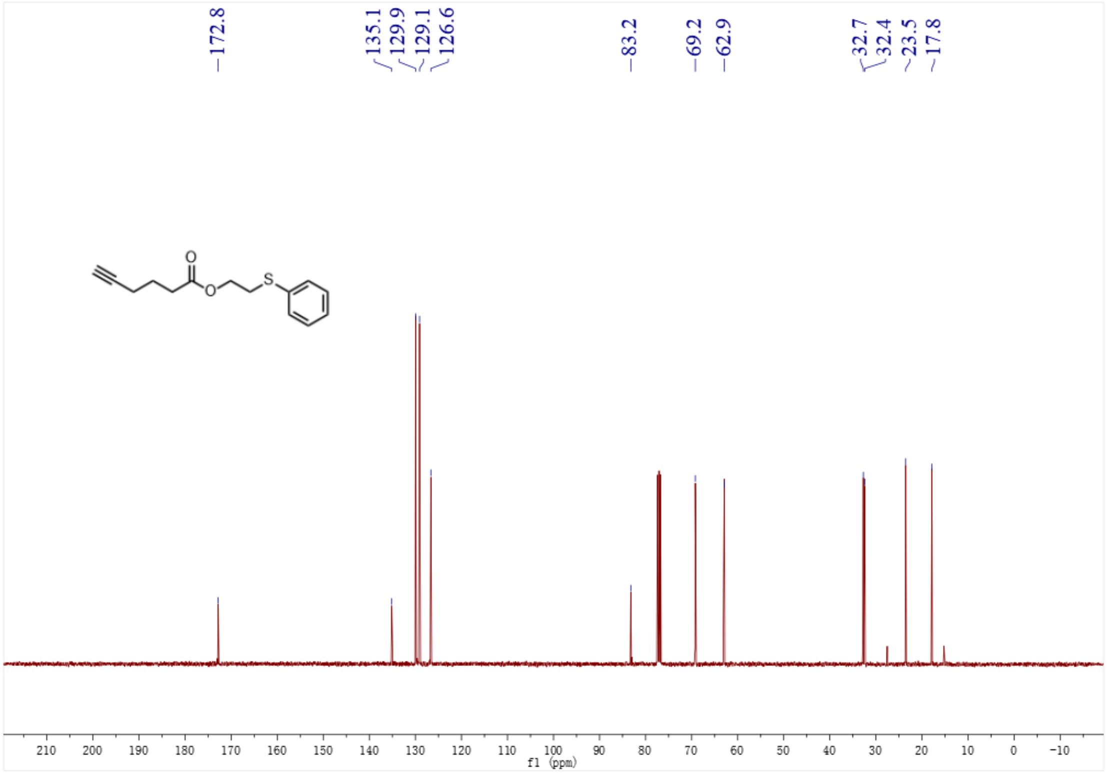

^{1}H NMR spectrum of **35** ($CDCl_3$)

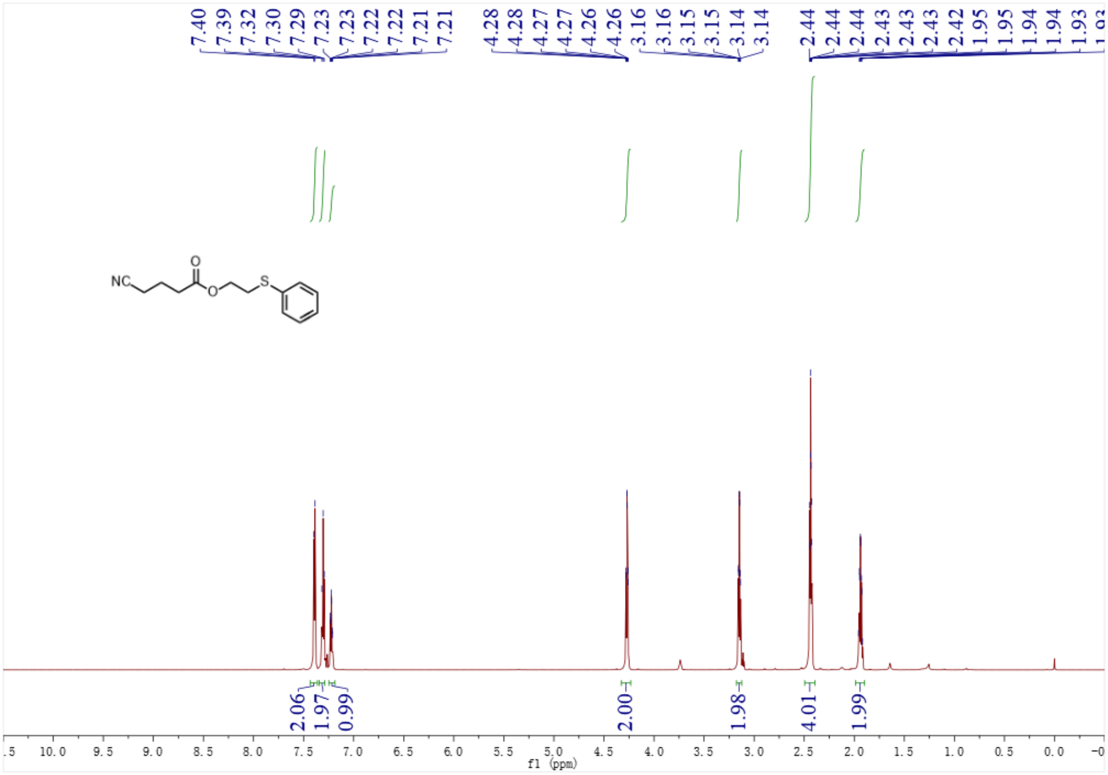

^{13}C NMR spectrum of **35** ($CDCl_3$)

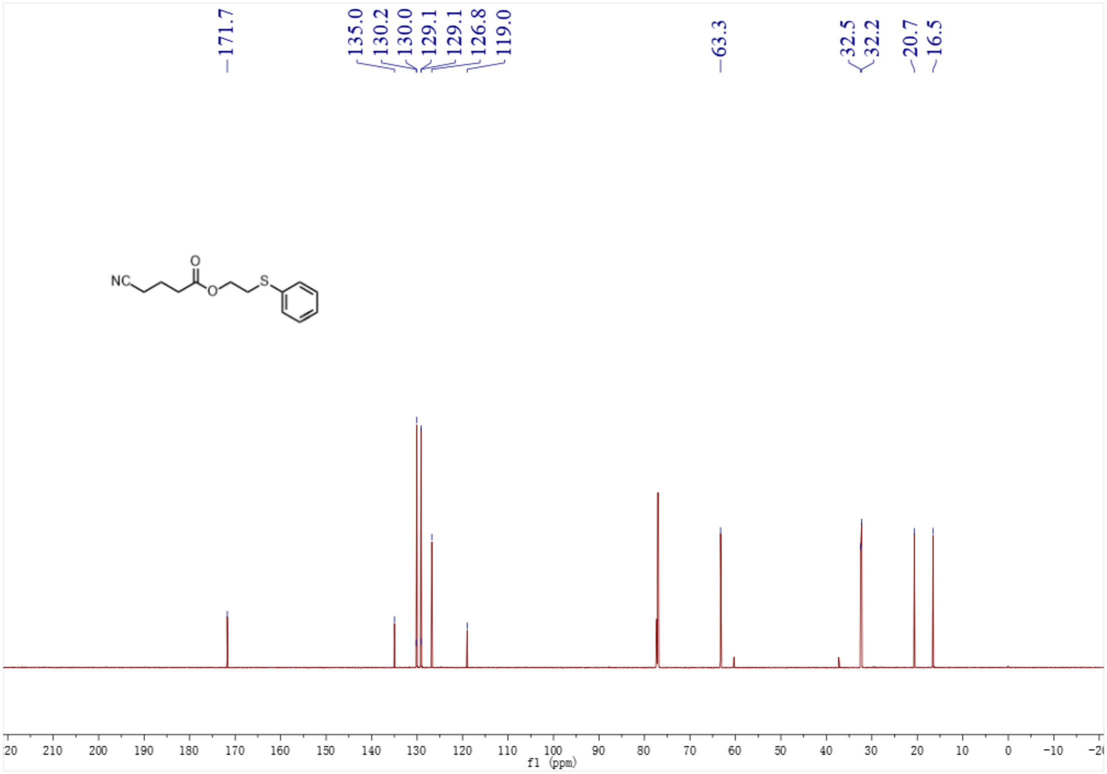

^{1}**H NMR** spectrum of **36** ($CDCl_3$)

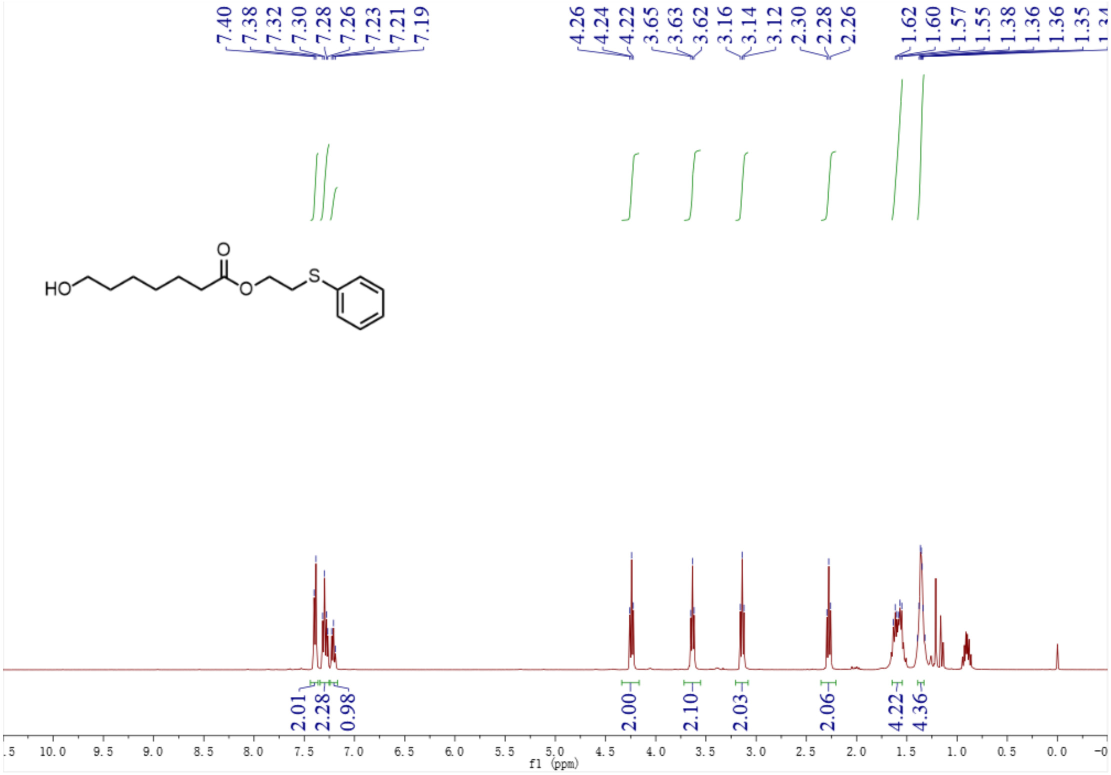

^{13}**C NMR** spectrum of **36** ($CDCl_3$)

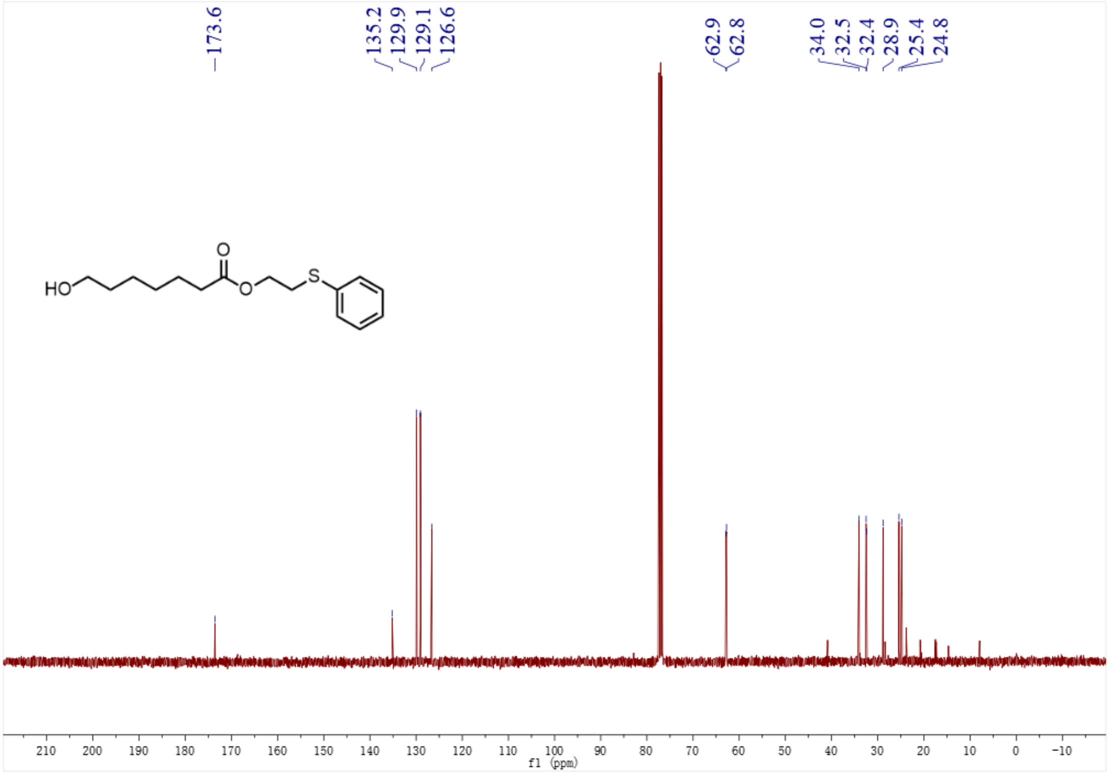

^{1}H NMR spectrum of **37** ($CDCl_3$)

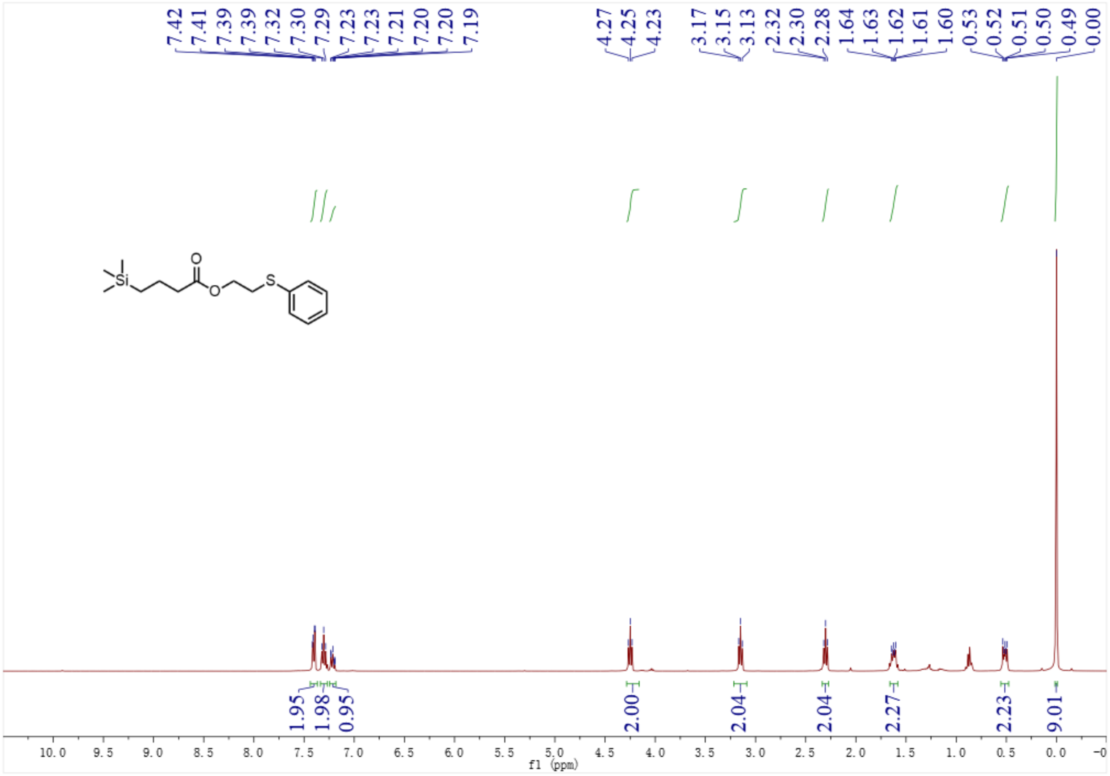

^{13}C NMR spectrum of **37** ($CDCl_3$)

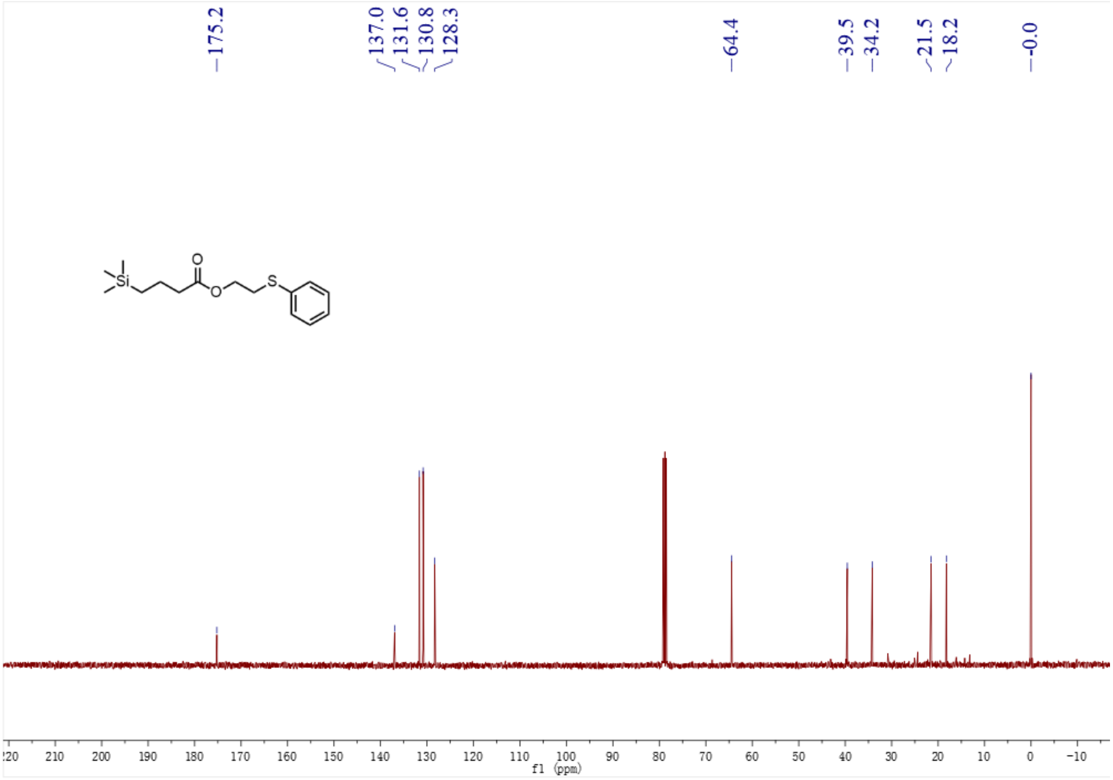

^{1}H NMR spectrum of **38** ($CDCl_3$)

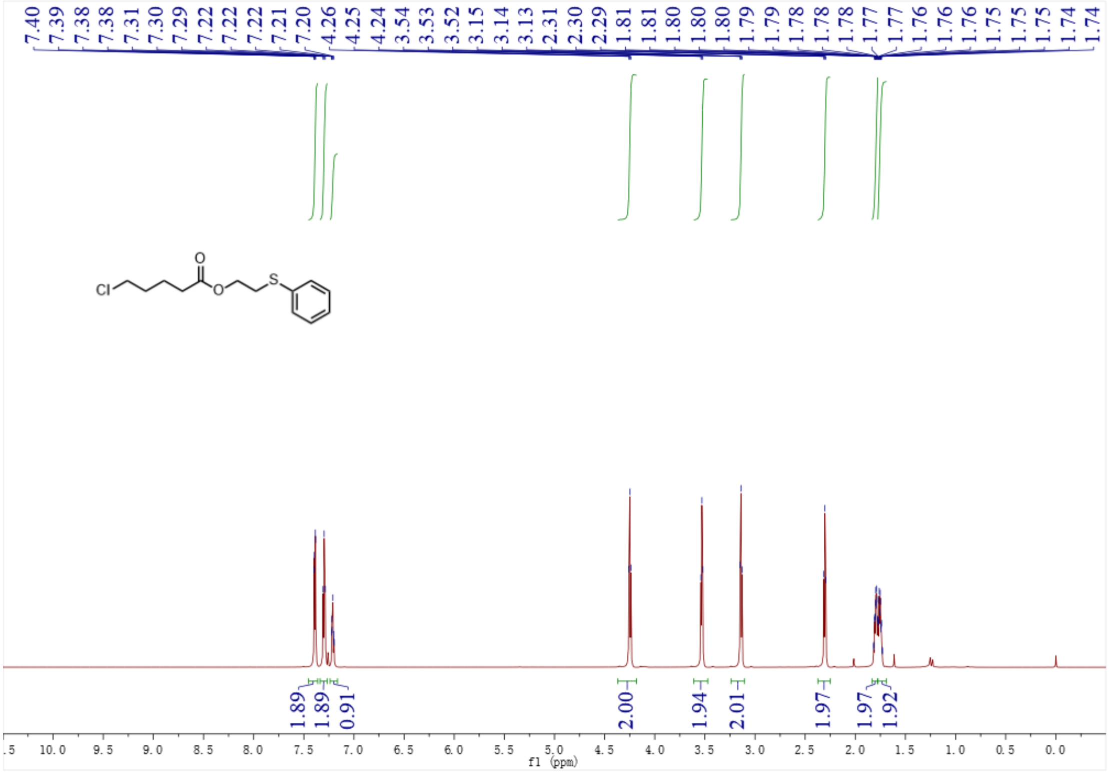

^{13}C NMR spectrum of **38** ($CDCl_3$)

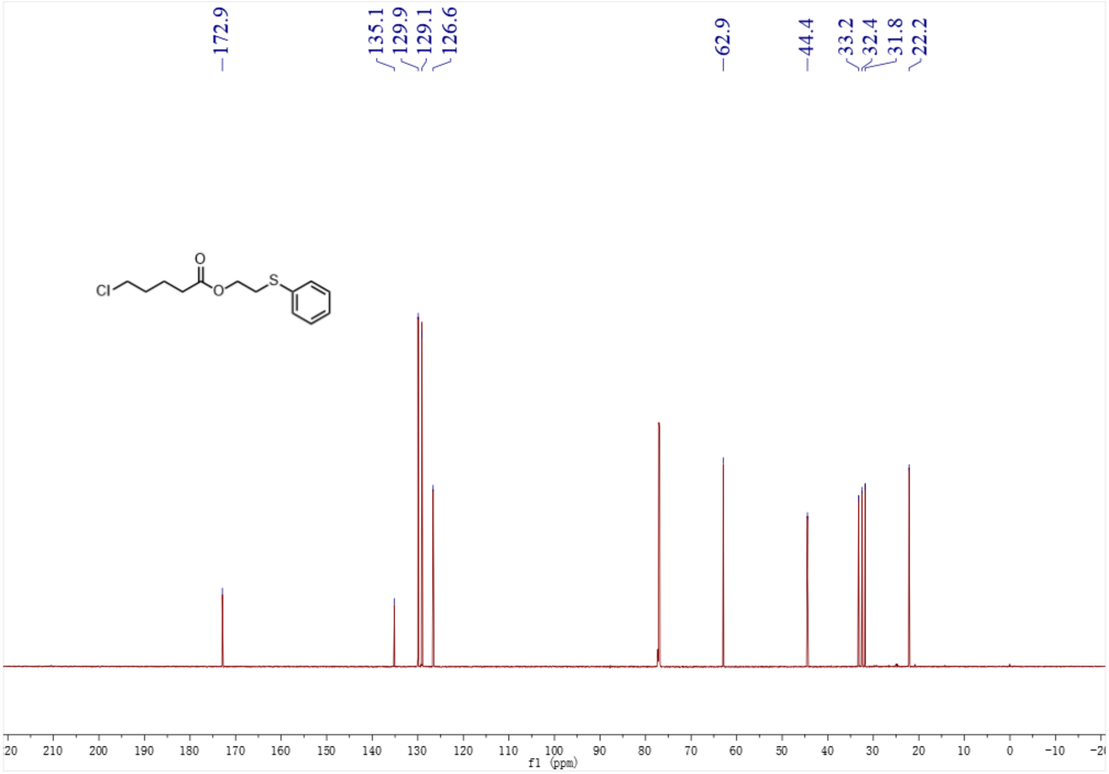

^{1}H NMR spectrum of **39** ($CDCl_3$)

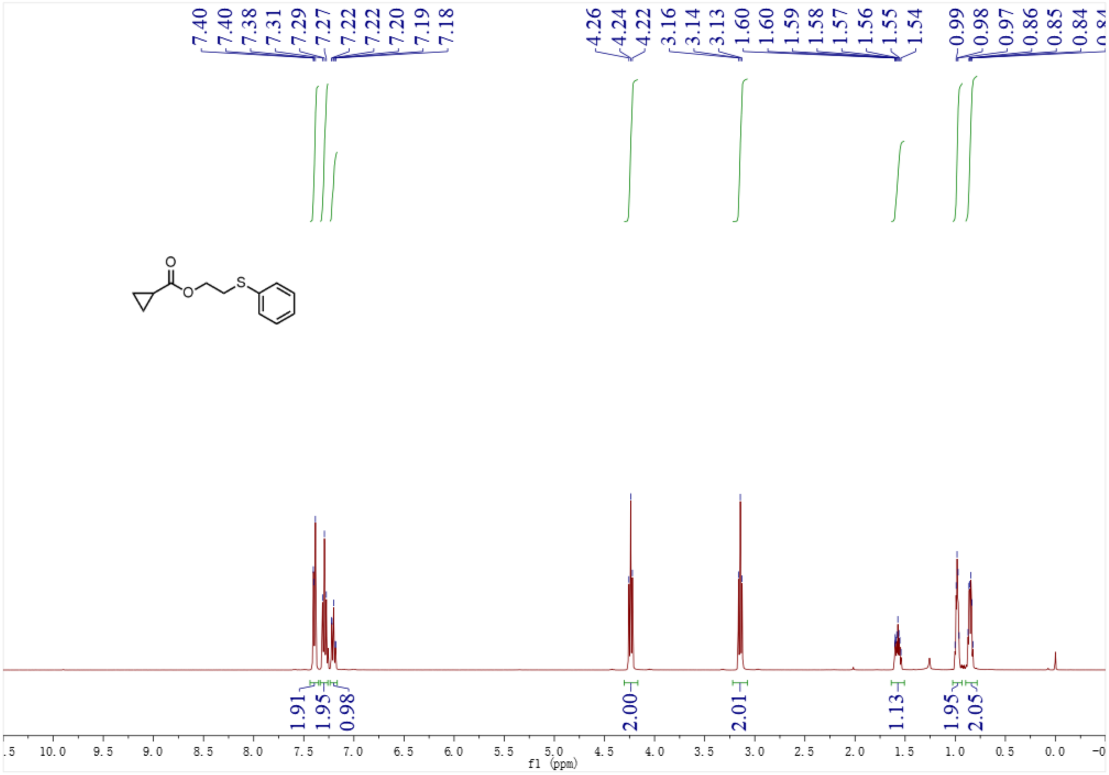

^{13}C NMR spectrum of **39** ($CDCl_3$)

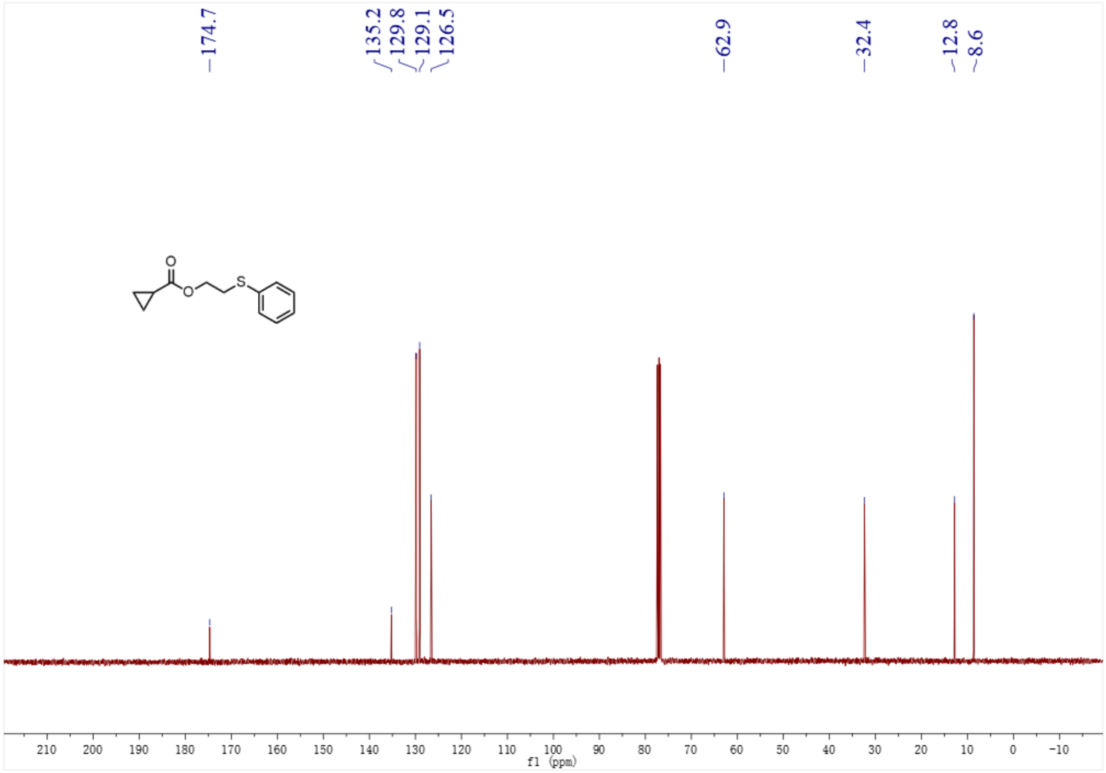

^{1}**H NMR** spectrum of **40** ($CDCl_3$)

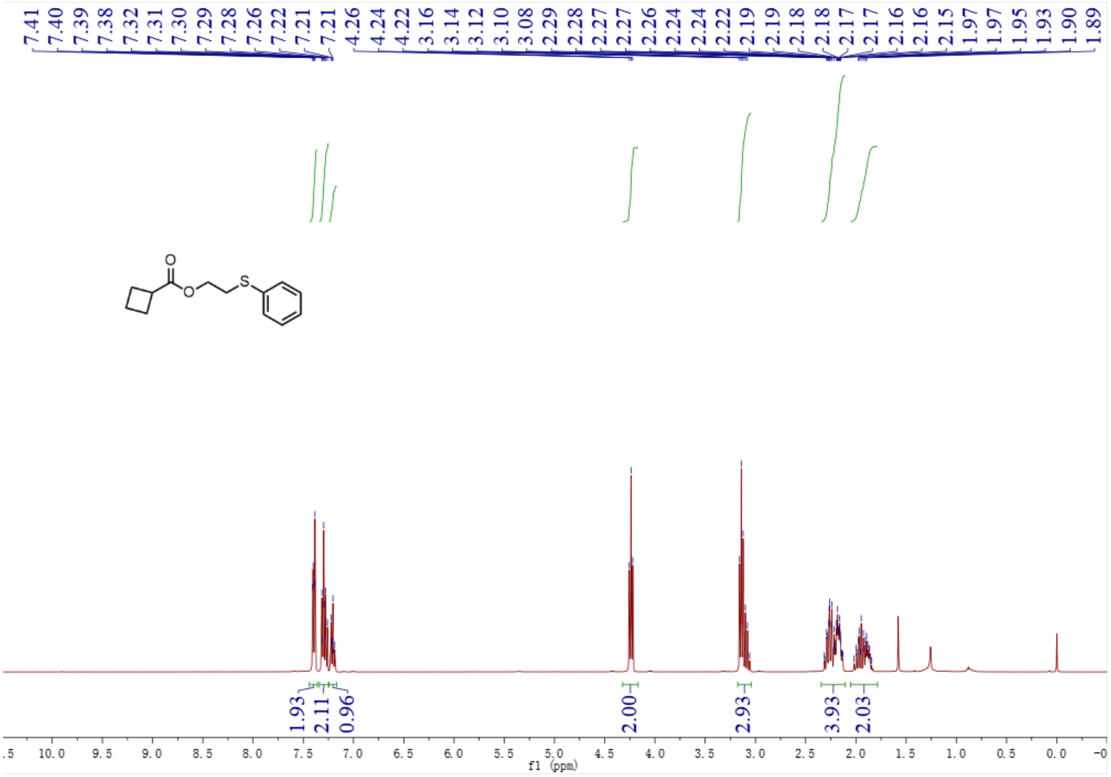

^{13}**C NMR** spectrum of **40** ($CDCl_3$)

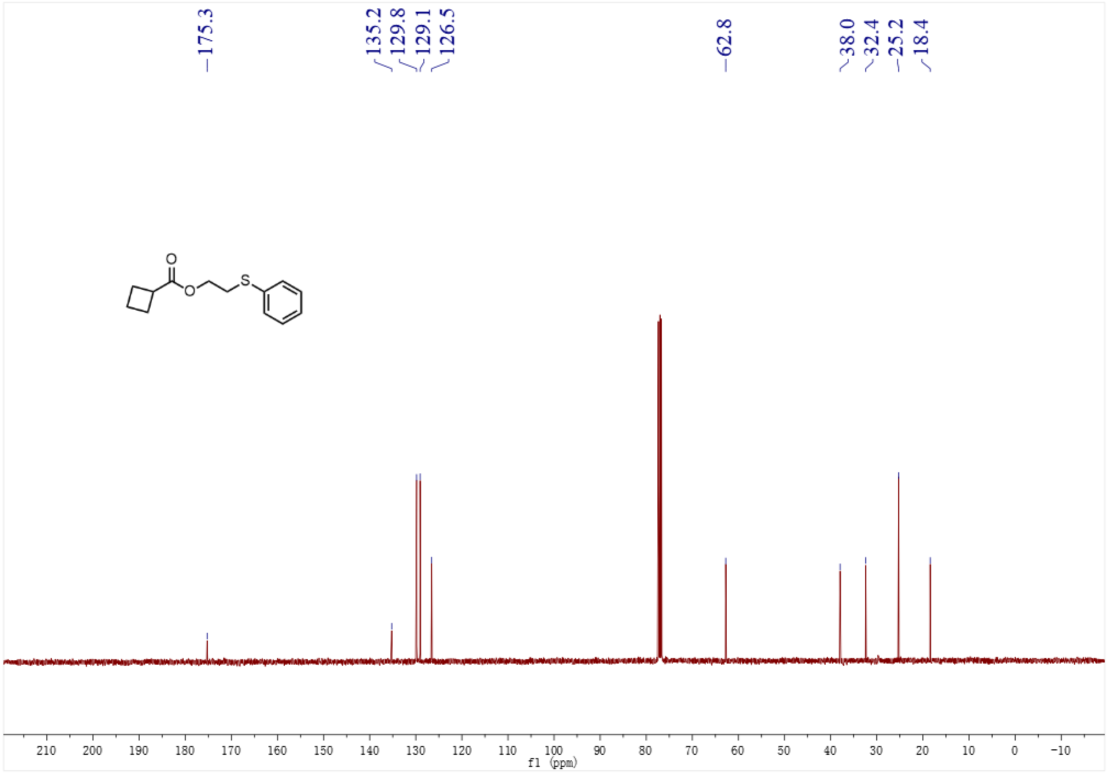

^{1}**H NMR** spectrum of **41** ($CDCl_3$)

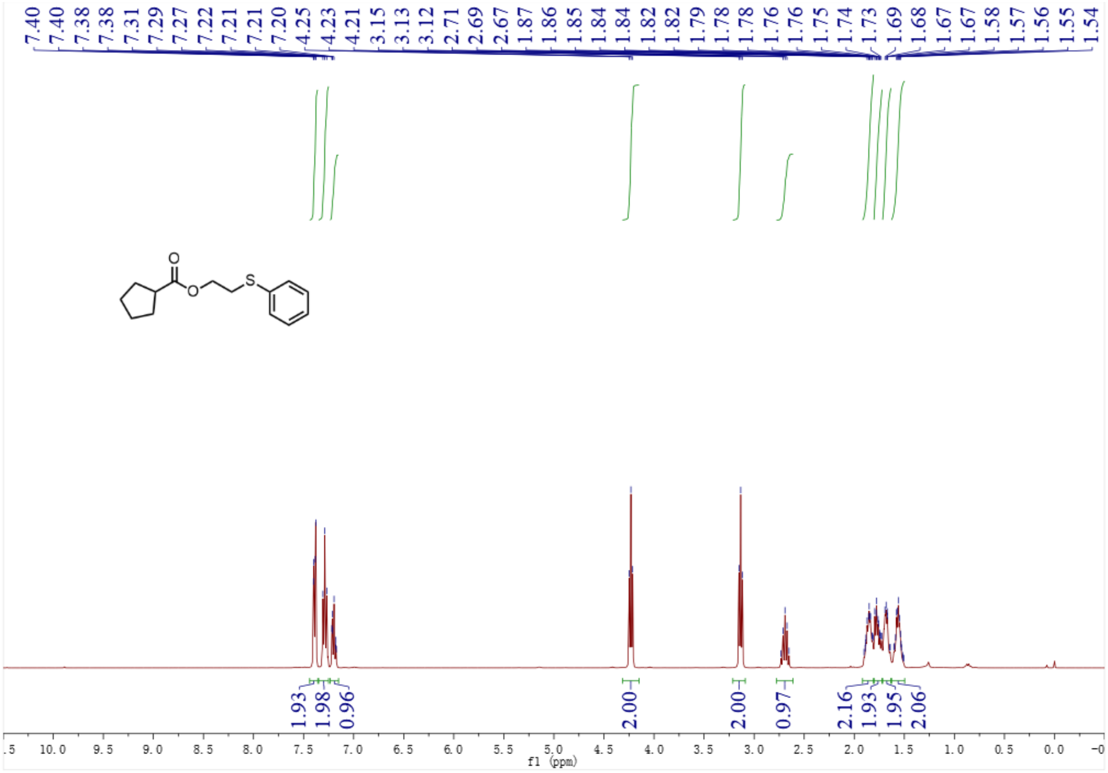

^{13}**C NMR** spectrum of **41** ($CDCl_3$)

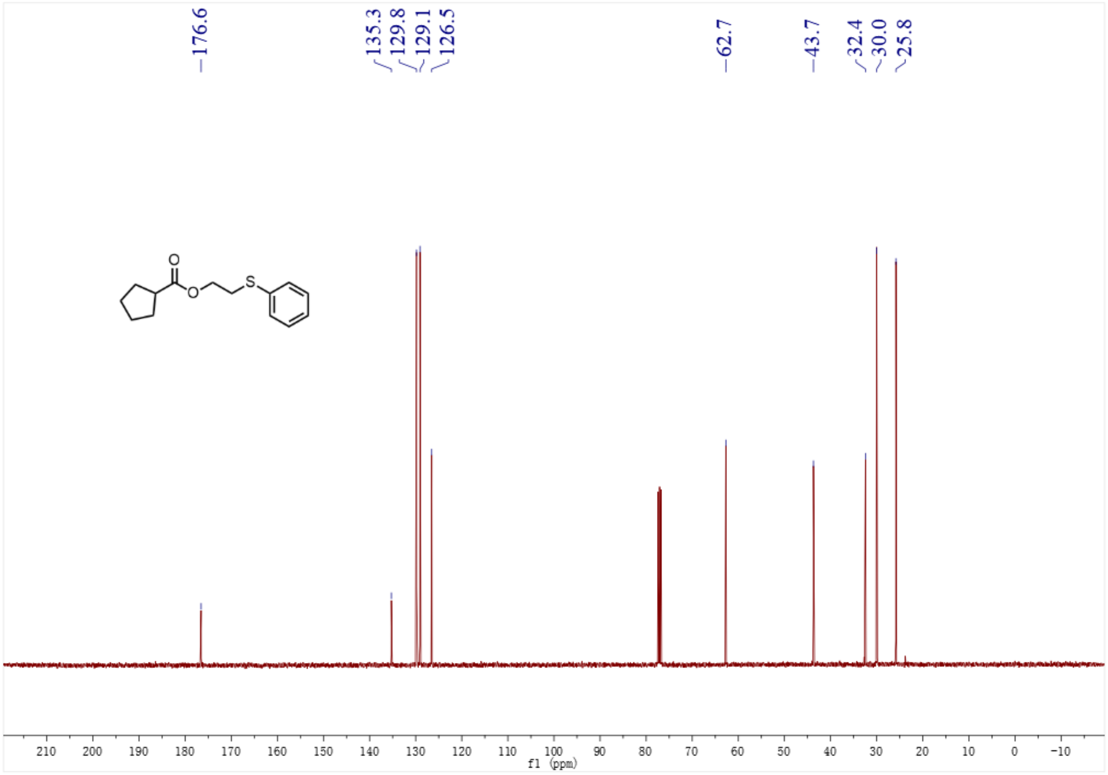

^{1}H NMR spectrum of 42 ($CDCl_3$)

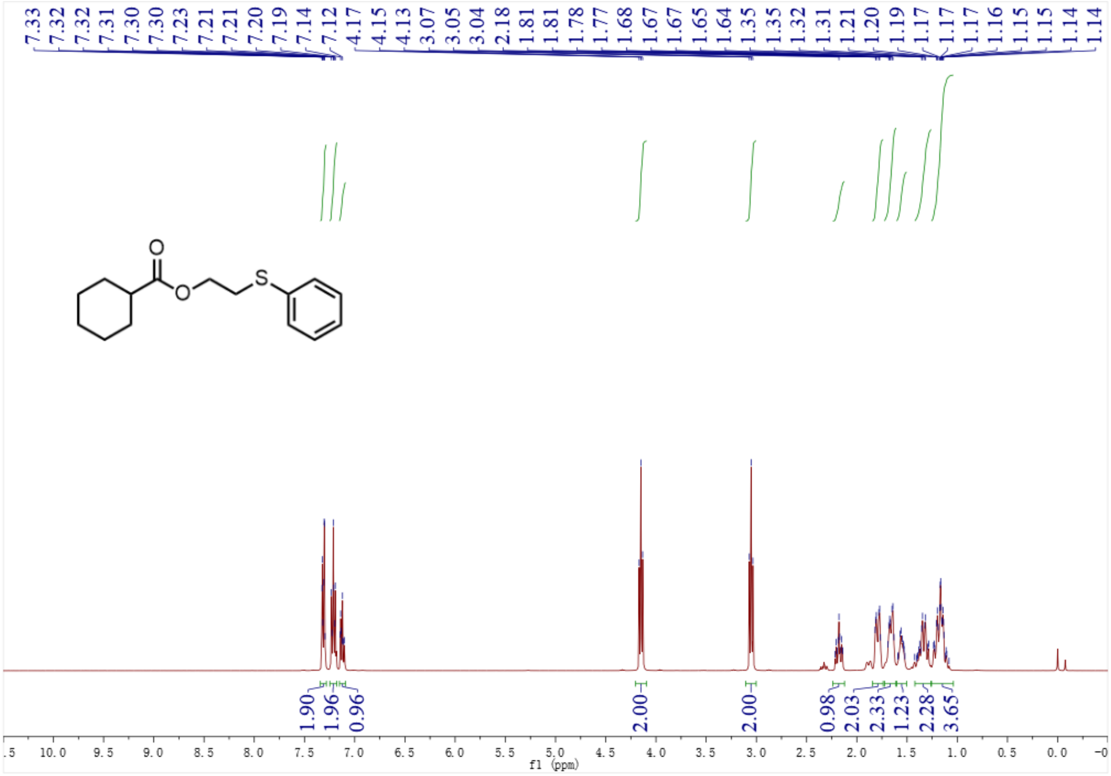

^{13}C NMR spectrum of 42 ($CDCl_3$)

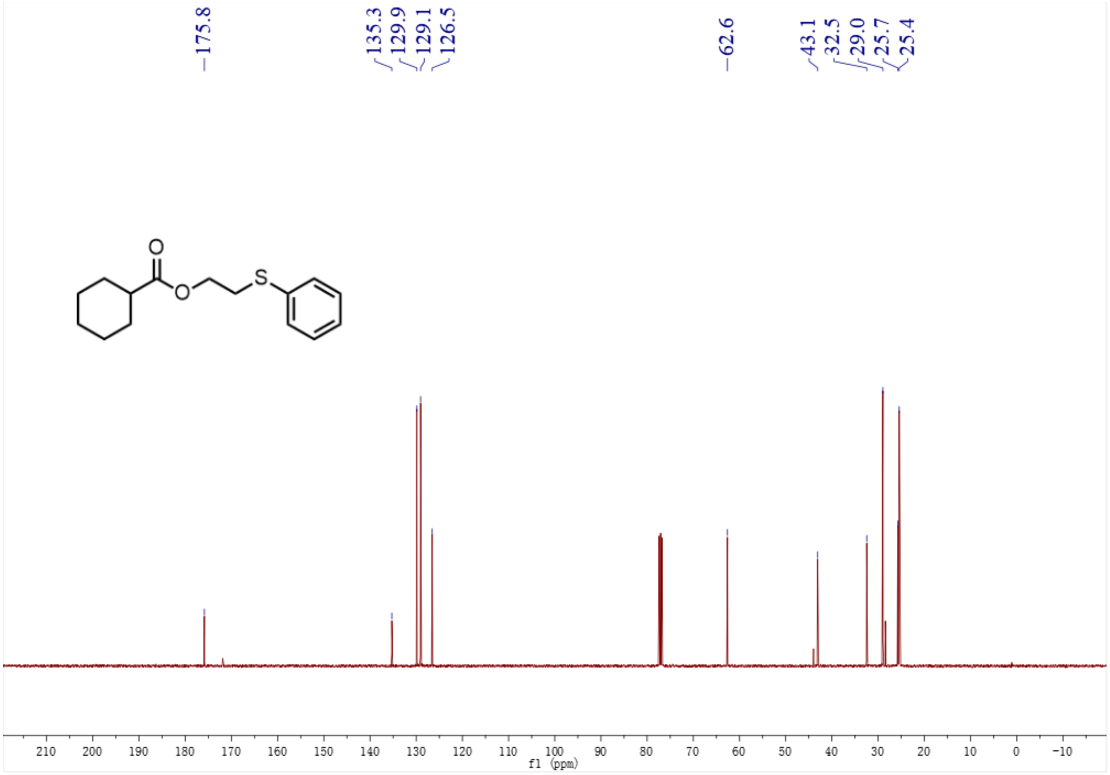

^{1}H NMR spectrum of **43** ($CDCl_3$)

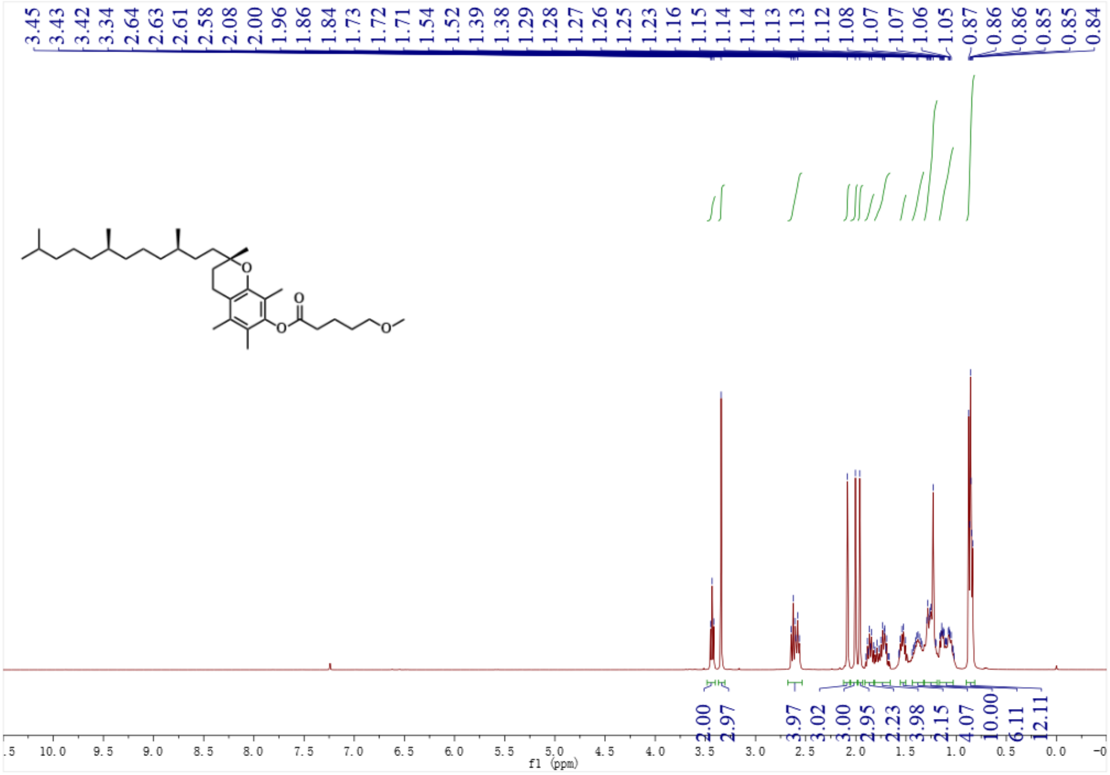

^{13}C NMR spectrum of **43** ($CDCl_3$)

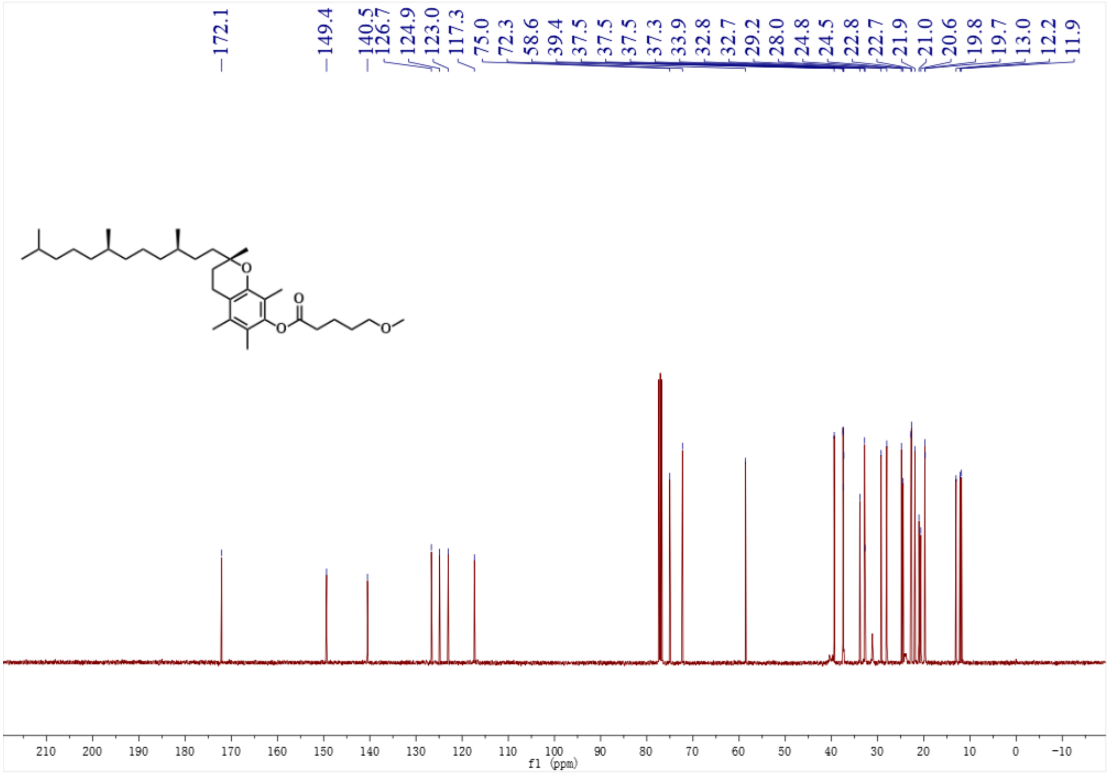

^{1}H NMR spectrum of 44 ($CDCl_3$)

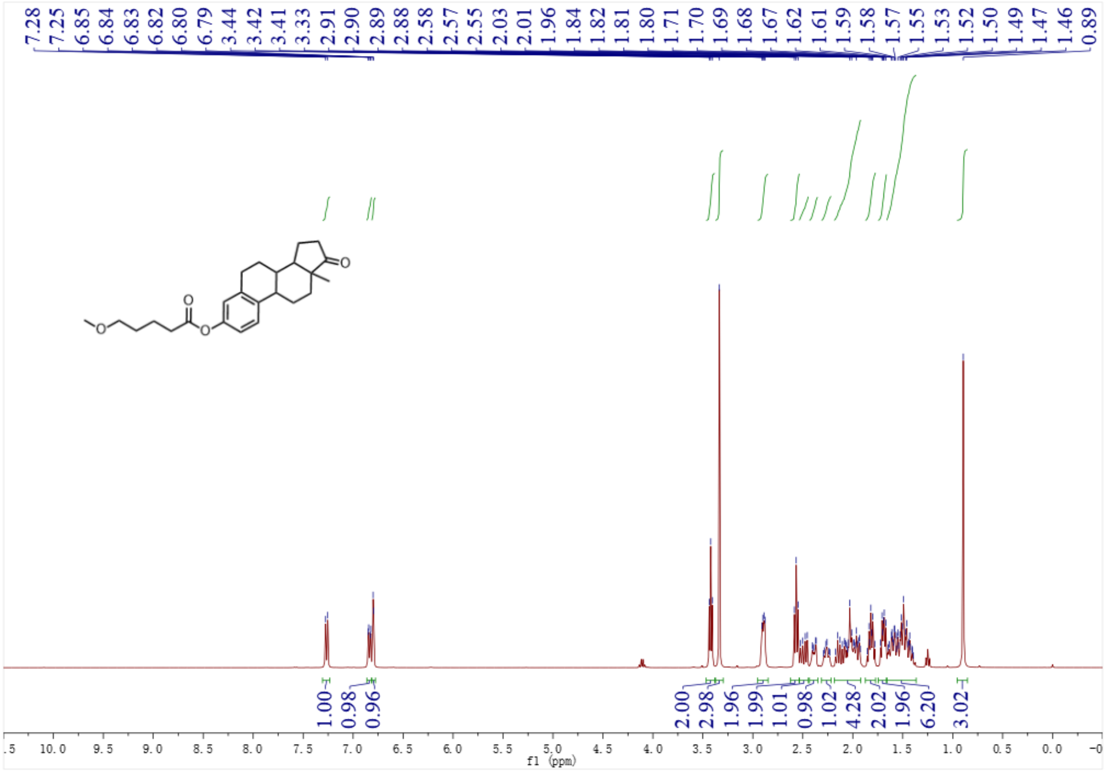

^{13}C NMR spectrum of 44 ($CDCl_3$)

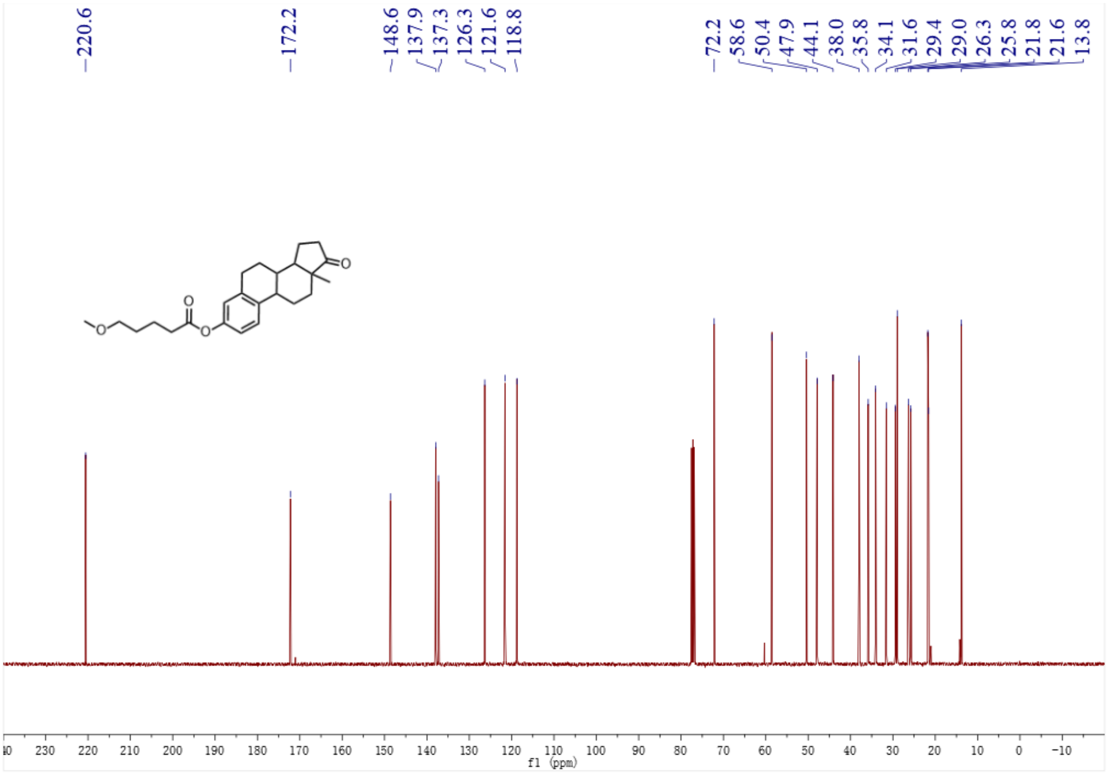

^{1}**H NMR** spectrum of **45** ($CDCl_3$)

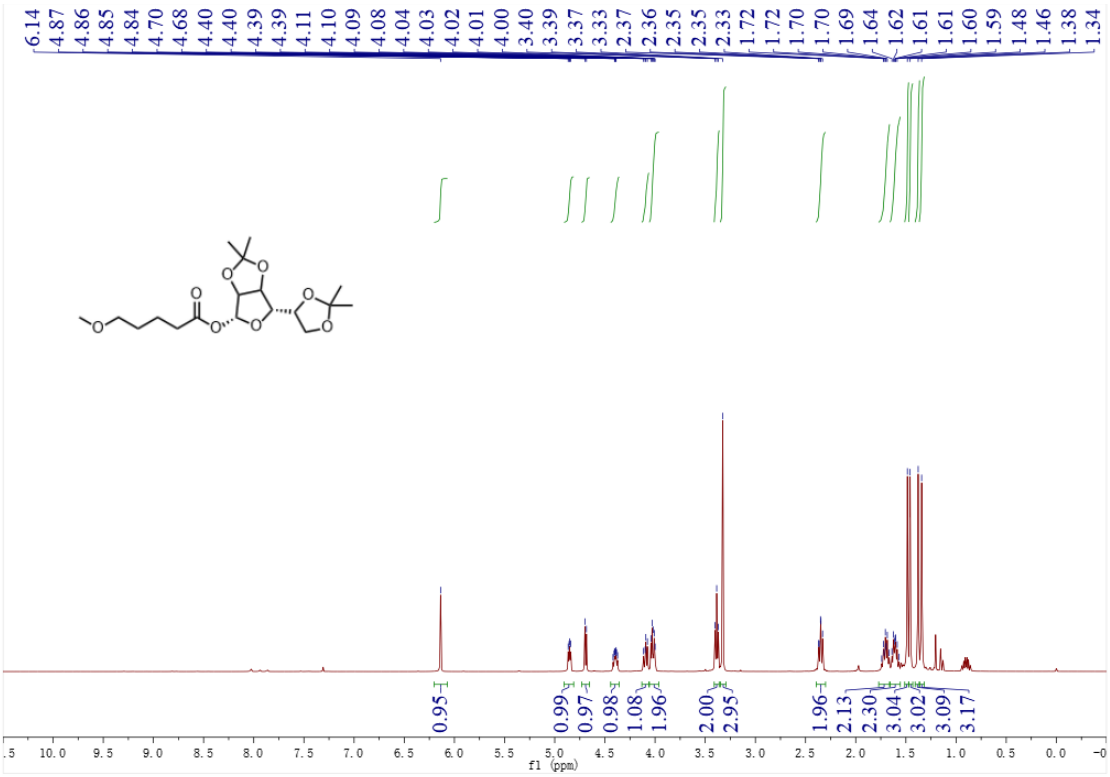

^{13}**C NMR** spectrum of **45** ($CDCl_3$)

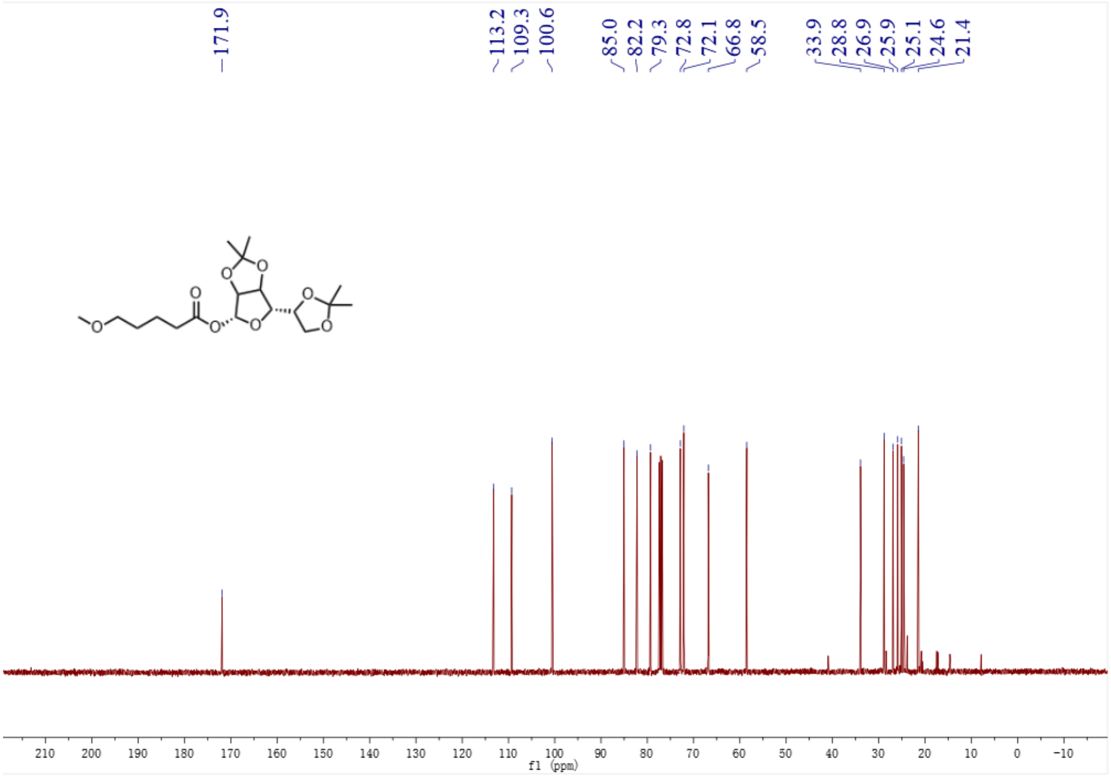

^{1}**H NMR** spectrum of **46** ($CDCl_3$)

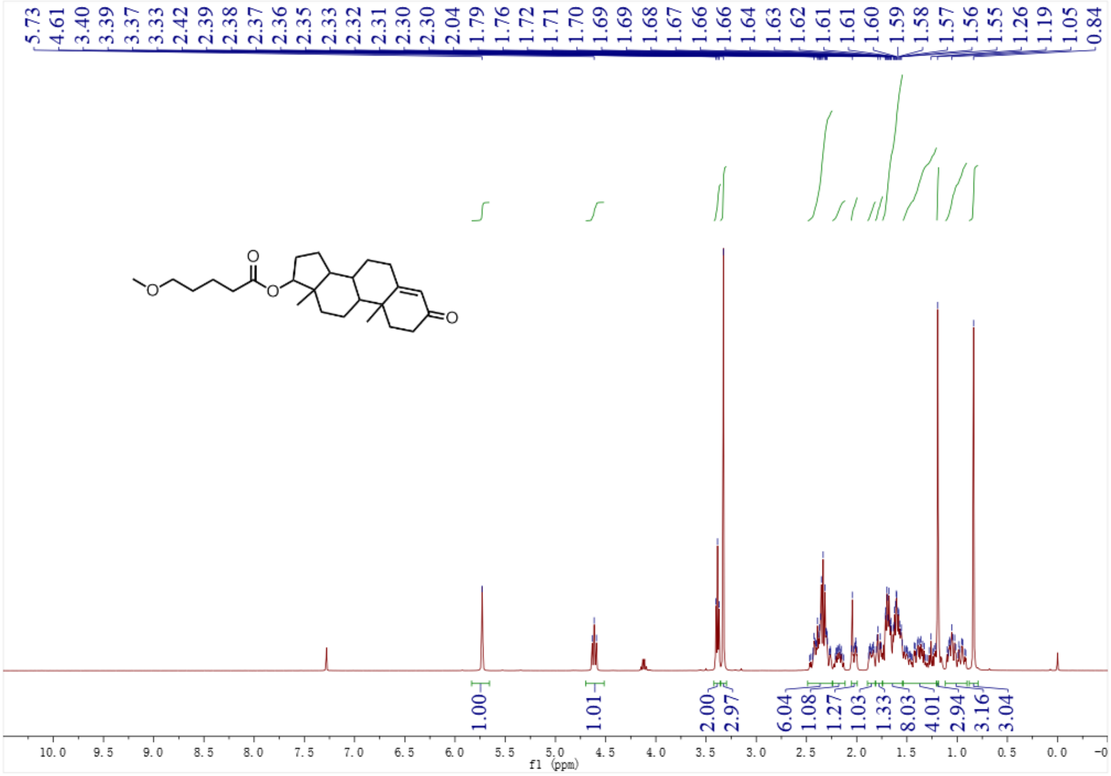

^{13}**C NMR** spectrum of **46** ($CDCl_3$)

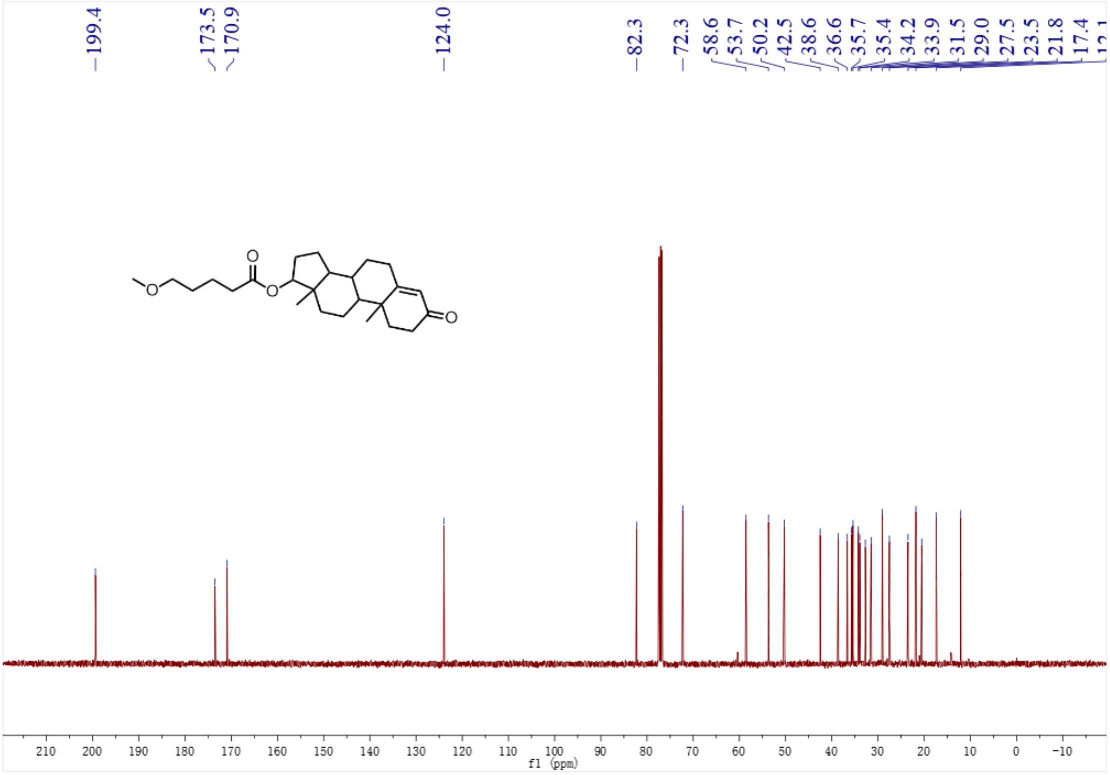

^{1}H NMR spectrum of **47** ($CDCl_3$)

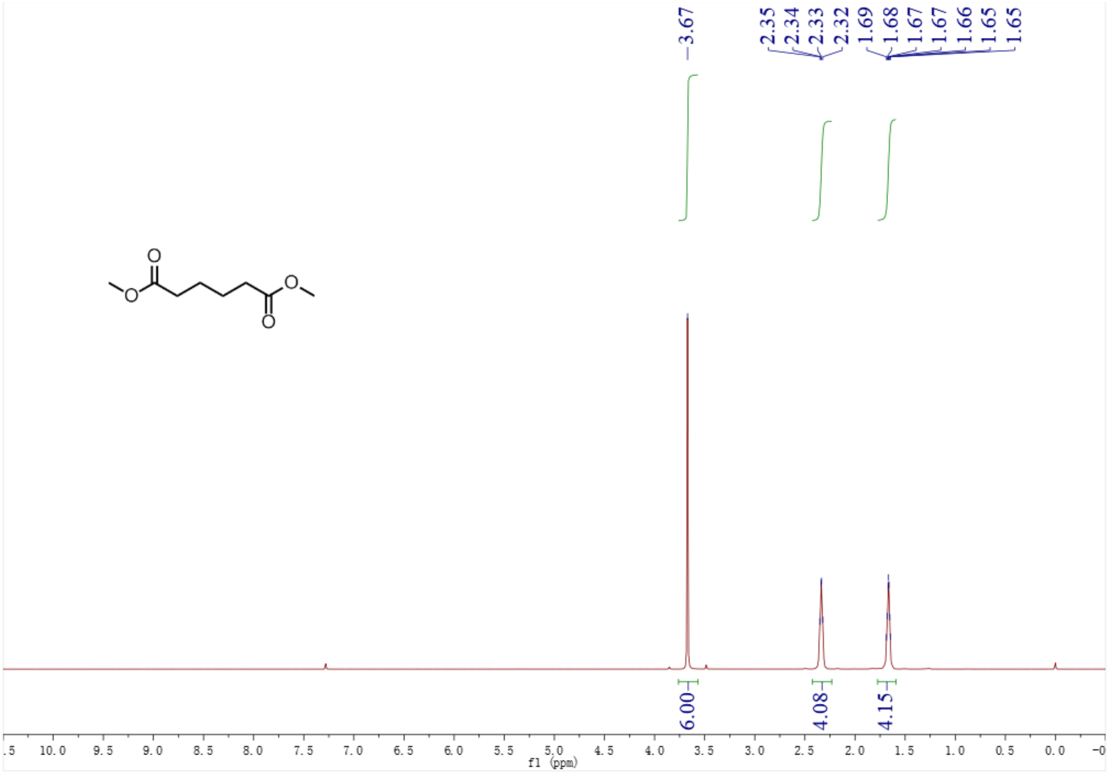

^{13}C NMR spectrum of **47** ($CDCl_3$)

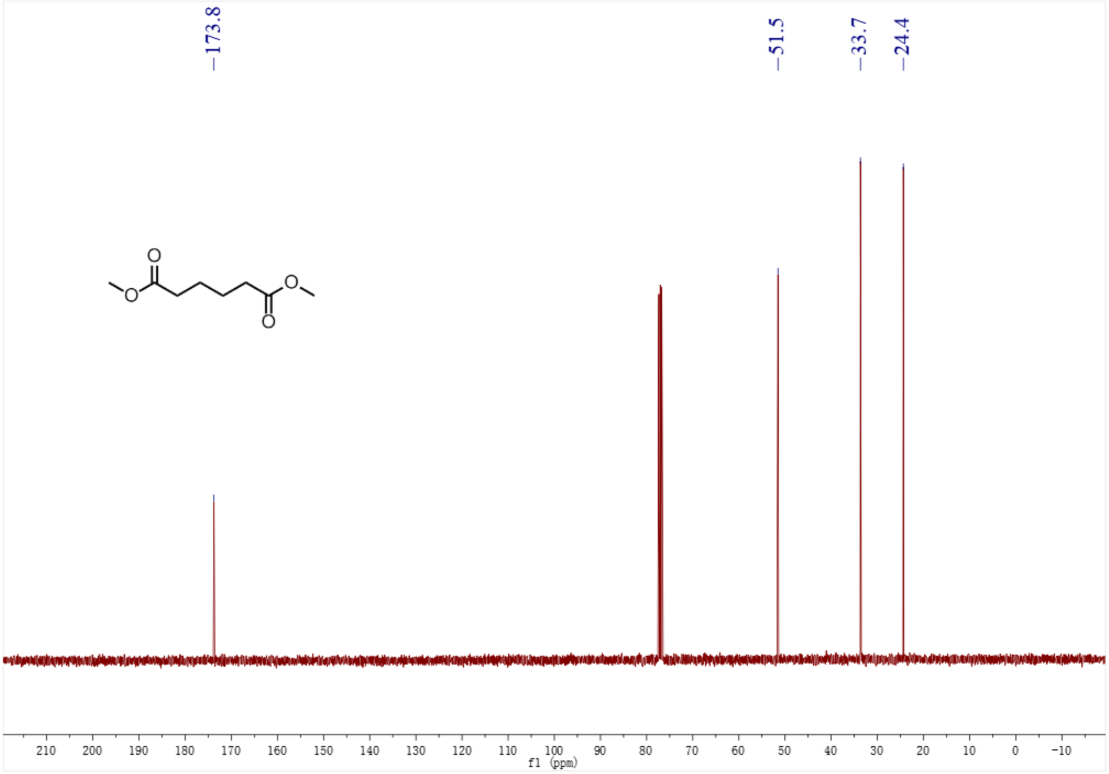

^{1}H NMR spectrum of **48** ($CDCl_3$)

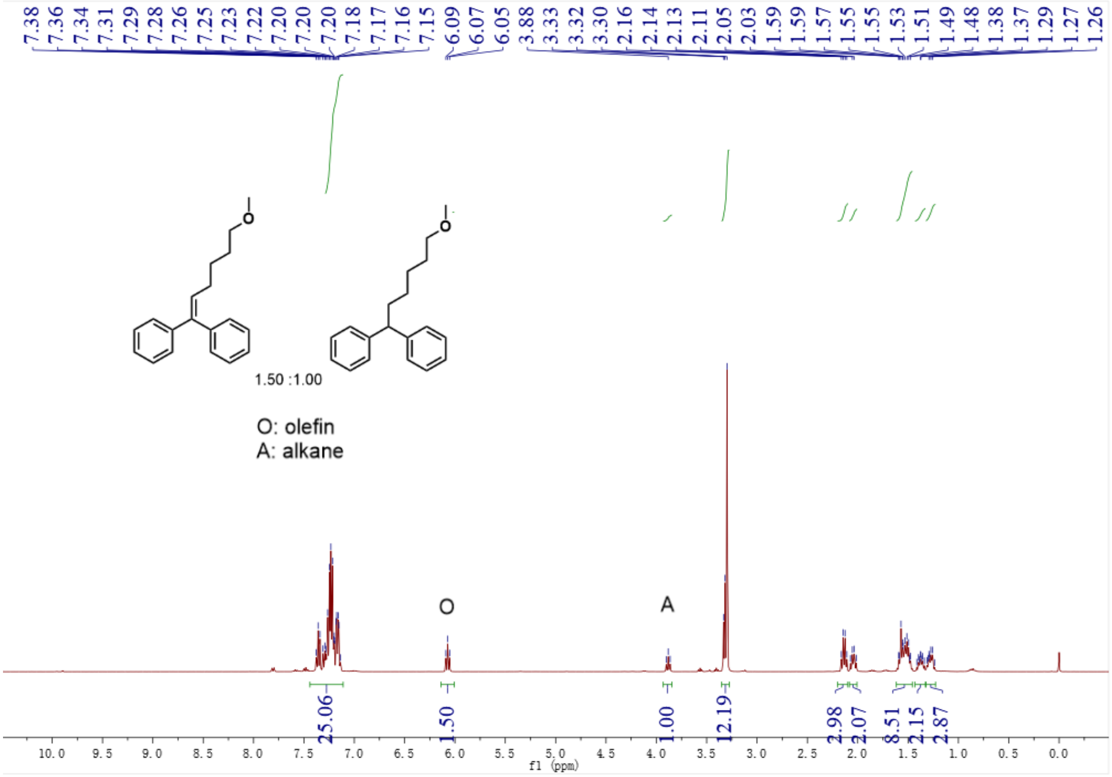

^{13}C NMR spectrum of **48** ($CDCl_3$)

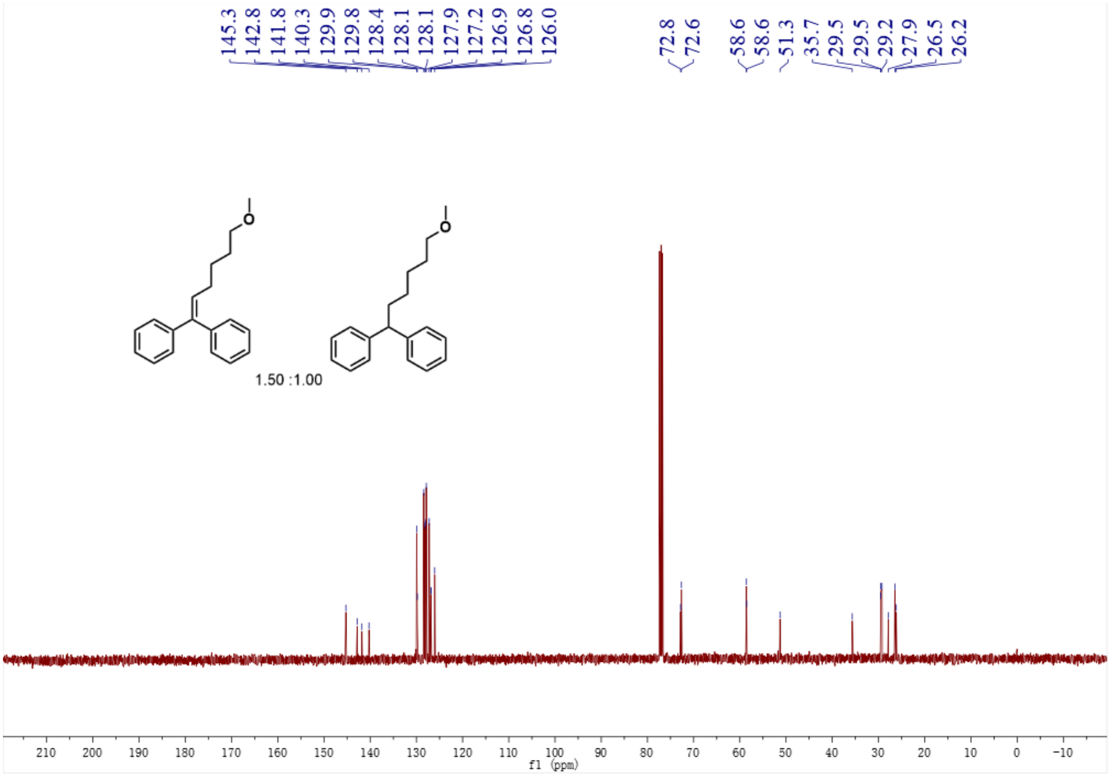

^{1}H NMR spectrum of **49** and **50** ($CDCl_3$)

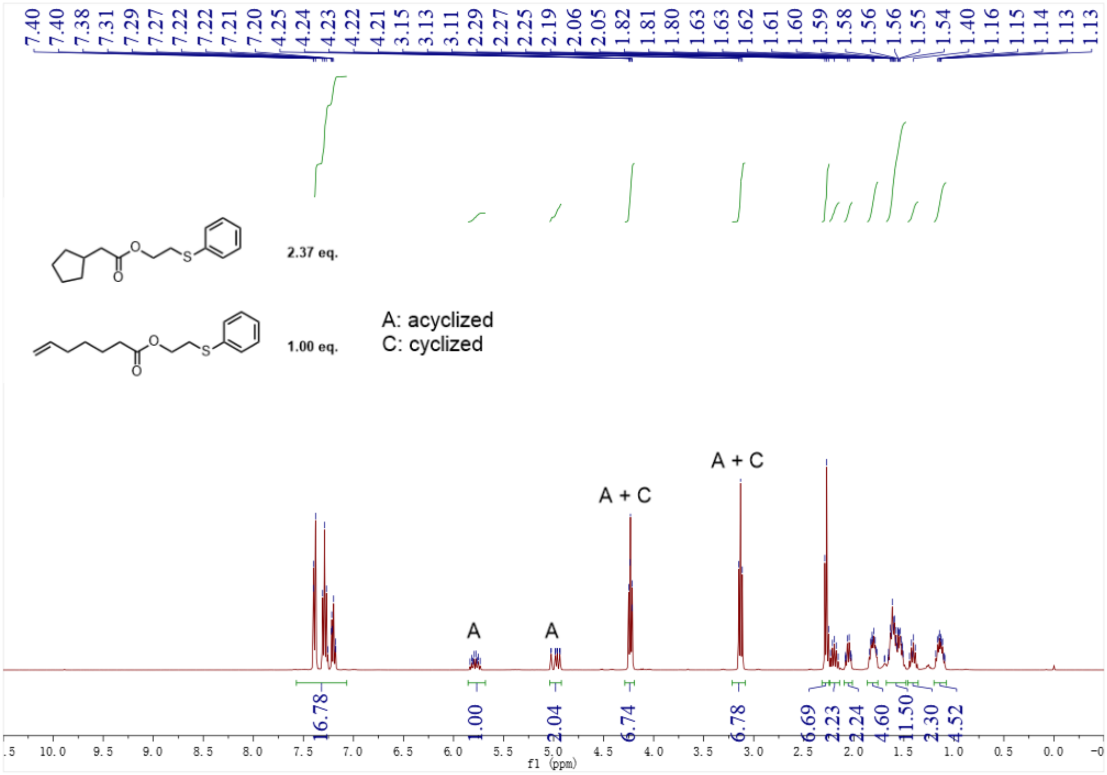

^{13}C NMR spectrum of **49** and **50** ($CDCl_3$)

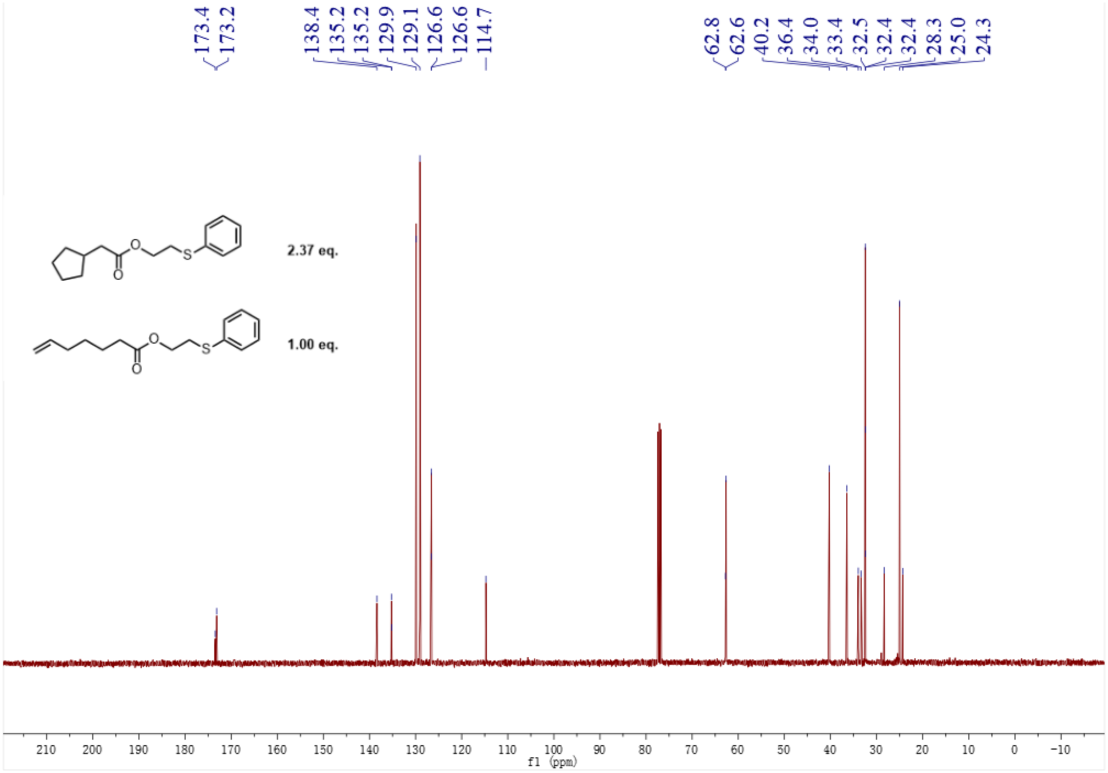

^{1}H **NMR** spectrum of **51** ($CDCl_3$)

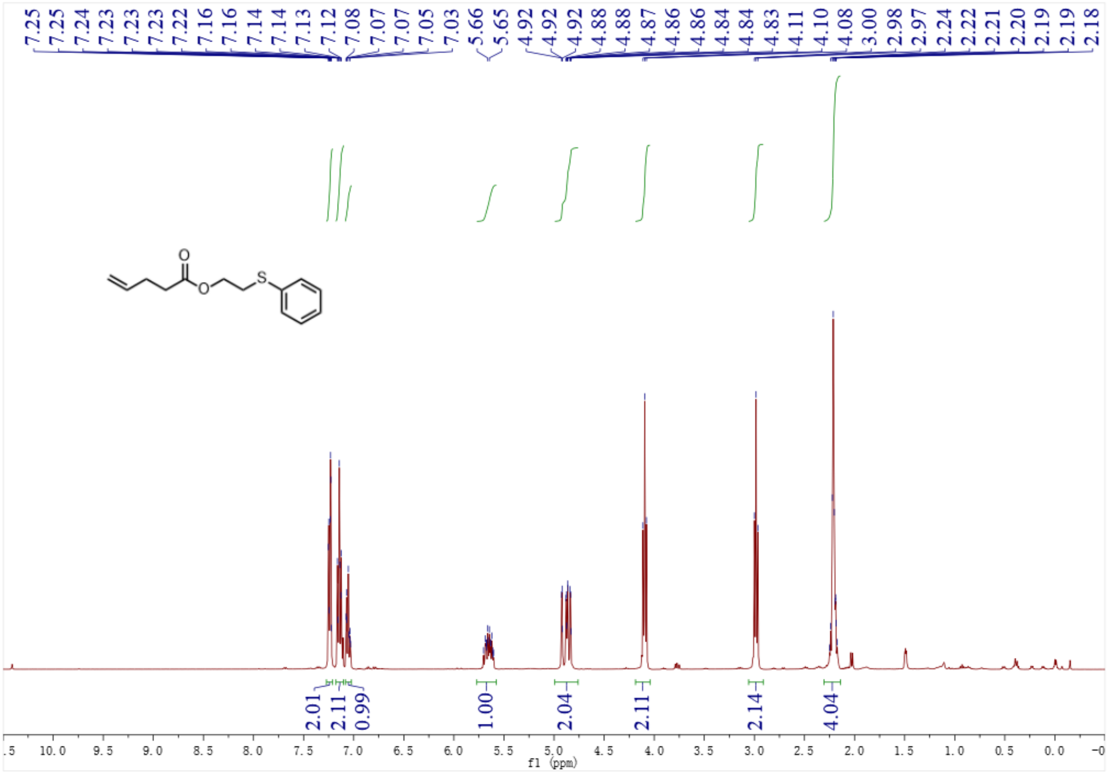

^{13}C **NMR** spectrum of **51** ($CDCl_3$)

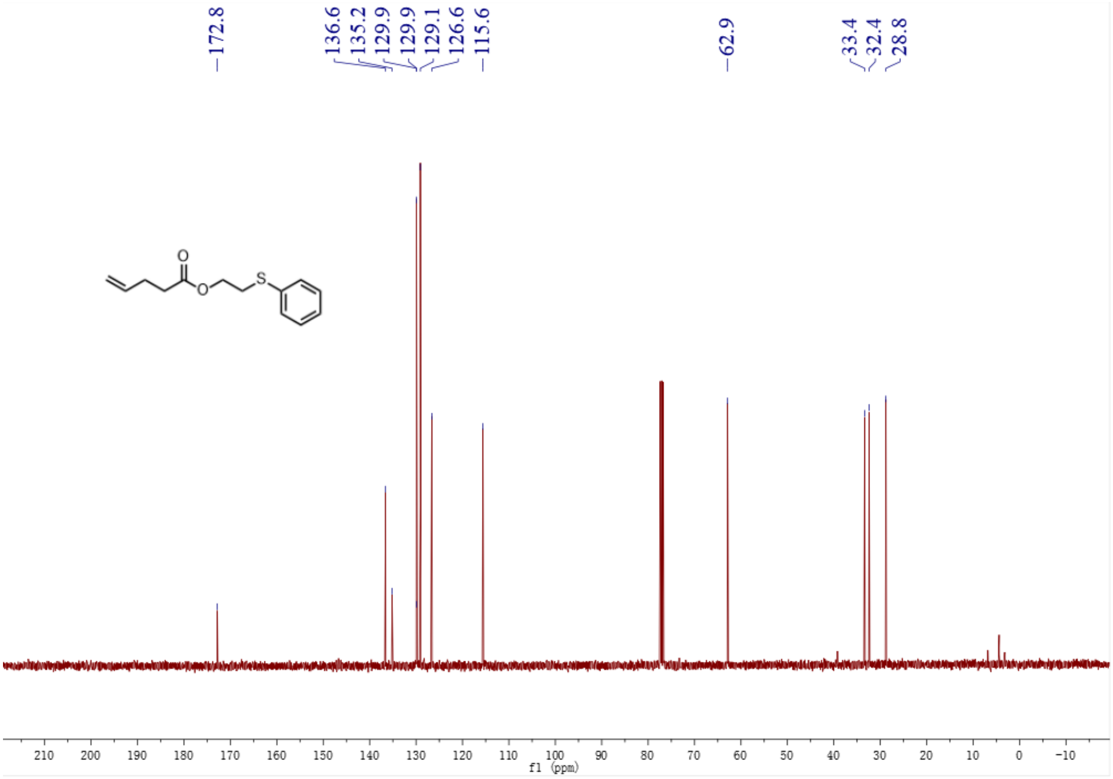

^{1}**H NMR** spectrum of **52** ($CDCl_3$)

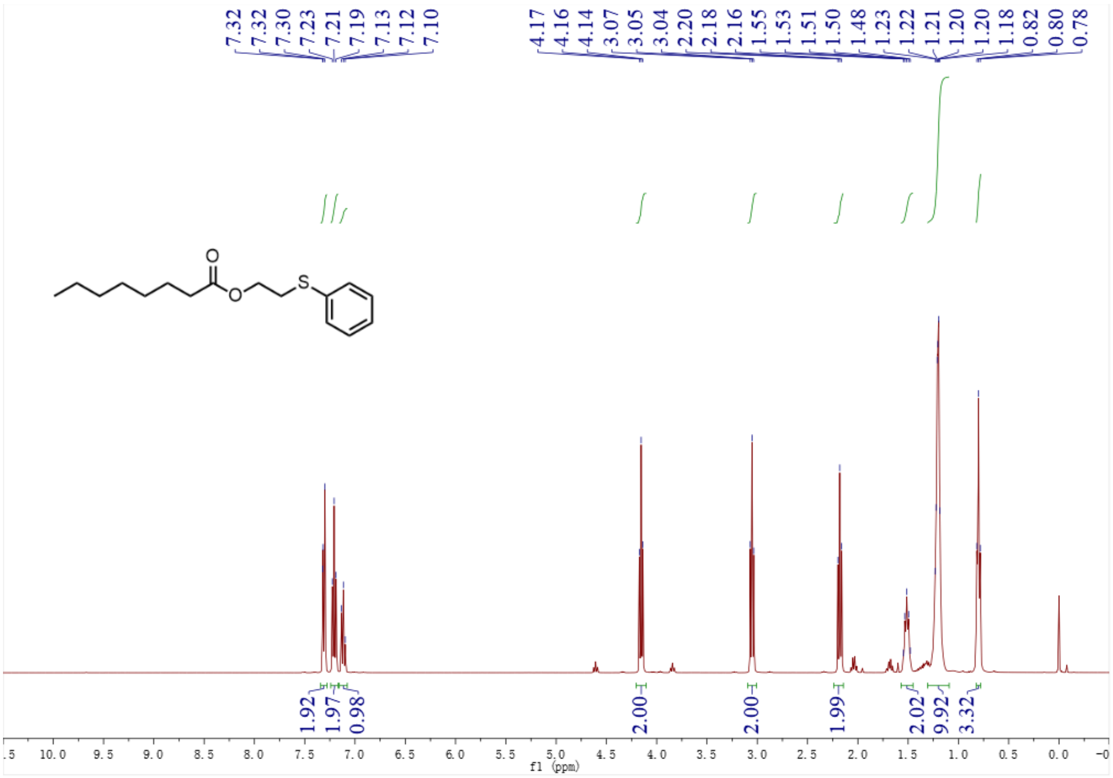

^{13}**C NMR** spectrum of **52** ($CDCl_3$)

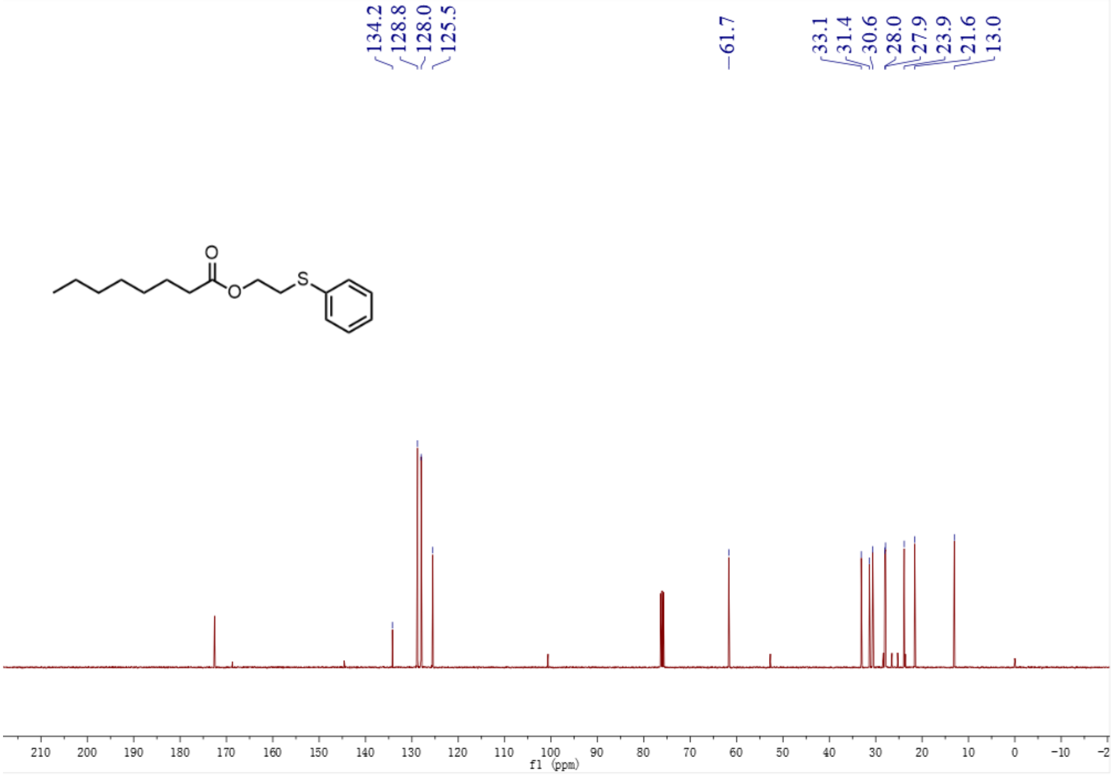

[1]**H NMR** spectrum of **53** ($CDCl_3$)

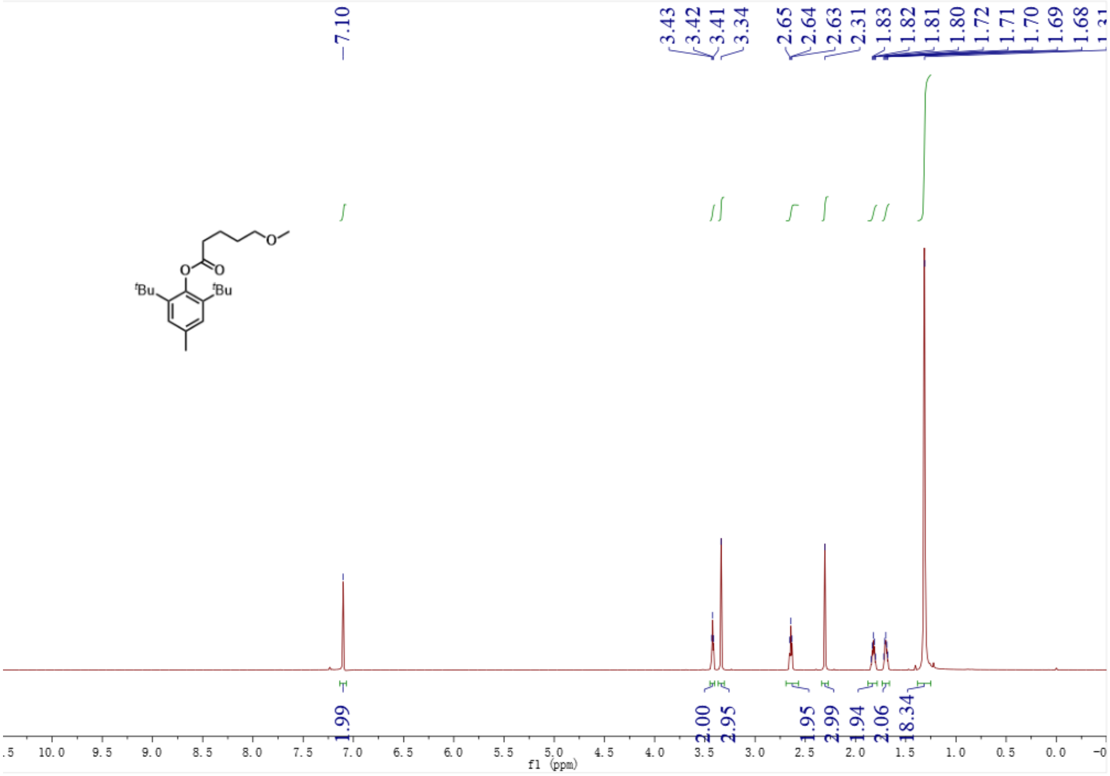

[13]**C NMR** spectrum of **53** ($CDCl_3$)

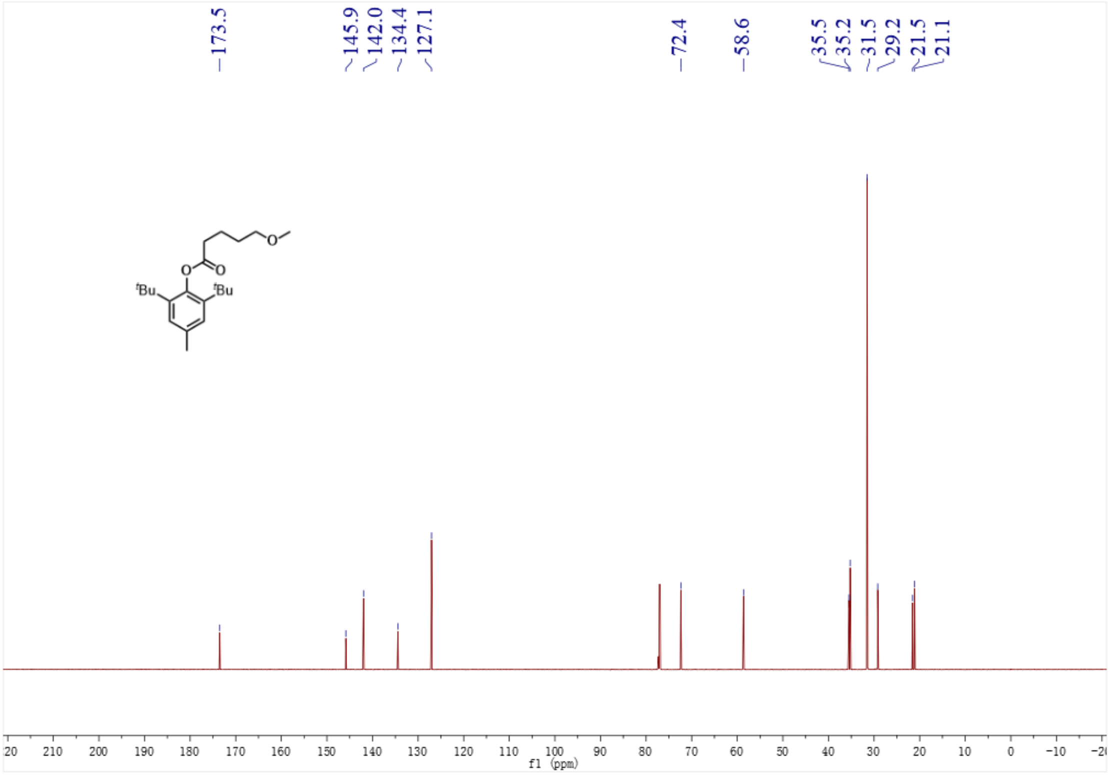

^{1}H NMR spectrum of **54** ($CDCl_3$)

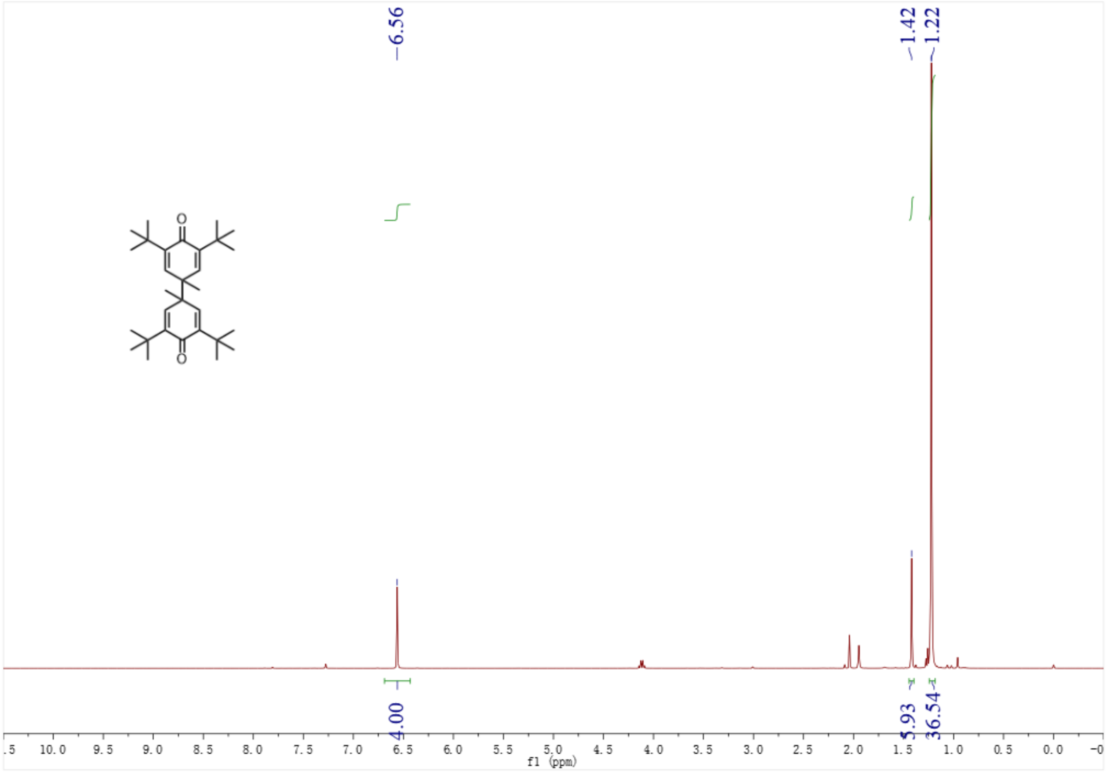

^{13}C NMR spectrum of **54** ($CDCl_3$)

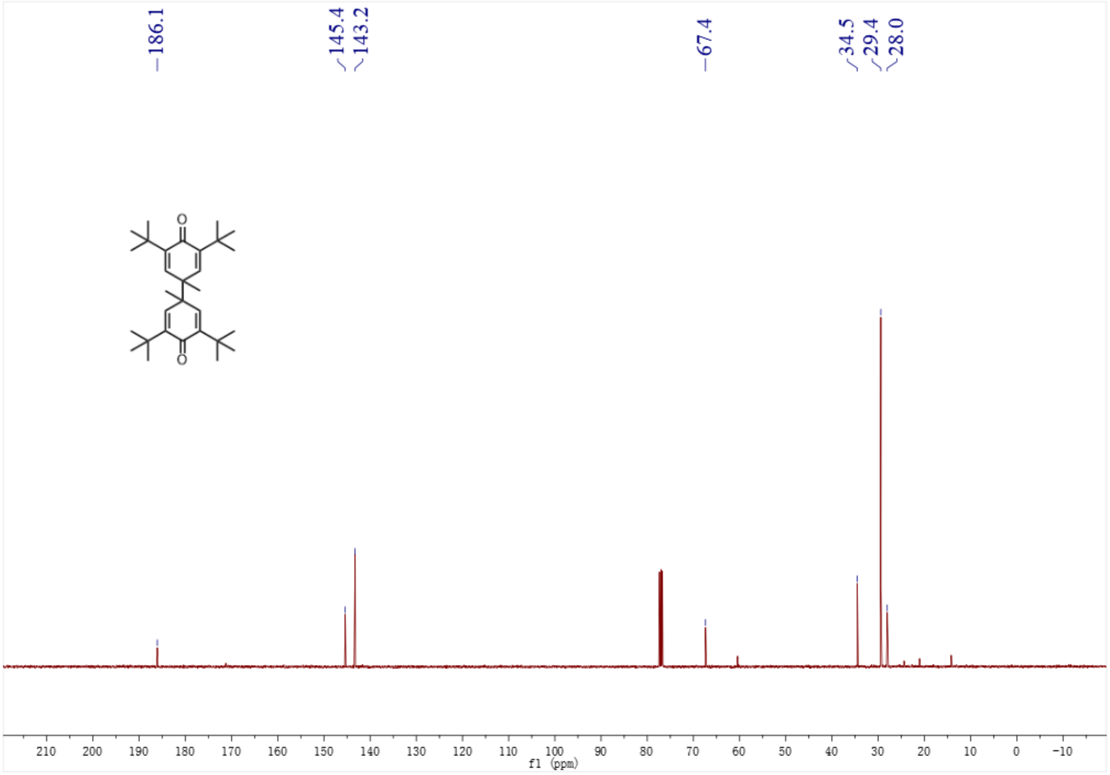

^{1}**H NMR** spectrum of **Co-A** (DMSO-d_6)

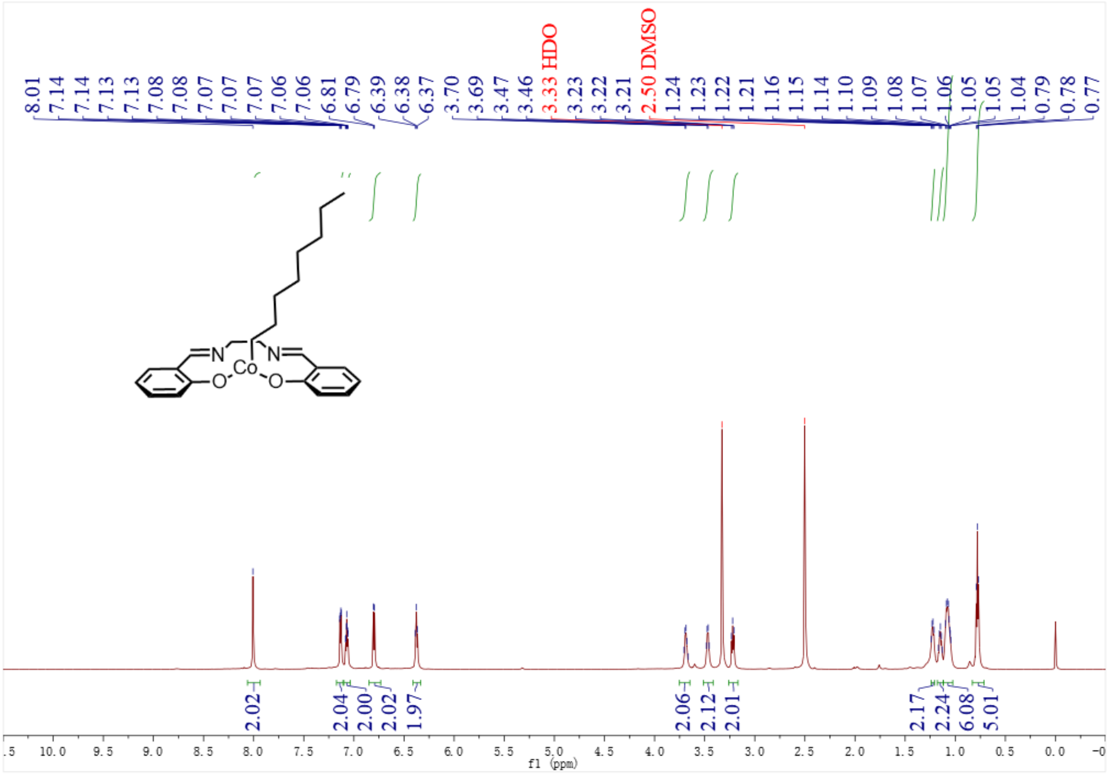

^{13}**C NMR** spectrum of **Co-A** (DMSO-d_6)

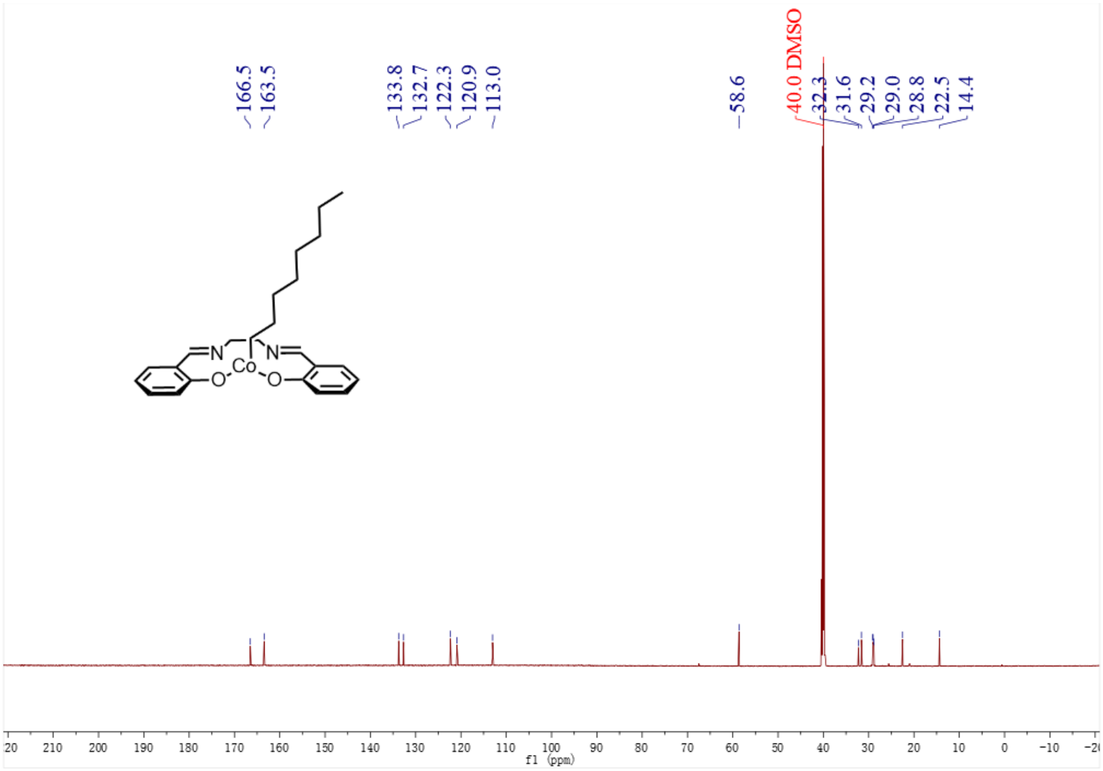

^{1}**H NMR** spectrum of **Co-B** (DMSO-d_6)

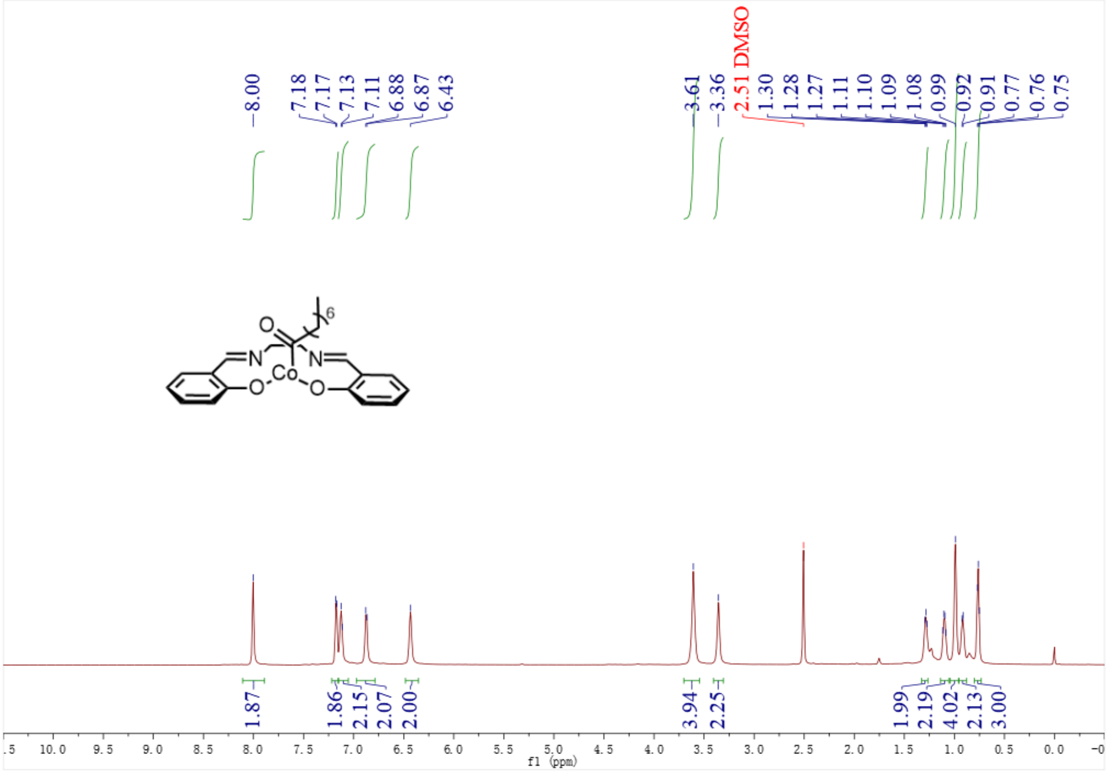

^{13}**C NMR** spectrum of **Co-B** (DMSO-d_6)

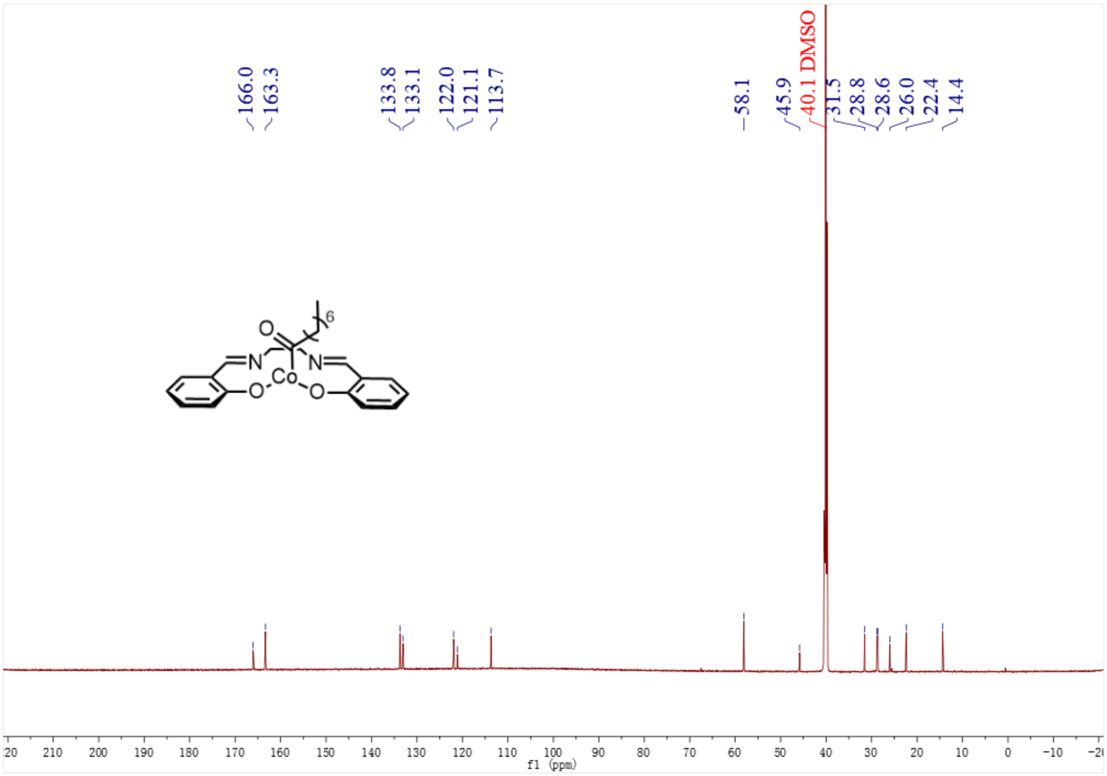

[1]**H NMR** spectrum of **Co-C** (DMSO-d_6)

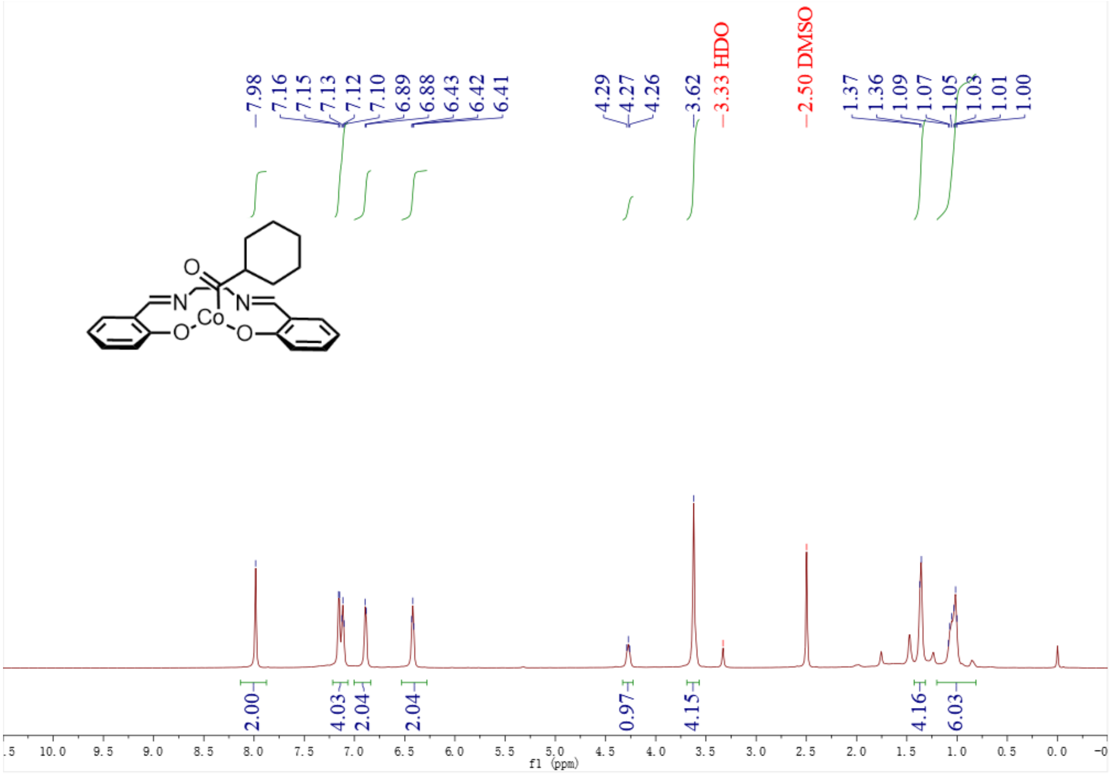

[13]**C NMR** spectrum of **Co-C** (DMSO-d_6)

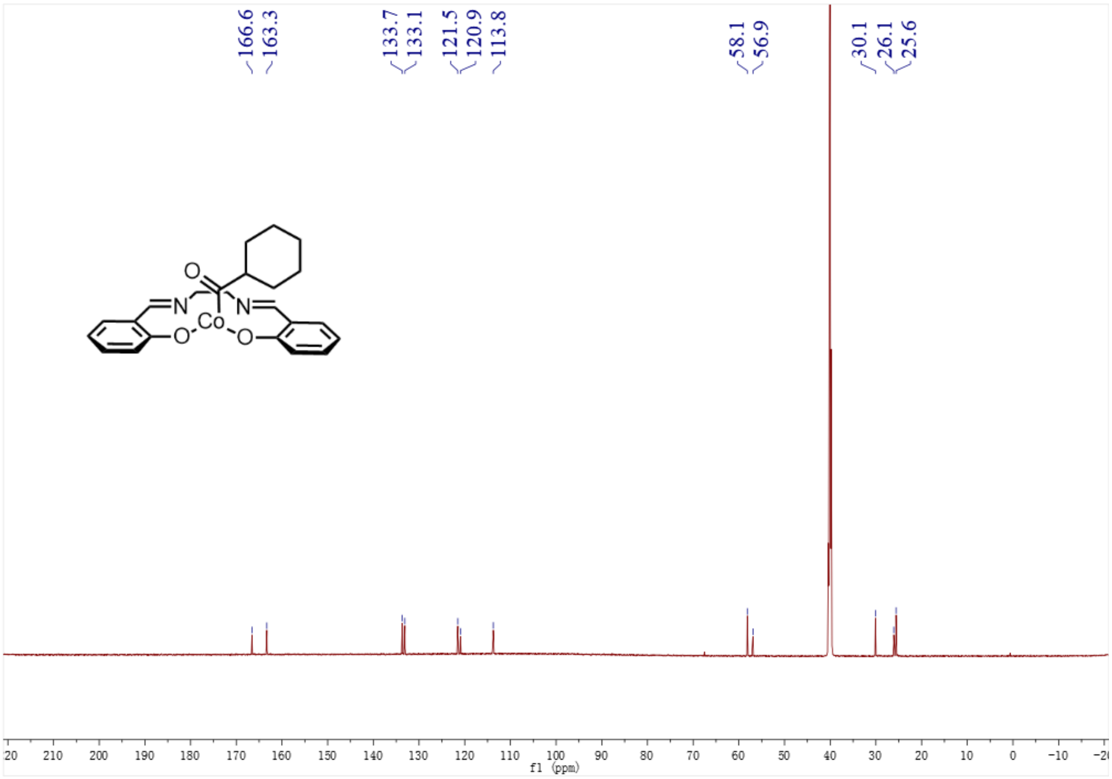

Supplement: Supplementary file 1 [file ja6c03507_si_001.pdf]
